# A latent reactive handle for functionalising heparin-like and LMWH deca- and dodecasaccharides†
†Electronic supplementary information (ESI) available: Details of experimental procedures not contained in the main text and also ^1^H, ^13^C, COSY, HMQC/HSQC NMR and MS data for compounds **2–27**. CCDC 998097–998099. For ESI and crystallographic data in CIF or other electronic format see DOI: 10.1039/c5ob01706h



**DOI:** 10.1039/c5ob01706h

**Published:** 2015-12-14

**Authors:** Gavin J. Miller, Karl. R. Broberg, Claire Rudd, Madeleine R. Helliwell, Gordon C. Jayson, John M. Gardiner

**Affiliations:** a Manchester Institute of Biotechnology and School of Chemistry , 131 Princess Street , University of Manchester M1 7DN , UK . Email: gardiner@manchester.ac.uk ; Tel: +44 (0)161 306 4530; b School of Chemistry , University of Manchester , Manchester , UK M13 9PL; c Institute or Cancer Studies and Christie Hospital , University of Manchester , Manchester , UK M20 4BX

## Abstract

d-Glucosamine derivatives bearing latent O4 functionality provide modified H/HS-type disaccharide donors for a final stage capping approach enabling introduction of conjugation-suitable, non-reducing terminal functionality to biologically important glycosaminoglycan oligosaccharides. Application to the synthesis of the first O4-terminus modified synthetic LMWH decasaccharide and an HS-like dodecasaccharide is reported.

## Abstract 2

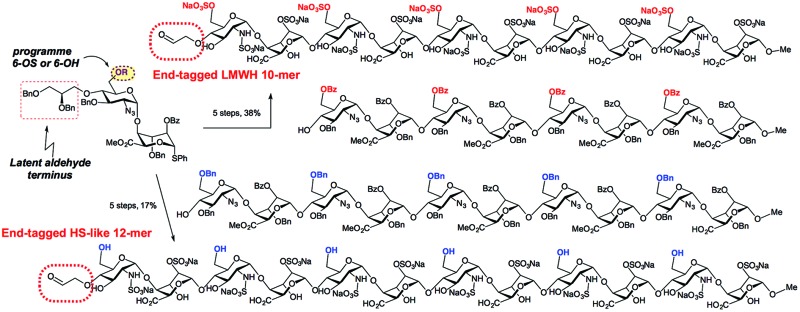
Disaccharide units containing a latent aldehyde surrogate at O4 provide late-stage access to terminal aldehyde LMWH and HS deca and dodecasaccharides.

## Introduction

The glycosylaminoglycan (GAG) family of carbohydrates constitute the most diverse group of biological carbohydrates. They are pervasive on cell surfaces and within the intra-cellular matrix, playing key roles in mediating regulatory functions relevant to numerous biomedical areas, such as cancer, inflammation, Alzheimer's disease, viral infectivity, and stem cell differentiation.^1^ Heparin and heparan sulphate (H/HS) are key members of this GAG family of sugars. The complex heterogeneity of the natural oligosaccharides requires syntheses of structurally-defined HS sequences to be able to evaluate the roles of HS substructures, which underpins the potential for ultimate therapeutic applications of synthetic fragments. Such synthetic endeavours have seen a number of important advances in recent years, targeting both anti-coagulant LMWH-replacements,^2^ but wide interest is directed at the diversity of bio-regulatory roles different synthetic heparin-like oligosaccharides could target.^3^

As part of our program to provide scalable syntheses of structurally-defined H/HS oligosaccharides, encompassing the first reported [GlcN-IdoA]_*n*_ 12-mer LMWH^4^ and gram-scale syntheses of heparin-like 12-mers,^5^ we have also been developing synthetic HS mimetics bearing a site-specifically located functional handle, to provide new GAG tools for chemical biology and biophysical applications of homogenous oligosaccharide conjugates.

Accessibility to new synthetic oligosaccharides bearing functional end groups is highly relevant to the viability of applications to microarray analysis, fluorescent labelling and other biomolecule or nanoparticle conjugations, essential for interrogating biological structure/function effects. Chemical modifications generating new carbohydrate tools are important, not only for the glycosaminoglycan field,^6^ but for wider potential applicability within the chemical glycobiology community.^7^

End-modification methods for native linear poly- and oligosaccharides, including GAGs, traditionally employ reductive amination chemistry at the expense of opening the reducing terminal sugar.^8^ Conjugation-amenable modifications which do not require a free reducing terminus are important. Modifications *via* the glycosidic terminus have become the default strategy for glycoside conjugation capabilities for GAGs and other oligosaccharides. Most of these approaches introduce glycosidic extensions bearing reactive amine, thiol, alcohol, azide or alkyne functions for further modifications. In the GAG and wider saccharide arena, these have seen valuable applications to arrays and other immobilizations,^9^ and recent end modifications to assist purification and thereby accelerate synthesis, such as through use of ionic liquid^9*e*^ or fluorous^9*f*^ tags. Invariably, the reducing terminus is employed for C1 modification *via O*-, but also *C*-, *N*- or *S*-glycosides.

**Fig. 1:**
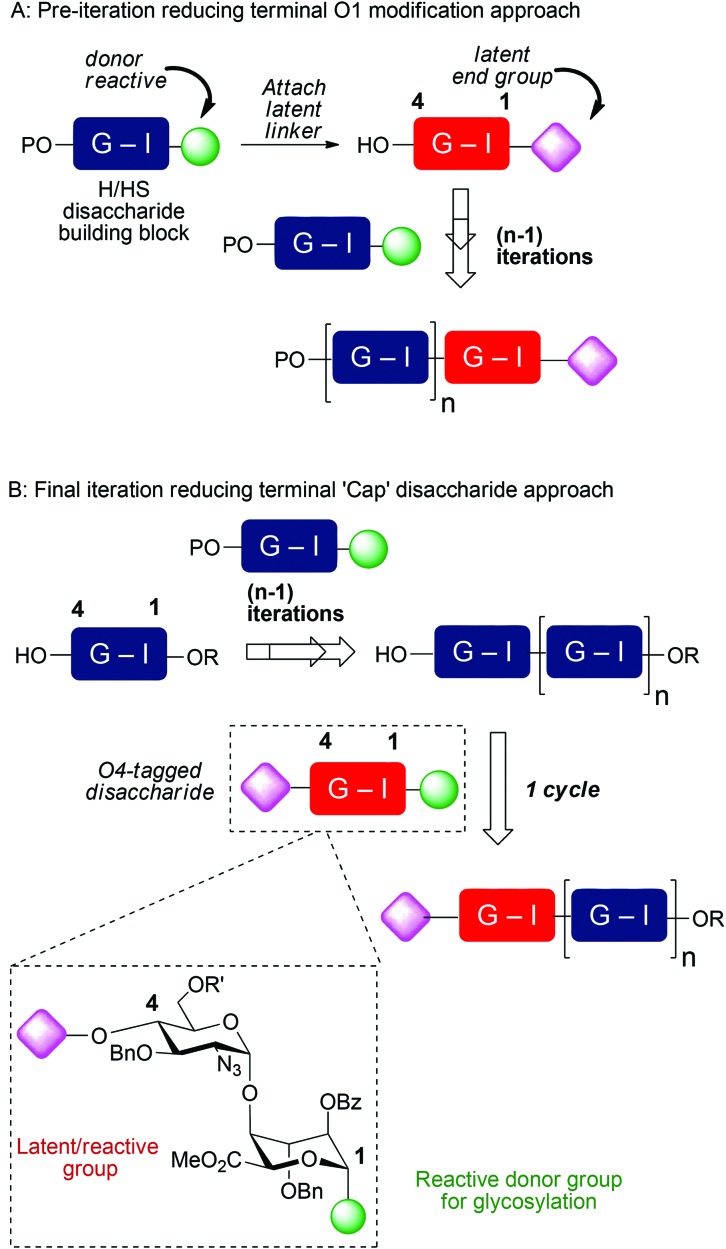
A: Reducing terminal *vs.* B: final stage capping approaches to introduce functional end groups into HS-like oligosaccharides, R = Me, R′ = Bz or Bn.

Though of high biological and biomedical importance, the challenges presented by chemical synthesis of longer synthetic heparins has limited approaches to end-modifications. A reducing terminal labelling approach *via* disaccharide iteration (Fig. 1A) requires that the conjugation site or precursor is introduced early and is followed by all the sequence iterations. Use of reactive aldehyde end tags can be envisaged as an alternative to use of a free reducing terminus (aldehyde) by installing the aldehyde on a linker, and though little exploited, has also seen use for amination-based facile dimerization of disaccharides.^10^ We recently reported introduction of a reducing end latent aldehyde tag (LAT) into a synthetic HS-dodecasaccharide and exploited this for ^3^H-radiolabelling, enabling the first example of determination of *in vivo* tissue distribution and metabolism of a pure, biologically-active heparin-like oligosaccharide (c.f. Fig. 1A).^11^ This reducing terminal modification approach was considerably facilitated by the high efficiency and fidelity of a new 4-mer based iteration 12-mer synthesis to two 2-step cycles, however, in this case still, the latent conjugation site (at the reducing terminus) is included from the beginning of the iterations.

Non-reducing terminal conjugation units would obviate the limitation of early-stage introduction of the commonly-employed O1-glycosidic based conjugate-ready modifications for iterative oligosaccharide synthesis and would be anticipated to deliver increasing benefits for longer heparanoids. In such approaches, all but the final iteration step would precede introduction of the latent conjugation site (Fig. 1B). Following our 12-mer HS end-labelling work, we therefore sought to investigate the utility of attaching such a LAT, or similar derivative, to the non-reducing terminus of heparanoid oligosaccharides. This has not been evaluated or exploited for the functional modification of longer, bio-relevant heparin-types or other GAGs. This would be valuable both for H/HS targets, but we envision such end modification would be portable to application in other important GAGs, of different constituent sequences. Given the known biological roles of O6 and O3 D-GlcN functionality in H/HS systems, this would most ideally be located at the O4 terminal position (Fig. 1B). A non-reducing terminal strategy could seek to modify the terminus post-oligomerization, *via* coupling to a native terminal hydroxyl unit which could be methodologically challenging. Instead, providing new generic end-modified non-reducing ‘capper’ units (*e.g.* disaccharides) would introduce a terminal, non-reducing end building block (Fig. 1B) bearing the latent conjugation site in the final iterative glycosylation step during oligosaccharide assembly. This convergent strategy would be attractive as it would enable late-stage diversification at the terminus of different acceptor saccharides and accelerate accessibility to HS-sequences of different constituents or containing mixed sulfation patterns, in a parallel manner.

Introducing this to our [GlcN-IdoA]_*n*_-based HS oligosaccharide synthetic strategy requires attaching a conjugation-suitable tag containing a terminal reactive group (or latent reactive group) at the d-GlcN-O4-position. Ideally, this would be incorporated into a GlcN-IdoA disaccharide donor unit, two variants of which we have shown are effective iduronate donors, whose coupling rections are routinely high yielding and highly anomerically selective, on even the longest synthetic heparin-like acceptors.^4,5,12^

Such O4 modification could be approached by attaching the protected handle either directly at disaccharide level or onto the monoglucoside, which could then be coupled with an l-idoA acceptor, providing reagent disaccharides.

Prior examples of d-GlcN derivative O4 alkylations and related modifications include a 4-position formyl moiety (*via* reduction of a 4-cyano species),^13^ ethanolamine based linkers for introducing BODIPY fluorophores,^14^ diethylene glycol derivatives for azide-based linkers,^15^ thiol terminated alkyl spacer units (*via* thiol addition to *O*-allyl)^16^ and carboxylate terminated linkers.^17^

## Results and discussion

We report here approaches to introduce different oxidation level end groups, namely a carboxylate or an aldehyde surrogate, directly onto a d-GlcN-O4 derivative with an anomeric donor group in place (thiophenyl). Both these functional groups would facilitate further complementary conjugation chemistries. Our strategy was to introduce the O4 unit into suitable d-GlcN monosaccharide derivatives and to then couple to an iduronate acceptor as a direct route to end-modified ‘capper’ disaccharides. We also sought to evaluate such O4 modification *via* a pre-prepared disaccharide.

### Alkylation of d-GlcN-O-4 derivatives with carboxylate-bearing tethers

**Scheme 1:**
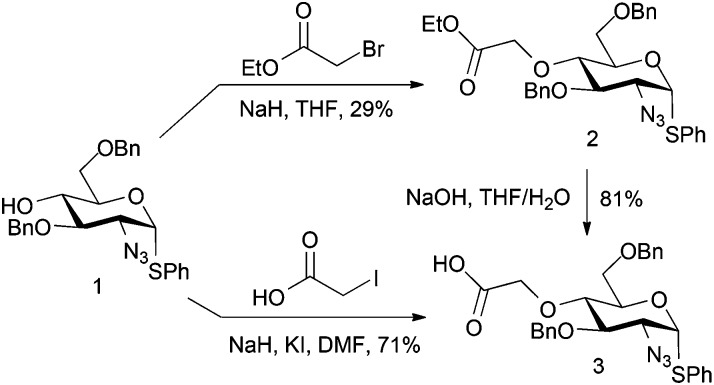
d-GlcN O4 modifications with activated carboxylate/ester electrophiles.

A first strategy considered introducing an O4 tag bearing a carboxylate, envisaging ultimate late-stage applications with suitably reactive labelling nucleophiles. Whilst a suitable ester differentiation for the iduronate might prove necessary, we reasoned that differential reactivity of a spacer-linked carboxylate over the l-IdoA 2-carboxylate could be evaluated or effected by use of a differentially labile terminal ester. Ethylbromoacetate was first employed as a model electrophile for d-GlcN O4-alkylation. Reaction of ethylbromoacetate with the O4 alkoxide salt of glucosamine derivative **1** ^18^ (Scheme 1) afforded the target ester **2**, but in relatively poor yield, owing to degradation of the starting material. However, with novel **2** in hand, saponification to the carboxylate derivative **3** proceeded in 81% yield. A more efficient one step route from **1** to **3** was subsequently effected using alkylation direct with iodoacetic acid in DMF, affording the acid **3** in significantly better overall yield (71%). If advancing this type of linker, a modified l-IdoA ester would thus be the candidate for any subsequent disaccharide formation.

**Scheme 2:**
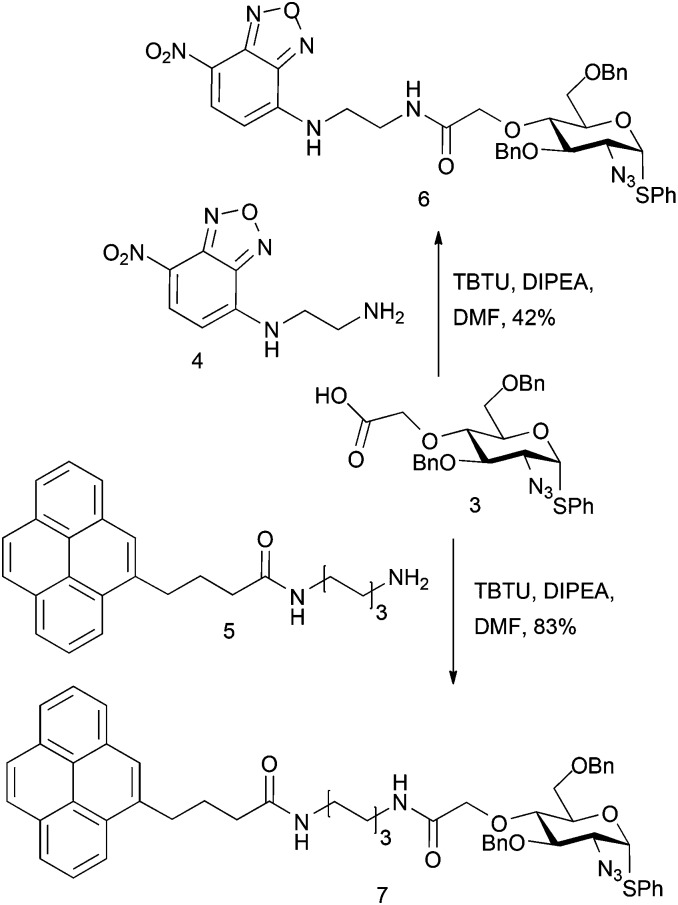
Fluorescently tagged glucosamine derivatives **6** and **7**.

Carboxylate **3** was utilised to evaluate model attachment of two fluorescent units, **4** ^19^ and **5**,^20^ which contained differing length C2 or C6, diaminoalkyl linker units, offering a divergent access to fluorescently tagged d-GlcN building blocks **6** and **7** (Scheme 2). Coupling of **4** or **5** with **3** using standard amide bond forming reagents afforded the fluoro-tagged d-GlcN derivatives **6** and **7**, respectively. This provides evidence for the potential viability of similar derivatization of d-GlcN embedded in longer chains. This approach also envisaged that such novel O4-carboxylate-bearing d-GlcN species could be precursors to extensions with other latent tag units *via* amide linkages.

We sought to evaluate whether this approach could be applied to the construction of an end-labelled HS-like disaccharide. This would establish the potential to use a pre-labelled HS disaccharide capper unit. Thus, the coupling of NBD-monosaccharide derivative **6** with l-iduronate acceptor **8** was attempted using NIS (1.5 equiv.) and AgOTf (0.1 equiv.).

**Scheme 3:**
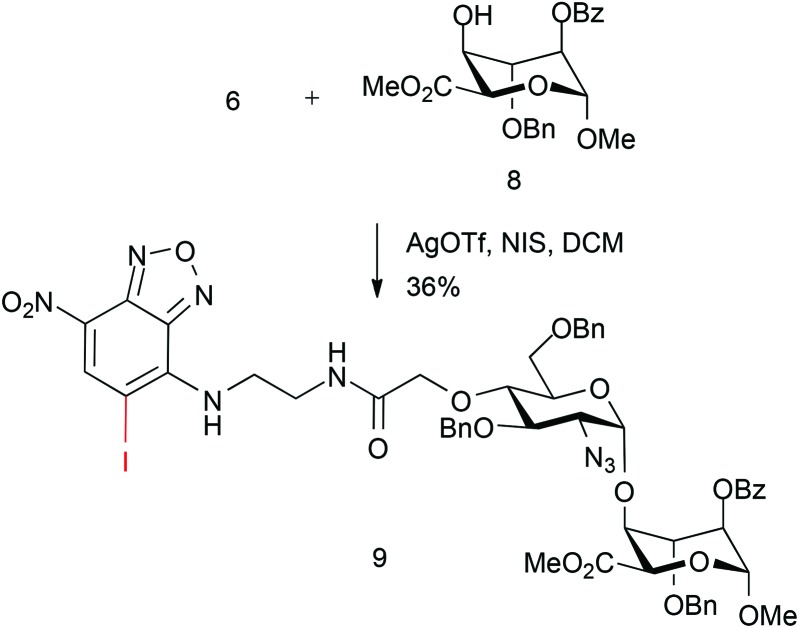
Fluorescently tagged disaccharide synthesis.

However, whilst disaccharide coupling occurred to form **9**, this was in modest yield, with significant amounts of acceptor recovered. Further inspection of the MS data showed a surprising concomitant iodination of the electron-deficient NBD ring (Scheme 3).

Attempts to couple pyrene-labelled glucoazide donor **7** with l-iduronate acceptor **8** returned hydrolysed donor and unreacted acceptor from the reaction mixture, under the same conditions employed for **9**. This suggested a poor capability of **7** as a glycosyl donor and, coupled with the unexpected glycosylation reactivity of **6**, suggested that a carboxylate-based approach would require further development to be viable.

Our alternative approach was *via* a d-GlcN O4-aldehyde-level bearing unit, which would remain latent throughout synthetic mono-, di- and oligosaccharide construction. This would parallel our O1-linked LAT approach and enable a final stage aldehyde unmasking on the complete sulphated heparin-type oligosaccharides.^21^ This would also obviate the need to carry a fluorescently labelled system throughout the remainder of the synthetic transformations to a complex HS target, which might have been necessary, if selective O4-ethanoate coupling were to prove problematic over internal l-IdoA carboxylate reactivity.

### Alkylation of d-GlcN derivatives with latent aldehyde O-4 tethers

**Scheme 4:**
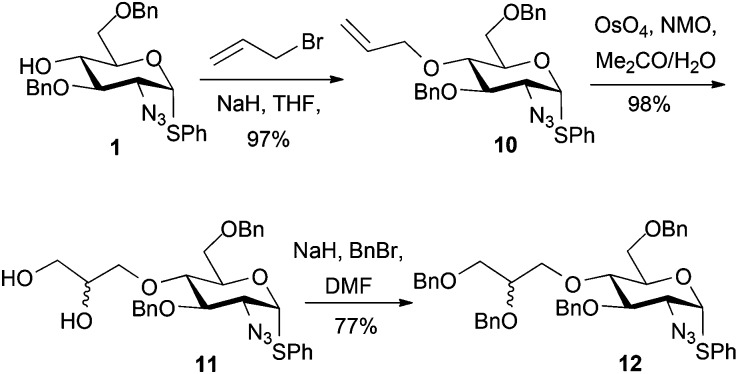
Synthesis of d-GlcN O4-glycidols 12 *via* allylation–oxidation.

Alkylation attempts of **1** with aldehyde- or protected aldehyde-containing electrophiles such as 3-bromo-1,1-dimethoxypropane, 4-bromomethylbenzaldehyde, 3-bromomethylbenzaldehyde and 2-(4-(bromomethyl)phenyl)-1,3-dioxolane were unsuccessful, mostly leading to no alkylation product and frequently to degradation of the starting d-GlcN derivative. We thus evaluated use of allylbromide to alkylate the O4 position of 1 with a view to using the derived alkene as an alternative precursor to a 1,2-diol aldehyde surrogate. Allylation of **1** was effected in 97% yield (Scheme 4) and subsequent OsO_4_-mediated dihydroxylation of **10** furnished diol **11** in 98% yield.

**Fig. 2:**
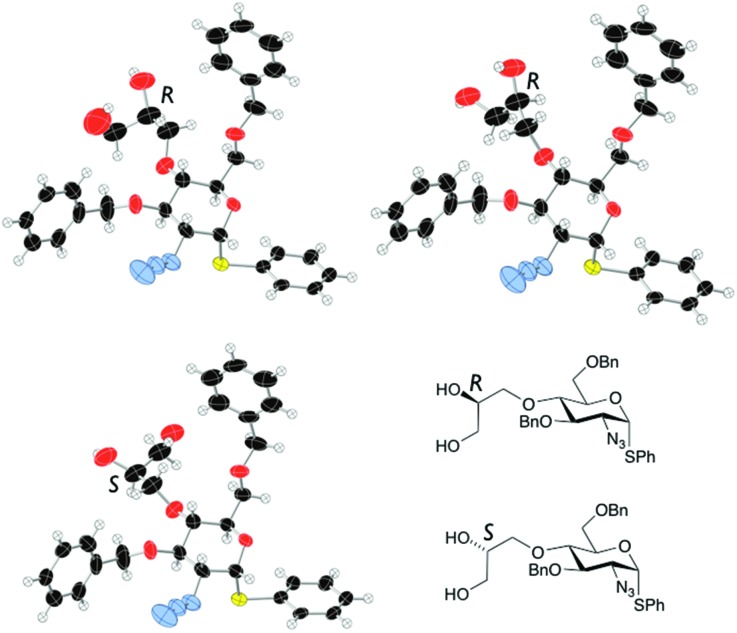
X-Ray crystal structure for diastereoisomers of diol **11** (major: *R* configuration in diol side chain; minor: *S*-configuration in side chain).

Double benzylation of **11** was completed in high yield to afford **12** with the required masked 1,2-diol unit installed at the O4-position. ^1^H NMR indicated that the penultimate dihydroxylation reaction to afford diol **11** yielded a mixture of diastereomeric diols (2 : 1, *R*/*S*). As the diol chiral centre is lost in the anticipated release of the aldehyde at the end of any labelling route, we envisaged this could be pursued using disatereomerically-mixed diol, once late stage analytical methods were in place. The crystal structure of **11** showed a 3 : 1 diastereomeric ratio (*R* : *S* configuration of the diol side chain) in the crystal, with a mixed packing of the two diastereomers (Fig. 2). In addition, the major (side chain *R*) diastereoisomer adopted two different conformations varying about several torsion angles in the O4 diol-bearing unit, whilst there are no significant differences in any aspects of the sugar ring or derivatives (such as the SPh or azide) on the ring, for either conformer or between diastereomers.

The monosaccharide building block **12** was suitable for glycosylation with an iduronate acceptor **19** and provided the required, iteration-ready, HS-like disaccharide building block in three steps and 19% overall yield in a manner directly analogous to that for diastereomerically pure **20** and **21** (*vide infra*).‡
‡Data not shown. Although it is not critical that the OsO_4_ mediated dihydroxylation of **10** did not proceed with complete diastereoselectivity (as stereochemistry lost on late-stage aldehyde release), we explored an alternative access to a diastereomerically-pure form of **12** to circumvent any analytical challenges of late stage oligosaccharide synthesis and characterisation using stereoisomeric mixtures, and to afford high purity final targets for analysis and structural proof.

**Scheme 5:**
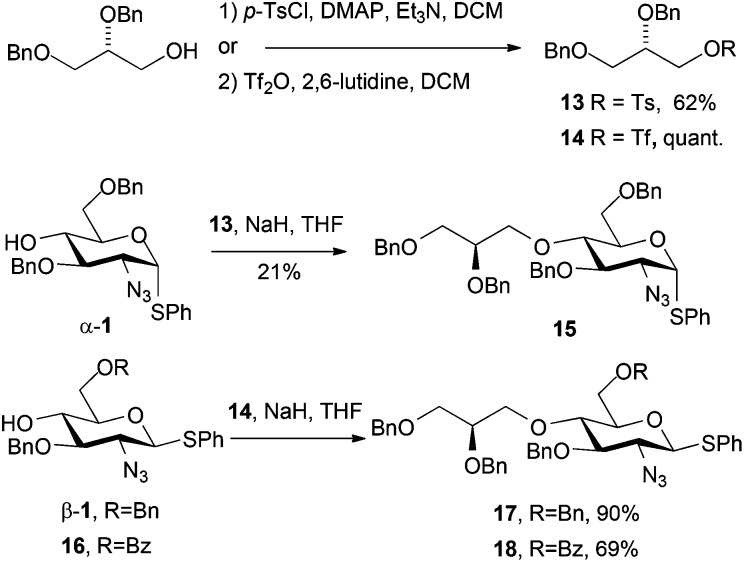
Diastereomerically pure *O*-glycidol-bearing d-GlcN derivatives **15–18**.

Having successfully employed a dibenzyl glycidol unit as an O1-LAT (*via* glycosylation)^11^ we sought to evaluate a similar unit introduced *via* O4 alkylation. This presented the same potential complications we had encountered in O4-alkylation with a number of unactivated electrophiles (*vide supra*).

(*S*)-2,3-Dibenzyloxy propanol was readily converted to its tosylate derivative **13** ^22^ and subsequent alkylation of **1** with **13** did proceed, but only in low yield, as the reaction required heating, and as we had seen with other O4 alkylations, resulted in competing starting material degradation (Scheme 5). To circumvent this, employing the corresponding triflate electrophile **14** ^23^ enabled efficient alkylation of O4 for two d-GlcN derivatives β-**1** and **16** (differentially protected at O6) in good to excellent yields. The slightly lower yield for the formation of **18** was attributed to a competing transesterification reaction, evidenced by formation of a species with very similar *R*_f_ to **18** as the reaction progressed. The reaction yield was improved by drop-wise addition of the electrophile at 0 °C.

Commercially available (*S*)-2,3-dibenzyloxypropanol is expensive (£100 per g). We thus developed a convenient 5-step route from d-mannitol (3 orders of magnitude cheaper than (*S*)-2,3-dibenzyloxypropanol), which readily delivered multi-gram quantities of the required alcohol (25% overall yield, 5 steps from d-mannitol, see ESI†).^24^ This enabled us to access the novel O4-LAT derivatized thioglyosides **17** and **18** in gram quantities and diastereomerically pure form. The inclusion of the differential O6 protection is important as this allows parallel access to backbone O6-sulfation pattern variations in any final HS-oligosaccharide targets.

With these materials in hand we then investigated their utility for synthesis of end-tagged HS-related di- and oligosaccharides.

### Synthesis of heparin-like di- and oligosaccharides bearing a functional non-reducing end tether

**Scheme 6:**
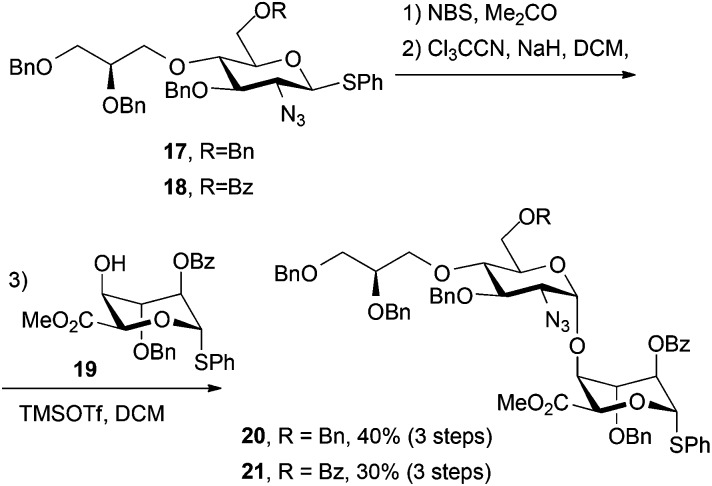
Synthesis of O4-end modified disaccharide donors.

Thioglycoside derivatives **17** and **18** were readily converted to their respective trichloroacetimidate donors in high yield, following NBS-mediated hydrolysis. Subsequent glycosylations with iduronate acceptor **19** afforded the expected high selectivity for the desired α-1,4 linkage, affording **20** (for H1′ *δ*: 4.61 ppm, d, *J* = 3.6 Hz) and **21** (for H1′ *δ*: 4.72 ppm, d, *J* = 3.6 Hz). Synthesis of both of these novel O4-modified disaccharides could be readily carried out on multi-hundred mg scale with the synthesis of **20** additionally scaled to gram level (Scheme 6). In each case iduronate accepter **19** was recovered (around 30% for **21** and 35% for **20**) following chromatography, suggesting a lower reactivity of the donor thioglycoside in these systems, compared to direct analogues lacking the modified O4-unit.

We attempted the direct alkylation of an analogue of disaccharide **21**, containing a free 4-OH at the non-reducing end terminus, with triflate **14**, but no product was observed. With recovery of iduronate acceptor in disaccharide synthesis and the poor alkylation of a disaccharide, this overall validated the more convergent strategy of including an O4 modification at the disaccharide level, and not by attempting its introduction into longer oligosaccharide sequences.

**Scheme 7:**
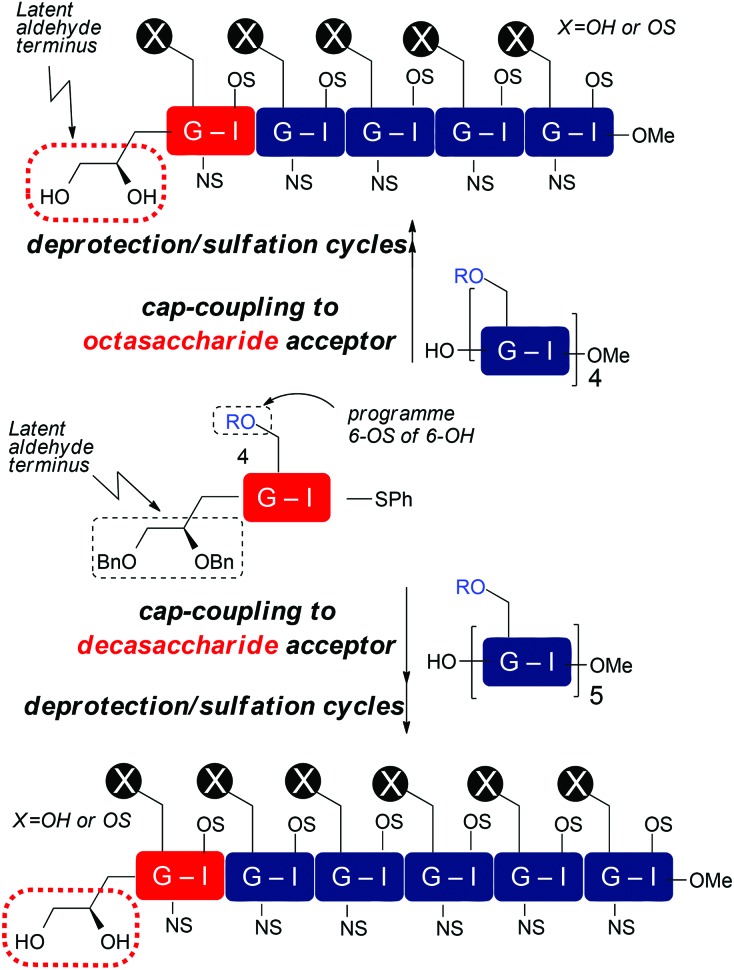
Strategy for end-capping to heparin and LMWHs bearing non-reducing terminal O4 diol tag, R = Bn or Bz.

With disaccharide building blocks **20** and **21** available we sought to demonstrate their application to synthesis of longer O4-tagged HS and LMWH-related oligosaccharides. To illustrate the capacity to access different lengths and backbone sulfation types, the strategy was as outlined in Scheme 7, capping acceptor octa- and decasaccharides with our differentially O6-derivatized O4-LAT-bearing “capper” disaccharides, which, after deprotection and sulfations, thereby aimed to provide O4-terminal diol tag bearing deca- and dodecasaccharides. This would establish this as a route to the longest currently available end-modified synthetic heparin- or LMWH-like oligosaccharides. It would also illustrate applicability to both different O6 sulfation systems, including LMWH. If successful, then release of an aldehyde terminus could be effected analogously to our O1-LAT release using NaIO_4_.

**Scheme 8:**
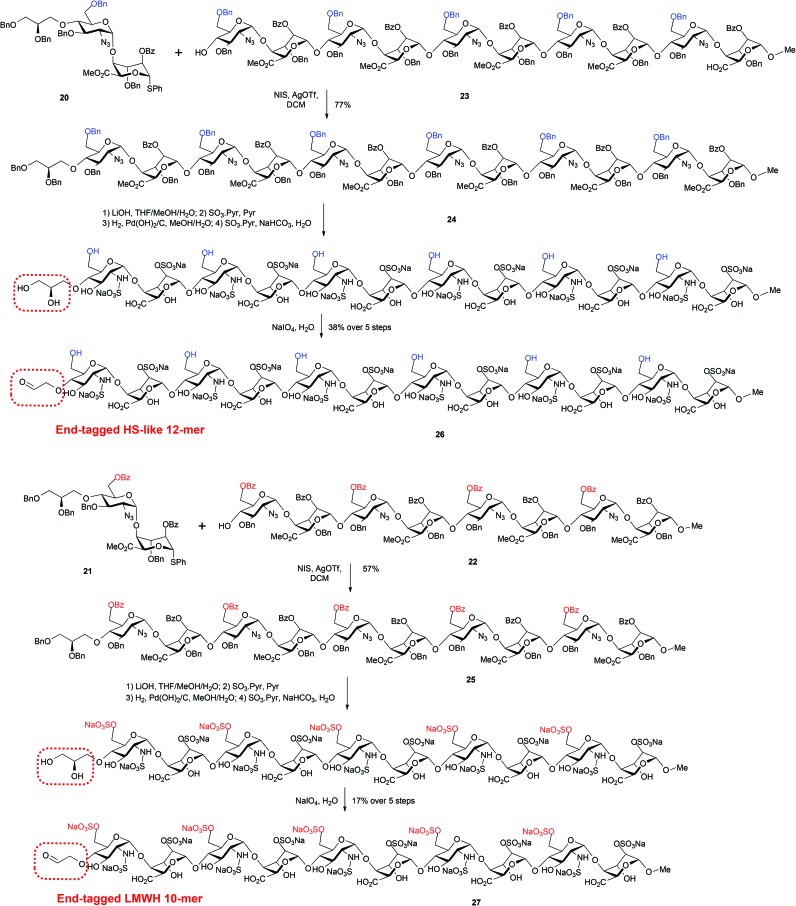
Synthesis of LMWH deca- and HS-like dodecasaccharides with non-reducing end latent aldehyde tethers and conversion to terminal aldehydes.

Accordingly, tagged glycosylation-capping of per-GlcN-6-OBz octasaccharide acceptor **22** ^4^ with O4-modified disaccharide **21** using NIS and AgOTf at 0 °C, provided the novel end-tagged decasaccharide **25** in good yield and high anomeric purity (Scheme 8). Further, the novel dodecasaccharide **24** was obtained in similarly good yield through tagged glycosylation-capping of decasaccharide acceptor **23** ^5a^ with O4-modified disaccharide **20**.

Finally, the fully protected oligosaccharides **24** and **25** were subjected to standard deprotection and *O*/*N*-sulfation steps to afford the native-type species **26** and **27** bearing non-reducing end reactive aldehyde groups (Scheme 8).

Analysis of dodecasaccharide **26** by 800 MHz NMR spectroscopy indicated formation of a hemiacetal between the free aldehyde and the 3-position of the terminal glucosamine unit. This was not unexpected given the potential ease of cyclisation to form a terminal 6-membered ring system. We are however confident that the material was homogenous in terms of the installed sulfation pattern and that the latent motif, whilst preferring to exist in solution predominantly as an acetal, would react as an aldehyde in subsequent conjugation/labelling studies. This was previously demonstrated on our reducing end analogue HS-like dodecasaccharide, which behaved smilarly.^11^

## Conclusions

A reliable and scalable synthesis of two non-reducing end modified GlcN-IdoA disaccharides containing a latent aldehyde group enables inclusion into LMWH- and HS-related deca- and dodecasaccharides. These are the first examples of such O4-modified heparin-like oligosaccharides. This provides a new approach to install an O4 tag onto defined HS oligosaccharide mimetics, which will enable applications in a variety of labelling and conjugation chemistries. The disaccharide units are generic tools that should find utility for final iteration stage coupling to diverse heparin-like oligosaccharides. These reagents also offer scope for exploitation in synthesis of other d-GlcN-containing oligosaccharides.

## Experimental section

### Phenyl 2-azido-3,6-di-*O*-benzyl-4-*O*-acetoxyethyl-2-deoxy-1-thio-α-d-glucopyranoside (**2**)

Glucosamine derivative **1** ^18^ (2.0 g, 4.2 mmol) was dissolved in dry THF (20 mL) under nitrogen. NaH (185 mg, 4.6 mmol, 60% in mineral oil) was added and the suspension stirred for 30 min at room temperature. Ethylbromoacetate (510 μL, 4.6 mmol) was added and the suspension heated at 55 °C overnight (16 h). TLC analysis (EtOAc/hexane, 3 : 1) showed starting material remained along with product at a slightly higher *R*_f_. Successive additions of NaH and ethylbromoacetate were made at 16 h and 24 h. After this time a new spot was seen to form on the baseline of the TLC plate and the reaction was stopped (quenching with AcOH). The solution was taken in EtOAc/H_2_O (20 mL each), the layers separated and the organics washed with saturated aqueous NaHCO_3_, saturated aqueous NaCl, dried (MgSO_4_) and solvent removed *in vacuo*. The crude material was purified by silica gel flash chromatography, eluting with EtOAc/hexane, 8 : 1, 5 : 1 to yield **2** (666 mg, 1.1 mmol, 29%) as a clear gum. *R*_f_ 0.59 (EtOAc/hexane 1 : 3); [*α*]_D_ +120.5 (*c* = 2.1, DCM); ^1^H NMR (400 MHz; CDCl_3_): *δ* 7.42 (ddt, *J* = 4.4, 2.8, 1.4 Hz, 2H, Ar*H*), 7.31–7.18 (m, 13H, Ar*H*), 5.52 (d, *J* = 5.2 Hz, 1H, H_1_), 4.85 (d, *J* = 10.5 Hz, 1H, C*H*_2_Ar), 4.77 (d, *J* = 10.5 Hz, 1H, C*H*_2_Ar), 4.54 (d, *J* = 11.9 Hz, 1H, C*H*_2_Ar), 4.37 (d, *J* = 11.9 Hz, 1H, C*H*_2_Ar), 4.36–4.34 (m, 1H, H_5_), 4.24 (d, *J* = 15.8 Hz, 1H, C*H*_2_C(O)OEt), 4.08–4.03 (m, 3H, C*H*_2_C(O)OEt, H_6B_, H_4_), 3.85–3.74 (m, 3H, OC*H*_2_CH_3_, H_2_), 3.61 (dd, *J* = 11.0, 1.9 Hz, 1H, H_6A_), 3.55 (dd, *J* = 9.9, 8.7 Hz, 1H, H_3_), 1.17 (t, *J* = 7.1 Hz, 3H, OCH_2_C*H*_3_); ^13^C NMR (100 MHz; CDCl_3_): *δ* 169.6, 137.9, 137.6, 133.4, 132.2, 129.1, 128.5, 128.4, 128.2, 128.0, 127.8, 127.7, 87.2, 81.7, 79.1, 75.7, 73.4, 71.4, 70.3, 68.3, 64.1, 61.0, 14.3; MS ES [M + Na]^+^ *m*/*z* 586.0; HRMS (TOF^+^) *m*/*z* calcd for C_30_H_33_N_3_O_6_SNa [M + Na]^+^ 586.1988, found 586.1995.

### Phenyl 2-azido-3,6-di-*O*-benzyl-4-*O*-acetoxy-2-deoxy-1-thio-α-d-glucopyranoside (**3**)

#### *Via* saponification from ester **2**

To a stirred solution of **2** (259 mg, 0.46 mmol) in THF (3 mL) was added sodium hydroxide solution (0.5 mL, 1 M solution) at 0 °C over 10 minutes. The mixture was then stirred at room temperature for 6 h after which time the reaction was shown to be complete by TLC analysis (EtOAc/Hex, 1 : 1). The reaction mixture was cooled back to 0 °C, acidified to pH 4 using hydrochloric acid (1 M), poured onto brine (25 mL) and extracted with ethyl acetate (3 × 40 mL). The organic layers were combined, washed with brine (25 mL), dried (MgSO_4_), filtered and concentrated *in vacuo*. The crude product was purified by silica gel flash chromatography eluting with hexane/ethyl acetate, 4 : 1 to give acid **3** (199 mg, 0.37 mmol, 81%) as a colourless oil. *R*_f_ = 0.21 (EtOAc/Hex, 1 : 2.5); [*α*]_D_ = +79.2 (*c* = 1.5, CHCl_3_); ^1^H NMR (400 MHz; CDCl_3_) *δ* 7.40–7.39 (m, 2H, Ar*H*), 7.30–7.16 (m, 13H, Ar*H*), 5.52 (d, *J* = 5.4 Hz, 1H, H_1_), 4.88 (d, *J* = 10.7 Hz, 1H, C*H*_2_Ar), 4.72 (d, *J* = 10.7 Hz, 1H, C*H*_2_Ar), 4.54 (d, *J* = 11.9 Hz, 1H, C*H*_2_Ar), 4.36 (d, *J* = 11.9 Hz, 1H, C*H*_2_Ar), 4.31–4.23 (m, 2H, H_5_, C*H*_2_CO_2_H), 4.08 (d, *J* = 16.7 Hz, 1H, C*H*_2_CO_2_H), 3.84 (dd, *J* = 10.2, 5.4 Hz, 1H, H_2_), 3.75–3.70 (m, 2H, H_4_, H_6B_), 3.57–3.51 (m, 2H, H_3_, H_6A_); ^13^C NMR (100 MHz, CDCl_3_) *δ* 137.2, 136.8, 132.9, 131.9, 129.0, 128.9, 128.5, 128.4, 128.2, 128.1, 127.7, 127.6, 127.5, 86.9, 80.7, 78.8, 73.3, 70.9, 69.9, 68.1, 63.9; MS NSI [M – H]^–^ *m*/*z* 534.2; HRMS (FTMS NSI^–^) *m*/*z* calcd for C_28_H_28_N_3_O_6_S_1_ [M – H]^–^ 534.1704, found 534.1710.

#### *Via* reaction of **1** with iodoacetic acid

To a solution of **1** (1.0 g, 2.10 mmol) in anhydrous DMF (10 mL) was added NaH (350 mg, 14.6 mmol, 60% in mineral oil) and KI (600 mg, 3.62 mmol) and the suspension stirred under nitrogen at 0 °C for 30 minutes. After this time iodoacetic acid (1.1 mL, 2.42 mmol) was added drop-wise and the reaction solution stirred at ambient temperature for a further 15 h. TLC analysis (EtOAc/Hex, 1 : 1) then indicated the reaction was incomplete and further NaH (70 mg, 2.92 mmol) and iodoacetic acid (0.2 mL, 0.48 mmol) were added and stirring continued for 6 h at 40 °C. Upon completion (as adjudged by TLC) the reaction solution was cooled to ambient temperature, diluted with distilled H_2_O (15 mL) and extracted with EtOAc (4 × 10 mL). The combined organics were then washed with H_2_O (5 × 10 mL), dried (MgSO_4_) and solvent removed *in vacuo*. The crude acid **3** was purified by silica gel flash chromatography eluting with hexane : EtOAc, 3 : 1, 1 : 1 to afford **3** (800 mg, 1.50 mmol, 71%) as a pale orange oil. Analytical data matched those above.

#### NBD-d-GlcN derivative (**6**)

NBD-amine salt **4** ^19^ (29 mg, 0.11 mmol), acid **3** (69 mg, 0.13 mmol), TBTU (41 mg, 0.13 mmol) and DIPEA (34 μL, 0.194 mmol) were dissolved in anhydrous DMF (4 mL) under nitrogen and stirred for 46 hours until the reaction was shown to have reached completion by TLC analysis (EtOAc/Hex, 1 : 2). The reaction was diluted with diethyl ether and washed with water three times. The organic phase was dried (MgSO_4_) and concentrated *in vacuo*. The crude material was purified by silica gel flash chromatography eluting with hexane/ethyl acetate, 3 : 1 to yield **6** as a yellow oil (40 mg, 0.05 mmol, 42%) *R*_f_ = 0.71 (hexane/EtOAc 3 : 1); [*α*]_D_ (*c* = 1.5, DCM) +24.9; ^1^H NMR (400 MHz; CDCl_3_) *δ* 8.31 (d, *J* = 8.6 Hz, 1H, NBD-Ar*H*), 7.79–7.76 (brs, 1H, N*H*), 7.60–7.58 (brs, 1H, N*H*), 7.42–7.38 (m, 2H, Ar*H*), 7.32–7.18 (m, 13H, Ar*H*), 5.87 (d, *J* = 8.6 Hz, 1H, NBD-Ar*H*), 5.54 (d, *J* = 5.5 Hz, 1H, H_1_), 5.01 (d, *J* = 10.6 Hz, 1H, C*H*_2_Ar), 4.61 (d, *J* = 10.6 Hz, 1H, C*H*_2_Ar), 4.48 (d, *J* = 11.7 Hz, 1H, C*H*_2_Ar), 4.40 (d, *J* = 11.7 Hz, 1H, C*H*_2_Ar), 4.23–4.19 (m, 2H, C*H*_2_C(O)NH, H_6B_), 4.12 (d, *J* = 16.3 Hz, 1H, C*H*_2_C(O)NH), 3.86 (dd, *J* = 10.2, 5.5 Hz, 1H, H_2_), 3.68–3.64 (m, 2H, H_3_, H_5_), 3.56–3.51 (m, 2H, H_4_, H_6A_), 3.10–3.07 (m, 3H, NHC*H*_2_C*H*_2_NH), 2.91–2.87 (m, 1H, HC*H*_2_C*H*_2_NH); ^13^C NMR (100 MHz, CDCl_3_) *δ* 172.4, 144.2, 143.9, 137.1, 136.8, 136.6, 132.9, 132.1, 129.2, 129.1, 128.9, 128.6, 128.5, 128.4, 128.3, 128.1, 128.0, 87.2, 81.1, 78.3, 76.1, 73.7, 71.4, 70.7, 68.4, 64.4, 37.5; MS ES [M + Na]^+^ *m*/*z* 763.0; HRMS (TOF^+^) *m*/*z* calcd for C_36_H_36_N_8_O_8_NaS^+^: 763.2270, found: 763.2293; IR (neat) *ν*_max_ 3280, 2922, 2104, 1663, 1579, 1294, 1260, 1103, 1047 cm^–1^; UV absorption *λ*_max_ 489 nm, *ε*_max_ 1.19 × 10^4^ L mol^–1^ cm^–1^ (CHCl_3_); fluorescence emission *λ*_max_ 716 nm.

#### Pyrene-d-GlcN derivative (**7**)

Carboxylic acid **3** (69 mg, 0.13 mmol), *N*-(6-aminohexyl)-4-(pyren-4-yl)butanamide hydrochloride **5** ^20^ (66 mg, 0.16 mmol), TBTU (41 mg, 0.13 mmol) and DIPEA (34 μL, 0.19 mmol) were dissolved in anhydrous DMF (4 mL) under nitrogen and the reaction stirred for 44 h whereupon it was shown to be complete by TLC analysis (EtOAc/Hex, 4 : 1). The reaction solution was diluted with diethyl ether (20 mL) and washed with water (7 × 20 mL). The diethyl ether was removed *in vacuo* giving the crude product as a pale yellow oil. This was purified by silica gel flash chromatography, eluting with EtOAc/hexane, 4 : 1 to yield **7** (45 mg, 0.05 mmol, 83%) as a colourless oil. *R*_f_ = 0.5 (EtOAc/Hex, 4 : 1); [*α*]_D_ +48.7 (*c* = 1.5, DCM); ^1^H NMR (400 MHz; CDCl_3_) *δ* 8.21 (d, *J* = 9.3 Hz, 1H, Ar*H*), 8.08 (d, *J* = 7.1 Hz, 2H, Ar*H*), 8.01 (d, *J* = 8.2 Hz, 2H, Ar*H*), 7.94 (s, 2H, Ar*H*), 7.90 (t, *J* = 7.6 Hz, 1H, Ar*H*), 7.77 (d, *J* = 7.8 Hz, 1H, Ar*H*), 7.41–7.40 (m, 3H, Ar*H*), 7.27–7.18 (m, 12H, Ar*H*), 6.73 (t, *J* = 5.8 Hz, 1H, NH), 5.51 (d, *J* = 5.4 Hz, 1H, H_1_), 5.42–5.40 (brs, 1H, NH), 4.84 (d, *J* = 10.5 Hz, 1H, C*H*_2_Ar), 4.61 (d, *J* = 10.5 Hz, 1H, C*H*_2_Ar), 4.46 (d, *J* = 11.9 Hz, 1H, C*H*_2_Ar), 4.36 (d, *J* = 11.9 Hz, 1H, C*H*_2_Ar), 4.22–4.19 (m, 1H, H_5_), 4.14 (d, *J* = 15.3 Hz, 1H, CH_2_C(O)NH), 3.95 (d, *J* = 15.3 Hz, 1H, C*H*_2_C(O)NH), 3.83 (dd, *J* = 10.2, 5.4 Hz, 1H, C*H*_2_Pyr), 3.72–3.48 (m, 6H, H_2_, H_3_, H_4_, H_6ab_, C*H*_2_Pyr × 1), 3.30 (t, *J* = 7.2 Hz, 2H, C*H*_2_C(O)), 3.13–3.02 (m, 3H, C*H*_2_NH), 2.88–2.79 (m, 1H, C*H*_2_NH), 2.18–2.12 (m, 2H, C*H*_2_ alkyl), 1.31–1.10 (m, 8H, C*H*_2_ alkyl);^13^C NMR (100 MHz, CDCl_3_) *δ* 172.7, 169.1, 137.3, 136.9, 135.9, 133.1, 132.1, 132.0, 131.4, 130.9, 129.9, 129.2, 128.7, 128.6, 128.4, 128.3, 128.0, 127.9, 127.8, 127.5, 127.4, 126.8, 125.9, 125.0, 124.9, 124.8, 123.4, 87.2, 81.0, 78.3, 75.9, 73.6, 71.5, 68.3, 64.2, 39.2, 38.4, 36.1, 32.8, 29.5, 27.5, 26.1; MS ES [M + Na]^+^ *m*/*z* 926.0; HRMS (TOF^+^) *m*/*z* calcd for C_54_H_58_N_5_O_6_S: 904.4130, Found: 904.4081; IR (neat) *ν*_max_ 3313, 3033, 2927, 2858, 2105, 1649, 1535, 1262, 1101, 1072, 1045, 1026 cm^–1^; UV absorption *λ*_max1_ 279 nm, *λ*_max2_ 329 nm, *ε*_max1_ 3.42 × 10^4^ L mol^–1^ cm^–1^, *ε*_max2_ 2.69 × 10^4^ L mol^–1^ cm^–1^ (CHCl_3_); fluorescence emission *λ*_max1_ 327 nm, *λ*_max2_ 207 nm.

### Phenyl-2-azido-2-deoxy-3,6-di-*O*-benzyl-4-*O*-[(*S*)-2,3-bis(benzyloxy)propoxy]-1-thio-β-d-glucopyranoside (**17**)

To **1** (2.67 g, 5.60 mmol) was added dry DMF (30 mL) under N_2_ and the solution cooled to 0 °C. NaH (60% in mineral oil) (248.0 mg, 6.2 mmol) was added in two portions over 30 min while being kept under N_2_. **14** ^23^ (2.49 g, 6.2 mmol) in dry DMF (20 mL) was then added dropwise and the suspension allowed to warm to RT and stirred for a further 3 h. TLC analysis (3/1, hexane/EtOAc) showed the reaction to be complete and quenching was effected with aqueous NaHCO_3_ (1 mL). The solution was partitioned between EtOAc and H_2_O. The layers were separated and the organic phase washed with 1 M HCl, H_2_O, brine, dried (MgSO_4_), filtered and evaporated. The crude product was purified by flash column chromatography (EtOAc/hexane gradient 1 : 9) yielding **17** (3.70 g, 5.1 mmol, 90%) as a clear oil. *R*_f_ 0.71 (EtOAc/hexane 1 : 3); [*α*]_D_ –19.1 (*c* = 3.0, DCM); ^1^H NMR (400 MHz; CDCl_3_) *δ* 7.53–7.50 (m, 2H, Ar*H*), 7.27–7.15 (m, 23H, Ar*H*), 4.76 (d, *J* = 10.5 Hz, 1H, C*H*_2_Ar), 4.73 (d, *J* = 10.6 Hz, 1H, C*H*_2_Ar), 4.55 (d, *J* = 12.0 Hz, 1H, C*H*_2_Ar), 4.51 (d, *J* = 12.0 Hz, 1H, C*H*_2_Ar), 4.45–4.38 (m, 4H, 2 × C*H*_2_Ar), 4.32 (d, *J* = 10.1 Hz, 1H, H_1_), 3.92–3.88 (m, 1H, H_6A_ or H_6B_), 3.72–3.57 (m, 4H, H_5_, H_6A_ or H_6B_, C*H*_2_CH[OBn]CH_2_OBn), 3.45–3.44 (m, 2H, CH_2_CH[OBn]C*H*_2_OBn), 3.38–3.32 (m, 3H, H_3_, H_4_, CH_2_C*H*[OBn]CH_2_OBn), 3.22 (dd, *J* = 10.0, 9.0 Hz, 1H, H_2_); ^13^C NMR (100 MHz; CDCl_3_) *δ* 138.5, 138.4, 138.2, 137.7, 133.7, 131.2, 129.1, 128.5, 128.4, 128.2, 128.0, 127.8, 127.7, 127.6, 127.5, 85.9, 84.9, 79.4, 78.2, 77.3, 75.8, 73.5, 73.4, 73.2, 72.4, 69.8, 68.8, 64.9; MS ES [M + NH_4_]^+^ *m*/*z* 750.0; HRMS (ES-TOF^+^) *m*/*z* calcd for C_43_H_49_N_4_O_6_S [M + NH_4_]^+^ 749.3368, found 749.3365.

### Phenyl-2-azido-2-deoxy-3-*O*-benzyl-4-*O*-[(*S*)-2,3-bis(benzyloxy)propoxy]-6-*O*-benzoyl-1-thio-β-d-glucopyranoside (**18**)

To **16** ^12^ (1.83 g, 3.72 mmol) was added dry THF (20 mL) under N_2_ and the solution cooled to 0 °C. NaH (60% in mineral oil) (164.0 mg, 4.10 mmol) was added in two portions over 30 min. while being kept under N_2_. **14** ^23^ (1.81 g, 4.46 mmol) in dry THF (10 mL) was then added dropwise and the suspension allowed to warm to RT and stirred for a further 2 h. TLC analysis (3/1, hexane/EtOAc) showed the reaction to be virtually complete (Note: allowing the reaction continue for longer resulted in no further consumption of starting material and the formation of another product; suggested to be transesterification) and quenching was effected with glacial AcOH (4.1 mmol) and solvents removed *in vacuo*. The solution was partitioned between EtOAc and H_2_O. The layers were separated and the organic phase washed with 1 M HCl, H_2_O, brine, dried (MgSO_4_), filtered and evaporated. The crude product was purified by flash column chromatography (EtOAc/hexane gradient 1 : 9, 1 : 7) yielding **18** (1.26 g, 1.60 mmol, 69%) as a clear oil along with recovered starting material **18** (979 mg). *R*_f_ 0.59 (EtOAc/hexane 1 : 3); [*α*]_D_ –19.7 (*c* = 3.8, DCM); ^1^H NMR (400 MHz; CDCl_3_) *δ* 7.97–7.94 (m, 2H, Ar*H*), 7.56–7.52 (m, 1H, Ar*H*), 7.45–7.38 (m, 4H, Ar*H*), 7.30–7.11 (m, 16H, Ar*H*), 7.02–6.98 (m, 2H, Ar*H*), 4.76 (d, *J* = 10.4 Hz, 1H, C*H*_2_Ar), 4.71 (d, *J* = 10.4 Hz, 1H, C*H*_2_Ar), 4.71 (dd, *J* = 12.0, 2.0 Hz, 1H, H_6A_ or H_6B_), 4.56 (d, *J* = 12.0 Hz, 1H, C*H*_2_Ar), 4.52 (d, *J* = 12.0 Hz, 1H, C*H*_2_Ar), 4.42 (d, *J* = 12.1 Hz, 1H, C*H*_2_Ar), 4.39 (d, *J* = 12.0 Hz, 1H, C*H*_2_Ar), 4.35–4.31 (m, 2H, H_1_, H_6A_ or H_6B_), 3.99–3.98 (m, 1H, CH_2_CH[OBn]C*H*_2_OBn), 3.64–3.58 (m, 2H, CH_2_CH[OBn]C*H*_2_OBn, CH_2_C*H*[OBn]CH_2_OBn), 3.54–3.50 (m, 1H, H_5_), 3.49–3.47 (m, 2H, C*H*_2_CH[OBn]C*H*_2_OBn), 3.40 (t, *J* = 9.6 Hz, 1H, H_3_), 3.34 (t, *J* = 9.6 Hz, 1H, H_4_), 3.20 (dd, *J* = 10.1, 9.6 Hz, 1H, H_2_); ^13^C NMR (100 MHz; CDCl_3_) *δ* 166.0, 138.4, 138.1, 137.5, 134.2, 133.4, 130.5, 130.0, 129.8, 129.0, 128.7, 128.6, 128.5, 128.4, 128.2, 127.8, 127.7, 85.6, 84.7, 78.4, 77.4, 77.2, 76.0, 73.5, 72.5, 69.5, 64.8, 63.1; MS ES [M + Na]^+^ *m*/*z* 768.0; HRMS (TOF^+^) *m*/*z* calcd for C_43_H_49_N_5_O_6_S [M + NH_4_]^+^ 763.3399, found 763.3398.

### Methyl (phenyl 4-*O*-(2-azido-3,6-di-*O*-benzyl-2-deoxy-4-*O*-[(*S*)-2,3-bis(benzyloxy)propoxy]-α-d-glucopyranosyl)-2-*O*-benzoyl-3-*O*-benzyl-1-thio-α-l-idopyranoside)-uronate (**20**)

To **17** (500 mg, 0.68 mmol) was added acetone (5.0 mL) and the mixture cooled to 0 °C in an icebath. *N*-Bromosuccinimide (242 mg, 1.36 mmol) was then added. After 3 h the reaction was quenched by addition of aqueous NaHCO_3_ (2.0 mL) and the solution stripped. The crude mixture was purified by flash column chromatography using EtOAc/hexane (1 : 4, 1 : 3) as the eluent to yield a mixture of hemi-acetals (333 mg, 0.52 mmol, 76%) as a white foam. The mixture of anomers was used immediately. *R*_f_ 0.14 (EtOAc/hexane 1 : 3); MS ES: [M + Na]^+^ *m*/*z* 663; HRMS (TOF-ES^+^): *m*/*z* calcd for C_37_H_41_N_3_O_7_Na [M + Na]^+^: 662.2837, found 662.2836. See ESI† for copies of ^1^H and COSY NMR spectra. To the mixture of hemi-acetals (1.8 g, 2.8 mmol) was added dry DCM (40 mL), CCl_3_CN (1.40 mL, 14.0 mmol) and sodium hydride 60% (11.0 mg, 0.3 mmol). The solution was stirred for 1 h. The solvent was evaporated and the crude residue purified by flash column chromatography using EtOAc/hexane (1 : 4 + 1% NEt_3_) as eluent. This yielded the desired imidate (1.70 g, 2.1 mmol, 95%) as a white foam and as a 5/1 (*α*/*β*) mixture, as determined by ^1^H NMR. This compound was used immediately. *R*_f_ 0.50 (EtOAc/hexane 1 : 3); HRMS (FTMS-NSI^+^): *m*/*z* calcd for C_39_H_45_N_5_O_7_ [M + NH_4_]^+^ 800.2379, found 800.2377. See ESI† for copy of ^1^H NMR spectrum. To iduronic ester acceptor **19** ^12^ (833 mg, 1.78 mmol) and the above imidate (1.70 g, 2.14 mmol) was twice added dry toluene and the solvent evaporated. The residue was dried under high vacuum for 2 h and, while kept under nitrogen, dry DCM (25 mL) was added. The solution was cooled to –30 °C using a 65 : 35 mixture of ^i^PrOH/H_2_O and a dry ice bath and TMSOTf (16 μL, 0.09 mmol) was then added. After 2 h the reaction was quenched with two drops of NEt_3_, solvents were removed *in vacuo* and flash column chromatography (EtOAc/hexane, 1 : 5, 1 : 2) as the eluent yielded **20** (1.10 g, 0.99 mmol, 55%) as a white foam, along with recovered acceptor **19** (300 mg); *R*_f_ 0.31 (EtOAc/hexane 1 : 3); [*α*]_D_ (*c* = 1.0, DCM) +327.1; ^1^H NMR (400 MHz; CDCl_3_) *δ* 8.07–8.04 (m, 2H, Ar*H*), 7.49–7.44 (m, 2H, Ar*H*), 7.43–7.38 (m, 2H, Ar*H*), 7.35–7.29 (m, 4H, Ar*H*), 7.27–7.09 (m, 23H, Ar*H*), 7.04–7.00 (m, 2H, Ar*H*), 5.72 (s, 1H, H_5_), 5.35 (s, 1H, H_4_), 5.29 (d, *J* = 2.0 Hz, 1H, H_1_), 4.91 (d, *J* = 11.7 Hz, 1H, C*H*_2_Ph), 4.69 (d, *J* = 11.7 Hz, 1H, C*H*_2_Ph), 4.61 (d, *J* = 3.6 Hz, 1H, H_1′_), 4.55–4.48 (m, 2H, C*H*_2_Ph), 4.43 (s, 2H, C*H*_2_Ph), 4.38–4.31 (m, 2H, C*H*_2_Ph), 4.18 (d, *J* = 10.6 Hz, 1H, C*H*_2_Ph), 4.12 (t, *J* = 2.8 Hz, 1H, H_3_), 3.97 (s, 1H, H_2_), 3.81 (dd, *J* = 9.7, 3.3 Hz, 1H, H6_A′_), 3.71–3.63 (m, 3H, C*H*_2_Ph, C*H*_2_CH[OBn]CH_2_OBn), 3.61 (s, 3H, OC*H*_3_), 3.59–3.37 (m, 7H, H_5′_, H_6B′_, H_4′_, H_3′_, CH_2_CH[OBn]C*H*_2_OBn, CH_2_C*H*[OBn]CH_2_OBn,), 3.14 (dd, *J* = 10.0, 3.7 Hz, 1H, H_2′_); ^13^C NMR (100 MHz; CDCl_3_) *δ* 169.3, 165.6, 138.6, 138.2, 137.9, 137.8, 137.1, 135.4, 133.4, 131.5, 130.0, 129.5, 129.1, 128.8, 128.5, 128.4, 128.3, 128.2, 128.1, 127.9, 127.8, 127.6, 127.6, 127.5, 100.4, 87.0, 80.0, 78.0, 77.6, 77.4, 74.6, 73.4, 73.3, 73.1, 72.8, 72.4, 72.2, 71.6, 70.1, 69.2, 68.4, 67.6, 63.6, 52.3; MS ES [M + NH_4_]^+^ *m*/*z* 1133.5; HRMS (FTMS-NSI^+^) *m*/*z* calcd for C_64_H_69_N_4_O_13_S [M + NH_4_]^+^ 1133.4576, found 1133.4578.

### Methyl (phenyl 4-*O*-(2-azido-3-*O*-benzyl-6-*O*-benzoyl-2-deoxy-4-*O*-[(*S*)-2,3-bis(benzyloxy)propoxy]-α-d-glucopyranosyl)-2-*O*-benzoyl-3-*O*-benzyl-1-thio-α-l-idopyranoside)-uronate (**21**)

To **18** (1.26 g, 1.60 mmol) was added acetone (15.0 mL) and the mixture cooled to 0 °C in an icebath. *N*-Bromosuccinimide (569 mg, 3.20 mmol) was then added. After 2 h the reaction was quenched by addition of aqueous NaHCO_3_ (2.0 mL) and the solution stripped. The crude mixture was purified by flash column chromatography using EtOAc/hexane (1 : 4) as the eluent to yield the mixture of hemi-acetals (963 mg, 1.40 mmol, 87%) as a pale yellow oil. This mixture of anomers was used immediately. *R*_f_ 0.24 (EtOAc/hexane 1 : 3); MS ES: [M + Na]^+^ *m*/*z* 676; HRMS (TOF-ES^+^): *m*/*z* calcd for C_37_H_39_N_3_O_8_Na [M + Na]^+^: 676.2630, found 676.2634. See ESI† for copies of ^1^H and COSY NMR spectra. To this mixture of hemi-acetals (963 mg, 1.40 mmol) was added dry DCM (10 mL), CCl_3_CN (421 μL, 4.20 mmol) and sodium hydride 60% (6.0 mg, 0.14 mmol). The solution was stirred for 2 h. The solvent was evaporated and the crude residue purified by flash column chromatography using EtOAc/hexane (1 : 5 + 1% NEt_3_) as eluent. This yielded the desired imidate (918 mg, 1.09 mmol, 77%) as a pale yellow foam and a 95/5 *α*/*β* mixture, as determined by ^1^H NMR. This compound was used immediately. *R*_f_ 0.57 (EtOAc/hexane 1 : 3). See ESI† for copy of ^1^H NMR spectrum. To iduronic ester acceptor **19** ^12^ (441 mg, 0.89 mmol) and the above imidate (900 mg, 1.07 mmol) was twice added dry toluene and the solvent evaporated. The residue was dried under high vacuum for 2 h and, while kept under nitrogen, dry DCM (15 mL) was added. The solution was cooled to –30 °C using a 65 : 35 mixture of ^i^PrOH/H_2_O and a dry ice bath and TMSOTf (8 μL, 0.04 mmol) was then added. After 0.5 h the reaction was quenched with two drops of NEt_3_, solvents were removed *in vacuo* and flash column chromatography (EtOAc/hexane, 1 : 7, 1 : 6, 1 : 5, 1 : 3) as the eluent yielded **21** (472 mg, 0.42 mmol, 47%) as a white foam, along with recovered acceptor **19** (123 mg). *R*_f_ 0.44 (EtOAc/hexane 1 : 3); [*α*]_D_ (*c* = 0.75, DCM) +287.1; ^1^H NMR (400 MHz; CDCl_3_) *δ* 8.18 (dd, *J* = 8.4, 1.3 Hz, 2H, Ar*H*), 8.02 (dd, *J* = 8.4, 1.3 Hz, 2H, Ar*H*), 7.60–7.58 (m, 3H, Ar*H*), 7.52–7.50 (m, 2H, Ar*H*), 7.46–7.40 (m, 6H, Ar*H*), 7.37–7.24 (m, 16H, Ar*H*), 7.22–7.20 (m, 2H, Ar*H*), 7.15 (td, *J* = 3.7, 1.7 Hz, 2H, Ar*H*), 5.84 (brs, 1H, H_1_), 5.46 (brs, 1H, H_2_), 5.43 (d, *J* = 2.0 Hz, 1H, H_5_), 5.02 (d, *J* = 11.7 Hz, 1H, C*H*_2_Ph), 4.81–4.77 (m, 2H, C*H*_2_Ph, H_6A_), 4.72 (d, *J* = 3.6 Hz, 1H, H_1′_), 4.66–4.59 (m, 2H, C*H*_2_Ph), 4.57–4.49 (m, 2H, C*H*_2_Ph), 4.43 (dd, *J* = 12.4, 2.7 Hz, 1H, H_6B_), 4.25–4.22 (m, 2H, H_3_, C*H*_2_Ph), 4.11 (brs, 1H, H_4_), 4.06–4.00 (m, 2H, H_5′_, CH_2_C*H*[OBn]CH_2_OBn), 3.84–3.81 (m, 4H, OC*H*_3_, C*H*_2_Ph), 3.73–3.67 (m, 2H, C*H*_2_CH[OBn]CH_2_OBn), 3.62–3.60 (m, 2H, CH_2_CH[OBn]C*H*_2_OBn), 3.55–3.44 (m, 2H, H_3′_, H_4′_), 3.23 (dd, *J* = 9.9, 3.6 Hz, 1H, H_2′_); ^13^C NMR (100 MHz; CDCl_3_) *δ* 167.0, 163.7, 163.3, 136.2, 135.9, 135.4, 134.9, 133.2, 131.2, 130.9, 129.2, 127.8, 127.7, 127.4, 126.9, 126.8, 126.6, 126.3, 126.2, 126.1, 126.0, 125.9, 125.7, 125.5, 125.4, 123.1, 97.9, 84.8, 77.7, 76.5, 72.5, 71.2, 71.1, 70.5, 70.2, 69.8, 68.1, 67.5, 67.1, 66.1, 61.6, 50.3; MS ES [M + Na]^+^ *m*/*z* 1153; HRMS (FTMS-NSI^+^) *m*/*z* calcd for C_64_H_67_N_4_O_14_S_1_ [M + NH_4_]^+^ 1147.4369, found 1147.4357.

#### Dodecasaccharide (**24**)

Decasaccharide acceptor **22** ^5^ (391.0 mg, 0.10 mmol) and disaccharide donor **20** (138.0 mg, 0.12 mmol) were combined in dry toluene (10 mL). Solvent was removed *in vacuo* and the residue dried under high vacuum for 1 h. The resulting foam was then dissolved in dry DCM (5.0 mL) and cooled to 0 °C. 4 Å molecular sieves (100 mg) were added followed by NIS (29.0 mg, 0.13 mmol) and AgOTf (2.6 mg, 10.0 μmol. The resultant suspension was stirred at this temperature for 0.5 h whereupon a deep red colour persisted. The reaction was quenched with NaHCO_3_ (25 mg) and Na_2_S_2_O_3_ (25 mg) in H_2_O (1.0 mL) and the mixture filtered through a Celite™ plug, washing with DCM. The layers were separated and the organics dried (MgSO_4_) and solvent removed *in vacuo* to reveal crude **24** as a yellow gum. The material was then purified by silica gel flash chromatography eluting with toluene/acetone, 20/1 to separate the product and unreacted decasaccharide acceptor **22** (131 mg recovered). The product **24** (367 mg, 76.0 μmol, 77%) was isolated as a white foam. *R*_f_ 0.38 (toluene/acetone 10 : 1); [*α*]_D_ (*c* = 1.1, DCM) +42.9; ^1^H NMR (400 MHz; CDCl_3_) *δ* 8.13–8.10 (m, 3H, Ar*H*), 8.01–7.92 (m, 11H, Ar*H*), 7.54–7.07 (m, 116H, Ar*H*), 5.57–5.56 (m, 5H, H_1IdoA_), 5.21–5.18 (m, 5H, H_2IdoA_), 5.10 (s, 1H, H_1IdoA_), 5.06 (s, 1H, H_2IdoA_), 4.97–4.90 (m, 6H, H_1GlcN_), 4.82–4.38 (m, 43H), 4.24–3.25 (m, 78H, incl. C(O)OC*H*_3_ × 6, OCH_3_); ^13^C NMR (100 MHz; CDCl_3_) *δ* 169.6, 169.4, 169.3, 167.7, 165.6, 165.2, 165.1, 138.6, 138.1, 138.0, 137.9, 137.8, 137.7, 137.5, 137.4, 133.6, 133.5, 132.3, 131.0, 130.0, 129.9, 129.8, 129.6, 129.4, 129.3, 129.2, 129.1, 129.0, 128.9, 128.8, 128.7, 128.6, 128.4, 128.3, 128.2, 128.1, 128.0, 127.9, 127.8, 127.7, 127.6, 127.5, 127.4, 127.3, 125.4, 100.3, 99.5, 99.3, 99.2, 98.2, 98.1, 98.0, 79.6, 78.3, 78.2, 78.1, 77.5, 77.4, 77.2, 76.9, 76.0, 75.6, 75.5, 75.4, 74.9, 74.4, 74.2, 74.0, 73.7, 73.6, 73.5, 73.4, 73.2, 72.5, 72.4, 71.6, 71.4, 70.7, 70.1, 68.0, 67.7, 67.4, 67.3, 67.2, 63.4, 63.3, 63.2, 63.1, 63.0, 61.7, 56.3, 52.1, 51.9, 51.8, 51.7, 51.6; (MS MALDI) *m*/*z* calcd for C_264_H_268_N_18_O_69_Na [M + Na]^+^ 4819.8, found 4819.8.

#### Decasaccharide (**25**)

Octasaccharide acceptor **23** ^4^ (161.0 mg, 52.0 μmol) and disaccharide donor **21** (71 mg, 62.0 μmol) were combined in dry toluene (10 mL). Solvent was removed *in vacuo* and the residue dried under high vacuum for 1 h. The resulting foam was then dissolved in dry DCM (5.0 mL) and cooled to 0 °C. 4 Å molecular sieves (100 mg) were added followed by NIS (15.2 mg, 68.0 μmol) and AgOTf (1.3 mg, 5.2 μmol. The resultant suspension was stirred at this temperature for 0.5 h whereupon a deep red colour persisted. The reaction was quenched with NaHCO_3_ (25 mg) and Na_2_S_2_O_3_ (25 mg) in H_2_O (1.0 mL) and the mixture filtered through a Celite™ plug, washing with DCM. The layers were separated and the organics dried (MgSO_4_) and solvent removed *in vacuo* to reveal crude **25** as a yellow gum. The material was then purified by silica gel flash chromatography eluting with toluene/acetone, 30/, 25/1, 20/1 to separate the product and unreacted octasaccharide acceptor **23** (52 mg recovered). The product was then purified again by silica gel flash chromatography eluting with hexane/EtOAc, 2/1, 1/1 to give **25** (82 mg, 20.0 μmol, 57%) as a white foam. *R*_f_ 0.29 (Ether/DCM 5 : 95); [*α*]_D_ (*c* = 0.5, DCM) +63.4; ^1^H NMR (400 MHz; CDCl_3_) *δ* 8.01–7.95 (m, 24H, Ar*H*), 7.17–7.11 (m, 86H, Ar*H*), 5.50–5.48 (m, 4H, H_1IdoA_), 5.13–5.08 (m, 4H, H_2IdoA_), 4.99 (brs, 1H, H_1IdoA_), 4.97 (brs, 1H, H_2IdoA_), 4.83–4.16 (m, 43H, C*H*_2_Ar × 23, H_5IdoA_, H_1GlcN_, H_6ABGlcN_), 4.06–4.70 (m, 21H, C*H*_2_Ar × 4, H_3IdoA_, H_4IdoA_, H_5GlcN_, H_4GlcN_ × 4, CH_2_C*H*[OBn]CH_2_OBn), 3.65–3.56 (m, 2H, C*H*_2_CH[OBn]CH_2_OBn), 3.53 (s, 3H, OC*H*_3_), 3.47–3.42 (m, 14H, H_3GlcN_, H_4GlcN_ × 1, CH_2_CH[OBn]C*H*_2_OBn C(O)OC*H*_3_ × 2), 3.25–3.04 (m, 14H, H_2GlcN_, C(O)OC*H*_3_ × 3); ^13^C NMR (100 MHz; CDCl_3_) *δ* 169.6, 169.3, 169.2, 166.0, 165.9, 165.6, 165.2, 165.1, 138.3, 138.0, 137.7, 137.5, 137.4, 137.3, 137.2, 133.7, 133.5, 133.1, 133.0, 132.5, 129.9, 129.6, 128.8, 128.7, 128.5, 128.4, 128.3, 128.2128.1, 128.0, 127.8, 127.6, 100.3, 99.1, 98.7, 98.4, 98.3, 98.3, 98.2, 98.1, 98.1, 98.0 anomeric carbons], 79.7, 78.6, 78.4, 78.3, 78.2, 77.8, 77.7, 77.6, 77.5, 77.3, 77.2, 77.0, 76.9, 76.7, 76.3, 75.9, 75.8, 75.7, 75.6, 75.4, 75.1, 75.0, 74.6, 74.5, 74.4, 74.3, 74.2, 73.9, 73.5, 72.4, 72.3, 71.7, 71.2, 70.2, 70.0, 69.9, 69.8, 69.6, 68.0, 67.1, 63.4 63.3, 63.2, 63.0, 62.3, 61.9, 61.8, 56.2, 52.3, 51.9, 51.7, 51.6, 51.6; (FTMS NSI^+^) *m*/*z* calcd for C_223_H_225_N_17_O_63_ [M+2NH_4_]^2+^ 2074.2457, found 2074.2422;

### General method for saponification of oligosaccharides **24** and **25**

Decasaccharide **25** (71.4 mg, 17.4 μmol) was dissolved in THF (1 mL) and MeOH (0.5 mL). The solution was cooled to 0 °C and LiOH (22.0 mg, 522.5 μmol) in H_2_O (0.5 mL) added dropwise, whereupon the solution turned a faint yellow colour. Stirring was continued for 5 h at this temperature, at room temperature overnight and quenched with 1 M HCl (520 μL). Solvents were removed *in vacuo* and the crude white solid purified by silica gel flash chromatography, eluting with DCM/MeOH, 95/5, 90/10 to give the decasaccharide acid (31.0 mg, 10.0 μmol, 60%) as a white solid. *R*_f_ 0.41 (MeOH/DCM 1 : 9); ^1^H NMR (400 MHz; Acetic acid, d_4_) *δ* 7.36–6.97 (m, 60H, Ar*H*), 5.22–5.15 (m, 4H, H_1IdoA_), 5.03 (s, 3H, H_1GlcN_), 4.99–4.98 (m, 2H, H_1GlcN_), 4.87 (s, 1H, H_1IdoA_), 4.81–4.80 (m, 4H), 4.73 (s, 1H), 4.69–4.60 (m, 8H), 4.56–4.51 (m, 8H), 4.47–4.39 (m, 4H), 4.36 (d, *J* = 2.9 Hz, 2H), 4.33–4.25 (m, 4H), 4.13 (s, 1H), 4.04–4.01 (m, 5H), 3.96–3.77 (m, 21H), 3.69–3.40 (m, 30H), 3.33 (brs, 4H, includes OC*H*_3_); HRMS (FTMS NSI^–^) *m*/*z* calcd for C_148_H_165_N_15_O_53_ [M – 2H]^2–^ 1500.0344, found 1500.0334.

Reaction of compound **24** (350 mg, 73.0 μmol) following the above procedure yielded the product dodecasaccharide acid (235 mg, 57.0 μmol, 79%). *R*_f_ 0.49 (MeOH/DCM 1 : 9); ^1^H NMR (400 MHz; MeOD) *δ* 7.56–7.16 (m, 100H, ArH), 5.32–5.30 (brs, 6H, H_1IdoA_), 5.14–5.12 (brs, 6H, H_1GlcN_), 4.78–3.44 (m, 60H, H_2GlcN_, H_3GlcN_, H_4GlcN_, H_5GlcN_, H_6abGlcN_, H_2IdoA_, H_3IdoA_, H_4IdoA_, H_5IdoA_); HRMS (FTMS NSI^–^) *m*/*z* calcd for C_216_H_229_N_18_O_63_ [M – 3H]^3–^ 1360.8428, found 1360.8405.

### General method for *O*-sulfation and hydrogenation of oligosaccharides

The above decasaccharide acid (31.0 mg, 10.0 μmol) was dissolved in dry pyridine (1 mL) under N_2_ at RT. SO_3_·Py complex (66 mg, 414 μmol) was added and the suspension stirred 50 °C for 5 h then at RT overnight. A white precipitate formed from the pale yellow solution after 2 h. TLC analysis (EtOAc/Pyr/H_2_O/AcOH, 6/5/3/1) showed no starting material remained and one product spot had formed at lower *R*_f_. The reaction was quenched by addition of NaHCO_3_ (102 mg) in H_2_O (0.5 mL) and the solvents removed *in vacuo* (co-evaporationg with toluene). The crude white paste was then purified by silica gel flash chromatography, eluting with DCM/MeOH, 9/1, 4/1. The residue obtained was dissolved in MeOH/DCM (2 mL, v/v), stirred with Amberlite IR120^+^-Na^+^ resin for 12 h, filtered and solvent removed *in vacuo* to give target *O*-sulfated species (24.0 mg, 5.8 μmol, 56%) as a white solid which was used immediately in the next step. *R*_f_ 0.37 (MeOH/DCM/NH_4_OH 6 : 14 : 2). This material (11.1 mg, 2.7 μmol) was dissolved in MeOH (0.5 mL) and THF (0.5 mL) at RT. NaHCO_3_ (5 mg, 54 μmol) in H_2_O (0.5 mL) was added along with Pd(OH)_2_/C (10 mg) and the system purged with H_2_. Stirring was continued at 50 °C for 4 d. The suspension was then filtered through Celite® and solvents removed *in vacuo.* The residue was dissolved in water and desalted by passage through a Sephadex G-20 resin using water as eluent to reveal the free sugar (4.6 mg, 1.6 μmol, 59%) as a clear glass. ^1^H NMR (400 MHz; D_2_O) *δ* 5.07.5.05 (m, 3H, H_1IdoA_), 5.98–4.92 (m, 4H, H_1GlcN_), 4.88 (s, 1H, H_1IdoA_), 4.81–4.78 (m, 4H, H_5IdoA_), 4.36 (s, 1H, H_5IdoA_), 4.22–4.05 (m, 20H, H_2IdoA_, H_3IdoA_, H_6GlcN_), 3.99–3.70 (m, 13H, H_4IdoA_, H_5GlcN_, 3H from NRE tether), 3.65–3.42 (m, 11H, H_3GlcN_, H_4GlcN_, 2H from NRE tether), 3.28 (s, 3H, OC*H*_3_), 3.27–3.19 (m, H_4GlcN_), 2.69–2.59 (m, 5H, H_2GlcN_); HRMS (FTMS NSI^–^) *m*/*z* calcd for C_64_H_99_N_5_O_83_S_10_ [M – 6H]^6–^ 430.8487, found 430.8491, calcd for C_64_H_100_N_5_O_83_S_10_ [M – 5H]^5–^ 517.2198, found 517.2206.

Reaction of the above dodecasaccharide acid (170.0 mg, 42.0 μmol) following the above *O*-sulfation procedure yielded the target *O*-sulfated species (120.0 mg, 25.0 μmol, 60%) which was used immediately in the next step. *R*_f_ 0.69 (MeOH/DCM, 1 : 5); FTMS (NSI^–^) *m*/*z* calcd for C_216_H_226_N_18_O_81_S_6_ [M – 6H]^6–^ 760.8746, found 760.8747. Reaction of this compound (65.0 mg, 13.5 μmol) following the above hydrogenation procedure yielded the free sugar (30.0 mg, 13.4 μmol, quant.). ^1^H NMR (400 MHz; D_2_O) *δ* 5.05–5.03 (brs, 5H, H_1IdoA_), 4.91–4.88 (m, 7H, H_1GlcN_ × 6, H_1IdoA_), 4.78 (brs, 5H, H_5IdoA_), 4.36 (s, 1H, H_5IdoA_), 4.20 (brs, 5H, H_2IdoA_), 4.11–4.02 (m, 7H, H_3IdoA_ × 6, H_2IdoA_), 3.95–3.87 (m, 6H, H_4IdoA_), 3.76–3.68 (m, 19H, H_5GlcN_ H_6ABGlcN_, 1H NRE tether), 3.56–3.45 (15H, H_3GlcN_ H_4GlcN_ × 5, 4H NRE tether), 3.29 (s, 3H, OMe), 3.23–3.17 (m, 1H, H_4GlcN_), 2.59–2.55 (m, 6H, H_2GlcN_); HRMS (FTMS-NSI^–^) *m*/*z* calcd for C_76_H_118_N_6_O_81_S_6_ [M – 6H]^6–^ 433.7276, found 433.7262, calcd for C_76_H_119_N_6_O_81_S_6_ [M – 5H]^5–^ 520.6746, found 520.6732.

### General method for *N*-sulfation of oligosaccharides

The decasaccharide free sugar (4.0 mg, 1.4 μmol) was dissolved in H_2_O (0.5 mL) at RT. NaHCO_3_ (10.1 mg, 120 μmol) and SO_3_·pyr complex (9.5 mg, 60.0 μmol) were then added and the suspension stirred at room temperature. Further additions of both reagents were made at 1 h, 3 h, 8 h, 20 h and 24 h. After 28 h solvent was removed *in vacuo* to reveal a crude white solid which was purified by passage through a Sephadex G-20 resin using water as eluent. The target material (4.6 mg, 1.4 μmol, 90%) was isolated as a clear glass. This material was used immediately in the periodate cleavage step.

Reaction of the dodecasaccharide free sugar (30.0 mg, 10.8 μmol) following the above procedure yielded the product (35.0 mg, 9.1 μmol, 92%). ^1^H NMR (400 MHz; D_2_O) *δ* 5.21–5.14 (m, 11H, H_1IdoA_, H_1GlcN_), 4.90 (brs, 1H, H_1IdoA_), 4.75–4.73 (m, 5H, H_5IdoA_), 4.32 (s, 1H, H_5IdoA_), 4.21–4.19 (m, 6H, H_2IdoA_), 4.11–4.09 (m, 6H, H_3IdoA_), 3.95–3.45 (m, 41H, H_4IdoA_, H_5GlcN_ H_6ABGlcN_, H_3GlcN_ H_4GlcN_, NRE tether, 5H), 3.29 (s, 3H, OMe), 3.13–3.09 (m, 6H, H_2GlcN_); HRMS (FTMS-NSI^–^) *m*/*z* calcd for C_76_H_118_N_6_O_99_S_12_ [M – 6H]^6–^ 513.6844, found 513.6840, C_76_H_117_N_6_O_99_S_12_ [M – 7H]^6–^ 440.1570, found 440.1567.

### General method for periodate cleavage of oligosaccharide NRE tether **26** and **27**

The *N*-sulfated decasaccharide (4.6 mg, 1.4 μmol) was dissolved in H_2_O (200 μL) at room temperature. NaIO_4_ (0.4 mg, 1.5 μmol) was added and the solution shaken in a 2.0 mL Eppendorf tube for 24 h. The reaction solution was diluted up to 1.0 mL with H_2_O and passed through a Sephadex G-20 resin using water as eluent. The target material **27** (4.3 mg, 1.3 μmol, 95%) was isolated as a white glass after freeze-drying. ^1^H NMR (600 MHz; D_2_O) *δ* 5.27–2.24 (m, 5H, H_1GlcN_), 5.08 (brs, 4H, H_1IdoA_), 4.88 (s, 1H, H_1IdoA_), 4.78 (brs, 4H, H_5IdoA_), 4.39 (brs, 1H, H_5IdoA_), 4.21–4.00 (m, 23H), 3.89–3.77 (m, 11H), 3.67–3.65 (m, 6H), 3.26 (s, 3H, OC*H*_3_), 3.24–3.21 (m, 5H, H_2GlcN_); HRMS (FTMS NSI^–^) *m*/*z* calcd for C_63_H_95_N_5_O_82_S_10_ [M – 6H]^6–^ 425.5110, found 425.5115.

Reaction of the *N*-sulfated dodecasaccharide (30.0 mg, 8.6 μmol) following the above procedure yielded the product **26** (26.3 mg, 7.6 μmol, 88%). ^1^H NMR (800 MHz; D_2_O) *δ* showed a complex mixture of cyclic acetal forms. See ESI† for copies of 800 MHz 1D and 2D NMR spectra; HRMS (FTMS-NSI^–^) *m*/*z* calcd for C_75_H_112_N_6_O_98_S_12_ [M – 8H]^8–^ 381.0066, found 381.0082.

## References

[cit1] Sasisekharan R., Shriver Z., Venkataraman G., Narayanasami U. (2002). Nat. Rev. Cancer.

[cit2] Xu Y., Masuko S., Takieddin M., Xu H., Liu R., Jing J., Mousa S. A., Linhardt R. J., Liu J. (2011). Science.

[cit3] Schwörer R., Zubkova O., Turnbull J. E., Tyler P. C. (2013). Chem. – Eur. J..

[cit4] Miller G. J., Hansen S. U., Avizienyte E., Rushton G., Cole C., Jayson G. C., Gardiner J. M. (2013). Chem. Sci..

[cit5] Hansen S. U., Miller G. J., Jayson G. C., Gardiner J. M. (2013). Org. Lett..

[cit6] Maza S., Mar Kayser M., Macchione G., López-Prados J., Angulo J., de Paz J. L., Nieto P. M. (2013). Org. Biomol. Chem..

[cit7] Ulrich S., Boturyn D., Marra A., Renaudet O., Dumy P. (2013). Chem. – Eur. J..

[cit8] Guerry A., Bernard J., Samain E., Fleury E., Cottaz S., Halila S. (2013). Bioconjugate Chem..

[cit9] de Paz J. L., Noti C., Seeberger P. H. (2006). J. Am. Chem. Soc..

[cit10] Shyam M., Rele S. M., Suri S., Iyer S. S., Subramanian Baskaran S., Elliot L., Chaikof E. L. (2004). J. Org. Chem..

[cit11] Hansen S. U., Miller G. J., Cole C., Rushton G., Avizienyte E., Jayson G. C., Gardiner J. M. (2013). Nat. Commun..

[cit12] Hansen S. U., Miller G. J., Baráth M., Broberg K. R., Avizienyte E., Helliwell M., Raftery J., Jayson G. C., Gardiner J. M. (2012). J. Org. Chem..

[cit13] Allen K. A., Brown R. L., Norris G., Tyler P. C., Watt D. K., Zubkova O. V. (2010). Carbohydr. Res..

[cit14] Hagihara S., Miyazaki A., Matsuo I., Tatami A., Suzuki T., Ito Y. (2007). Glycobiology.

[cit15] Johnson C. L., Guo Z. (2013). J. Carbohydr. Chem..

[cit16] Lindberg J., Strålfors P., Konradsson P. (2002). Tetrahedron.

[cit17] Koshida S., Suda Y., Fukui Y., Ormsby J., Sobel M., Kusumoto S. (1999). Tetrahedron Lett..

[cit18] Garegg P. J., Kvarnström I., Niklasson A., Niklasson G., Svensson S. C. T. (1993). J. Carbohydr. Chem..

[cit19] Cotte A., Bader B., Kuhlmann J., Waldmann H. (1999). Chem. – Eur. J..

[cit20] Etika K. C., Jochum F. D., Cox M. A., Schattling P., Theato P., Grunlan J. C. (2010). Macromolecules.

[cit21] Recent effective methodology for aldehyde conjugation using various labelling reagents has been described, see: Ulrich S. Boturyn D. Marra A. Renaudet O. Dumy P., Chem. – Eur. J., 2013, 20 , 34 –41 .24302514

[cit22] Tocanne J. F., Verheij H. M., Op Den Kamp J. A. F., Van Deenen L. L. M. (1974). Chem. Phys. Lipids.

[cit23] Esswein A., Rembold H., Schmidt R. R. (1990). Carbohydr. Res..

[cit24] See ESI pages 4–5 for details of gram-scale synthesis

