# Supplementary material for: A latent reactive handle for functionalising heparin-like and LMWH deca- and dodecasaccharides
Source: Org Biomol Chem. 2015 Dec 14;13(46):11208–19. doi: 10.1039/c5ob01706h (PMC4672752; doi:10.1039/c5ob01706h)

Electronic Supplementary Material (ESI) for Organic & Biomolecular Chemistry.
This journal is © The Royal Society of Chemistry 2015

Supporting Information

A latent reactive handle for functionalising heparin-like and LMWH deca- and dodecasaccharides

Gavin J. Miller,[a] Karl. R. Broberg,[a] Claire Rudd,[a] Madeleine R. Helliwell,[c] Gordon C. Jayson[b] and John M. Gardiner*[a]

[a] Manchester Institute of Biotechnology, School of Chemistry, The University of Manchester, 131 Princess Street, Manchester, M1 7DN, UK.
[b] Institute of Cancer Sciences, The University of Manchester, Wilmslow Road, Manchester M20 4BX, United Kingdom.
[c] School of Chemistry, The University of Manchester, 131 Princess Street, Manchester, M1 7DN, UK.

E-mail: gardiner@manchester.ac.uk

The following pages contains representative experimental protocols, data and spectra.

1. Synthetic procedures for compounds 4, 5, 10, 11, 12, 13, 14 and 15

All the chemicals used were purchased from commercial sources without further purification. All reactions were monitored by TLC on Merck silica gel plates $^{60}F_{254}$. Silica gel 60 (particle size 0.035-0.070 mm) was used for column chromatography. ^{1}H NMR spectra were recorded at 400 MHz and ^{13}C spectra at 100 MHz respectively on Bruker DPX spectrometers. Mass spectra (MS) were recorded using a Micromass Platform II spectrometer using an electro spray ionization source or via the EPSRC National Mass Spectrometry Service (Swansea). ESI spectra and isotope patterns for compounds with mass > 1000 are included with the NMR data. High resolution data are for the monoisotopic mass. Infrared spectra were obtained by using a Bruker Alpha instrument. Melting points were determined using Stuart Scientific SMP10 apparatus and are uncorrected. Optical rotations were obtained using an AA-1000 polarimeter. Elemental analyses were performed by Micro Analytical Laboratory, School of Chemistry, The University of Manchester. ESI MS conditions for final saccharide compounds: prior to MS, the sample for analysis was salt counterion switched from Na^+ to NH_4^+ using Amberlite IRC86-H^+ resin (pretreated with 5% aqueous NH_4OH solution).

N[1]-(7-nitrobenzo[*c*][1,2,5]oxadiazol-4-yl)ethane-1,2-diamine (4)

To a stirring solution of N-Boc ethylenediamine (180 μL, 1.14 mmol) and sodium hydrogen carbonate (239 mg, 2.08 mmol) in THF/EtOH (1:1, 2.0 mL) at 0 °C a solution of NBD-chloride (190mg, 0.95

mmol) in THF/EtOH (1:1, 2 mL) was added over 15 minutes. The reaction mixture was stirred at room temperature for 12 hours and then poured onto water. The aqueous layer was extracted with DCM (2 x 20 mL). The organic phases were combined, washed with brine, dried ($MgSO_4$), concentrated *in vacuo* and purified by silica gel flash chromatography eluting with DCM/MeOH, 2:1+5%NH_4OH to yield N-BOC protected **4** as a yellow/brown solid (266 mg, 0.82 mmol, 87%). R_f = 0.56 (DCM/MeOH, 9:1). Hydrochloric acid (240 μL, 3M) was added to a stirring solution of N-BOC protected **4** (226 mg, 0.70 mmol) in THF (3 mL) at 60 ^{o}C and the mixture was stirred for 4.5 hours, whereupon the reaction was shown to be complete by TLC analysis (DCM/MeOH, 9/1). The solution was concentrated *in vacuo* to yield **4** (150 mg, 0.67 mmol, 83%) as a yellow/brown solid. Mp**:** 236-238 ^{o}C; HRMS (TOF) *m/z* calcd for $C_8H_{10}N_5O_3$ $[M+H]^+$ 224.0779, found 224.0789. Other analytical data matched those reported previously.[1]

N-(6-aminohexyl)-4-(pyren-4-yl)butanamide hydrochloride (5)

Under an inert nitrogen atmosphere tert-butyl-N-(6-amino-hexyl)carbamate hydrochloride (228 mg, 0.90 mmol), 1-pyrenebutyric acid (216 mg, 0.75 mmol), TBTU (362 mg, 1.13 mmol) and DIPEA (392 μL, 2.25 mmol) were dissolved in anhydrous DMF (4 mL) and the mixture was stirred at 50 oC for 23 hours whereupon the reaction was shown to be complete by TLC analysis. The solution was diluted with diethyl ether (25 mL) and washed with water (5 x 10 mL). The organic phase was dried (MgSO4) and concentrated in vacuo. The product was purified by silica gel flash chromatography eluting with EtOAc/ hexane, 2/1) to yield N-BOC pyrene derivative 5 as an off- white solid (90 mg, 0.18 mmol, 25%). Rf = 0.61(EtOAc/hexane, 3:1). Hydrochloric acid (147 μL, 3M) was added to a stirring solution of N-BOC pyrene derivative 5 (85 mg, 0.18 mmol) in THF (2.0 mL) at 60 oC and the mixture was stirred for 4 hours whereupon the reaction was shown to be complete by TLC analysis (EtOAc/hexane, 2:1). The mixture was concentrated in vacuo to reveal 5 (70 mg, 0.18 mmol, 95%) as a white solid. Mp: 228-231 ºC; LRMS (ESI+) m/z 387.0 [M+H]+. Other analytical data matched those reported previously.[2]

Phenyl-4-*O*-allyl-2-azido-2-deoxy-3,6-di-*O*-benzyl-1-thio-α-D-glucopyranoside (10)

Thioglycoside **1**[18] (485 mg, 1.0 mmol) and sodium hydride (34 mg, 1.2 mmol, 60% dispersion in oil) were dissolved in dry DMF (10 mL) under nitrogen and the solution stirred at 0 °C for 30 minutes. Allyl bromide (132 μL, 1.5 mmol) was added to the stirring solution, the reaction temperature was then raised to 50 °C and stirred for 1 hour. Addition of water (5 mL) caused simultaneous quenching of remaining sodium hydride as well as precipitation of the allylated sugar from the reaction mixture. The product

was filtered and the filtrate extracted with DCM (30 mL) then washed with water (4 x 15 mL). The organic phase was dried ($MgSO_4$), filtered and solvents removed *in vacuo* to yield **10** as a white solid (41 mg). This was combined with the previously precipitated material (466 mg) to give an overall yield of **10** (507 mg, 0.98 mmol, 97%). mp 94-96 °C; $[\alpha]_D$ +154.2 (c = 10.0, DCM); ^{1}H NMR ($CDCl_3$, 400MHz) δ 7.52-7.26 (m, 15H, Ar*H*), 5.80-5.70 (ddt, *J* = 17.2, 10.4, 5.6 Hz, 1H, H_8), 5.60 (d, *J* = 5.6 Hz, 1H, H_1), 5.21 (ddd, *J* = 17.2, 3.2, 1.6 Hz, 1H, H_{9a}), 5.14 (ddd, 1H, *J* = 10.4, 2.8, 1.2 Hz, H_{9b}), 4.90 (d, *J* = 10.4 Hz, 1H, C*H*$_2$Ar), 4.87 (d, *J* = 10.8 Hz, 1H, C*H*$_2$Ar), 4.63 (d, *J* = 12.0 Hz, 1H, C*H*$_2$Ar), 4.87 (d, *J* = 12.0 Hz, 1H, C*H*$_2$Ar), 4.32 (ddd, 1H, *J* = 10.0, 3.6, 2.0 Hz, 1H, H_5), 4.27 (ddt, 1H, *J* = 12.4, 5.6, 1.6 Hz, H_{7a}), 4.02 (ddt, *J* = 12.0, 5.6, 1.2 Hz, 1H, H_{7b}), 3.91 (dd, *J* = 10.4, 5.2 Hz, 1H, H_2), 3.80-3.74 (m, 2H, H_3, H_{6a}), 3.66-3.63 (m, 1H, H_{6b}), 3.62 (dd, *J* = 7.6, 6.4 Hz, 1H, H_4); ^{13}C NMR (100 MHz; $CDCl_3$) δ 137.8, 137.7, 134.4, 132.0, 129.1, 128.5, 128.4, 128.2, 128.0, 127.9, 127.8, 127.7, 117.1, 87.2, 81.7, 78.1, 75.8, 73.9, 73.5, 71.8, 68.3, 63.9; MS ES $[M+Na]^+$ *m/z* 540.0; HRMS (TOF^+) *m/z* calcd for $C_{29}H_{31}N_3O_4SNa$ 540.1927, found 540.1919; IR (neat) ν_{max} 2923, 2853, 2105; Elemental analysis: calcd for $C_{29}H_{31}N_3O_4S$. C 67.3; H 6.0; N 8.1, found C 67.1; H 6.2; N 8.2.

Phenyl-2-azido-2-deoxy-3,6-di-*O*-benzyl-4-*O*-[(*S*/*R*)-2,3-dihydroxypropoxy]-1-thio-α-D-glucopyranoside (11)

The allylated glucosamine derivative **10** (500 mg, 0.97 mmol) was dissolved in an ice-cooled mixture of acetone:water (9:1, 5 mL). NMO (180 mg, 1.54 mmol) and osmium tetroxide (316 μL, 2.5 mol% in ^{t}BuOH) was added to the solution and the mixture stirred for 8 hours at 0 °C warming to room temperature. The reaction was quenched with 10% aqueous sodium thiosulfate (5 mL) followed by extraction of the product into DCM (3 x 60 mL). The combined organics were dried ($MgSO_4$), filtered and evaporated *in vacuo* to yield **11** (521 mg, 0.95 mmol, 98 %) as a white crystalline solid. R_f (hexane:EtOAc, 1:1) 0.24; ^{1}H NMR (400 MHz, $CDCl_3$) δ 7.50-7.48 (m, 2H, Ar*H*), 7.44-7.27 (m, 2H, Ar*H*), 5.98 (d, *J* = 5.2 Hz, 1H, H_1), 4.94 (d, *J* = 10.4 Hz, 1H, C*H*$_2$Ar), 4.81 (d, *J* = 10.8 Hz, 1H, C*H*$_2$Ar), 4.63 (d, *J* = 12.0 Hz, 1H, C*H*$_2$Ar), 4.46 (d, *J* = 12.0 Hz, 1H, C*H*$_2$Ar), 4.30- 4.24 (m, 1H, H_5), 3.92 (dd, *J* = 10.0, 5.2 Hz, 1H, H_2), 3.82-3.40 (m, 9H, H_3, H_4, H_{6ab}, H_7 H_8, H_9), 2.87 (d, *J* = 4.4 Hz, 1H, OH, C_{8major}), 2.69 (d, *J* = 5.2 Hz, 1H, OH, C_{8minor}), 1.86 (dd, *J* = 6.8, 5.2 Hz, 1H, OH, C_{9major}), 1.84 (dd, *J* = 7.2, 5.2 Hz, 1H, OH, C_{9minor}); ^{13}C NMR (100 MHz; $CDCl_3$) δ 137.5, 137.4, 137.2, 133.3, 132.0, 132.0, 129.1, 128.6, 128.5, 128.4, 128.3, 128.2, 128.1, 128.1, 127.9, 127.7, 87.2, 81.6, 78.8, 78.7, 75.9, 75.8, 74.3, 73.6, 73.6, 71.7, 71.6, 71.2, 71.1, 68.3, 68.2, 64.3, 64.2, 63.6, 63.5; IR (neat) ν_{max} 3422, 3061,

3031, 2922, 2869, 2104, 1045; MS ES $[M+Na]^+$ *m/z* 574.0; HRMS (TOF^+) *m/z* calcd for $C_{29}H_{33}N_3O_6SNa$ 574.1982, found 574.1981.

Phenyl-2-azido-2-deoxy-3,6-di-*O*-benzyl-4-*O*-[(*S/R*)-2,3-bis(benzyloxy)propoxy]-1-thio-α-D-glucopyranoside (12)

To a solution of **11** (742 mg, 1.34 mmol) in anhydrous DMF (10 mL) was added sodium hydride (83.5 mg, 2.09 mmol, 60% dispersion in oil) and the mixture was stirred for 20 minutes at room temperature. Benzyl bromide (480 μL, 4.0 mmol) was added and the reaction left to stir for 90 minutes at room temperature. The mixture was diluted with chloroform (80 mL), washed with water (4 x 20 mL) and then brine (20 mL). The organics were dried ($MgSO_4$), filtered and solvents removed *in vacuo* to give the crude product as a yellow gum. Purification by silica gel flash chromatography eluting with hexane/ EtOAc, 5/1, 4/1 furnished **12** (757 mg, 1.04 mmol, 77%). ^1H NMR (400 MHz, $CDCl_3$) δ 7.60-7.58 (m, 2H, Ar*H*), 7.47-7.44 (m, 2H, Ar*H*), 7.40-7.30 (m, 21H, Ar*H*), 5.65 (d, *J* = 5.2 Hz, 1H, H_1), 4.98-4.89 (m, 2H, CH_2Ar), 4.74-4.64 (m, 2H, CH_2Ar), 4.58-4.45 (m, 4H, CH_2Ar), 4.42-4.37 (m, 1H, H_5), 4.11-4.06 (m, 1H, H_8), 3.96 (dd, *J* = 10.4, 5.6 Hz, 1H, H_2), 3.86-3.70 (m, 5H, H_3, H_4, H_{6a}, H_7), 3.67-3.59 (m, 3H, H_{6a}, H_9); ^{13}C NMR (100 MHz; $CDCl_3$) δ 138.5, 138.4, 138.1, 138.0, 137.9, 137.7, 137.6, 133.6, 133.5, 132.1, 132.0, 129.1, 128.5, 128.4, 128.3, 128.2, 128.1, 127.9, 127.8, 127.7, 127.6, 87.2, 81.62, 81.56, 78.8, 78.7, 77.5, 77.3, 77.2, 75.6, 73.4, 73.3, 73.2, 72.4, 72.3, 71.8, 71.8, 70.0, 69.8, 68.4, 64.0, 63.9; MS ES $[M+Na]^+$ *m/z* 554.3; HRMS (FTMS-NSI^+) *m/z* calcd for $C_{43}H_{49}N_4O_6S_1$ $[M+NH_4]^+$ 749.3367, found 749.3368; IR (neat) ν_{max} 3061, 3029, 2864, 2104, 1088.

Phenyl-2-azido-2-deoxy-3,6-di-*O*-benzyl-4-*O*-[(*S*)-2,3-bis(benzyloxy)propoxy]-1-thio-α-D-glucopyranoside (15)

To **1** (611 mg, 1.28 mmol) was added dry THF (10 mL) under N_2 and the solution cooled to 0 °C. NaH (60% in mineral oil) (76.0 mg, 1.9 mmol) was added in two portions over 30 min. while being kept under N_2. **13**[22] (600 mg, 1.41 mmol) in dry THF (5 mL) was then added dropwise and the suspension allowed to warm to RT and heated at 50 °C overnight. TLC analysis (4/1, hexane/EtOAc) showed the reaction to be complete and quenching was effected with aqueous $NaHCO_3$ (1 mL). The solution was partitioned between EtOAc and H_2O. The layers were separated and the organic phase washed with 1M HCl, H_2O, saturated aqueous NaCl, dried ($MgSO_4$), filtered and evaporated. The crude product was purified twice by flash column chromatography (EtOAc/hexane gradient 1:10, 1:8, then DCM/Ether gradient 99:1) yielding **15** (115 mg, 0.16 mmol, 21%) as

a clear oil. R_f 0.40 (EtOAc/hexane, 1:4); []$_D$ -19.4 (c = 0.7, DCM); ^{1}H NMR (400 MHz; $CDCl_3$) δ 7.44-7.39 (m, 2H, Ar*H*), 7.30-7.25 (m, 2H, Ar*H*), 7.25-7.11 (m, 21H, Ar*H*), 5.48 (d, *J* = 5.3 Hz, 1H, H_1), 4.80 (d, *J* = 10.6 Hz, 1H, CH_2Ar), 4.76 (d, *J* = 10.6 Hz, 1H, CH_2Ar), 4.55 (d, *J* = 11.9 Hz, 1H, CH_2Ar), 4.51 (d, *J* = 11.9 Hz, 1H, CH_2Ar), 4.39 (s, 2H, CH_2Ar), 4.35 (d, *J* = 12.0 Hz, 1H, CH_2Ar), 4.28 (d, *J* = 12.0 Hz, 1H, CH_2Ar), 4.23 (ddd, *J* = 9.9, 3.8, 1.7 Hz, 1H, H_5), 3.94-3.91 (m, 1H, CH_2C*H*[OBn]CH_2OBn), 3.79 (dd, *J* = 10.3, 5.3 Hz, 1H, H_2), 3.69-3.63 (m, 2H, H_3, H_4), 3.63-3.52 (m, 3H, H_{6A}, CH_2CH[OBn]CH_2OBn), 3.49-3.41 (m, 3H, H_{6B}, CH_2CH[OBn]CH_2OBn); ^{13}C NMR (100 MHz; $CDCl_3$) δ 138.6, 138.1, 138.0, 137.8, 133.7, 132.1, 129.1, 128.5, 128.4, 128.3, 128.2, 127.9, 127.8, 127.7, 127.6, 87.3, 81.6, 78.9, 77.4, 75.6, 73.5, 73.4, 73.3, 72.5, 71.9, 69.8, 68.5, 64.0; MS ES [M+Na]$^+$ *m/z* 754.0; HRMS (ES-TOF$^+$) *m/z* calcd for $C_{43}H_{45}N_3O_6SNa$ [M+Na]$^+$ 754.2921, found 754.2918.

1-*O*-*p*-toluene-sulfonyl-(S)-2,3-di-O-benzylglycerol (13)

(*S*)-2,3-Di-*O*-benzyloxypropanol (700 mg, 2.57 mmol) was dissolved in dry DCM (10 mL) under nitrogen. To this stirred pale yellow solution was added Et_3N (522 µL, 3.5 mmol), p-TsCl (540 mg, 2.83 mmol) and DMAP (16.0 mg, 0.13 mmol). Stirring was continued at room temperature for 24 h whereupon TLC analysis indicated no starting material remained. DCM (10 mL) was added and the organics washed with 1M HCl, H_2O, saturated aqueous NaCl, dried ($MgSO_4$), filtered and evaporated. The crude material was then purified by silica gel flash chromatography eluting with hexane/EtOAc, 8/1, 4/1 to yield **13** (680 mg, 1.60 mmol, 62%) as clear oil. R_f = 0.75 (hexane/EtOAc, 2/1); ^{1}H NMR (400 MHz; $CDCl_3$) δ 7.80-7.78 (m, 2H, ArH), 7.36-7.26 (m, 12H, ArH), 4.59 (s, 2H, CH_2Ar), 4.49 (s, 2H, CH_2Ar), 4.24 (dd, J = 10.4, 4.2 Hz, 1H, CH_2OTs), 4.14 (dd, J = 10.4, 5.8 Hz, 1H, CH_2OTs), 3.82-3.80 (m, 1H, CHOBn), 3.54 (dd, J = 5.2, 0.7 Hz, 2H, CH_2OBn), 2.44 (s, 3H, ArCH_3); MS ES [M +Na]$^+$ m/z 449.0; HRMS (ES-TOF$^+$) m/z calcd for $C_{24}H_{26}O_5NaS$ [M+Na]$^+$ 449.1393, found 449.1390. Other analytical data matched those previously reported.[3]

1-*O*-trifluoromethanesulfonyl-(*S*)-2,3-di-*O*-benzyl-glycerol (14)

(*S*)-2,3-Di-O-benzyloxypropanol (3.0 g, 11.0 mmol) was dissolved in dry DCM (25 mL) under nitrogen. To this stirred pale yellow solution was added lutidine (2.10 mL, 17.6 mmol) and Tf_2O (2.80 mL, 16.5 mmol) at 0 °C. Stirring was continued for 1 h whereupon TLC analysis indicated no starting material remained. The volume of DCM was reduced to *ca* 5 mL *in vacuo*, the material loaded onto a short silica plug and eluted with hexane/EtOAc, 20/1 to yield **14** (4.40 g, 11.0 mmol, quant.) as clear oil. R_f = 0.85 (hexane/EtOAc, 3/1); ^{1}H NMR (400 MHz; $CDCl_3$) δ 7.41-7.32 (m, 10H, Ar*H*), 4.71-4.60 (m, 4H, C*H*$_2$Ar, C*H*$_2$OTf), 4.59-4.52 (m, 2H, C*H*$_2$Ar), 3.93-3.88 (m, 1H, C*H*), 3.65-3.56 (m, 2H, C*H*$_2$OBn). The

material was stored under nitrogen in a freezer until use. Other analytical data matched those previously reported.[4]

References:

1. A. Cotte, B. Bader, J. Kuhlmann and H.Waldmann, *Chem.-Eur. J.*, 1999, **5**, 922.
2. K. C. Etika, F. D. Jochum, M. A. Cox, P. Schattling, P. Theato and J. C. Grunlan, *Macromolecules*, 2010, **43**, 9447.
3. J. F. Tocanne, H. M. Verheij, J. A. F. Op Den Kamp and L. L. M. Van Deenen, *Chemistry and Physics of Lipids*, 1974, **13**, 389.
4. A. Esswein, H. Rembold and R. R. Schmidt, *Carbohydr. Res.*, 1990, **287**, 305.

Synthesis of (S)-2,3-dibenzyoxy-1-propanol from D-mannitol

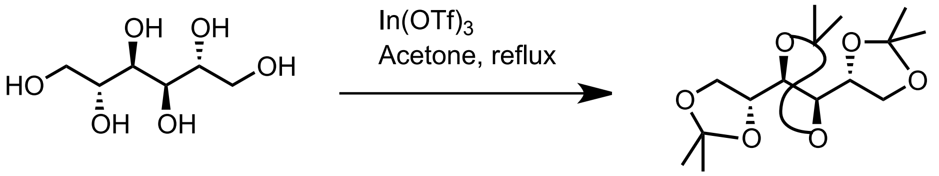

D-mannitol (5.0 g, 27.4 mmol) and $In(OTf)_3$ (462 mg, 0.82 mmol) were suspended in acetone (50 mL) at RT under air. The suspension was heated to 65 ^oC for 3 h whereupon the suspension became clear. TLC analysis (DCM/MeOH, 4/1) showed the reaction to be complete and the solvent was removed in vacuo. The resulting white paste was dissolved in DCM (200 mL) and washed with water (50 mL). The organic phase was dried ($MgSO_4$), filtered and stripped to a clear oil which solidified upon high vacuum removal of final solvent traces to give the tris-dimethylacetal product (6.48 g, 21.5 mmol, 78%) as a while solid.

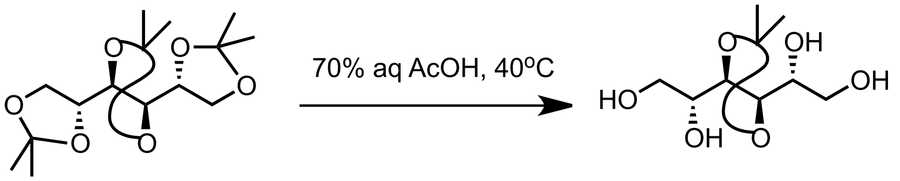

Tris-dimethylacetal-D-mannitol derivative (4.4 g, 14.6 mmol) was dissolved in MeOH (50 mL) at RT under air. 0.5M H_2SO_4 (5 mL) was added and the solution heated at 40 ^oC for 2 h. TLC analysis (hexane/EtOAc, 3/1) showed no starting material remained and the reaction was cooled to RT, neutralized to pH = 8 with 1M NaOH, the suspension filtered and solvent removed in vacuo. The crude oil was then purified by silica gel flash chromatography eluting with DCM/MeOH, 9/1 to furnish the product tetra-ol (2.18 g, 9.8 mmol, 67%) was a white solid.

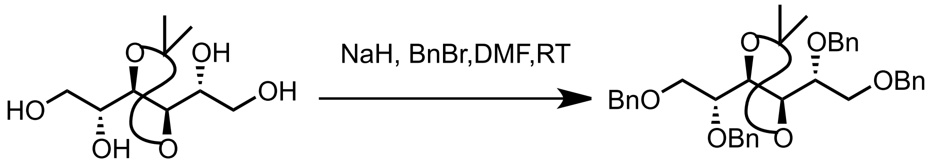

Tetra-ol derivative (2.20 g, 9.9 mmol) was dissolved in dry THF (50 mL) at RT under N_2. The solution was cooled to 0 ^oC in an ice bath and NaH (1.98 g, 50.0 mmol, 60% in mineral oil) added portionwise over 15 minutes. The suspension was stirred at 0 ^oC for 30 minutes, BnBr (11.8 mL, 99.1 mmol) and TBAI (3.66 g, 9.91 mmol) were then added and the solution warmed to RT and stirred overnight. TLC analysis (DCM/MeOH, 4/1) showed no starting material and one product spot. The reaction solution was cooled once again to 0 ^oC and quenched with water (10 mL). This solution was then poured onto diethyl ether (100 mL) and the layers separated. The aqueous phase was extracted with diethyl ether (3 x 20

mL), the organics combined, dried ($MgSO_4$), filtered and stripped to a brown oil. This crude material was then purified by silica gel flash chromatography eluting firstly with hexane/EtOAc, 6/1 and then with hexane/diethyl ether, 4/1 to give the tetrabenzylated product (5.42 g, 9.30 mmol, 93%) as a pale pink oil.

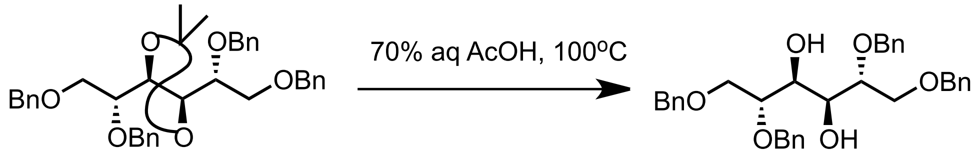

The tetrabenzylated-D-mannitol derivative (4.24 g, 7.3 mmol) was suspended in 60% aq. AcOH (40 mL) and heated to reflux overnight. TLC analysis (hexane/EtOAc, 4/1) showed no starting material and one product spot. The solvent was removed in vacuo azeotroping with toluene (2 x 100 mL) to give the crude material as a yellow oil which was purified by silica gel flash chromatography, eluting with hexane/EtOAc, 4/1, 2/1 to give the product 3,4-diol (2.85 g, 5.25 mmol, 72%) as a clear oil.

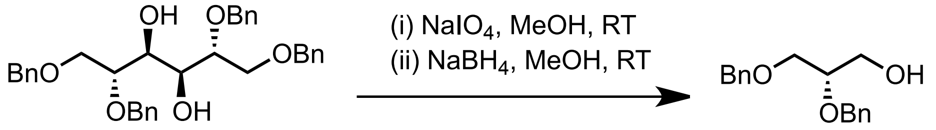

The D-mannitol-3,4-diol (2.70 g, 4.98 mmol) was dissolved in MeOH (50 mL) and cooled to 0 °C. $NaIO_4$ (1.20 g, 5.63 mmol) was added and the thick white suspension stirred vigorously for 30 minutes, filtered through Celite, washing with MeOH (25 mL) and then re-cooled to 0 °C whereupon $NaBH_4$ (259 mg, 4.48 mmol) was added and the suspension stirred for a further 2 h at 0 °C. TLC analysis (hexane/ EtOAc, 3/1) showed a spot that matched the commercially available material and the reaction was quenched at 0 °C with glacial acetic acid. The suspension was filtered through Celite and the majority of the MeOH removed in vacuo. The residue was partitioned between EtOAc (100 mL) and brine (25 mL). The layers were separated and the organics extracted with EtOAc (3 a 25 mL), combined, dried ($MgSO_4$), filtered and solvent removed in vacuo to give the crude material as a yellow oil. This was purified by silica gel flash chromatography eluting with hexane/EtOAc, 4/1 to give (S)-2,3-dibenzyoxy-1-propanol (1.86 g, 6.8 mmol, 69%) as a clear oil. ^1H NMR and optical rotation data matched those available from a commercial supplier (Sigma Aldrich).

2. Spectral Data: ^{1}H, ^{13}C, COSY, HMQC/HSQC NMR and MS data for compounds 2-27

Phenyl 2-azido-3,6-di-O-benzyl-4-O-acetoxyethyl-2-deoxy-1-thio-α-D-glucopyranoside (α-2)

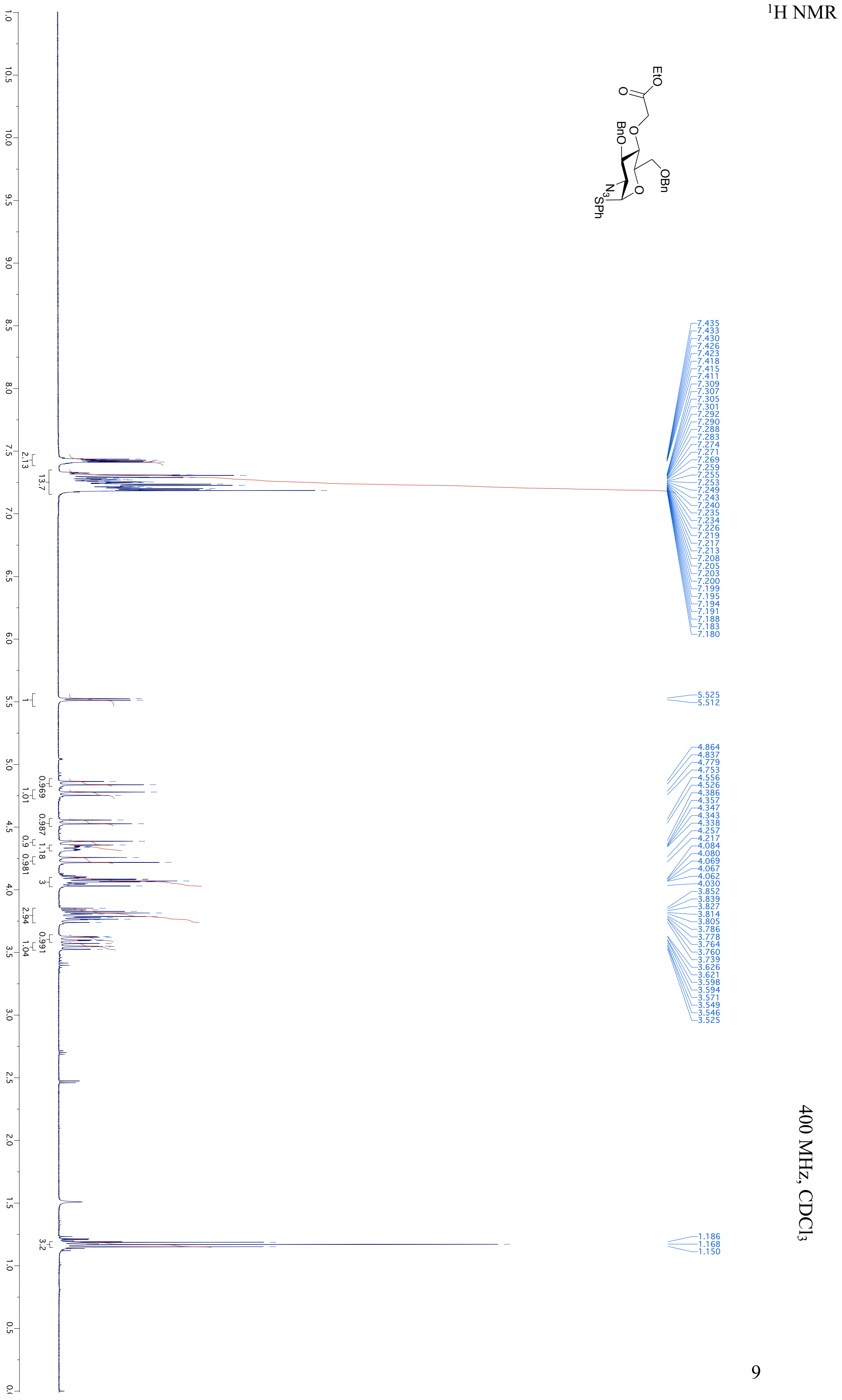

Phenyl 2-azido-3,6-di-O-benzyl-4-O-acetoxyethyl-2-deoxy-1-thio-α-D-glucopyranoside (α-2)

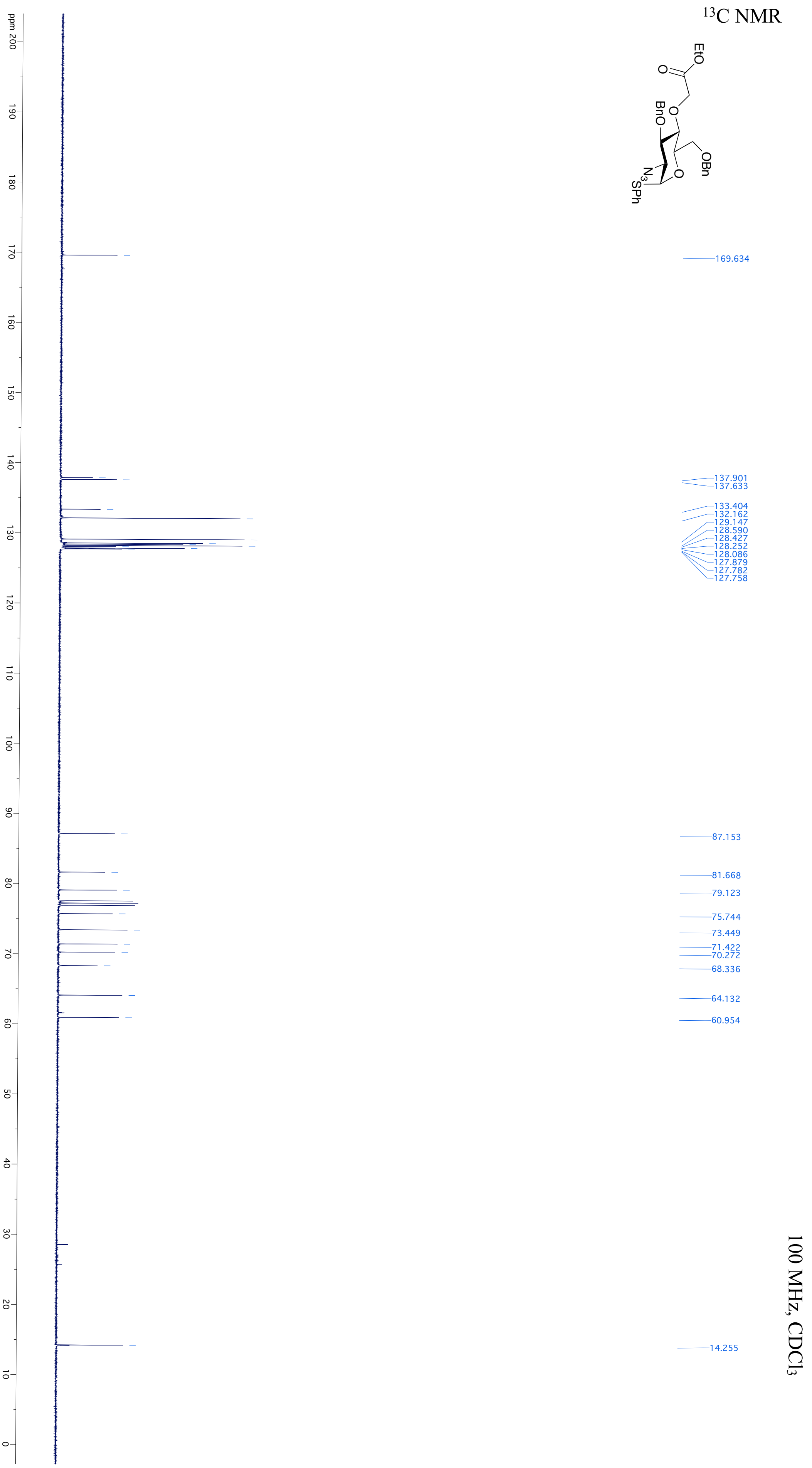

Phenyl 2-azido-3,6-di-O-benzyl-4-O-acetoxy-2-deoxy-1-thio-α-D-glucopyranoside (α-3)

^{1}H NMR

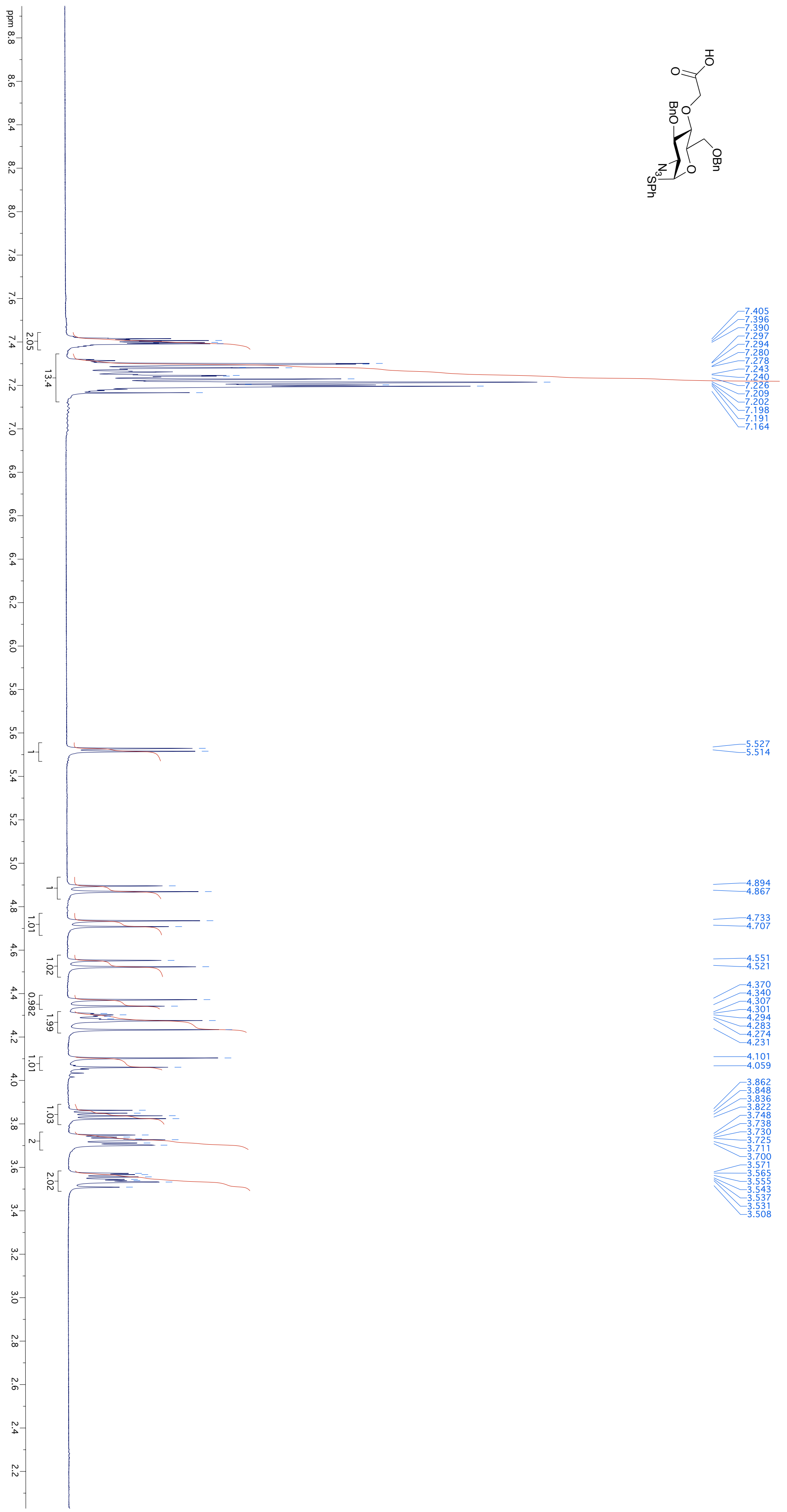

400 MHz, $CDCl_3$

Phenyl 2-azido-3,6-di-O-benzyl-4-O-acetoxy-2-deoxy-1-thio-α-D-glucopyranoside (α-3)

^{13}C NMR

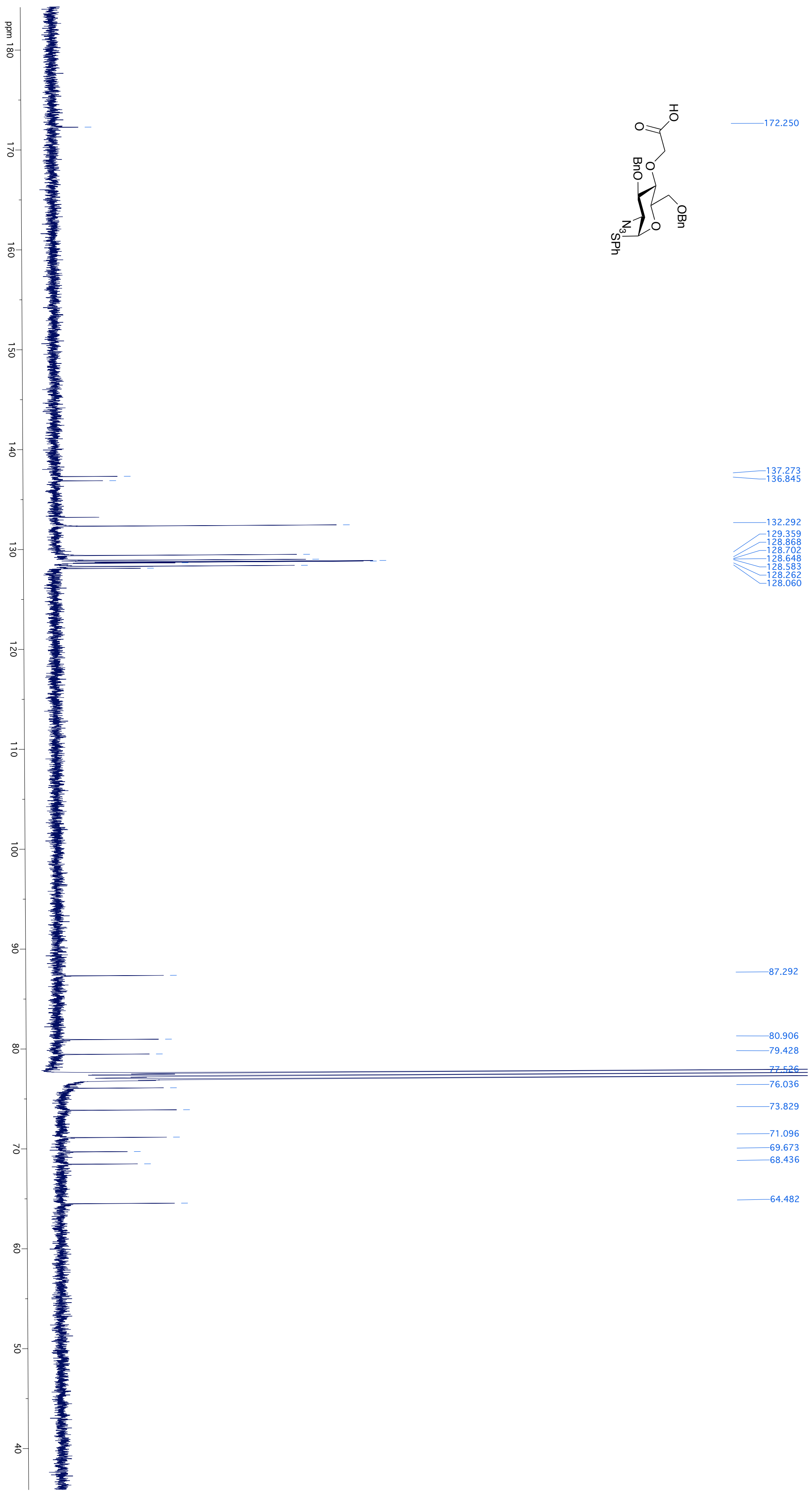

100 MHz, $CDCl_3$

Phenyl 2-azido-3,6-di-O-benzyl-4-O-acetoxy-2-deoxy-1-thio-α-D-glucopyranoside (α-3)

COSY

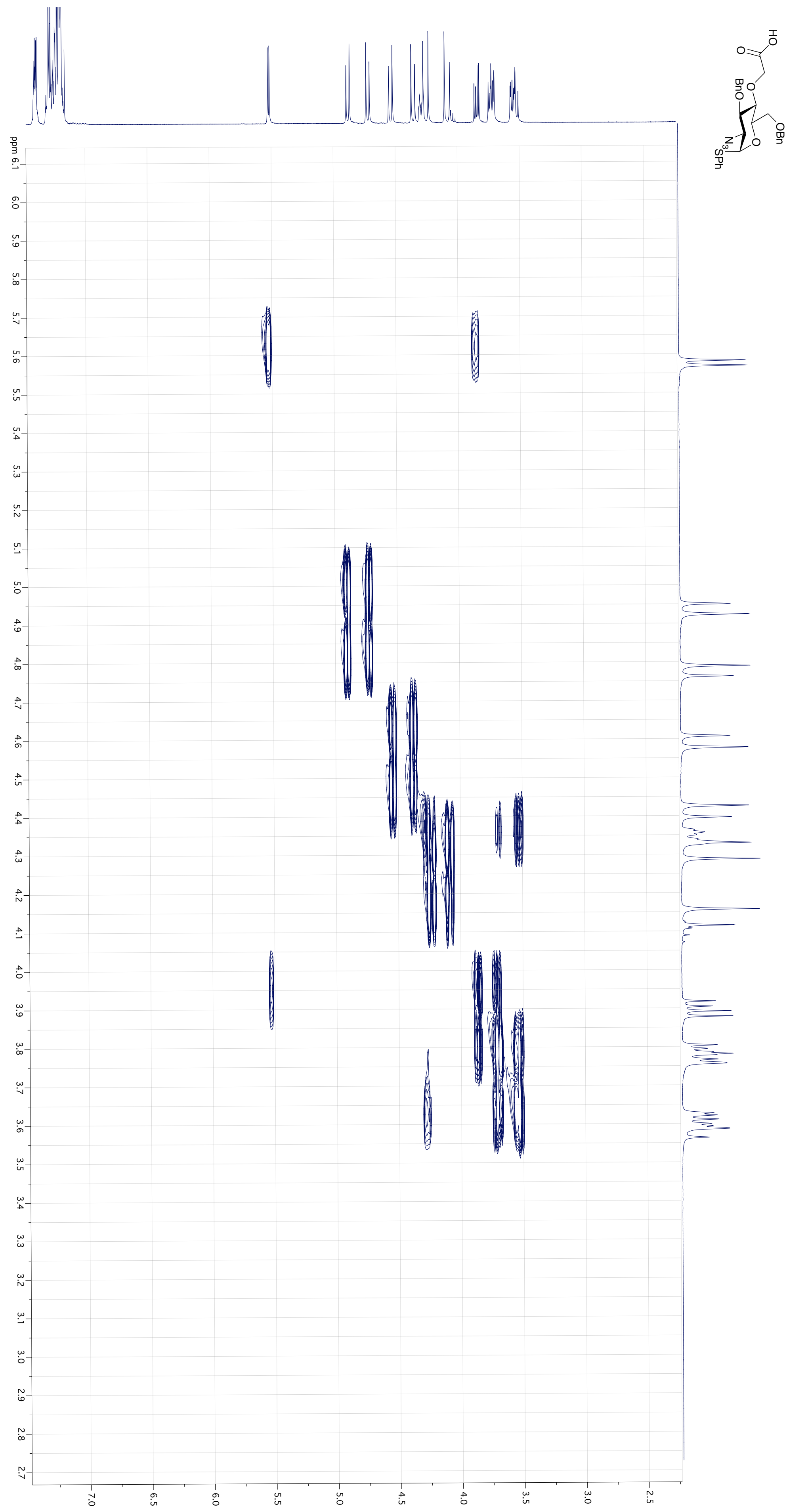

400 MHz, $CDCl_3$

Phenyl 2-azido-3,6-di-O-benzyl-4-O-acetoxy-2-deoxy-1-thio-α-D-glucopyranoside (α-3)

HMQC

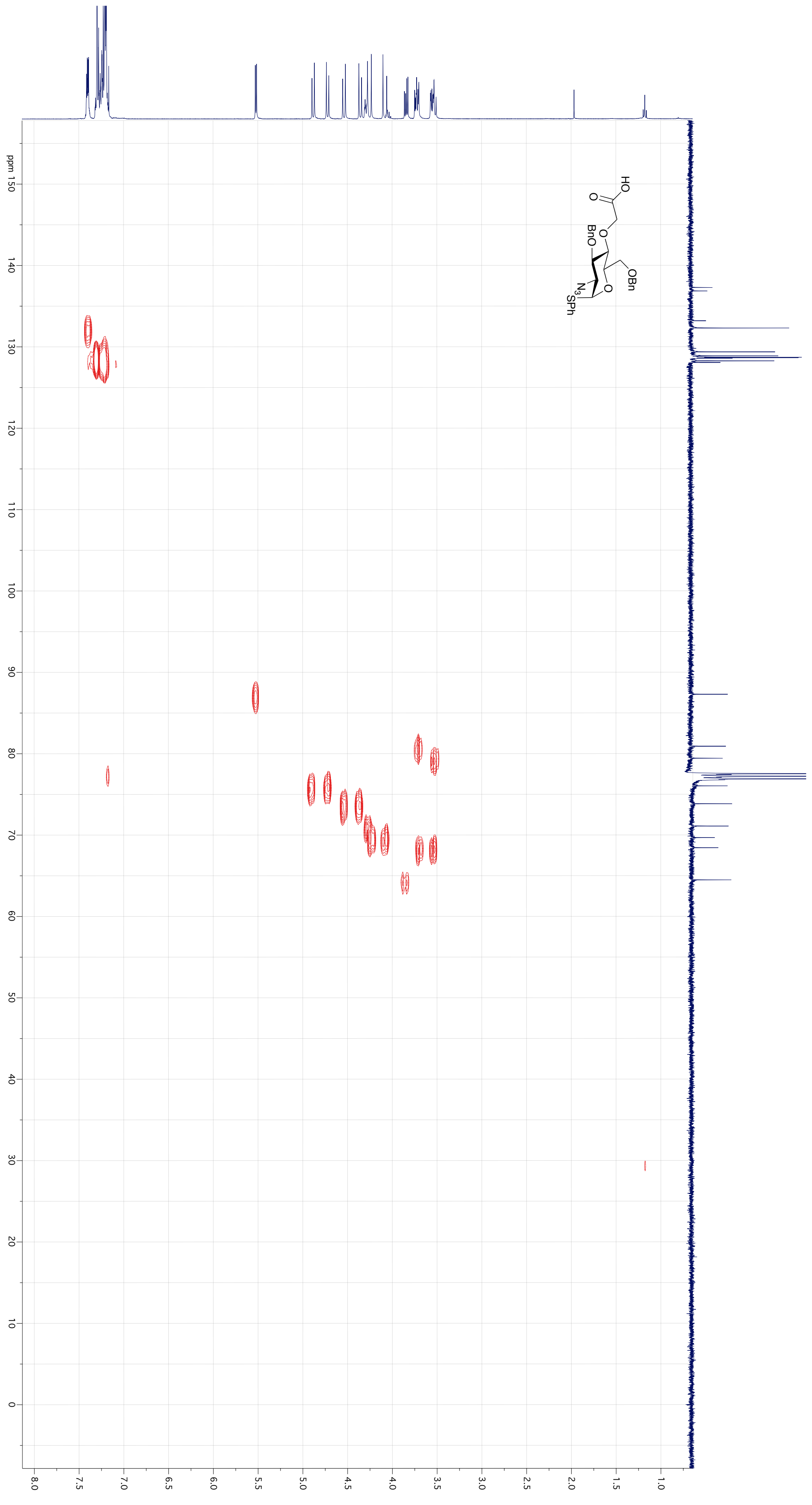

400 MHz, $CDCl_3$

NBD-D-GlcN derivative (6)

^{1}H NMR

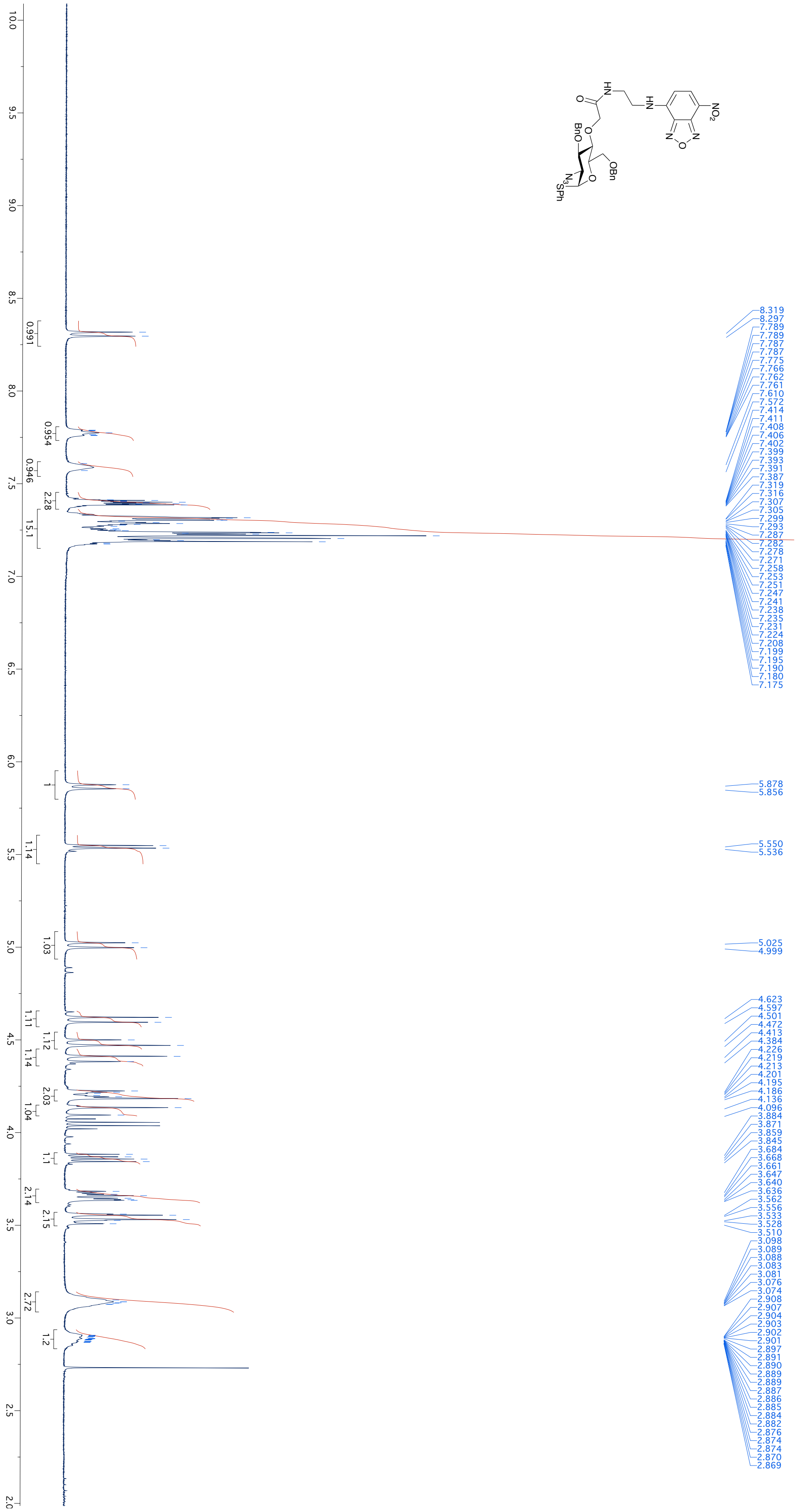

400 MHz, $CDCl_3$

NBD-D-GlcN derivative (6)

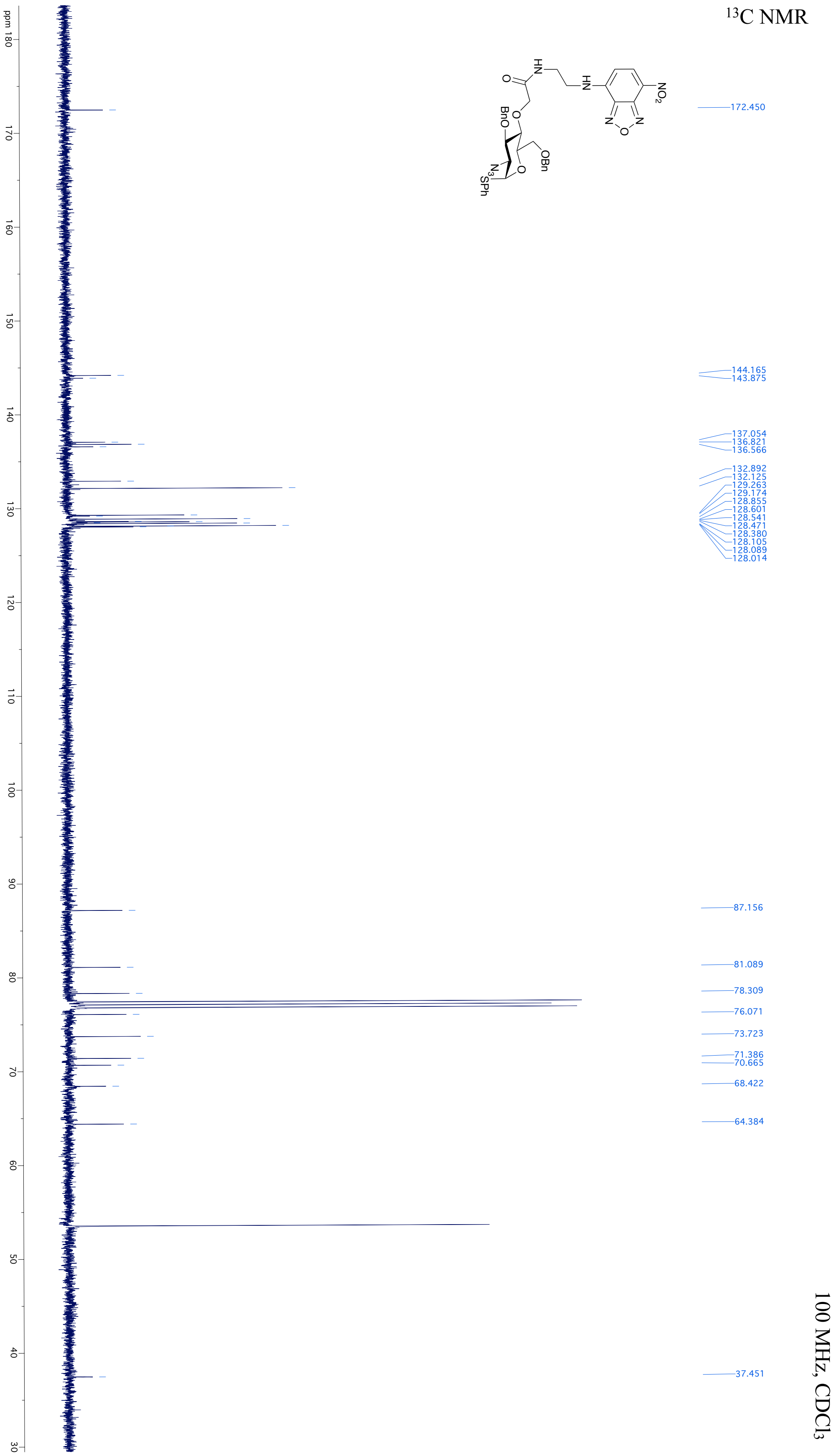

NBD-D-GlcN derivative (6)

COSY

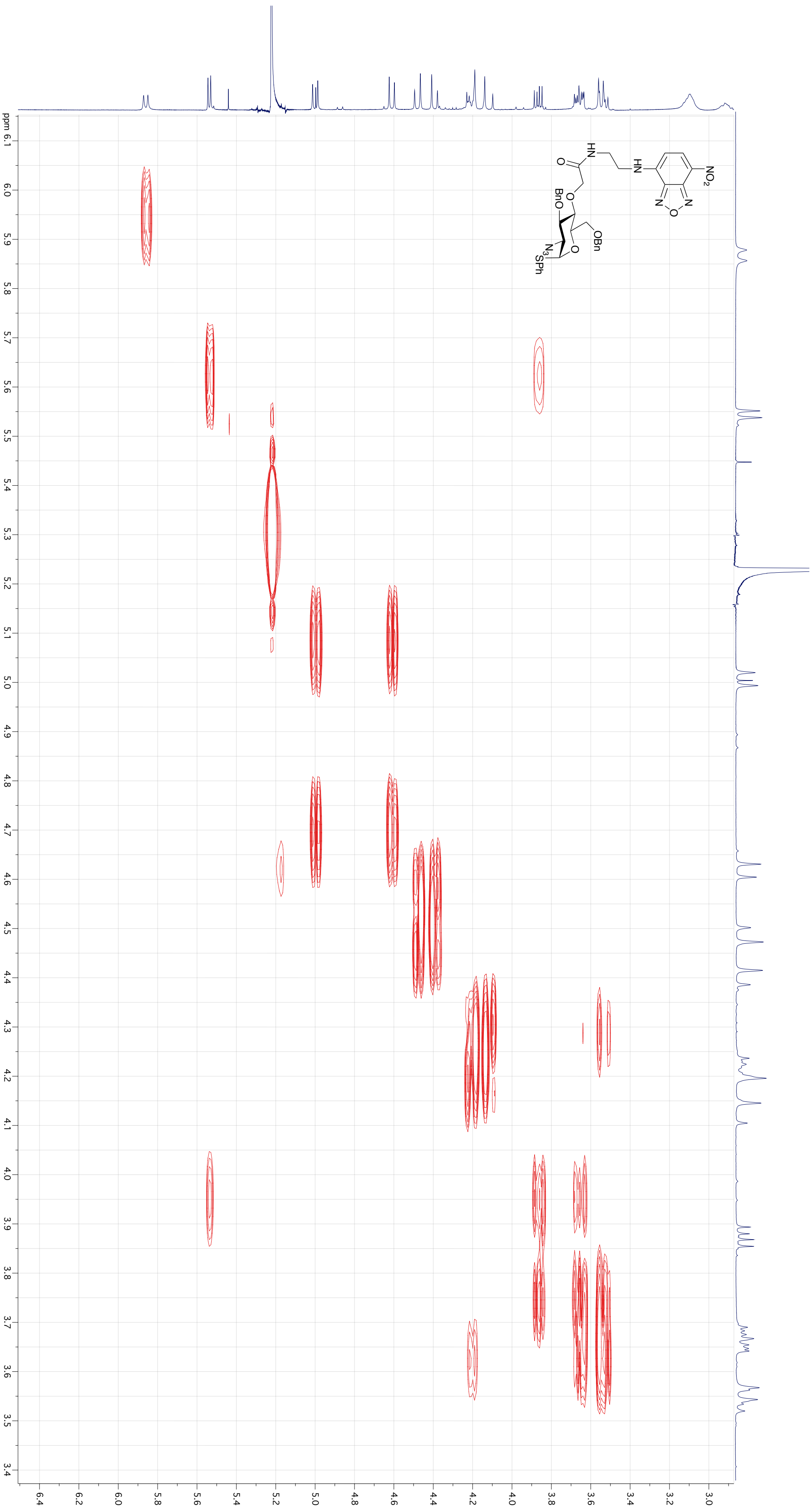

400 MHz, $CDCl_3$

NBD-D-GlcN derivative (6)

HMQC

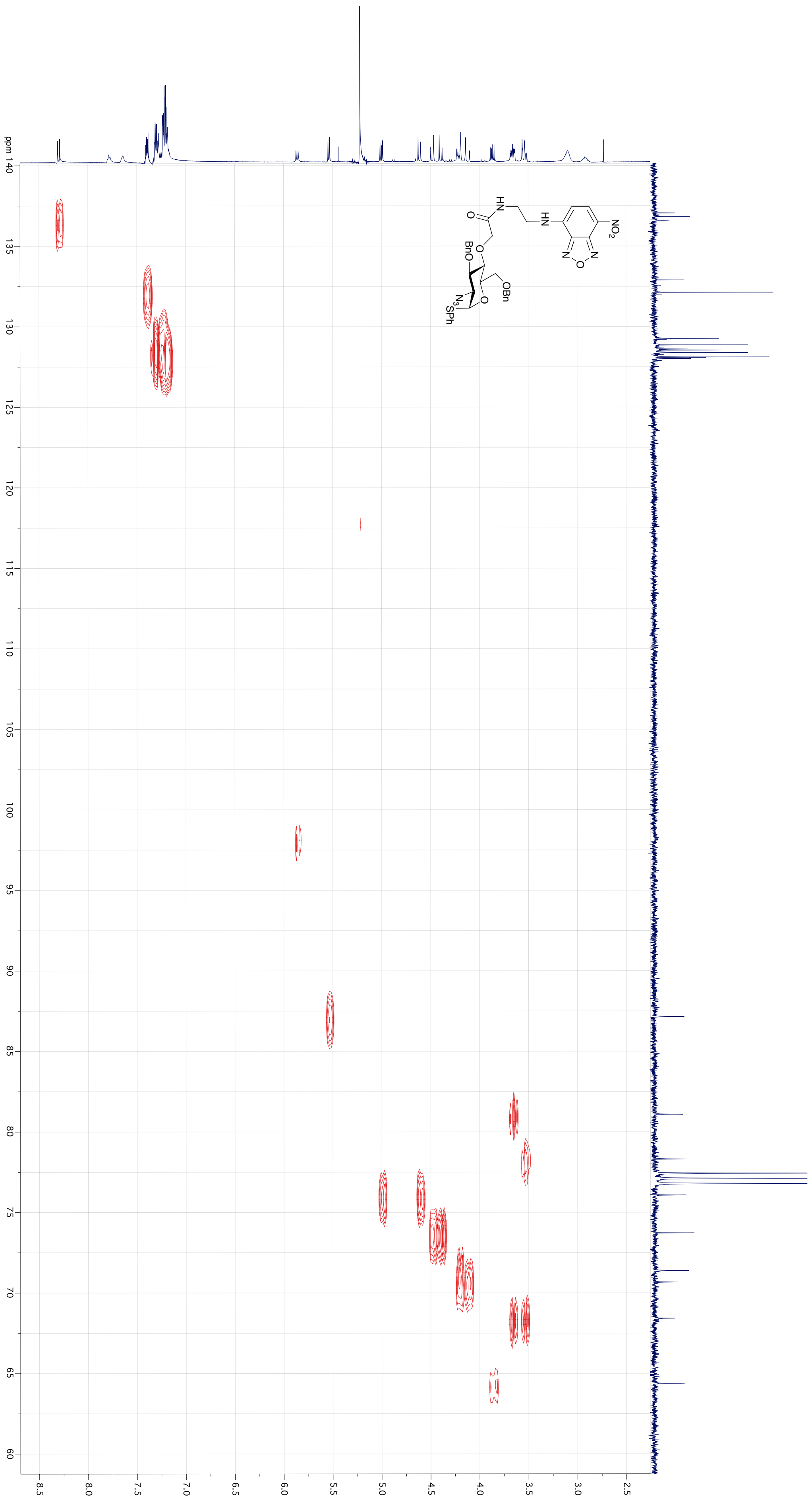

400 MHz, $CDCl_3$

NBD-D-GlcN derivative (6)

UV Spectrum

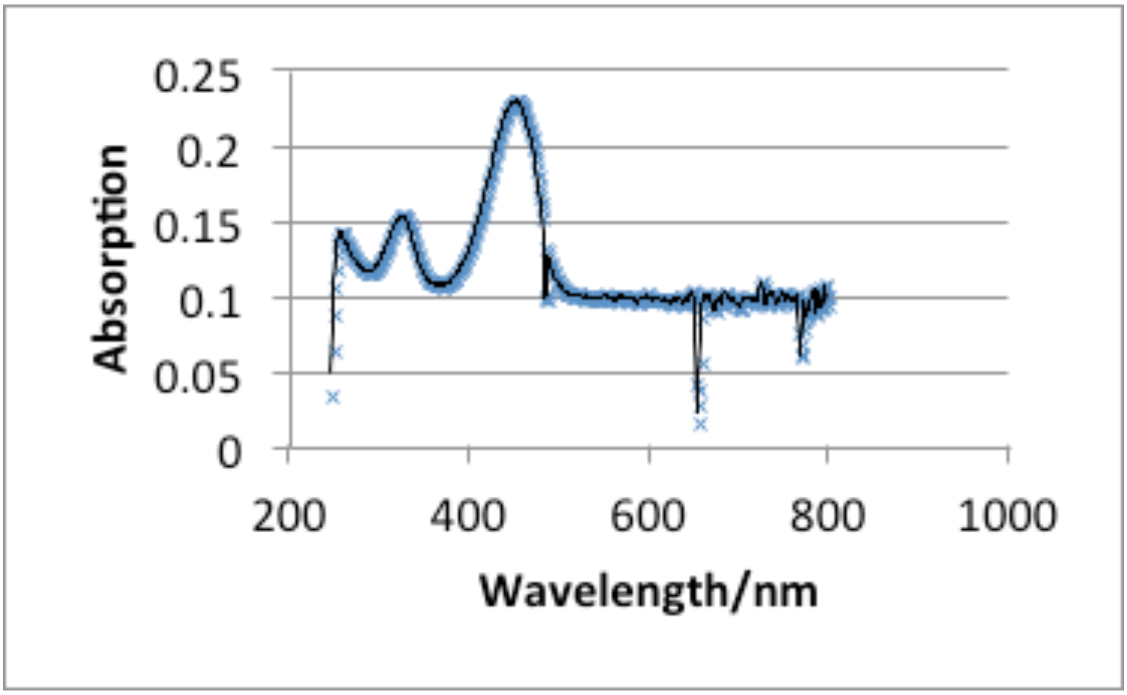

Fluorescence Spectrum

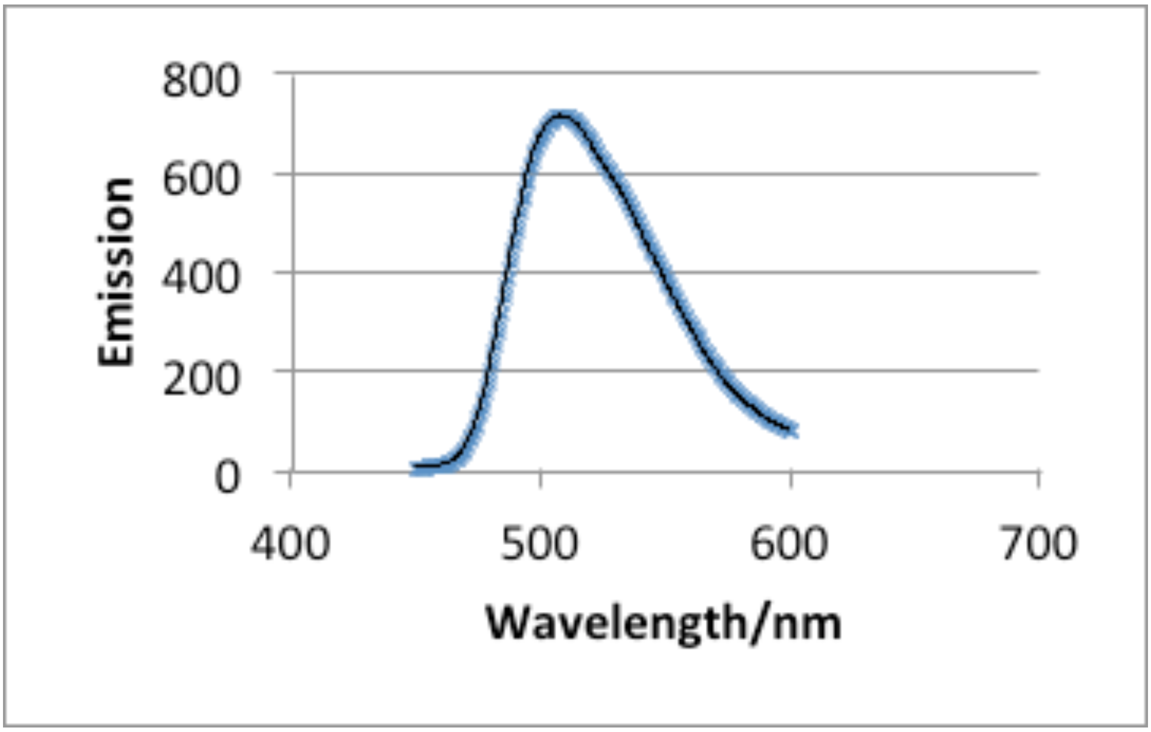

Pyrene-D-GlcN derivative (7)

^{1}H NMR

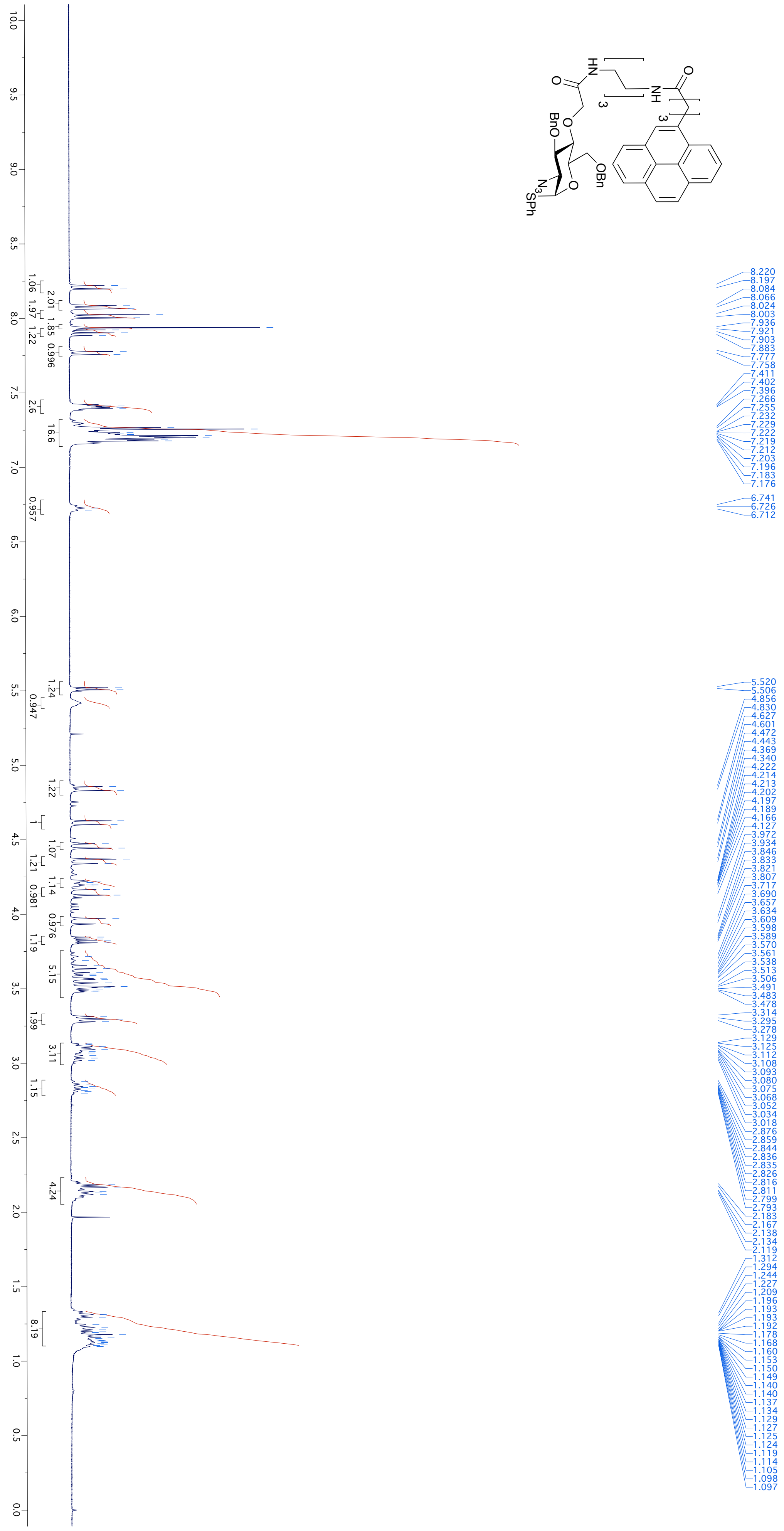

400 MHz, CDCl$_3$

Pyrene-D-GlcN derivative (7)

^{13}C NMR

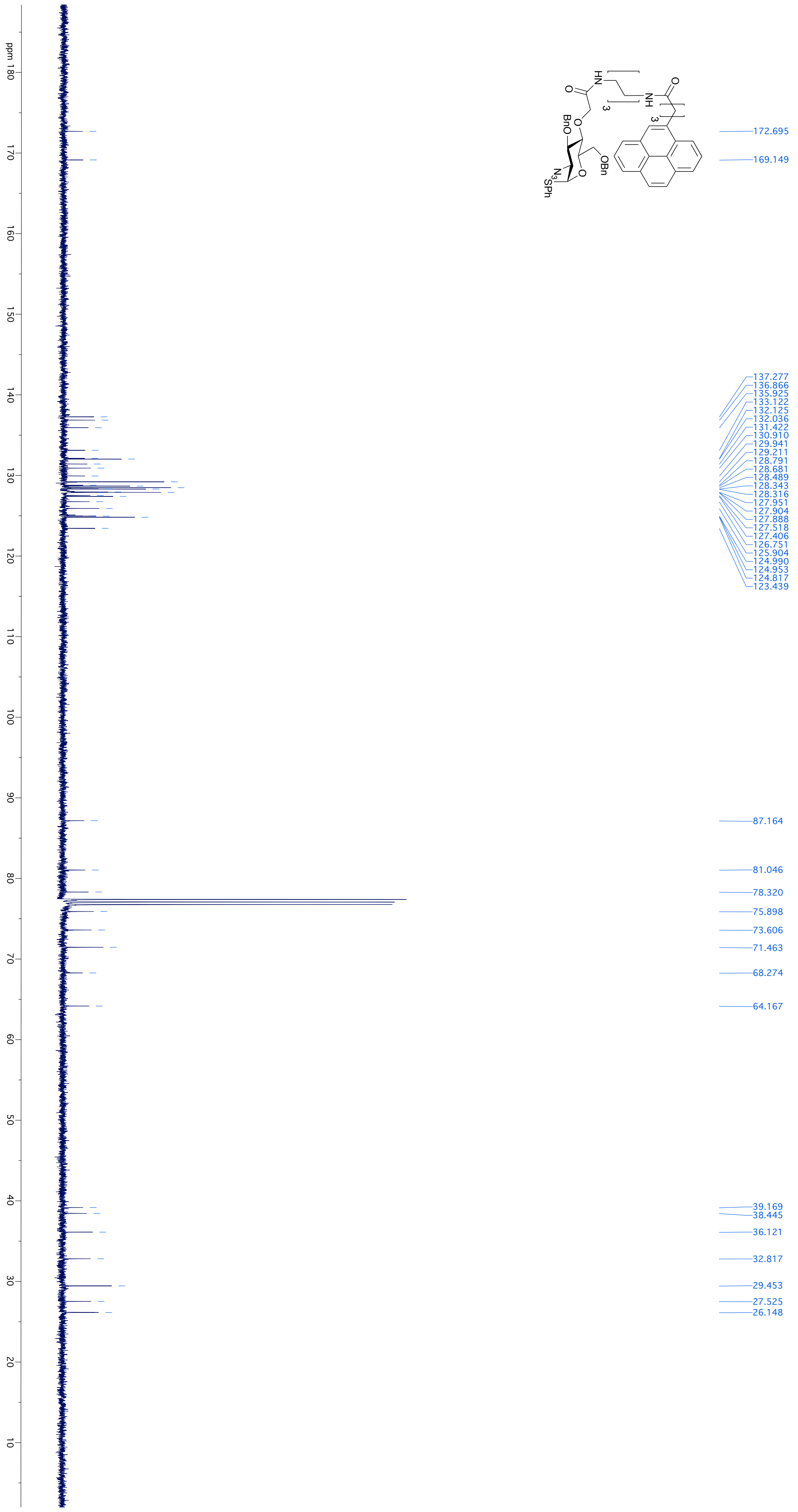

100 MHz, $CDCl_3$

Pyrene-D-GlcN derivative (7)

COSY

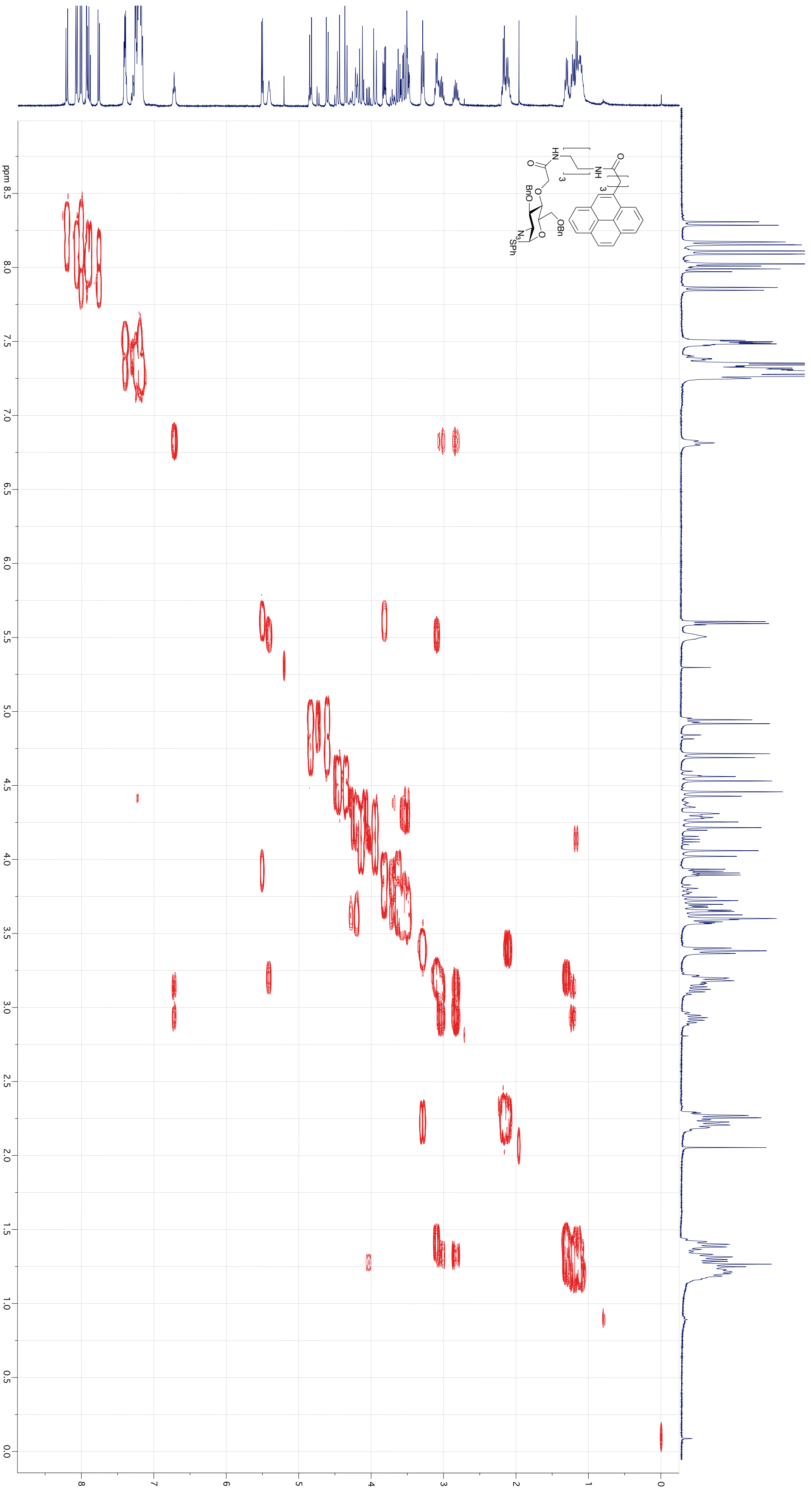

400 MHz, $CDCl_3$

Pyrene-D-GlcN derivative (7)

HMQC

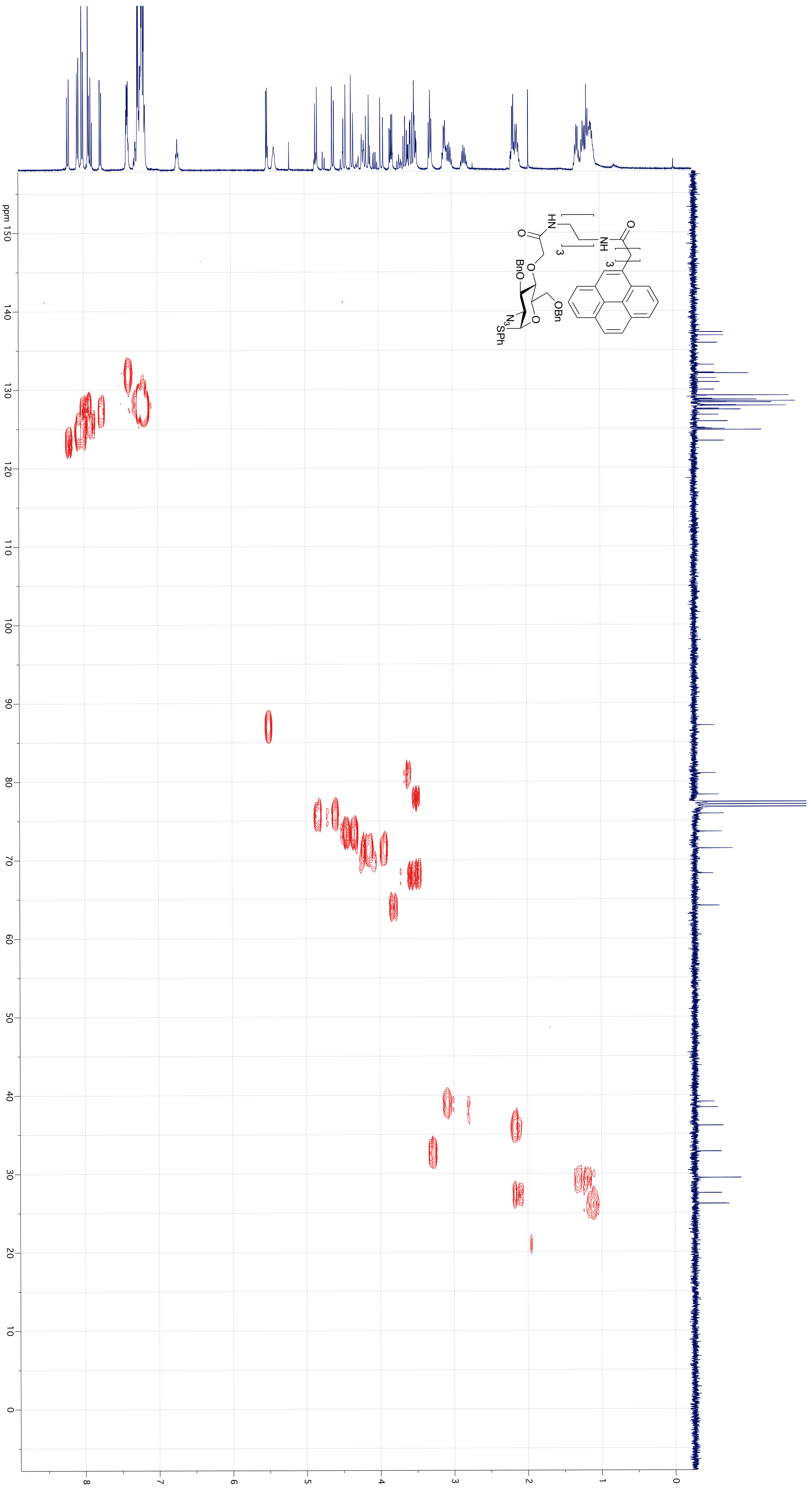

400 MHz, $CDCl_3$

Pyrene-D-GlcN derivative (7)

UV Spectrum

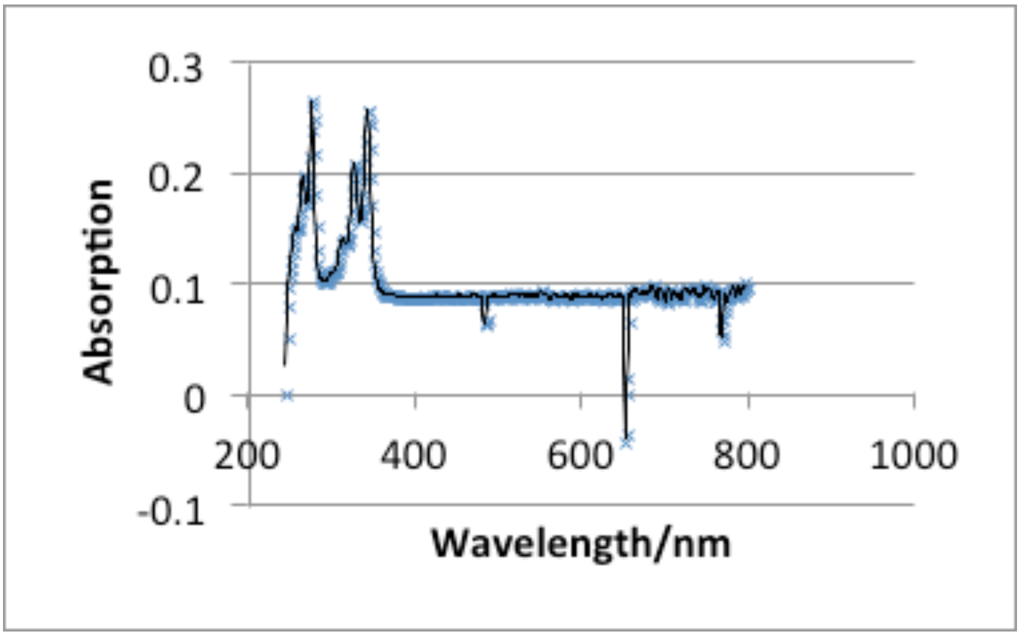

Fluorescence Spectrum

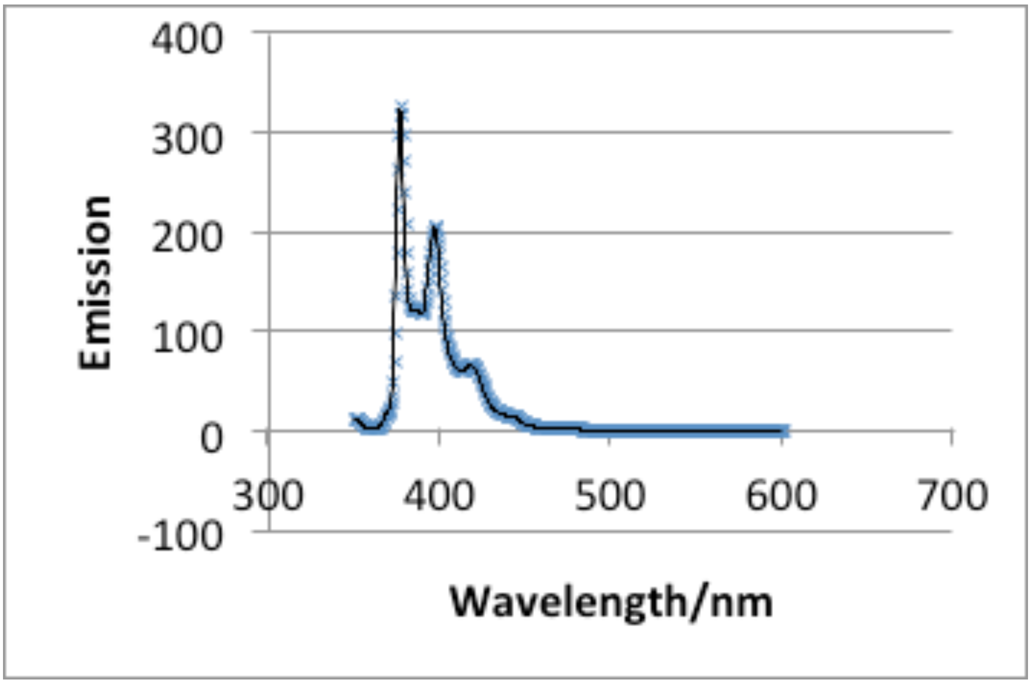

Phenyl-4-O-allyl-2-azido-2-deoxy-3,6-di-O-benzyl-1-thio-α-D-glucopyranoside (10)

^{1}H NMR

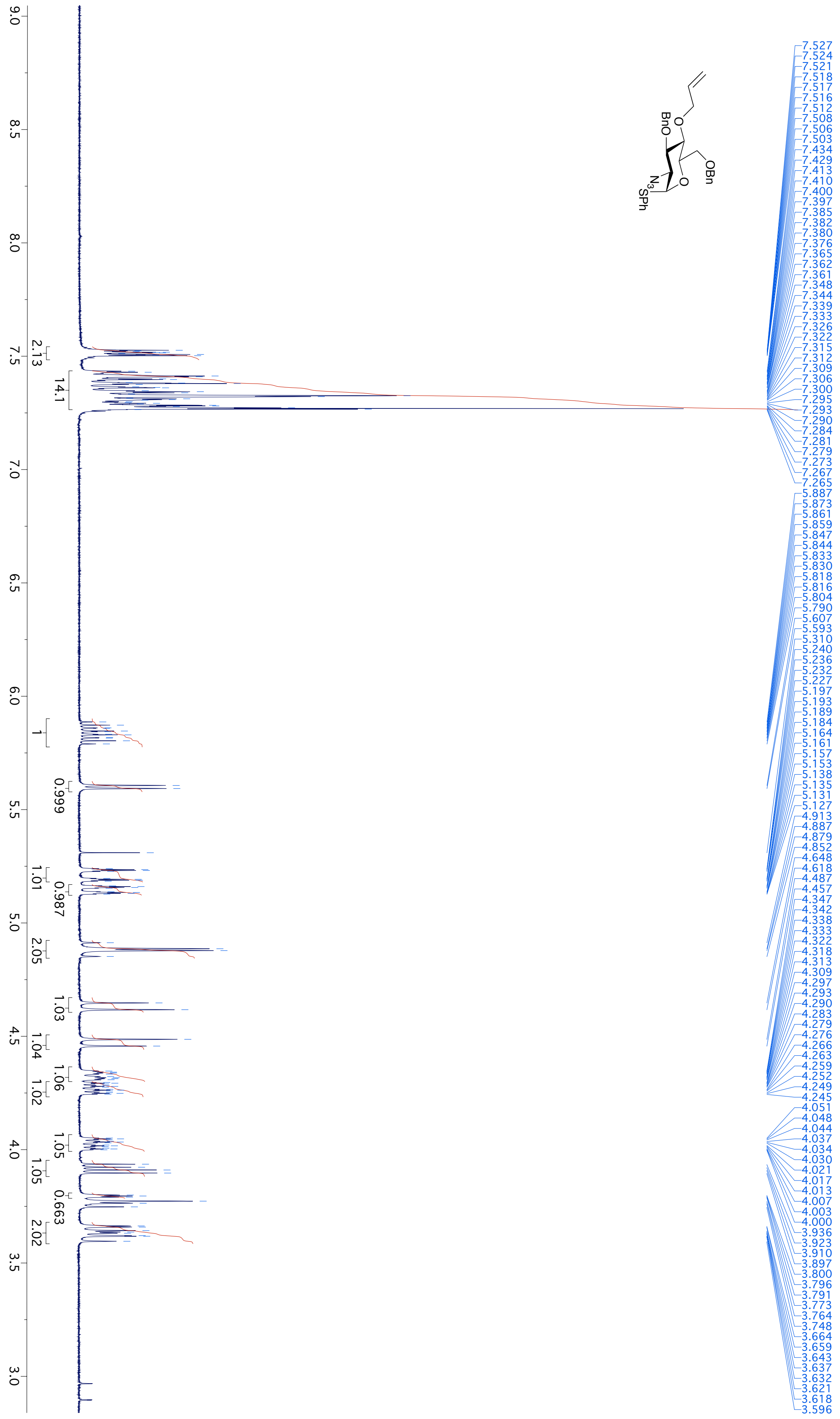

400 MHz, $CDCl_3$

Phenyl-4-O-allyl-2-azido-2-deoxy-3,6-di-O-benzyl-1-thio-α-D-glucopyranoside (10)

^{13}C NMR

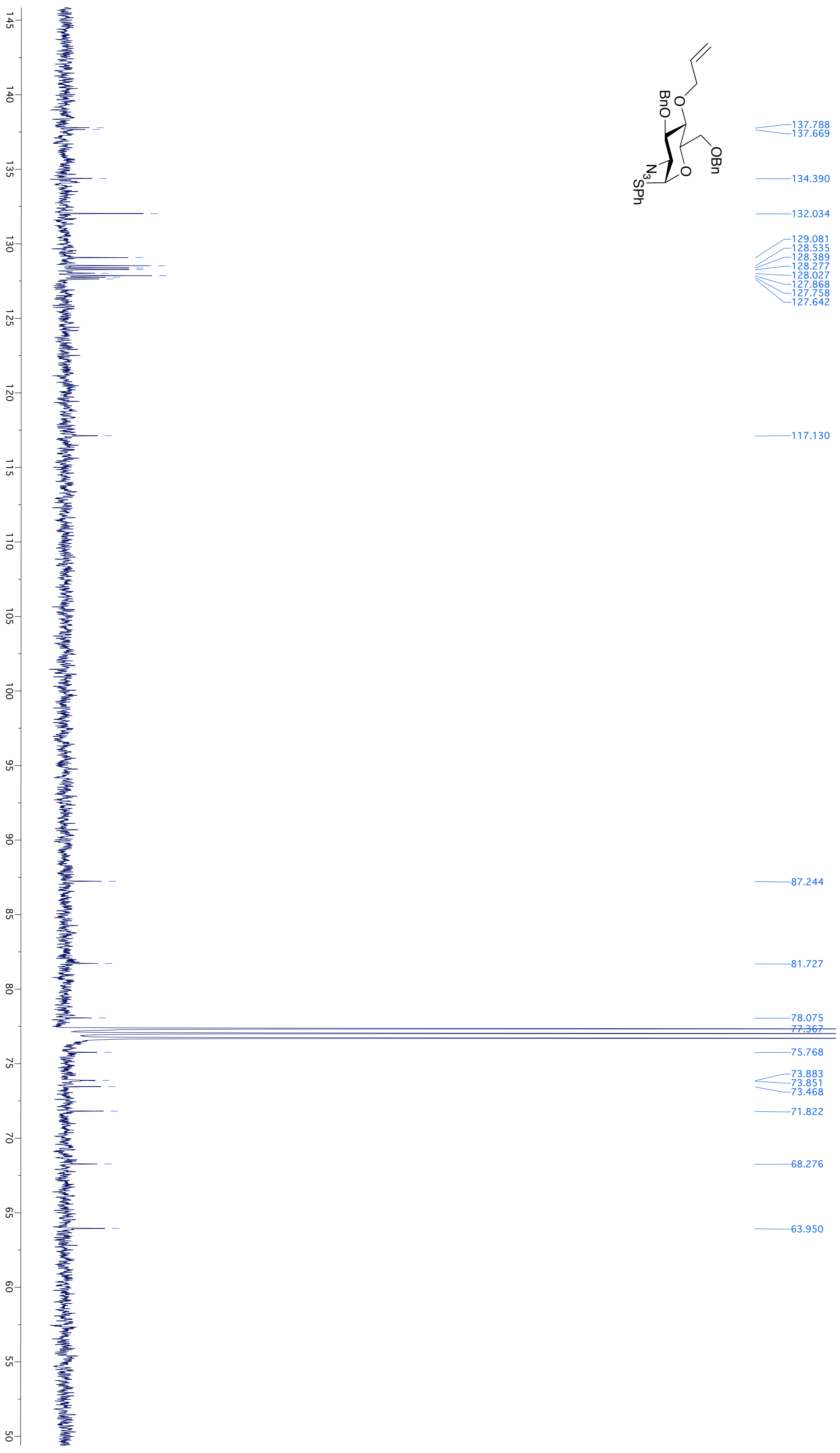

100 MHz, $CDCl_3$

Phenyl-4-O-allyl-2-azido-2-deoxy-3,6-di-O-benzyl-1-thio-α-D-glucopyranoside (10)

COSY

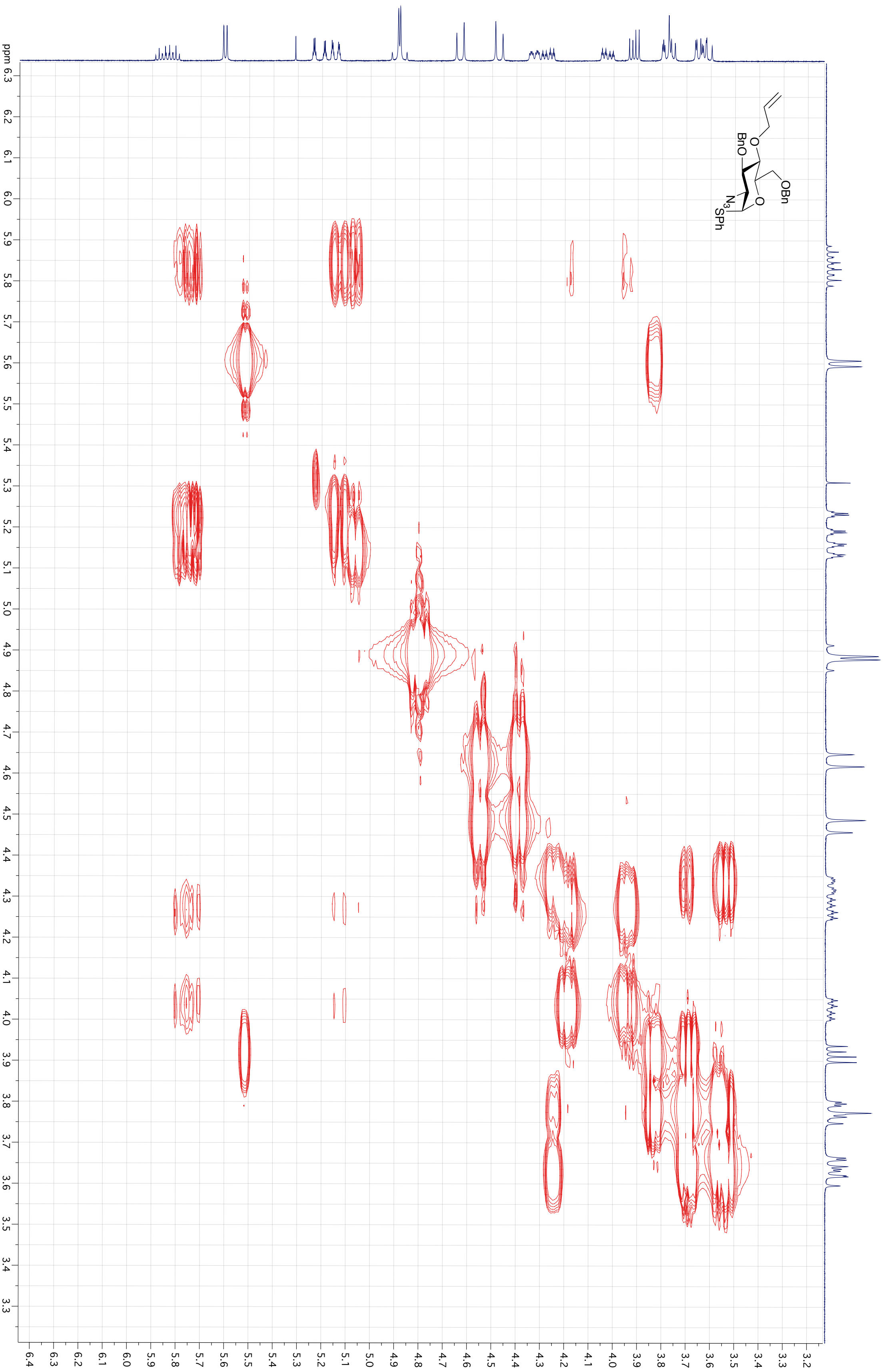

400 MHz, $CDCl_3$

Phenyl-2-azido-2-deoxy-3,6-di-O-benzyl-4-O-[(*S*/*R*)-2,3-dihydroxypropoxy]-1-thio-α-D-glucopyranoside (11)

^{1}H NMR

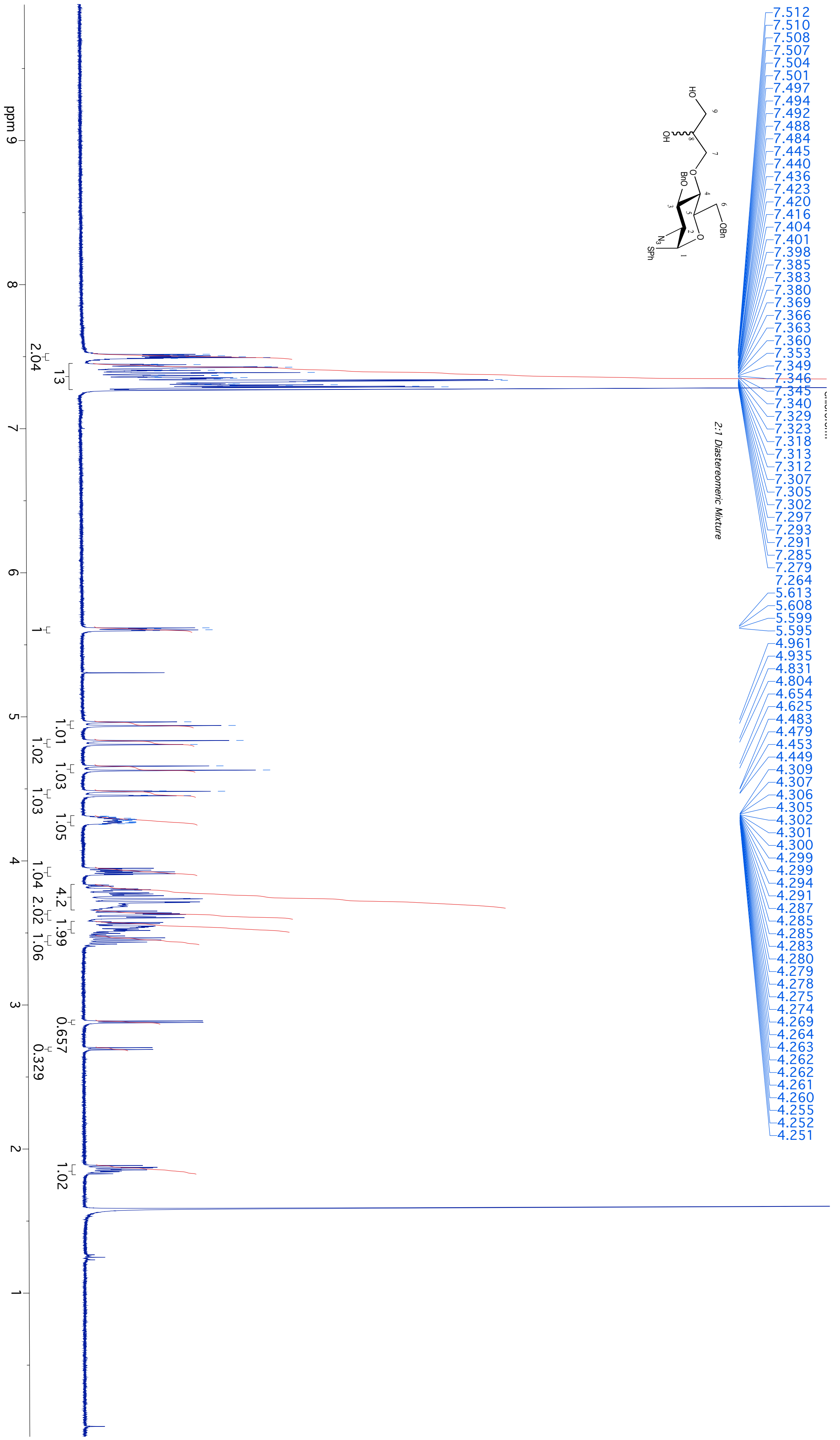

400 MHz, $CDCl_3$

Phenyl-2-azido-2-deoxy-3,6-di-O-benzyl-4-O-[(*S*/*R*)-2,3-dihydroxypropoxy]-1-thio-α-D-glucopyranoside (11)

^{13}C NMR

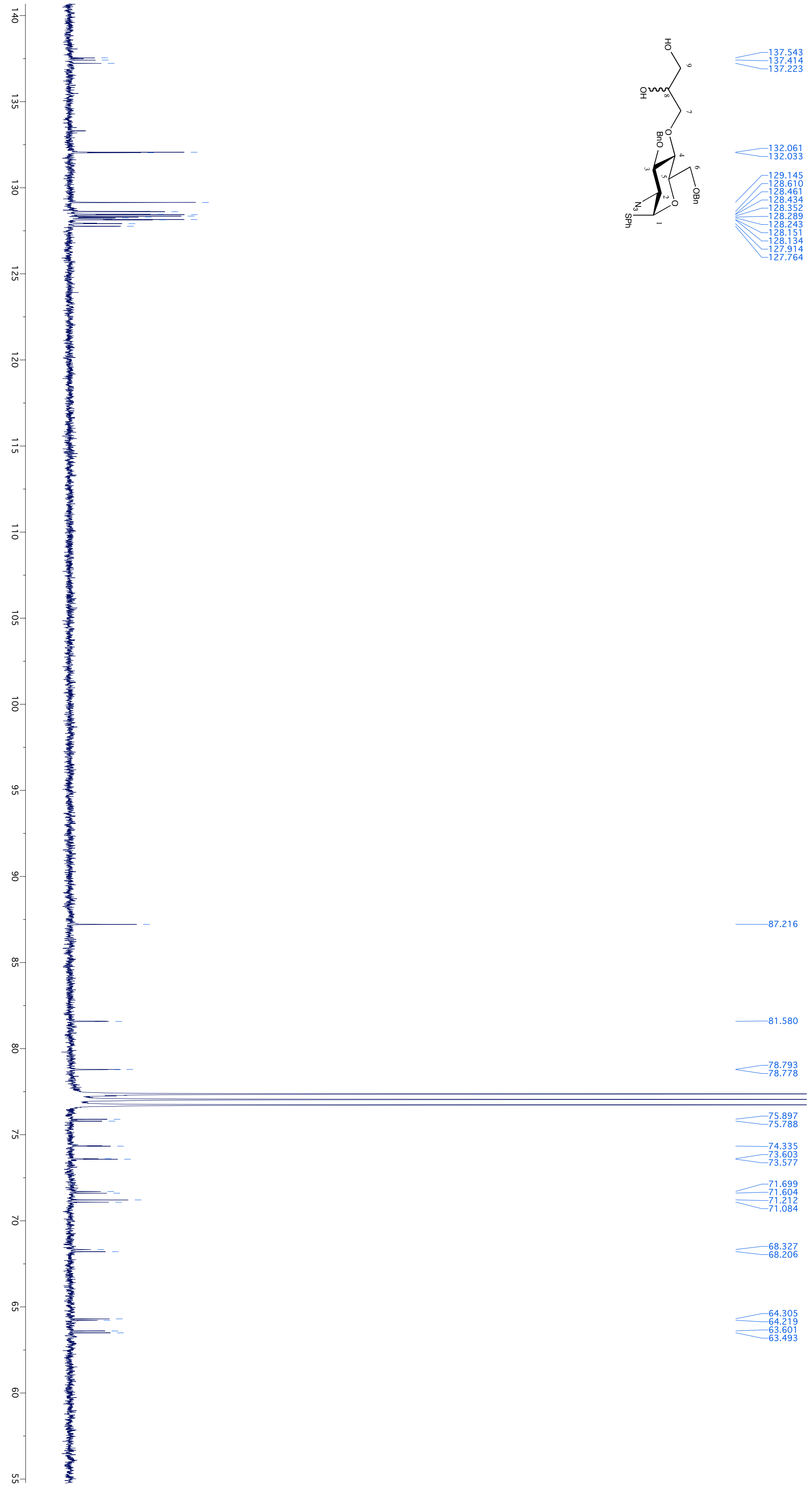

100 MHz, $CDCl_3$

Phenyl-2-azido-2-deoxy-3,6-di-O-benzyl-4-O-[(*S*/*R*)-2,3-dihydroxypropoxy]-1-thio-α-D-glucopyranoside (11)

HMQC

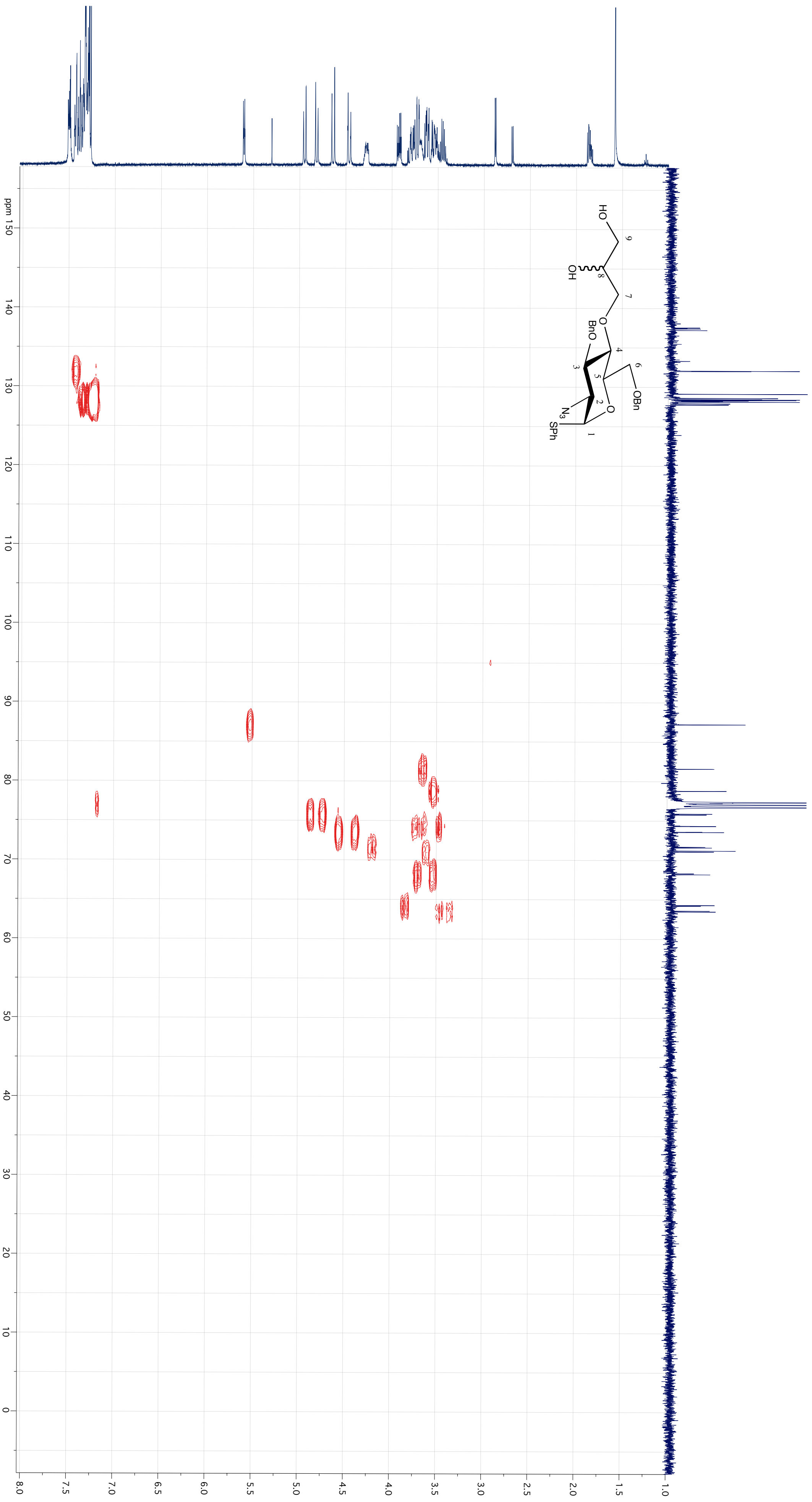

400 MHz, $CDCl_3$

Phenyl-2-azido-2-deoxy-3,6-di-O-benzyl-4-O-[(*S/R*)-2,3-bis(benzyloxy)propoxy]-1-thio-α-D-glucopyranoside (12)

^{1}H NMR

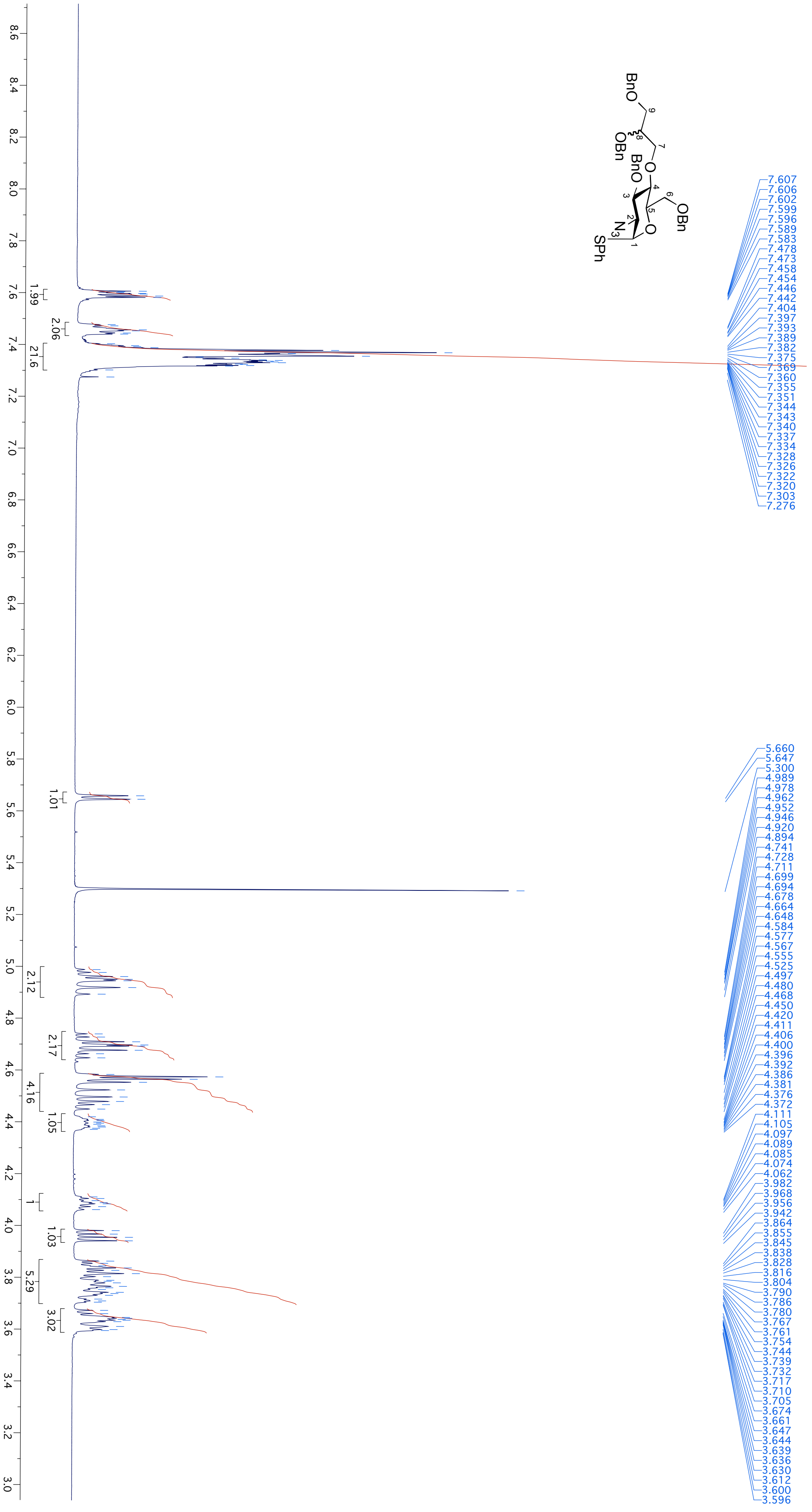

400 MHz, $CDCl_3$

Phenyl-2-azido-2-deoxy-3,6-di-O-benzyl-4-O-[(*S/R*)-2,3-bis(benzyloxy)propoxy]-1-thio-α-D-glucopyranoside (12)

^{13}C NMR

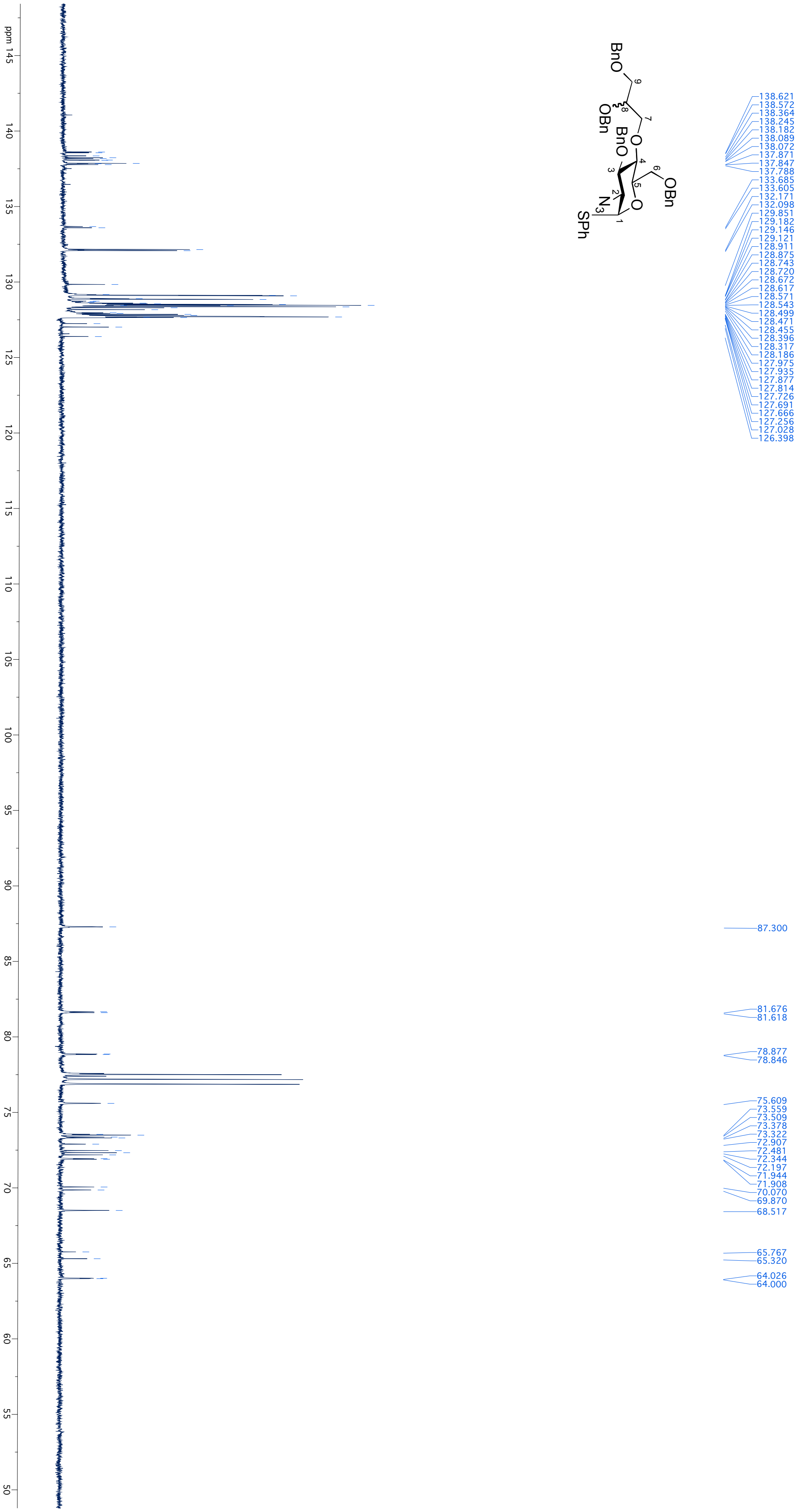

100 MHz, $CDCl_3$

Phenyl-2-azido-2-deoxy-3,6-di-O-benzyl-4-O-[(*S*)-2,3-bis(benzyloxy)propoxy]-1-thio-α-D-glucopyranoside (15)

^{1}H NMR

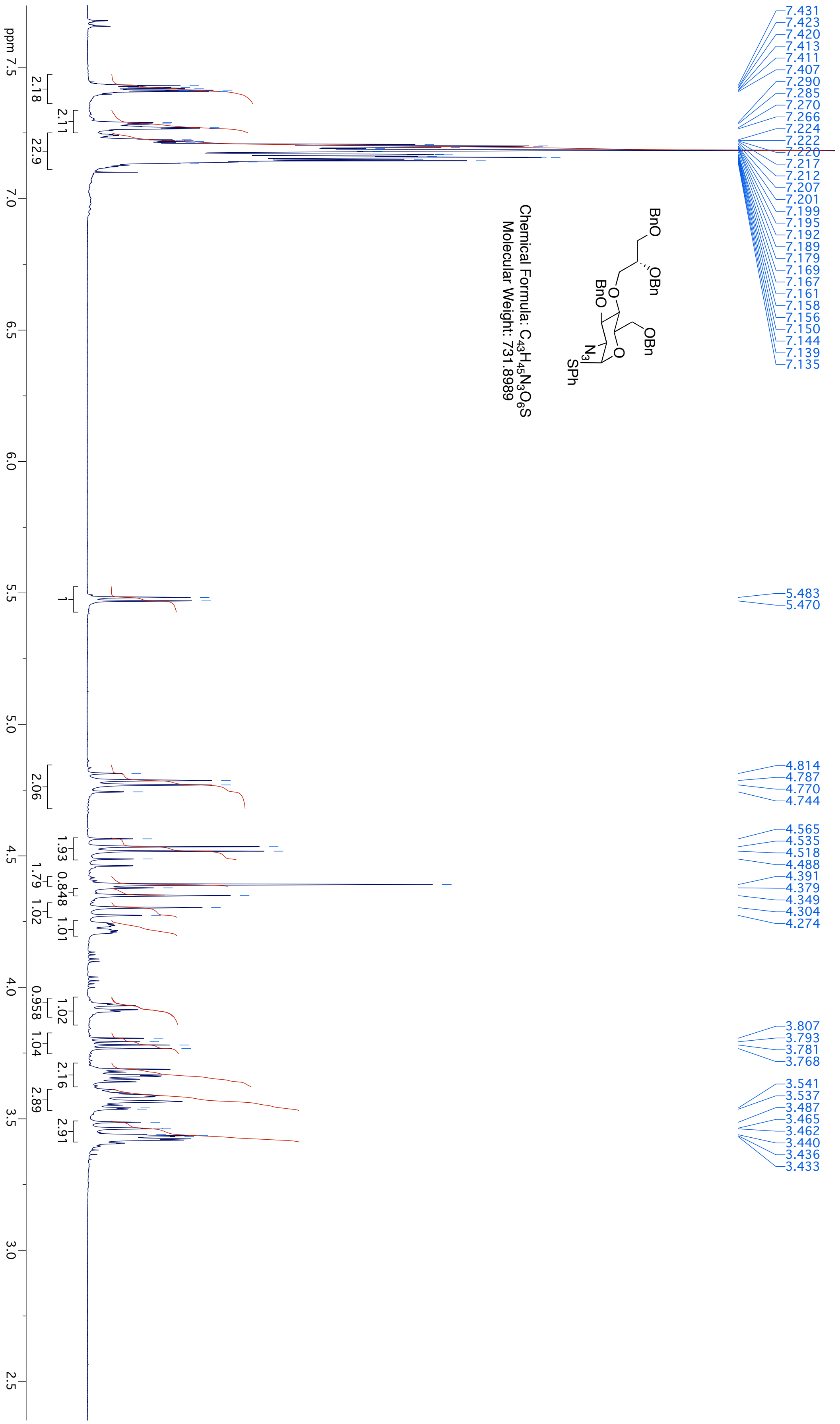

400 MHz, $CDCl_3$

Phenyl-2-azido-2-deoxy-3,6-di-O-benzyl-4-O-[(*S*)-2,3-bis(benzyloxy)propoxy]-1-thio-α-D-glucopyranoside (15)

^{13}C NMR

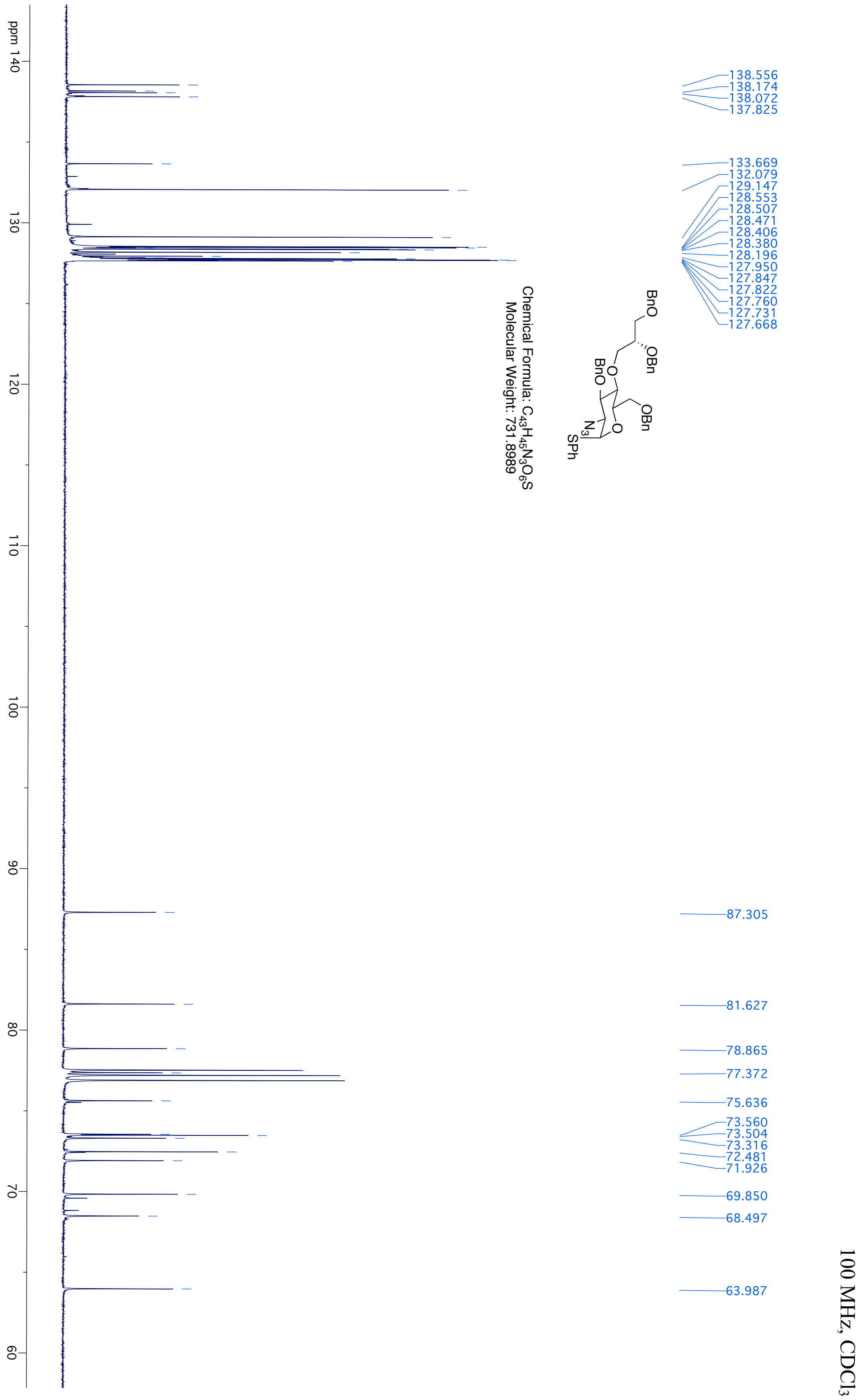

Phenyl-2-azido-2-deoxy-3,6-di-O-benzyl-4-O-[(*S*)-2,3-bis(benzyloxy)propoxy]-1-thio-α-D-glucopyranoside (15)

COSY

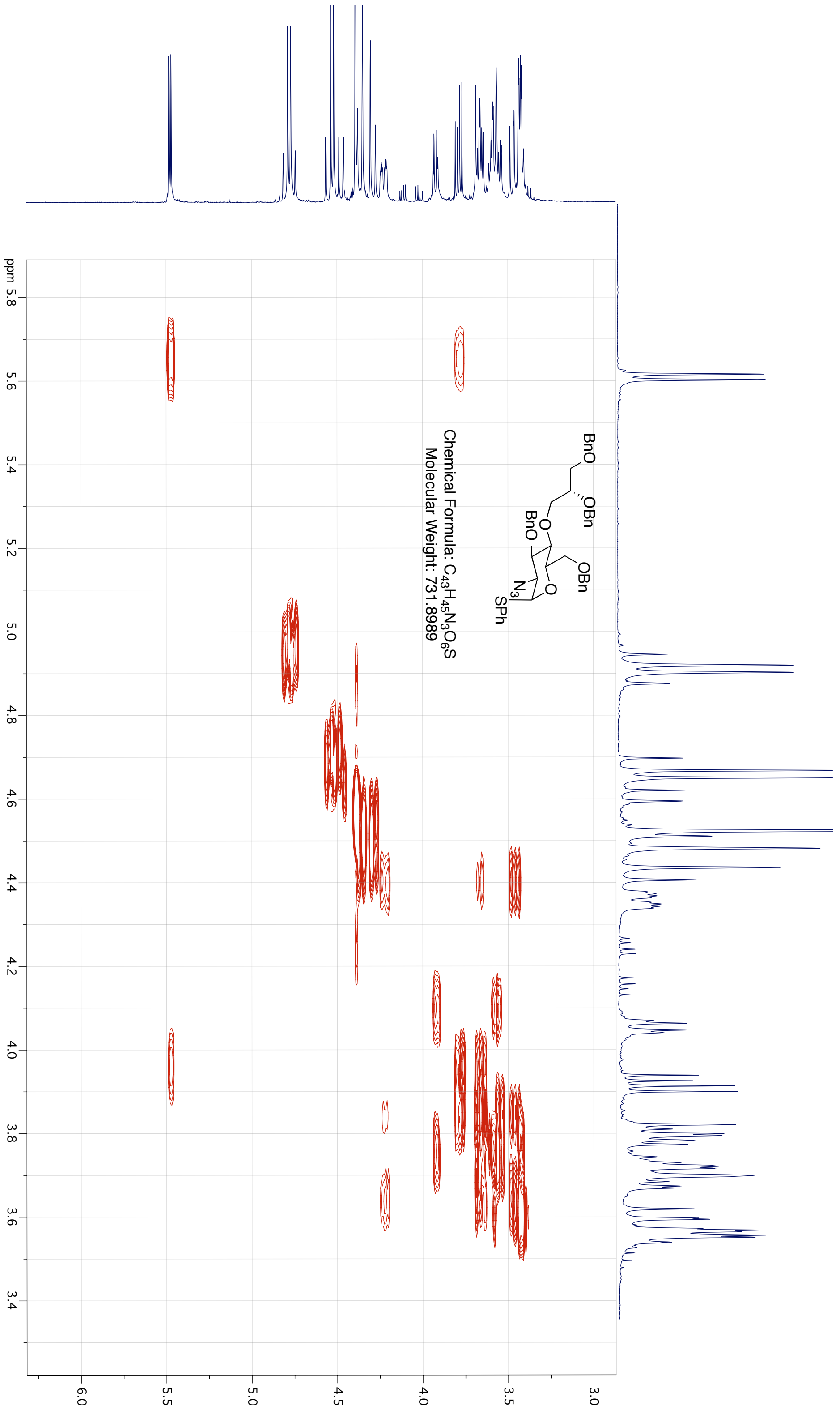

400 MHz, $CDCl_3$

Phenyl-2-azido-2-deoxy-3,6-di-O-benzyl-4-O-[(*S*)-2,3-bis(benzyloxy)propoxy]-1-thio-β-D-glucopyranoside (17)

^{1}H NMR

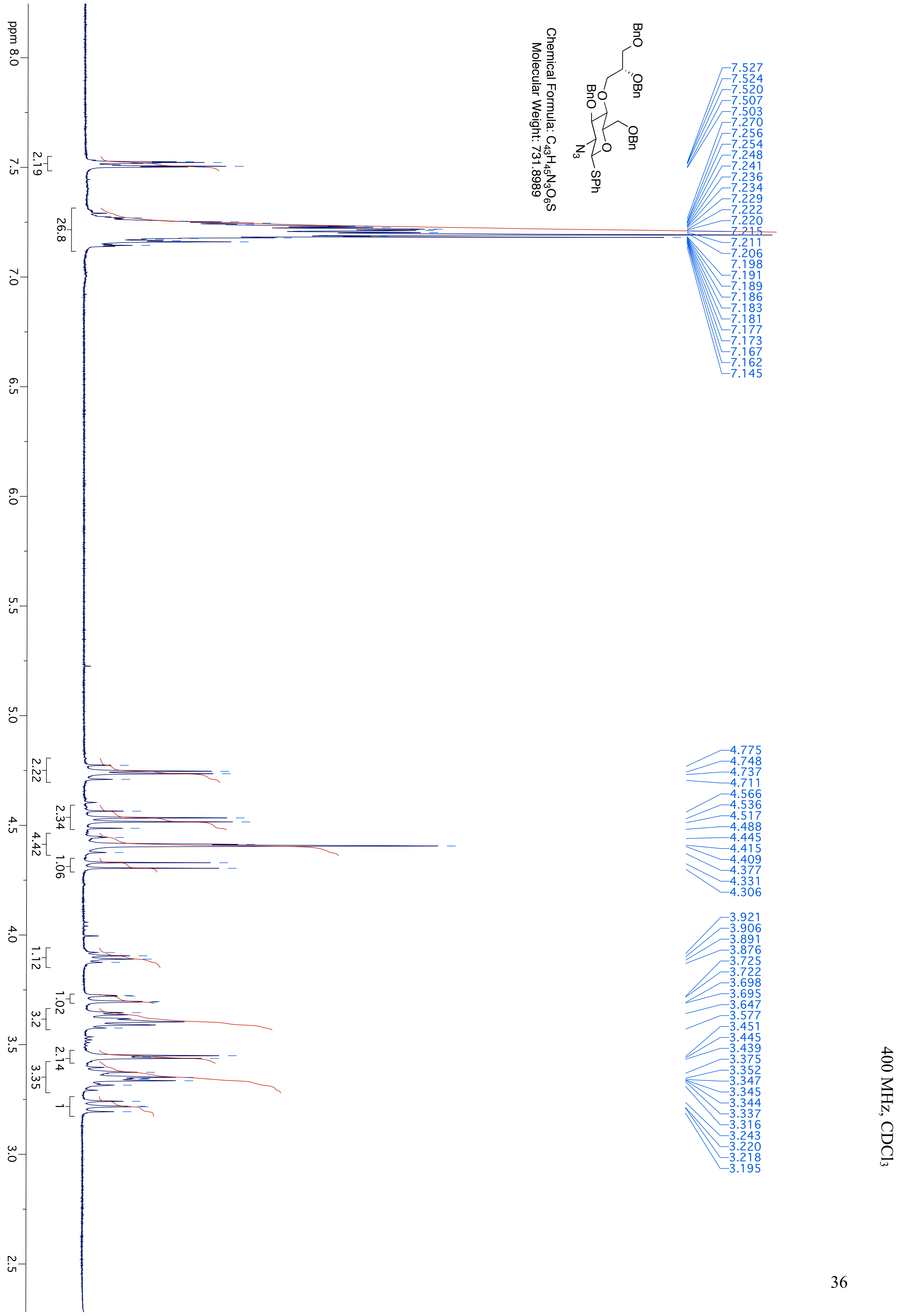

Phenyl-2-azido-2-deoxy-3,6-di-O-benzyl-4-O-[(*S*)-2,3-bis(benzyloxy)propoxy]-1-thio-β-D-glucopyranoside (17)

^{13}C NMR

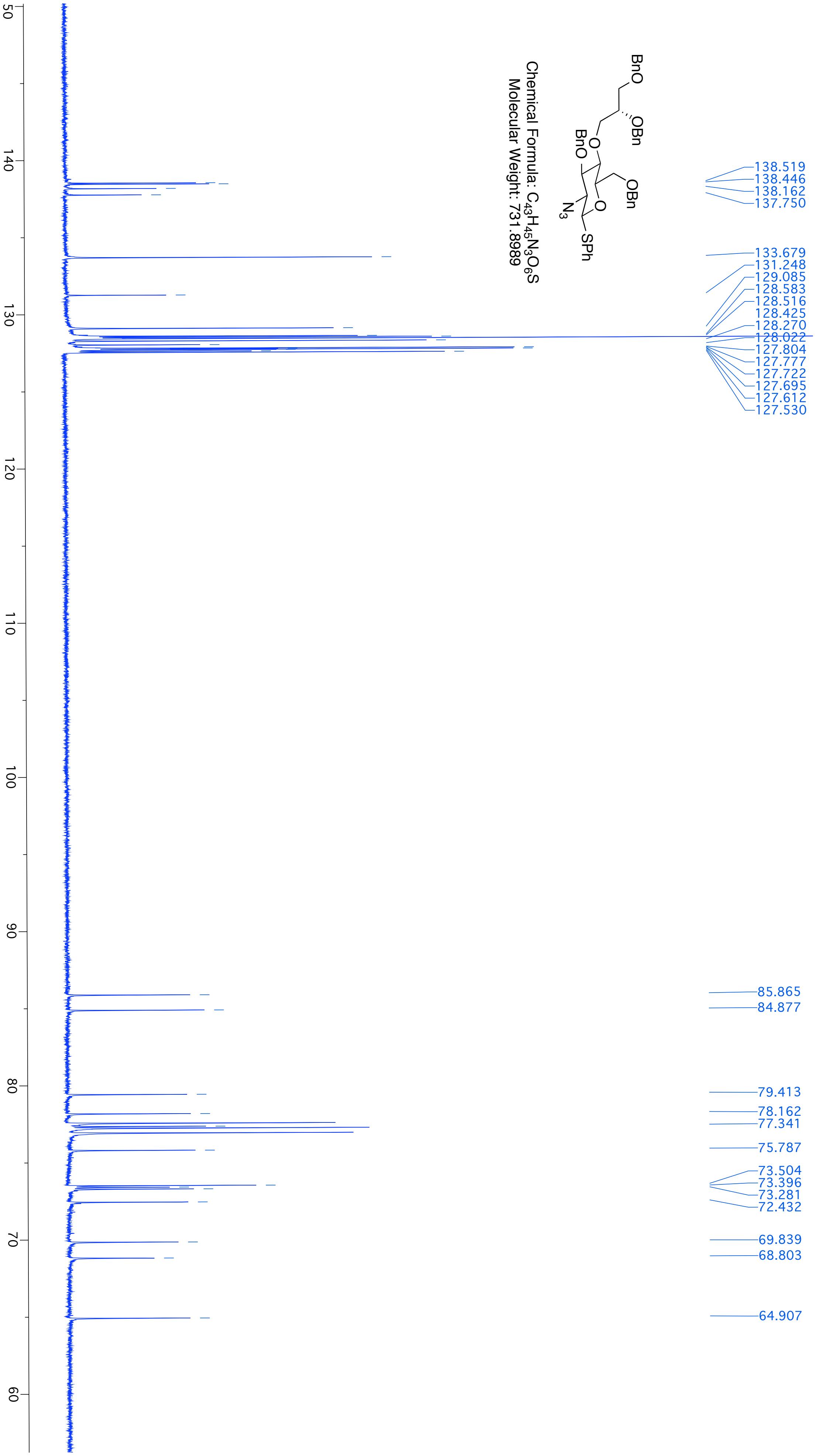

100 MHz, $CDCl_3$

Phenyl-2-azido-2-deoxy-3,6-di-O-benzyl-4-O-[(*S*)-2,3-bis(benzyloxy)propoxy]-1-thio-β-D-glucopyranoside (17)

COSY

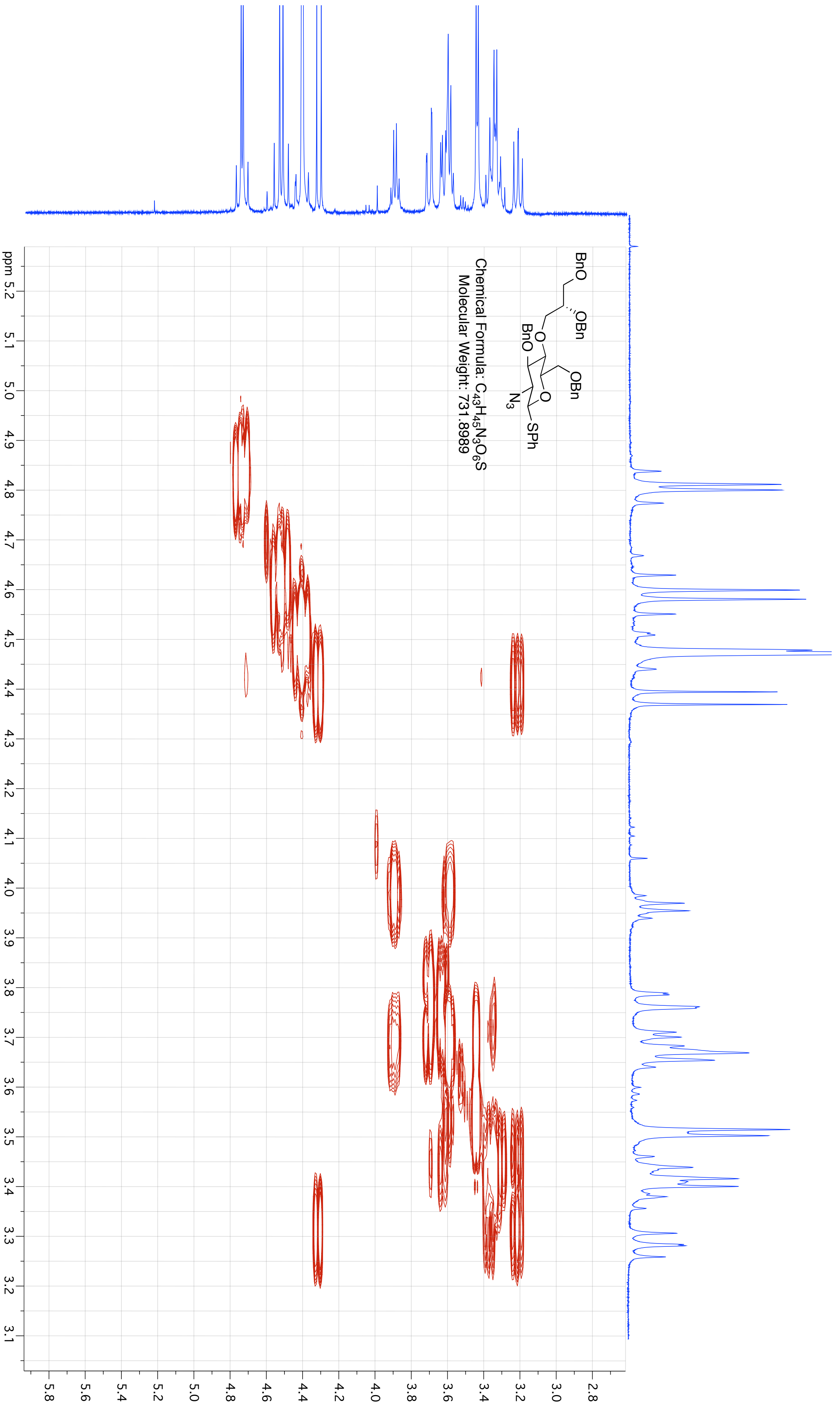

400 MHz, $CDCl_3$

Phenyl-2-azido-2-deoxy-3,6-di-O-benzyl-4-O-[(*S*)-2,3-bis(benzyloxy)propoxy]-1-thio-β-D-glucopyranoside (17)

HMQC

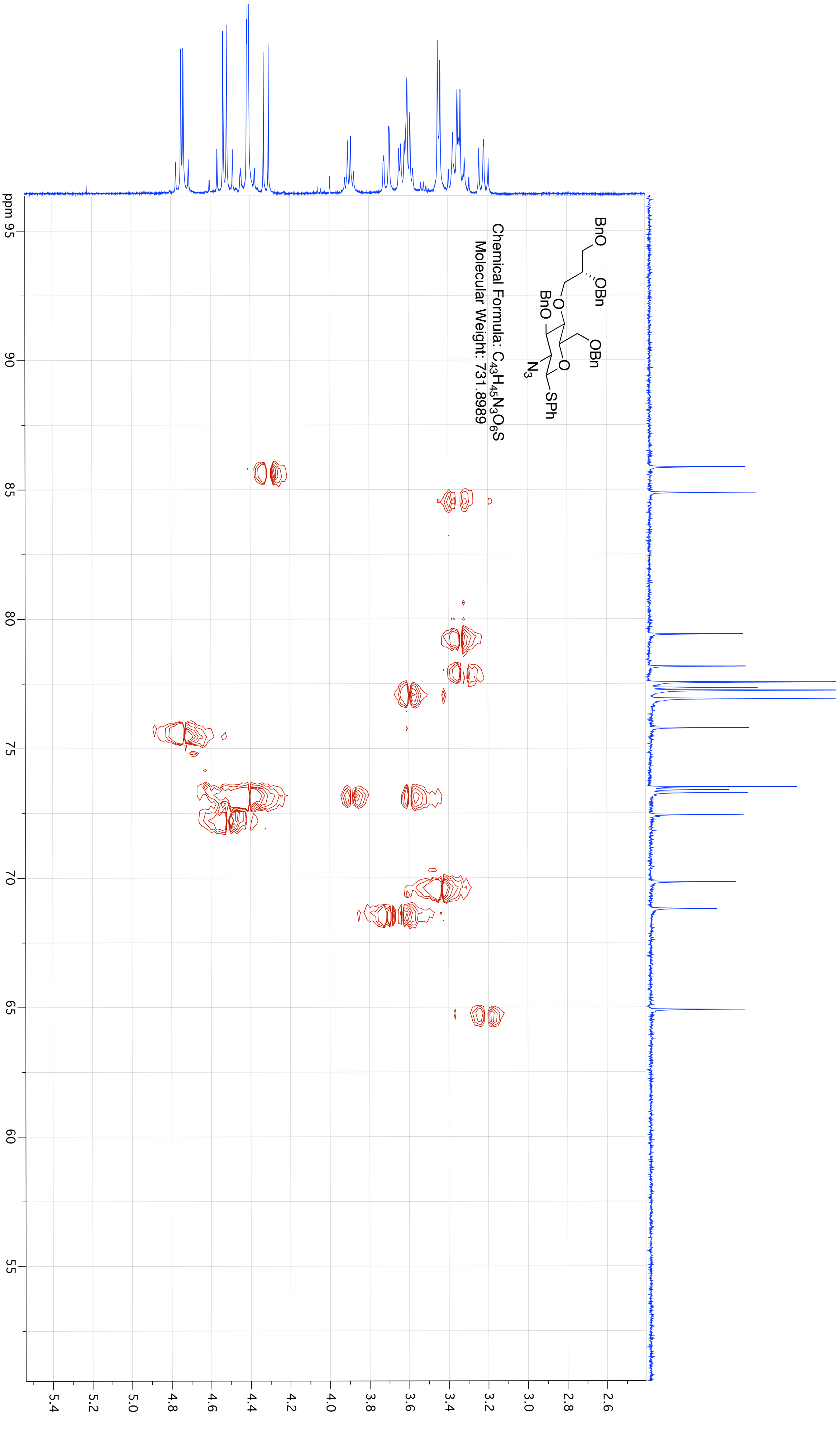

400 MHz, $CDCl_3$

Phenyl-2-azido-2-deoxy-3-O-benzyl-4-O-[(*S*)-2,3-bis(benzyloxy)propoxy]-6-O-benzoyl-1-thio-β-D-glucopyranoside (18)

^{1}H NMR

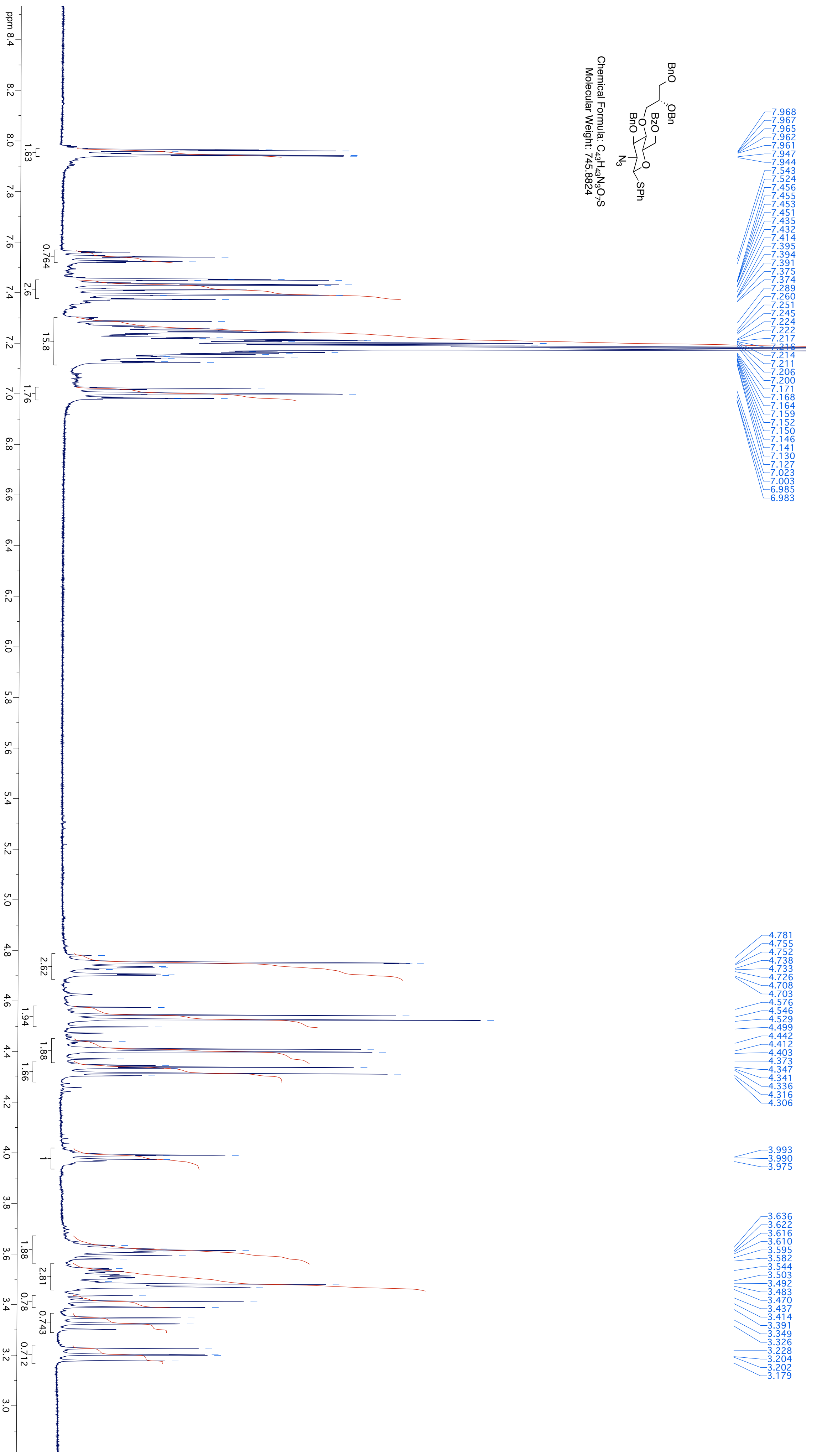

400 MHz, $CDCl_3$

Phenyl-2-azido-2-deoxy-3-O-benzyl-4-O-[(*S*)-2,3-bis(benzyloxy)propoxy]-6-O-benzoyl-1-thio-β-D-glucopyranoside (18)

^{13}C NMR

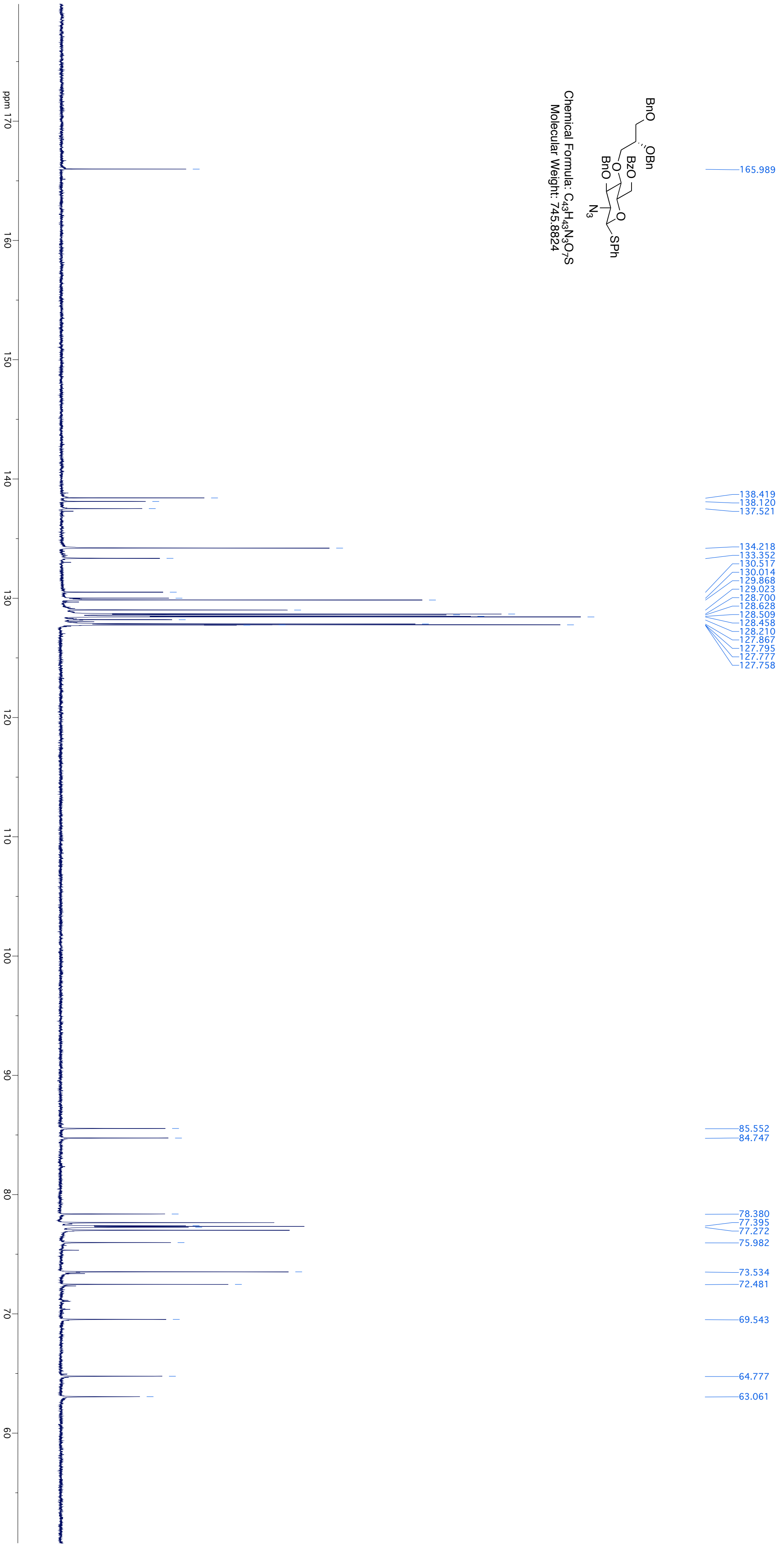

100 MHz, CDCl$_3$

Phenyl-2-azido-2-deoxy-3-O-benzyl-4-O-[(*S*)-2,3-bis(benzyloxy)propoxy]-6-O-benzoyl-1-thio-β-D-glucopyranoside (18)

COSY

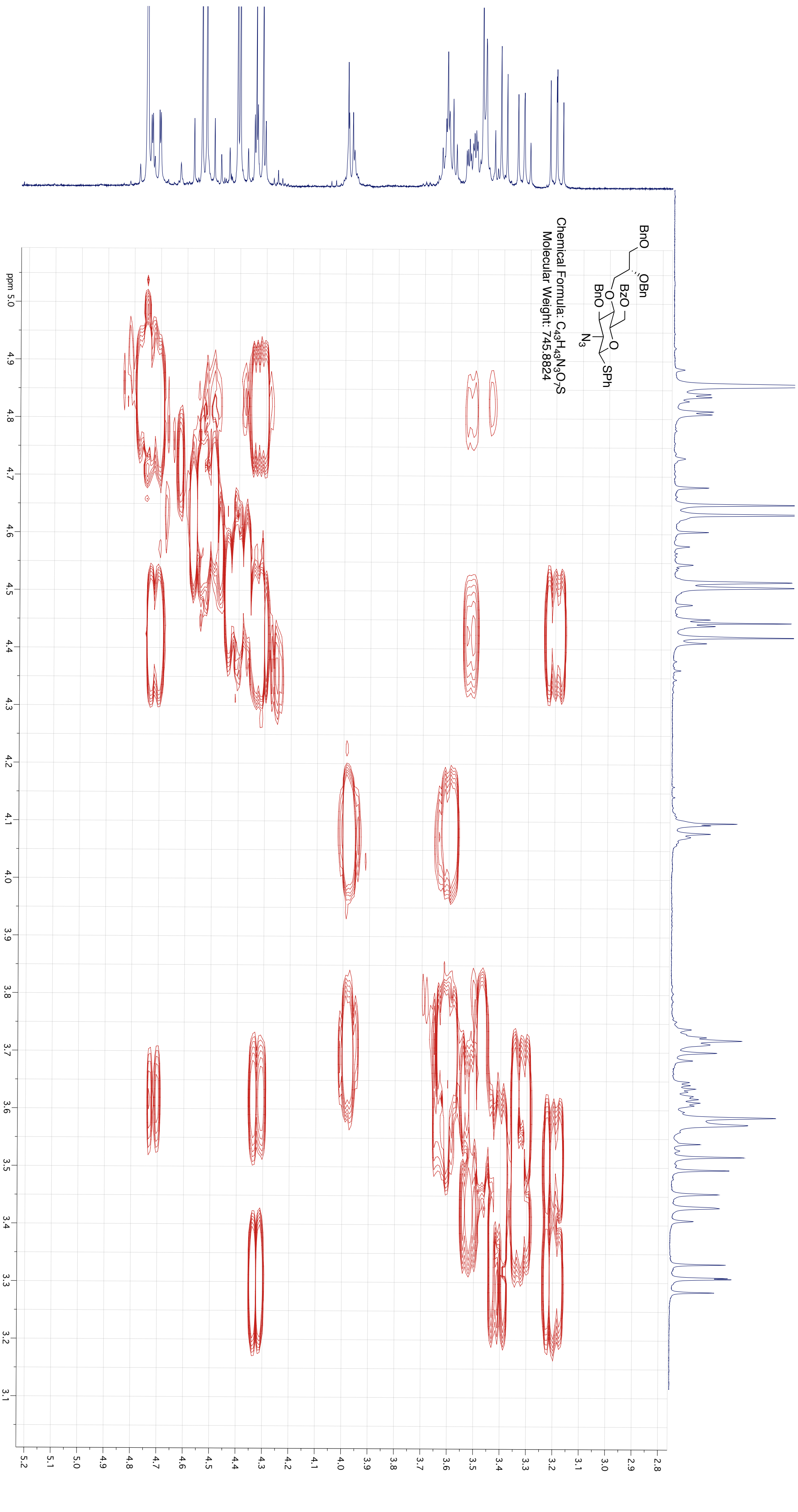

400 MHz, $CDCl_3$

Phenyl-2-azido-2-deoxy-3-O-benzyl-4-O-[(*S*)-2,3-bis(benzyloxy)propoxy]-6-O-benzoyl-1-thio-β-D-glucopyranoside (18)

HMQC

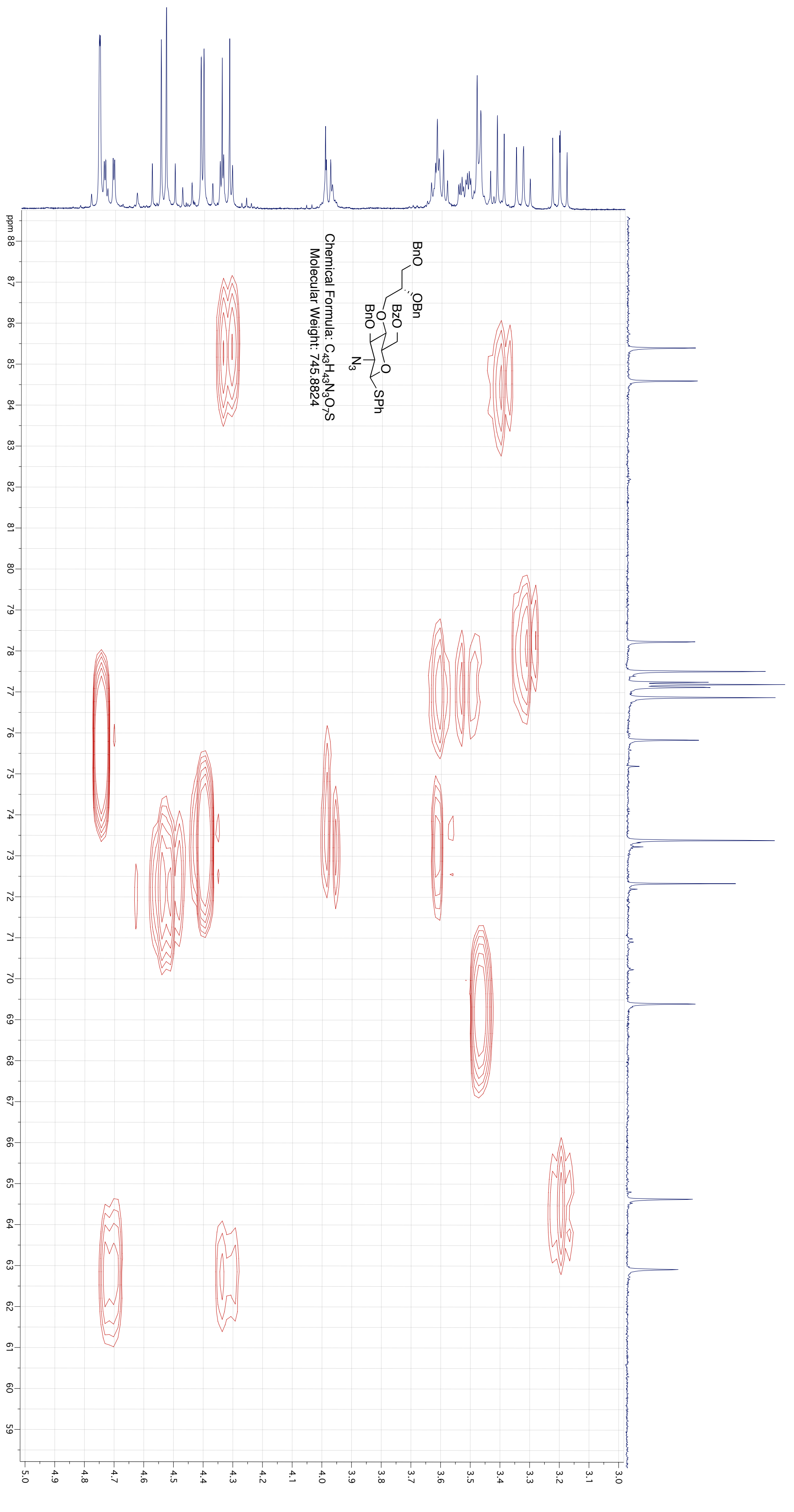

400 MHz, CDCl$_3$

2-Azido-3,6-di-O-benzyl-2-deoxy-4-O-[(*S*)-2,3-bis(benzyloxy)propoxy]-αβ-D-glucopyranose

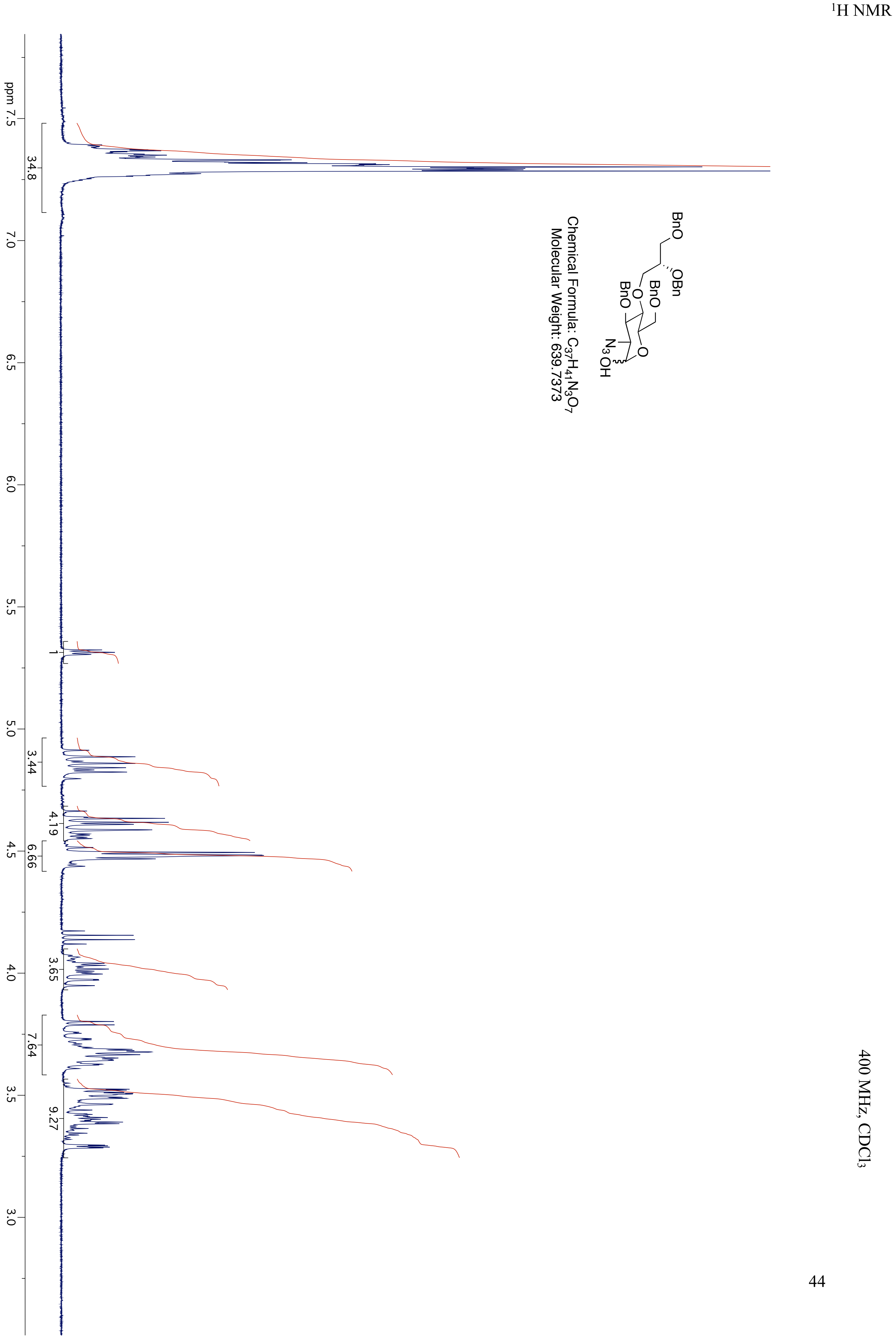

2-Azido-3,6-di-O-benzyl-2-deoxy-4-O-[(*S*)-2,3-bis(benzyloxy)propoxy]-αβ-D-glucopyranose

COSY

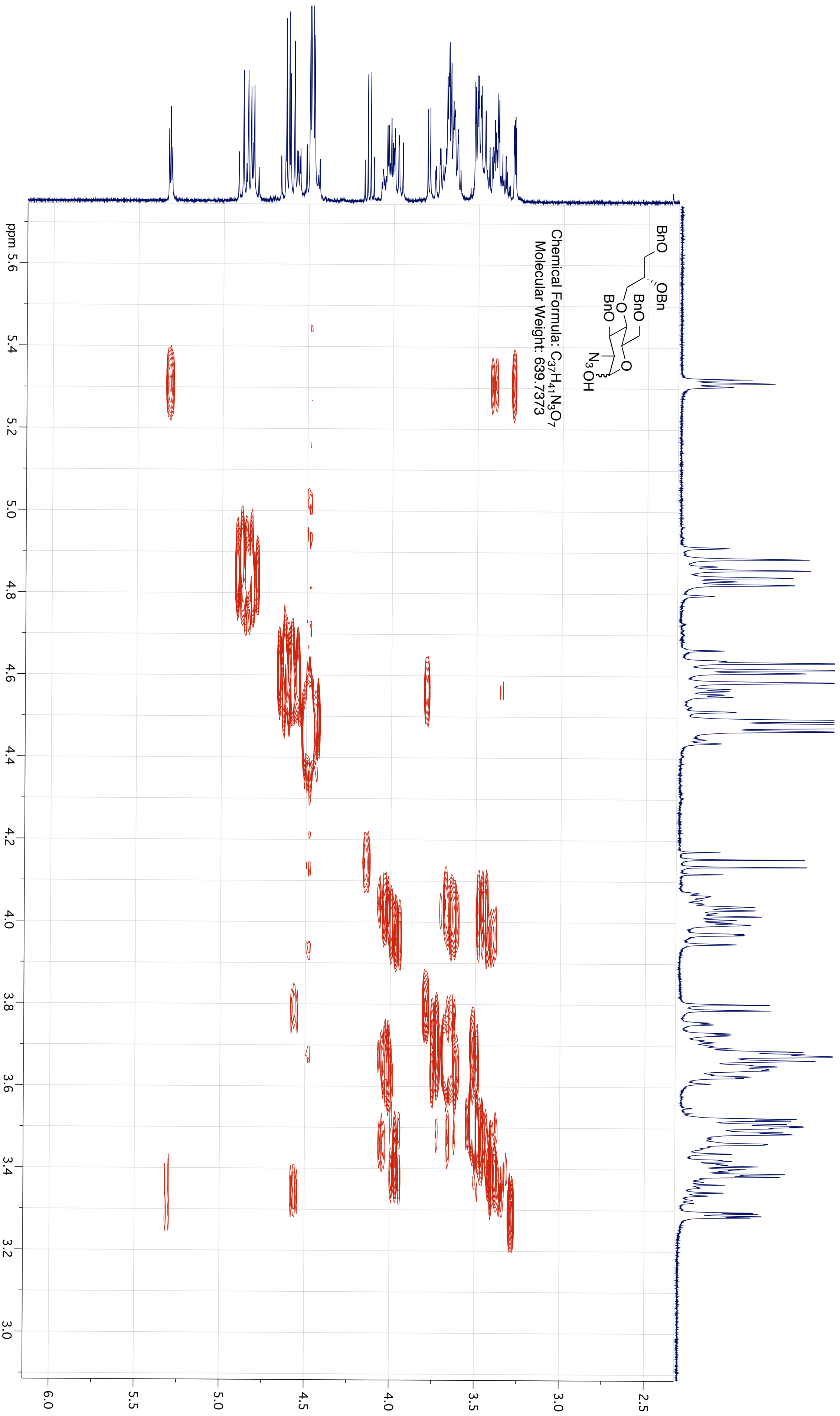

400 MHz, $CDCl_3$

2-Azido-3,6-di-O-benzyl-2-deoxy-4-O-[(*S*)-2,3-bis(benzyloxy)propoxy]-1-O-trichloroacetimidate-αβ-D-glucopyranoside

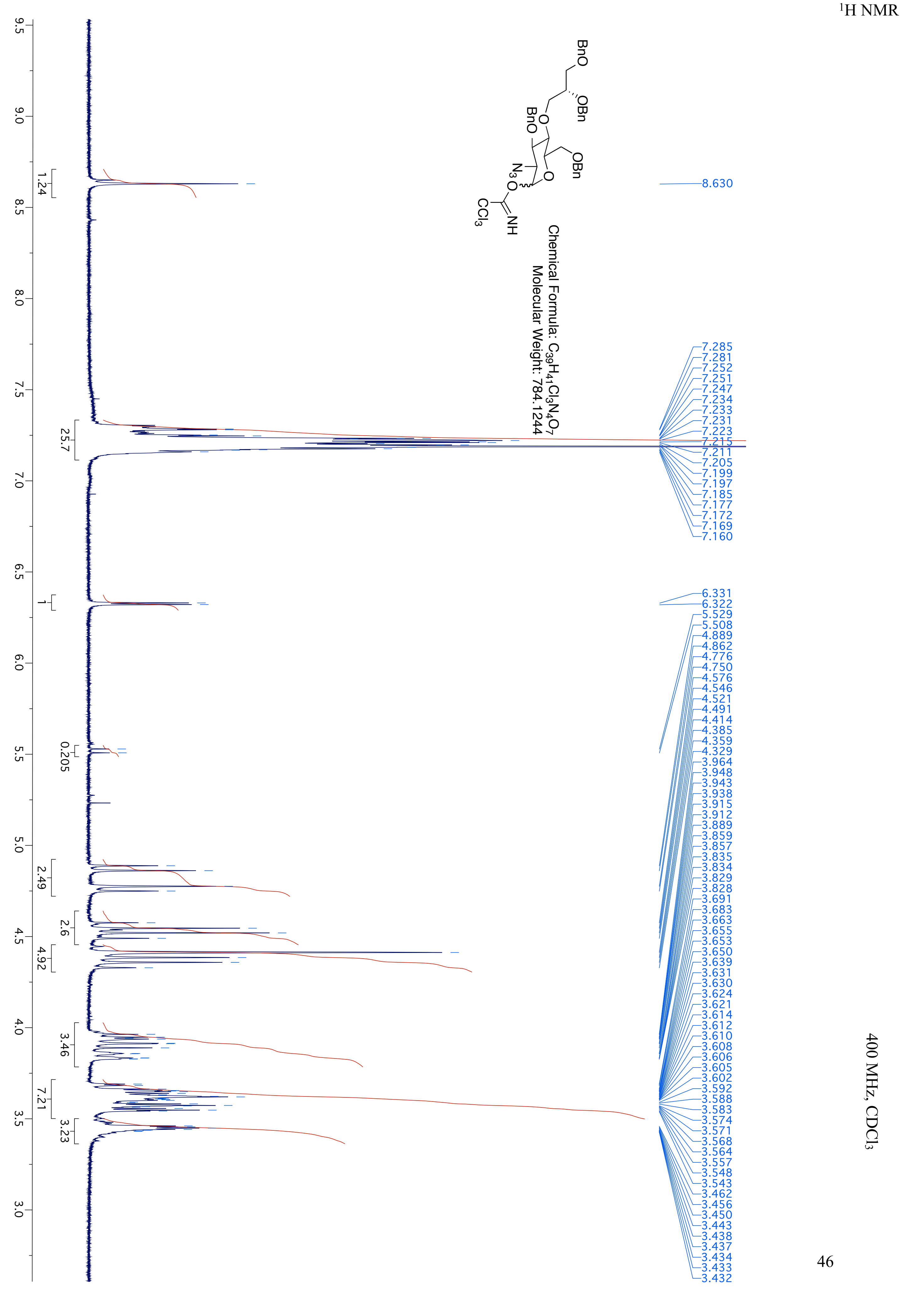

Methyl (phenyl 4-O-(2-azido-3,6-di-O-benzyl-2-deoxy-4-O-[(*S*)-2,3-bis(benzyloxy)propoxy]-α-D-glucopyranosyl)-2-O-benzoyl-3-O-benzyl-1-thio-α-L-idopyranoside)-uronate (20)

^{1}H NMR

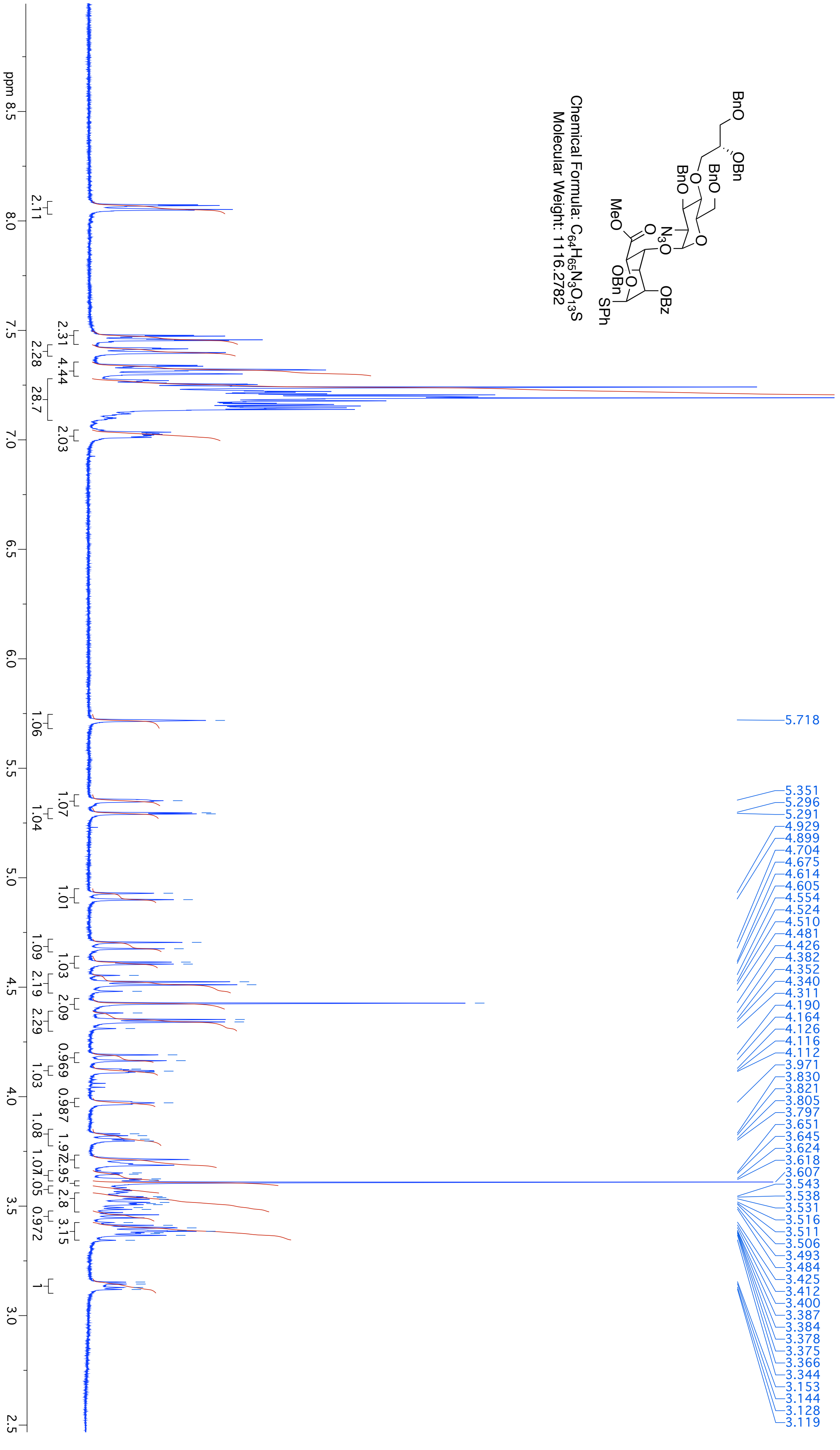

400 MHz, CDCl$_3$

Methyl (phenyl 4-O-(2-azido-3,6-di-O-benzyl-2-deoxy-4-O-[(*S*)-2,3-bis(benzyloxy)propoxy]-α-D-glucopyranosyl)-2-O-benzoyl-3-O-benzyl-1-thio-α-L-idopyranoside)-uronate (20)

^{13}C NMR

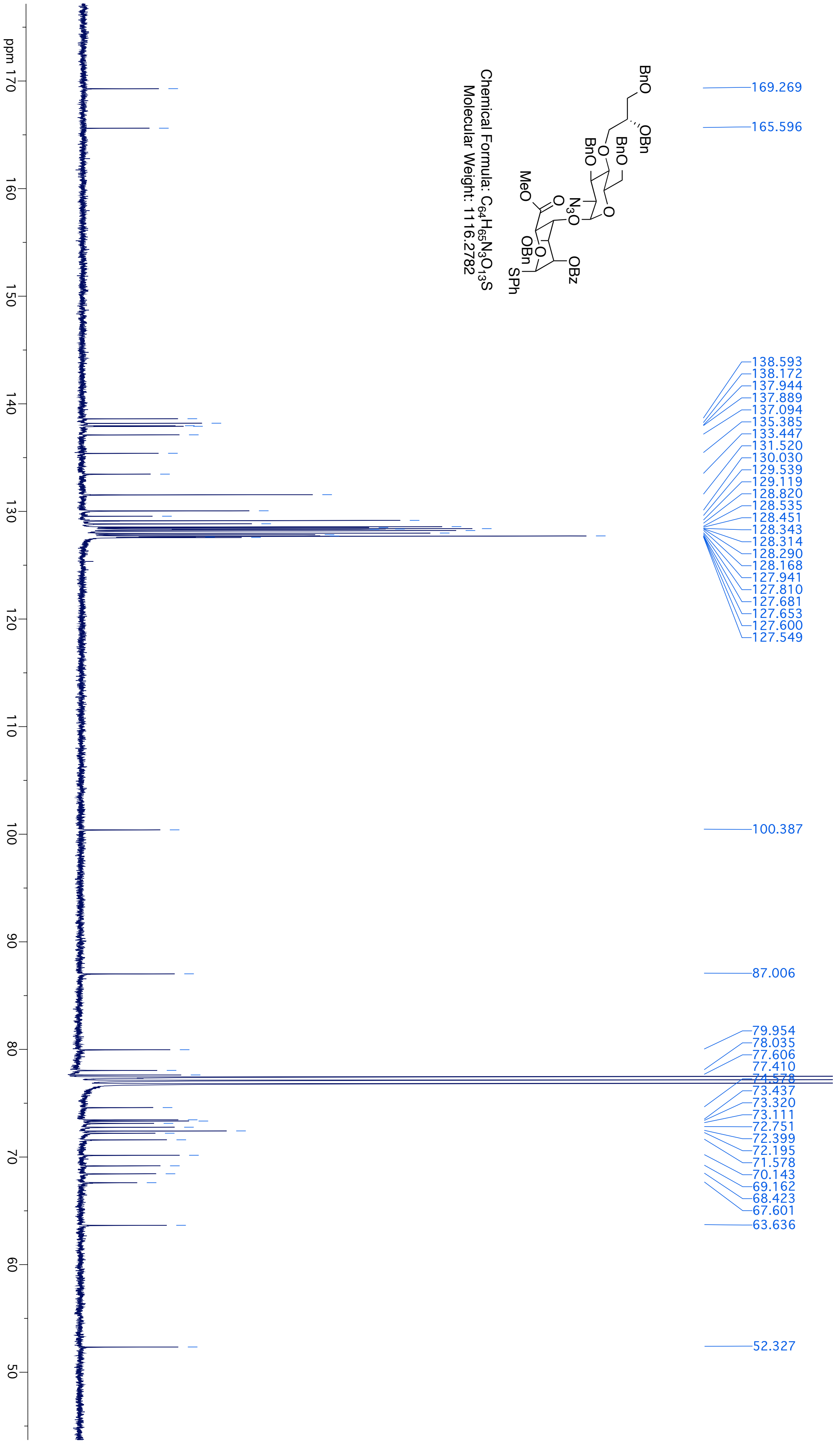

100 MHz, $CDCl_3$

Methyl (phenyl 4-O-(2-azido-3,6-di-O-benzyl-2-deoxy-4-O-[(*S*)-2,3-bis(benzyloxy)propoxy]-α-D-glucopyranosyl)-2-O-benzoyl-3-O-benzyl-1-thio-α-L-idopyranoside)-uronate (20)

COSY

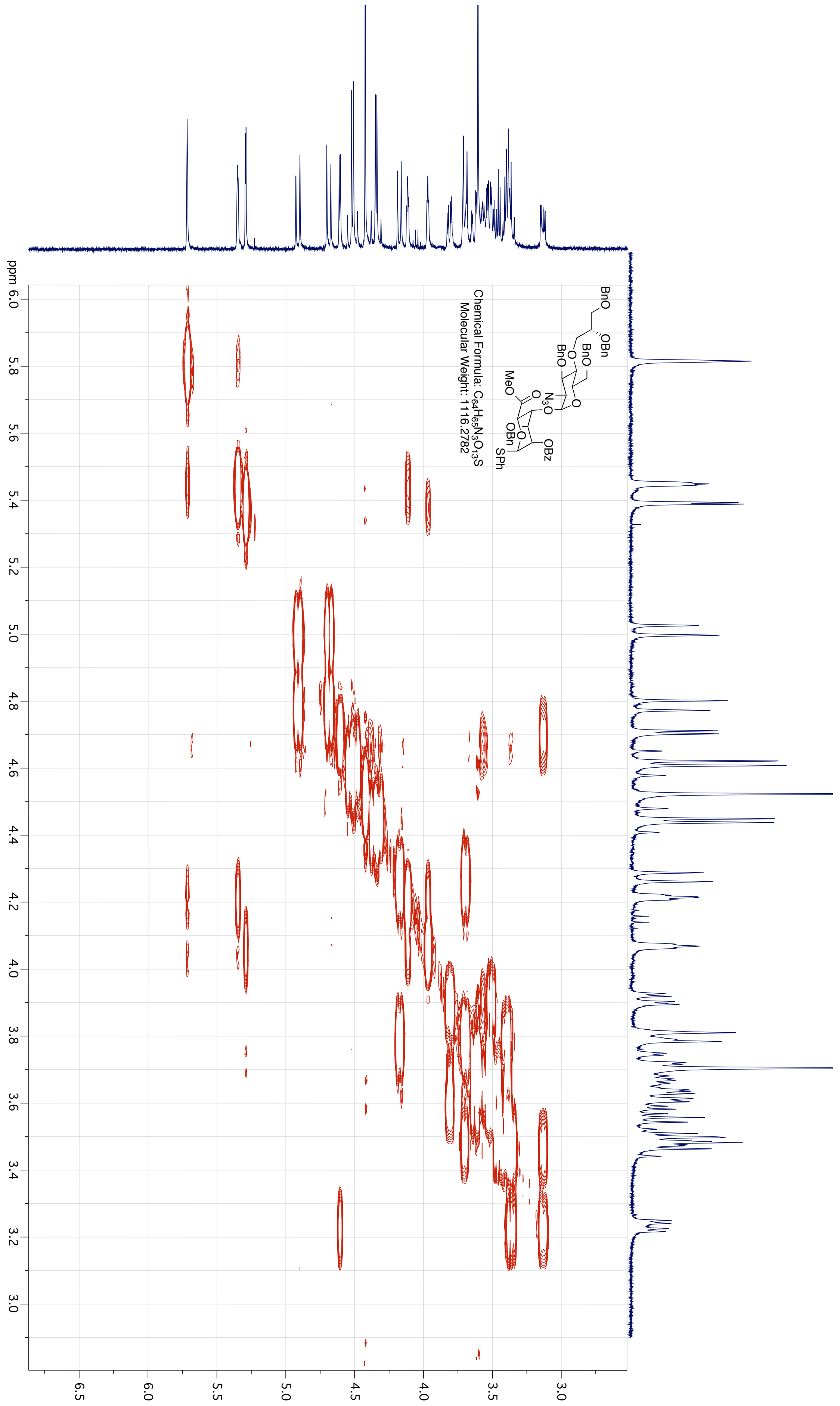

400 MHz, $CDCl_3$

Methyl (phenyl 4-O-(2-azido-3,6-di-O-benzyl-2-deoxy-4-O-[(*S*)-2,3-bis(benzyloxy)propoxy]-α-D-glucopyranosyl)-2-O-benzoyl-3-O-benzyl-1-thio-α-L-idopyranoside)-uronate (20)

HMQC

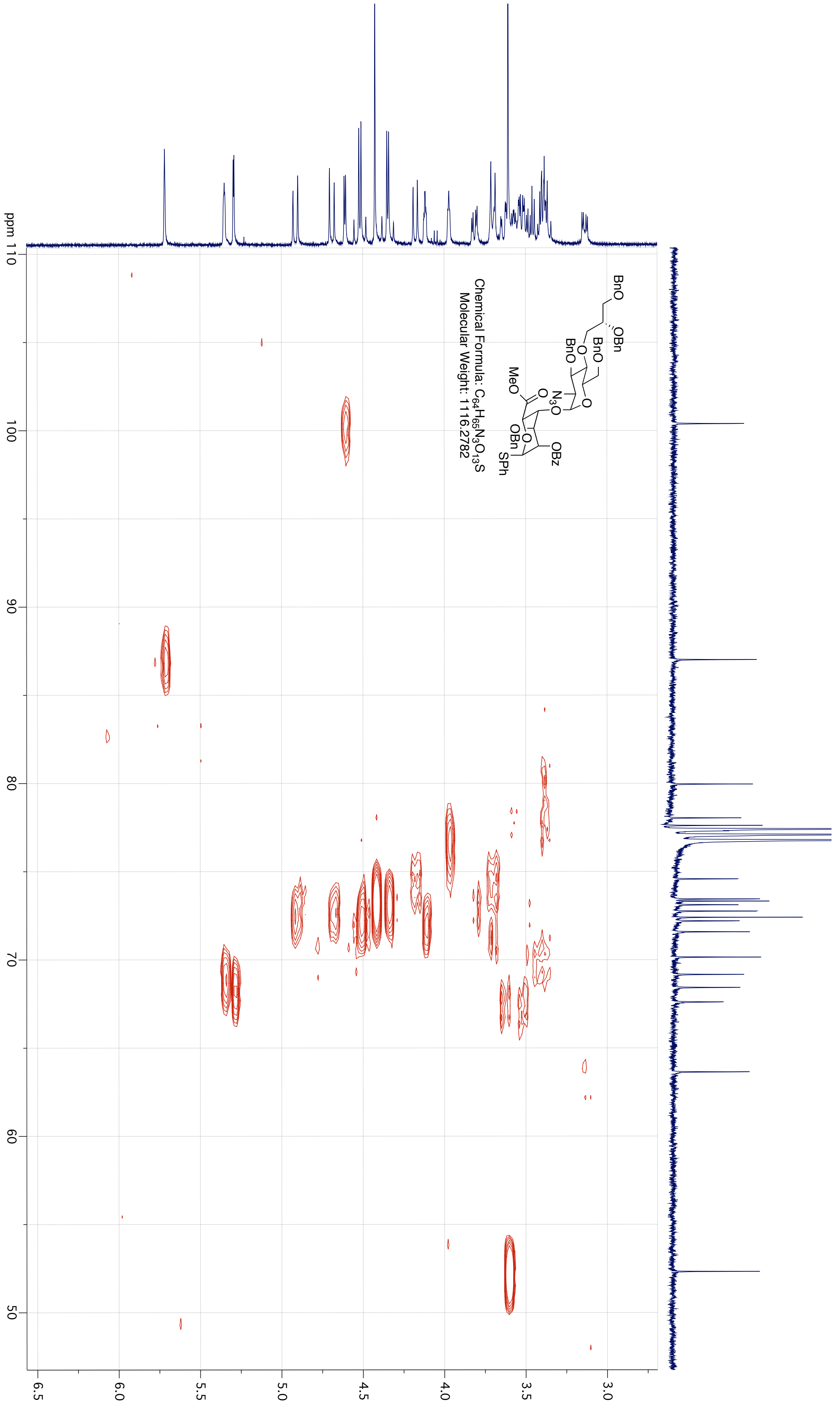

400 MHz, $CDCl_3$

Methyl (phenyl 4-O-(2-azido-3,6-di-O-benzyl-2-deoxy-4-O-[(*S*)-2,3-bis(benzyloxy)propoxy]-α-D-glucopyranosyl)-2-O-benzoyl-3-O-benzyl-1-thio-α-L-idopyranoside)-uronate (20)

ESI MS

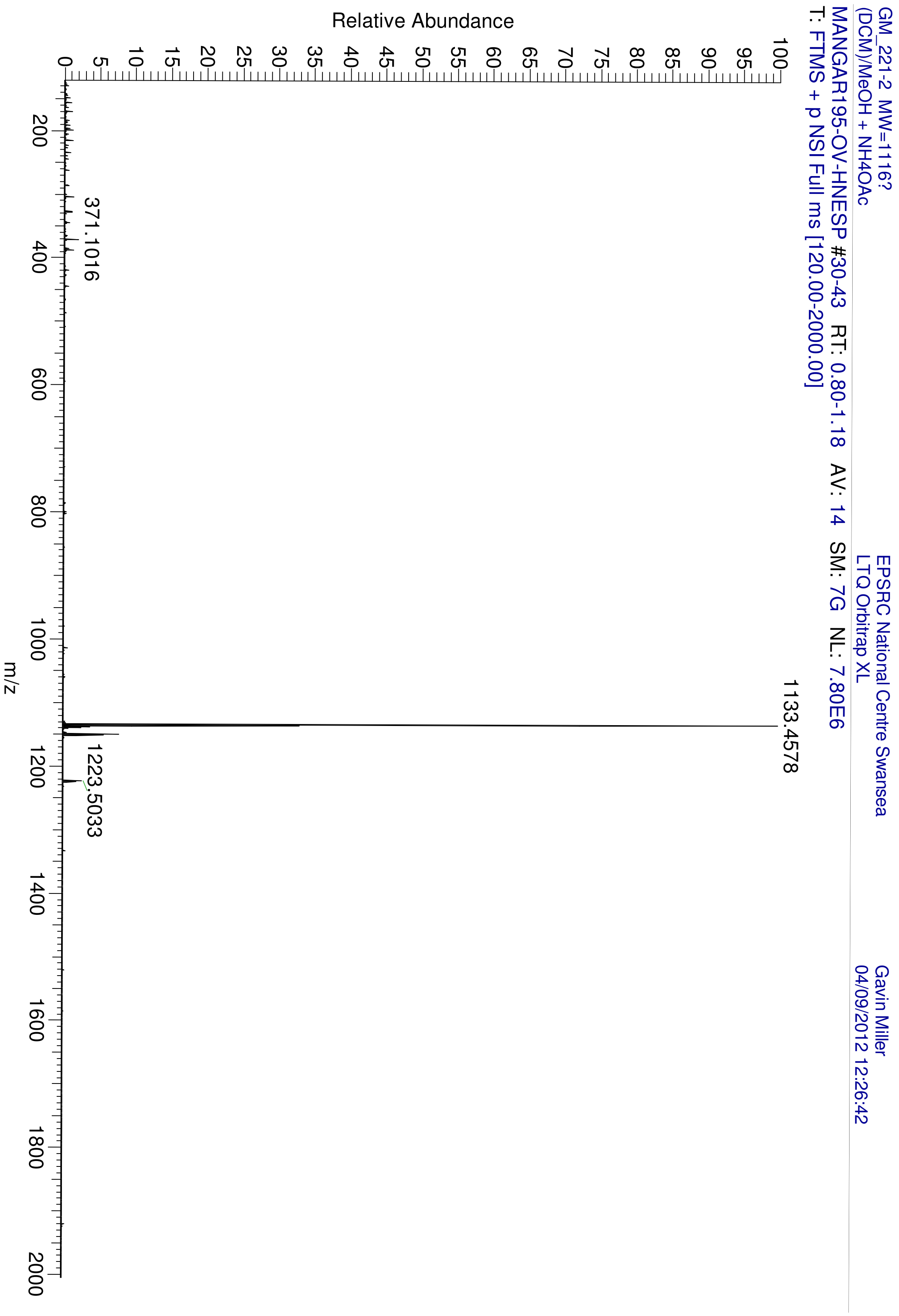

Methyl (phenyl 4-O-(2-azido-3,6-di-O-benzyl-2-deoxy-4-O-[(*S*)-2,3-bis(benzyloxy)propoxy]-α-D-glucopyranosyl)-2-O-benzoyl-3-O-benzyl-1-thio-α-L-idopyranoside)-uronate (20)

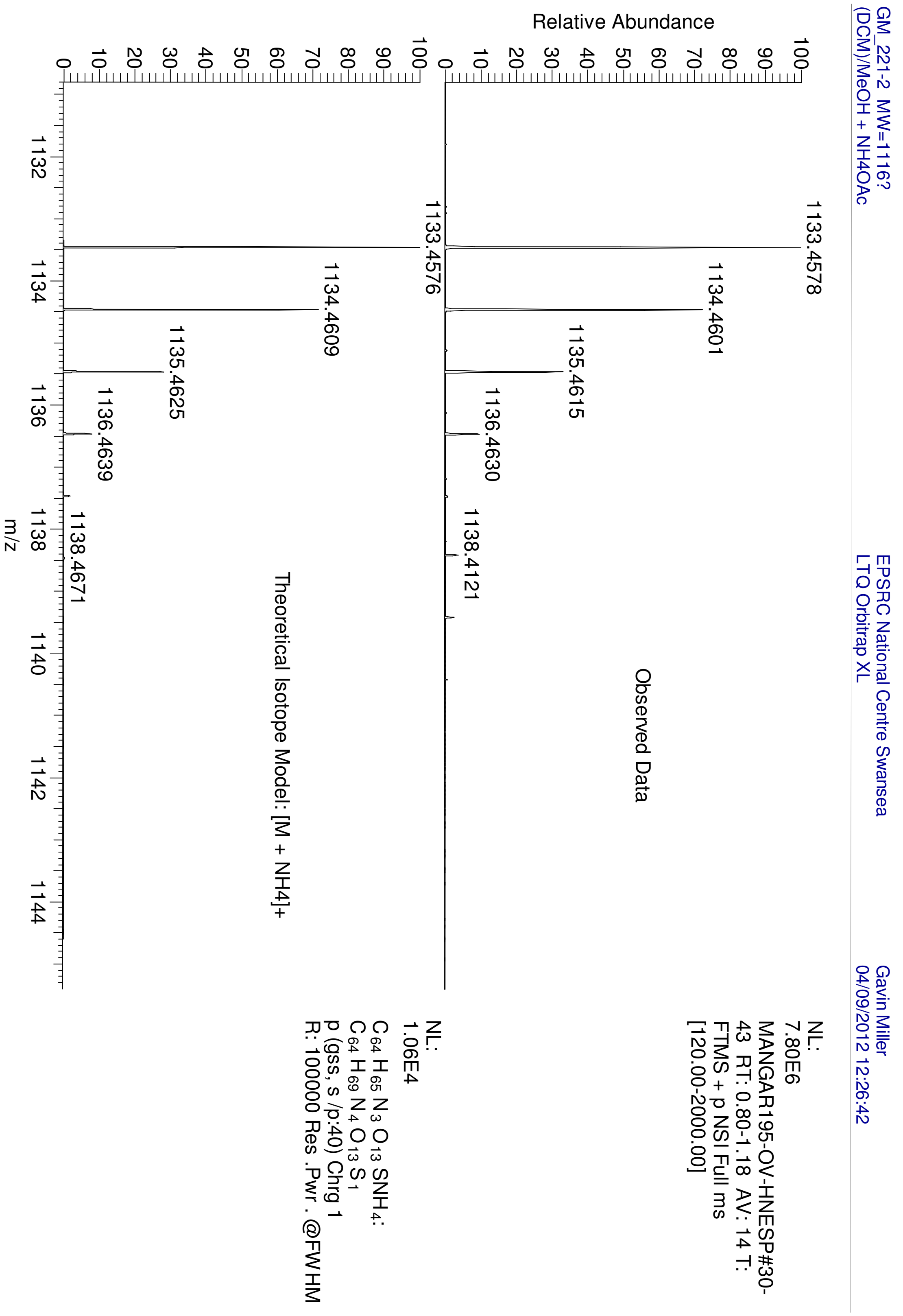

2-Azido-3-O-benzyl-6-O-benzoyl-2-deoxy-4-O-[(*S*)-2,3-bis(benzyloxy)propoxy]-αβ-D-glucopyranose

^{1}H NMR

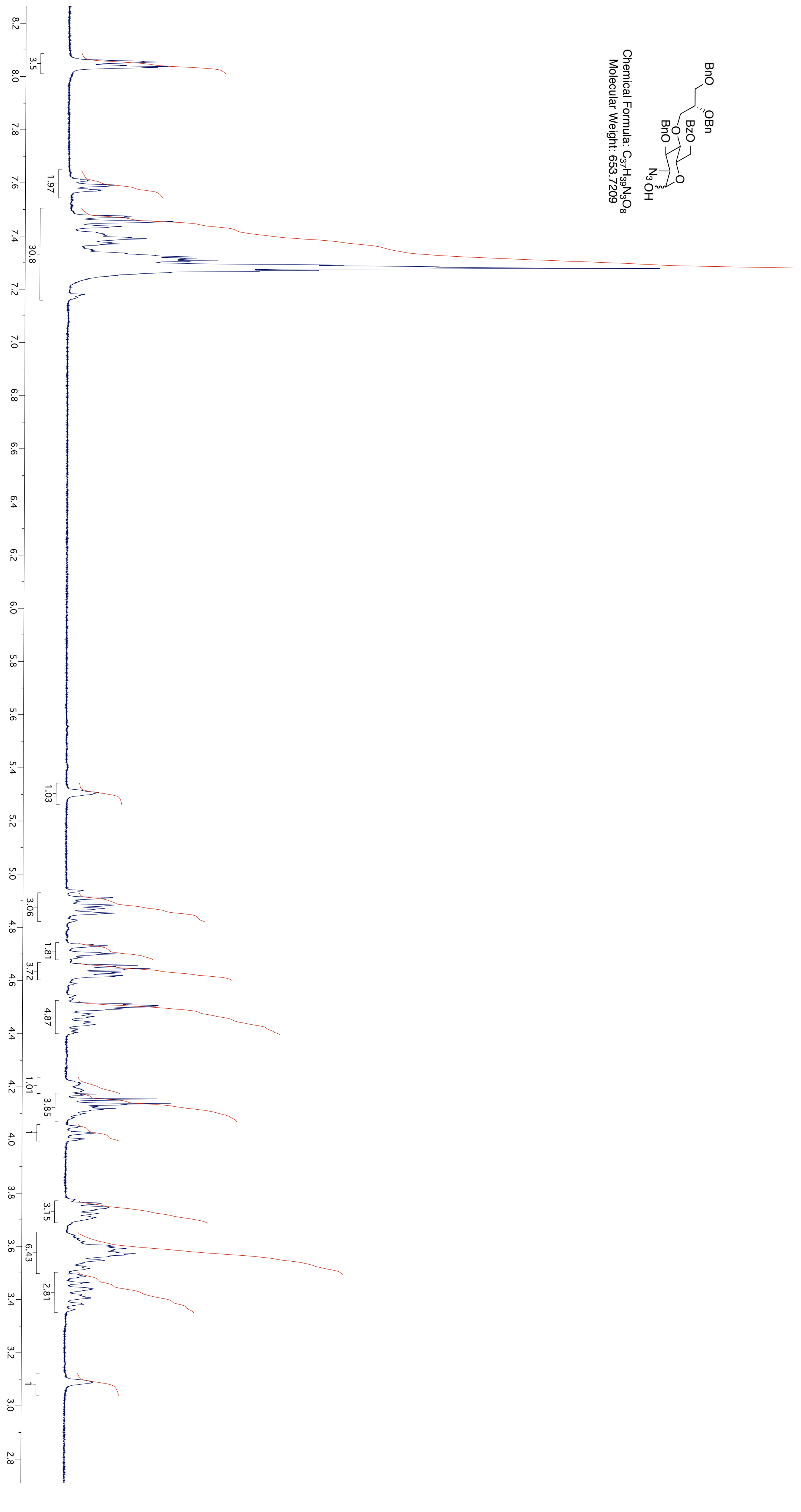

400 MHz, $CDCl_3$

2-Azido-3-O-benzyl-6-O-benzoyl-2-deoxy-4-O-[(*S*)-2,3-bis(benzyloxy)propoxy]-αβ-D-glucopyranose

COSY

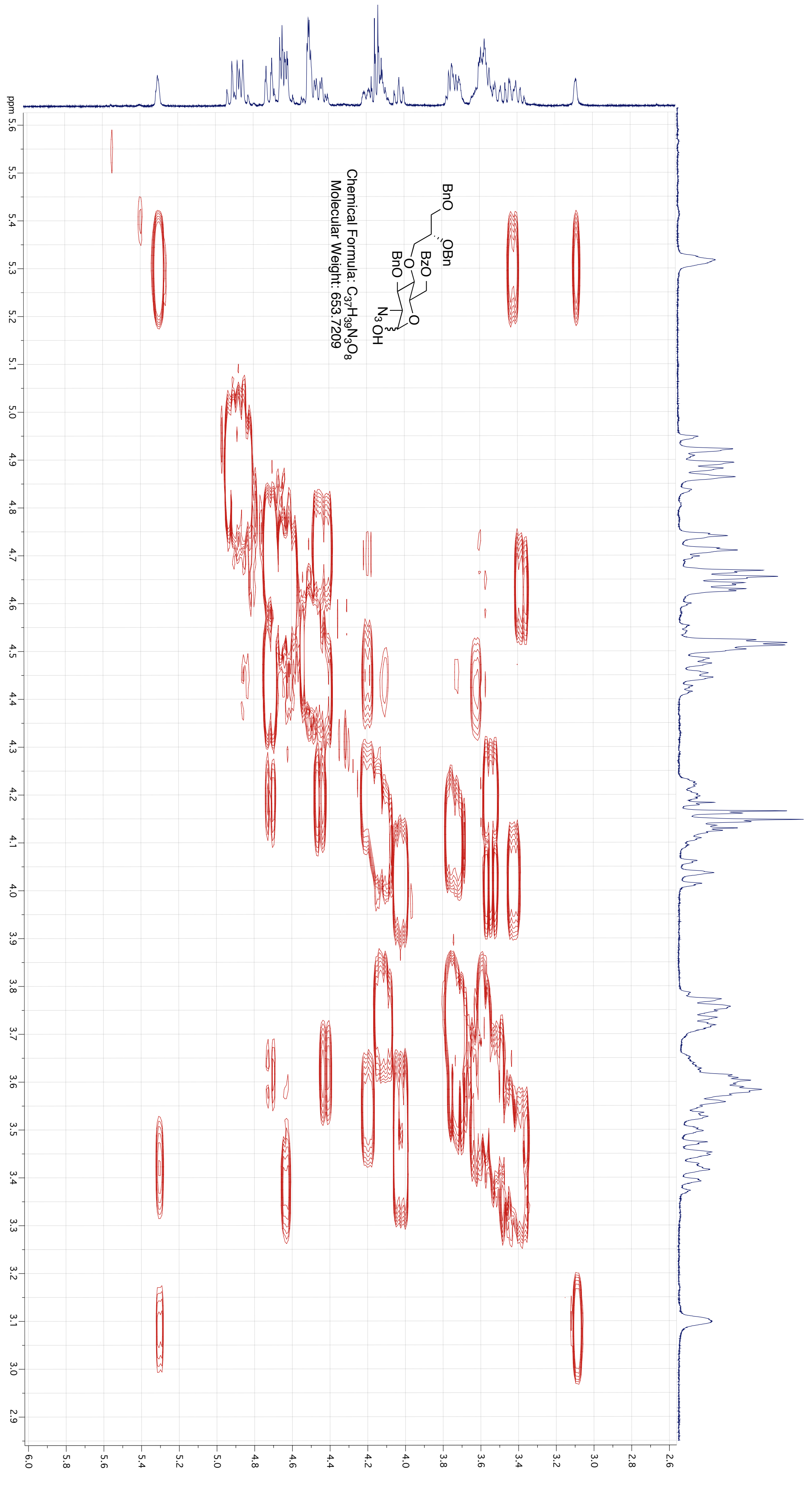

400 MHz, $CDCl_3$

2-Azido-3-O-benzyl-6-O-benzoyl-2-deoxy-4-O-[(*S*)-2,3-bis(benzyloxy)propoxy]-1-O-trichloroacetimidate-αβ-D-glucopyranoside

^{1}H NMR

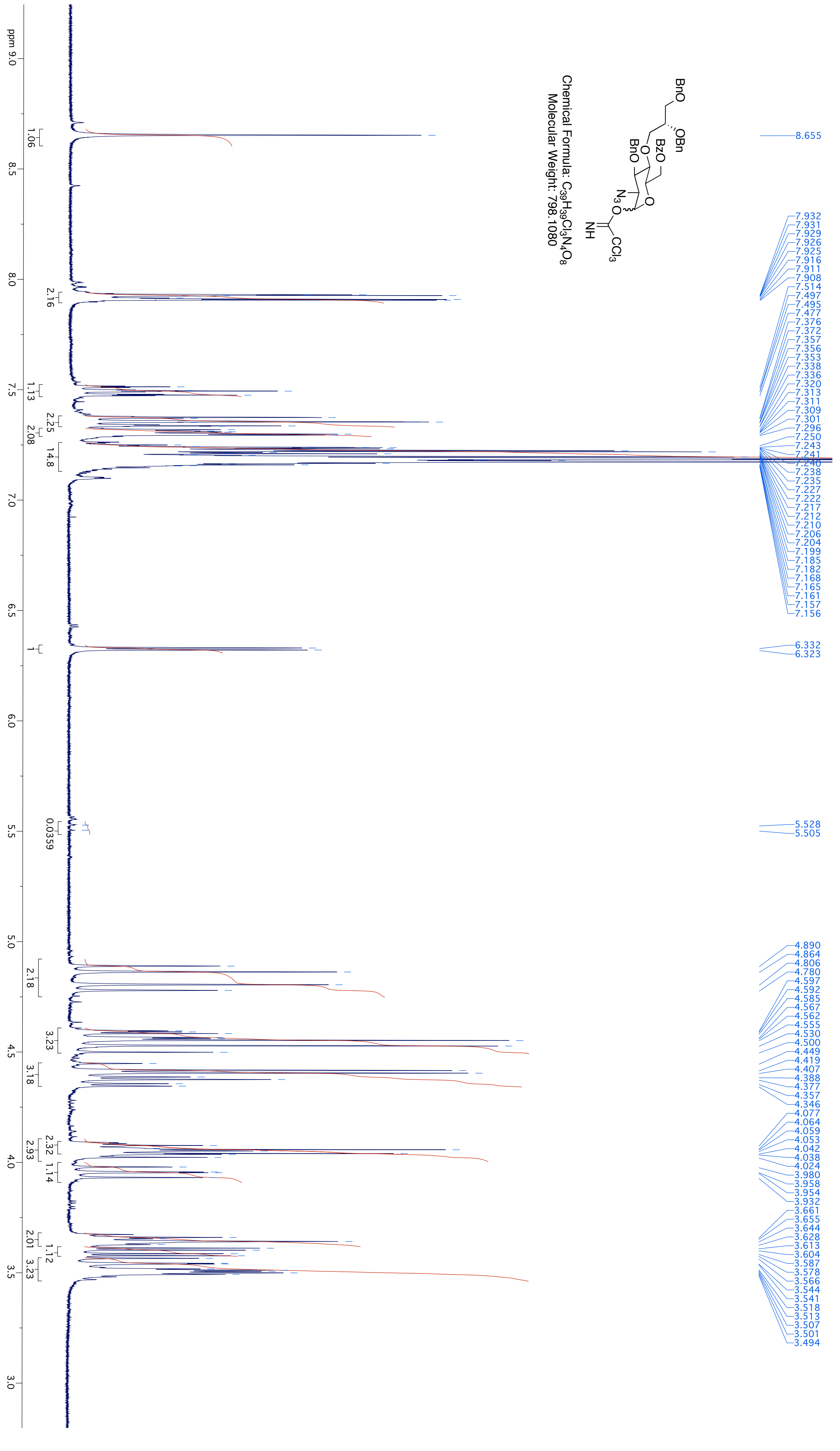

400 MHz, $CDCl_3$

Methyl (phenyl 4-O-(2-azido-3-O-benzyl-6-O-benzoyl-2-deoxy-4-O-[(*S*)-2,3-bis(benzyloxy)propoxy]-α-D-glucopyranosyl)-2-O-benzoyl-3-O-benzyl-1-thio-α-L-idopyranoside)-uronate (21)

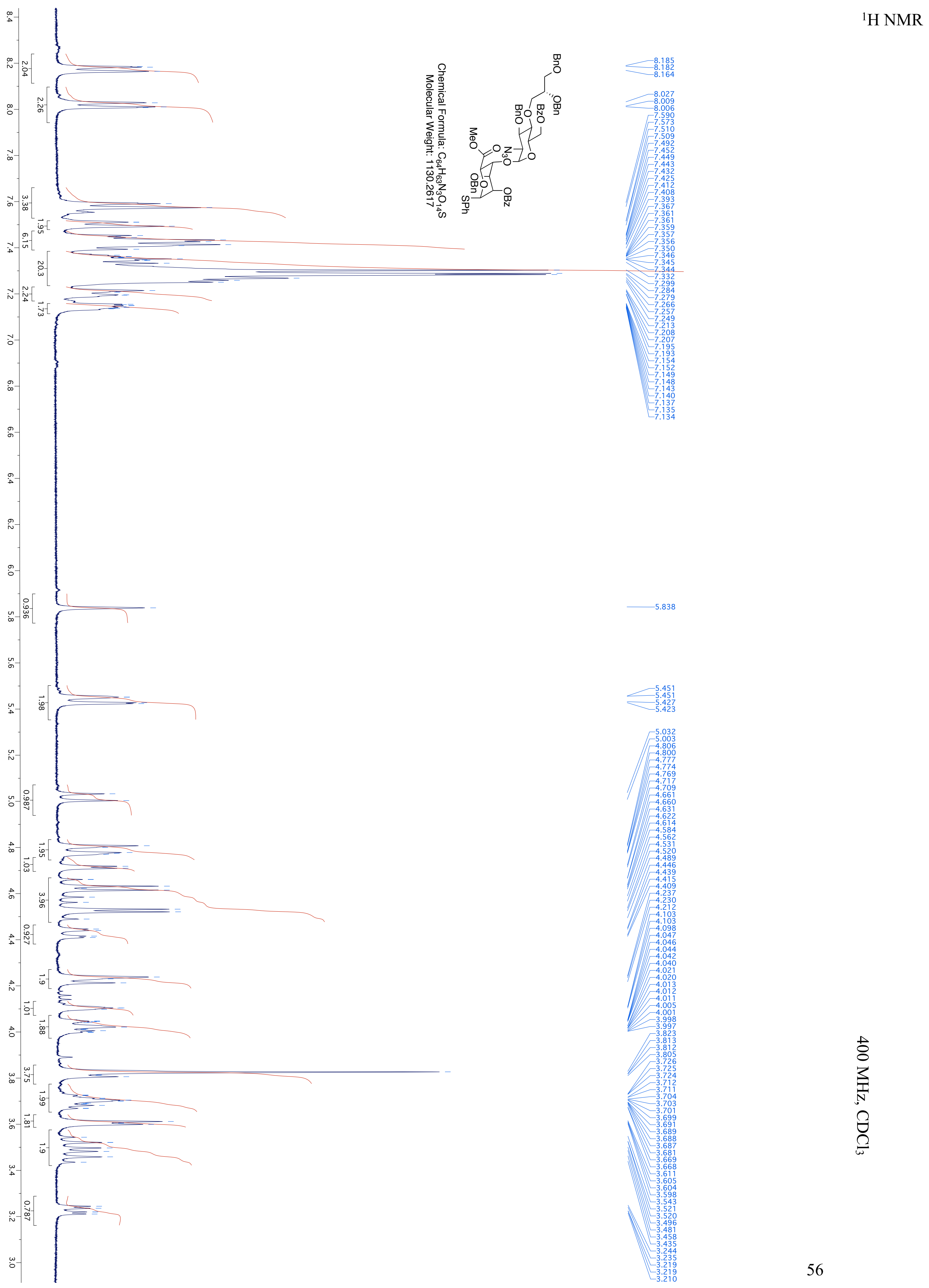

Methyl (phenyl 4-O-(2-azido-3-O-benzyl-6-O-benzoyl-2-deoxy-4-O-[(*S*)-2,3-bis(benzyloxy)propoxy]-α-D-glucopyranosyl)-2-O-benzoyl-3-O-benzyl-1-thio-α-L-idopyranoside)-uronate (21)

^{13}C NMR

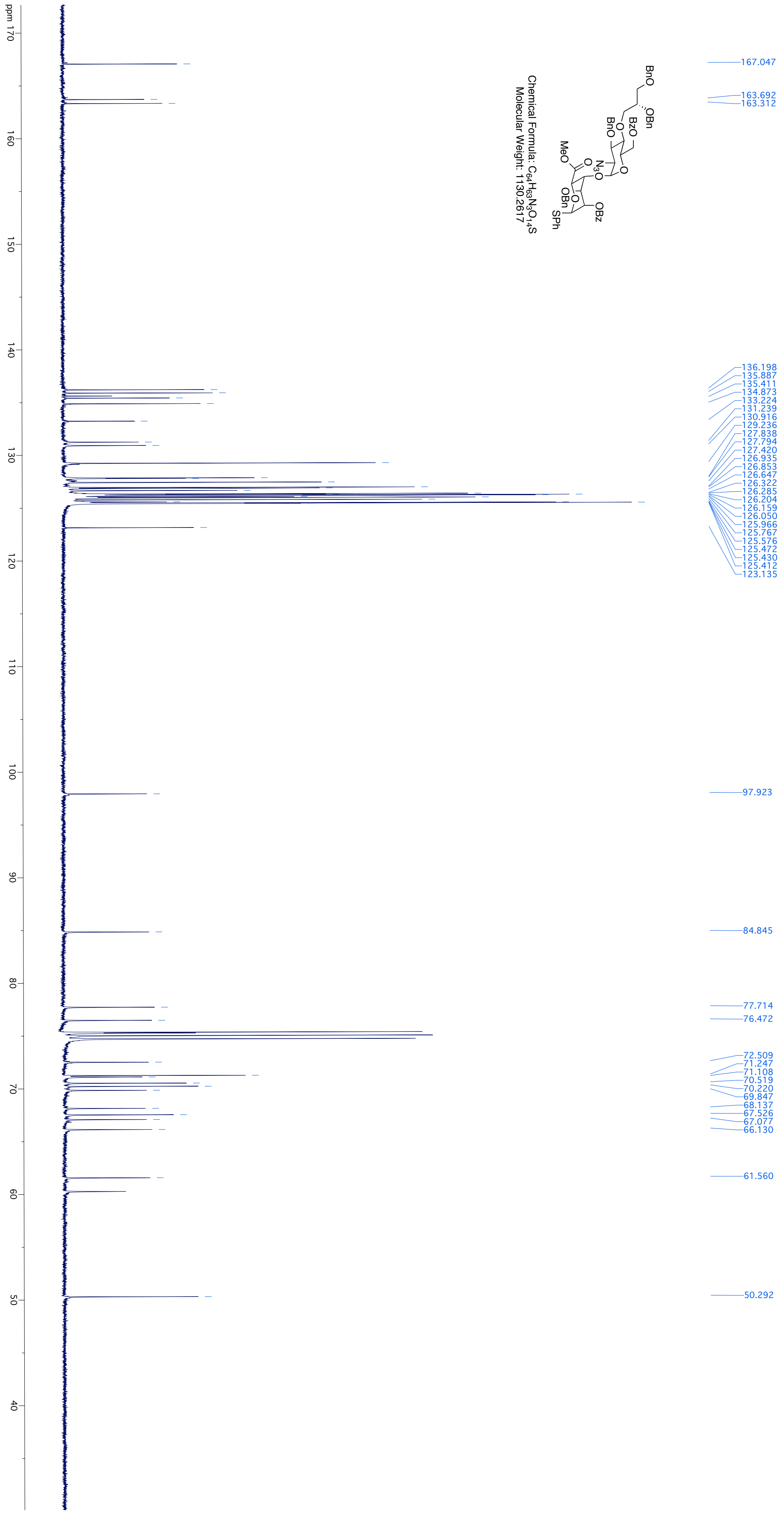

100 MHz, CDCl$_3$

Methyl (phenyl 4-O-(2-azido-3-O-benzyl-6-O-benzoyl-2-deoxy-4-O-[(*S*)-2,3-bis(benzyloxy)propoxy]-α-D-glucopyranosyl)-2-O-benzoyl-3-O-benzyl-1-thio-α-L-idopyranoside)-uronate (21)

COSY

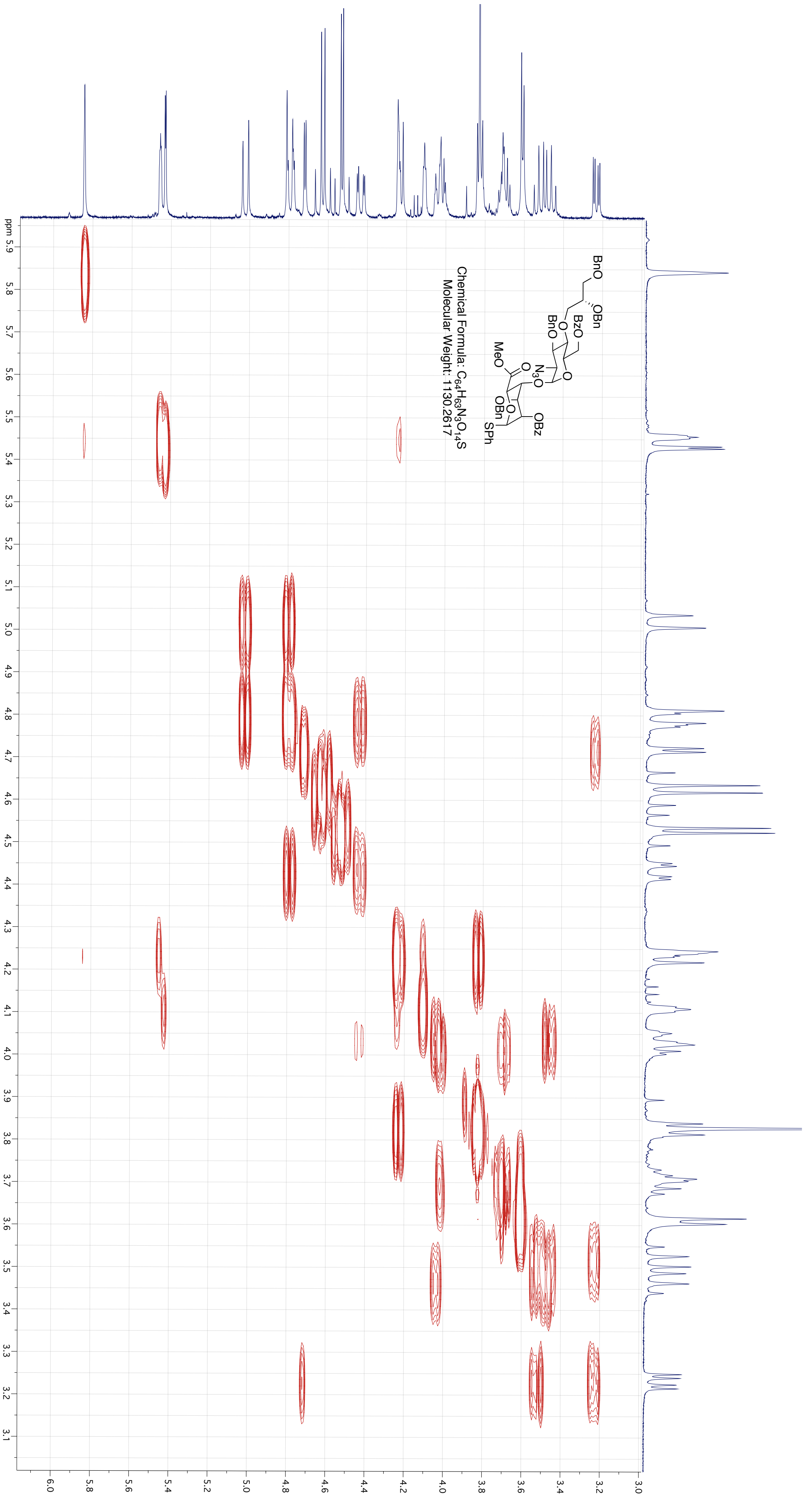

400 MHz, CDCl3

Methyl (phenyl 4-O-(2-azido-3-O-benzyl-6-O-benzoyl-2-deoxy-4-O-[(*S*)-2,3-bis(benzyloxy)propoxy]-α-D-glucopyranosyl)-2-O-benzoyl-3-O-benzyl-1-thio-α-L-idopyranoside)-uronate (21)

HMQC

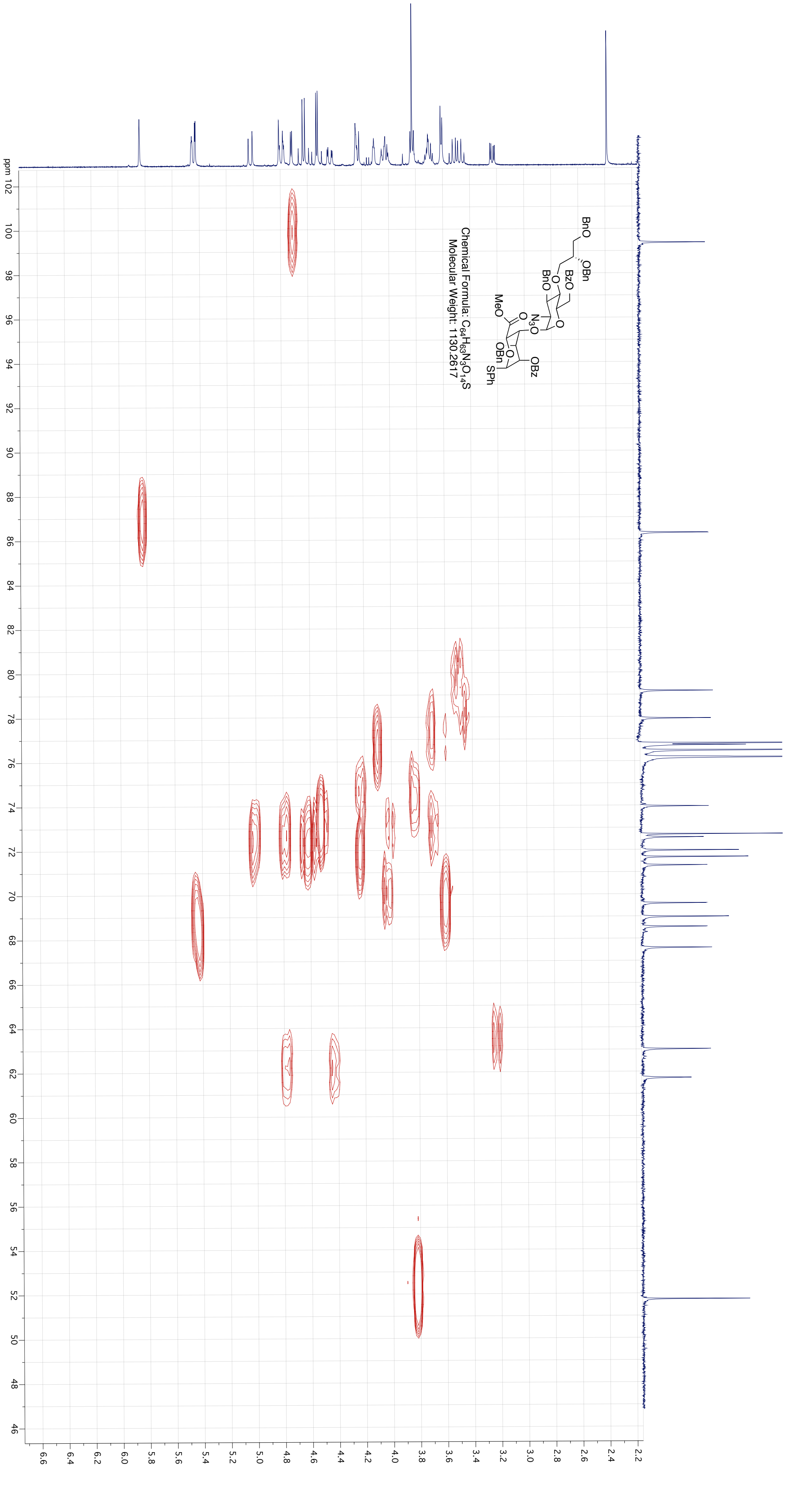

400 MHz, CDCl3

Methyl (phenyl 4-O-(2-azido-3-O-benzyl-6-O-benzoyl-2-deoxy-4-O-[(*S*)-2,3-bis(benzyloxy)propoxy]-α-D-glucopyranosyl)-2-O-benzoyl-3-O-benzyl-1-thio-α-L-idopyranoside)-uronate (21)

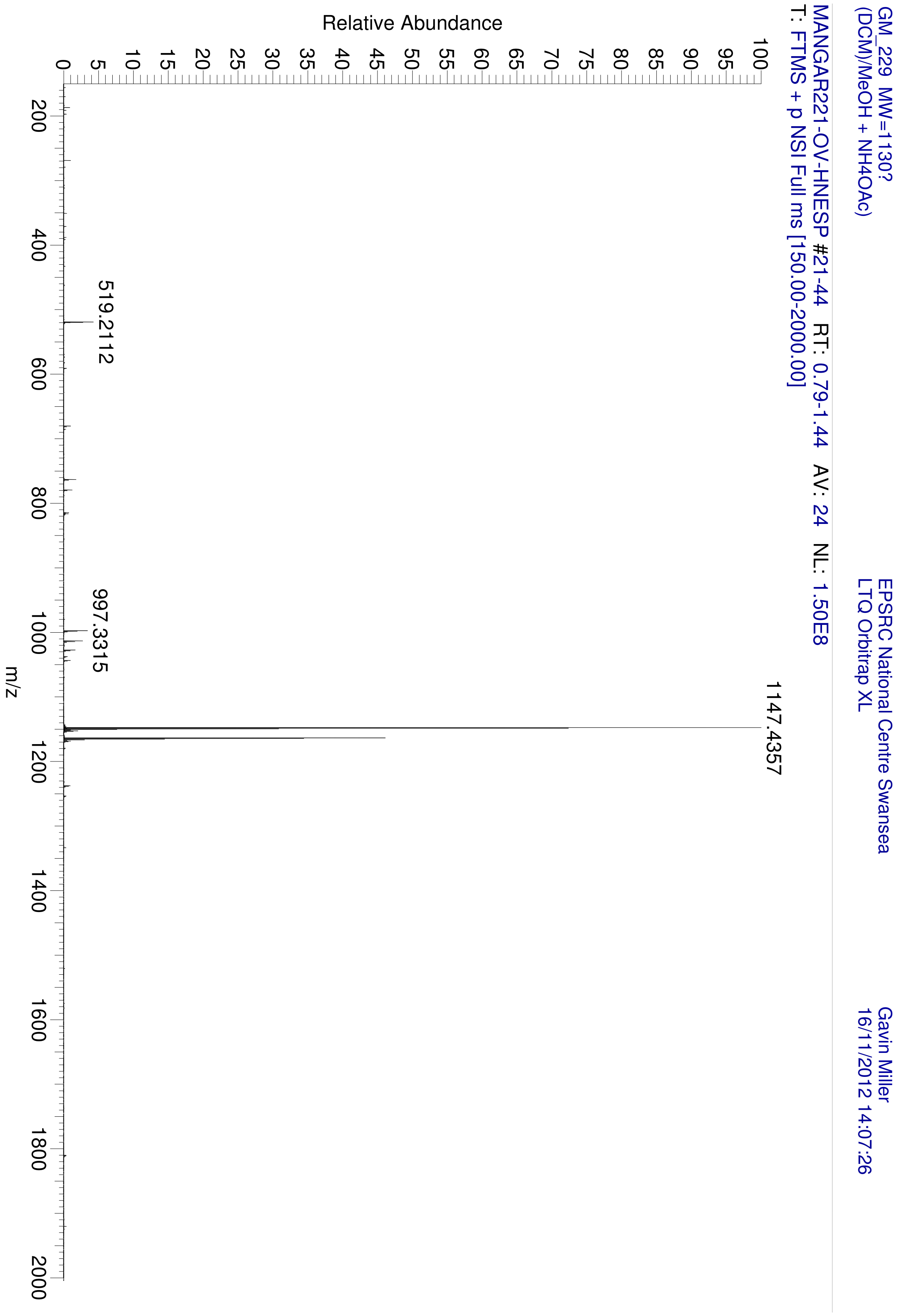

Methyl (phenyl 4-O-(2-azido-3-O-benzyl-6-O-benzoyl-2-deoxy-4-O-[(*S*)-2,3-bis(benzyloxy)propoxy]-α-D-glucopyranosyl)-2-O-benzoyl-3-O-benzyl-1-thio-α-L-idopyranoside)-uronate (21)

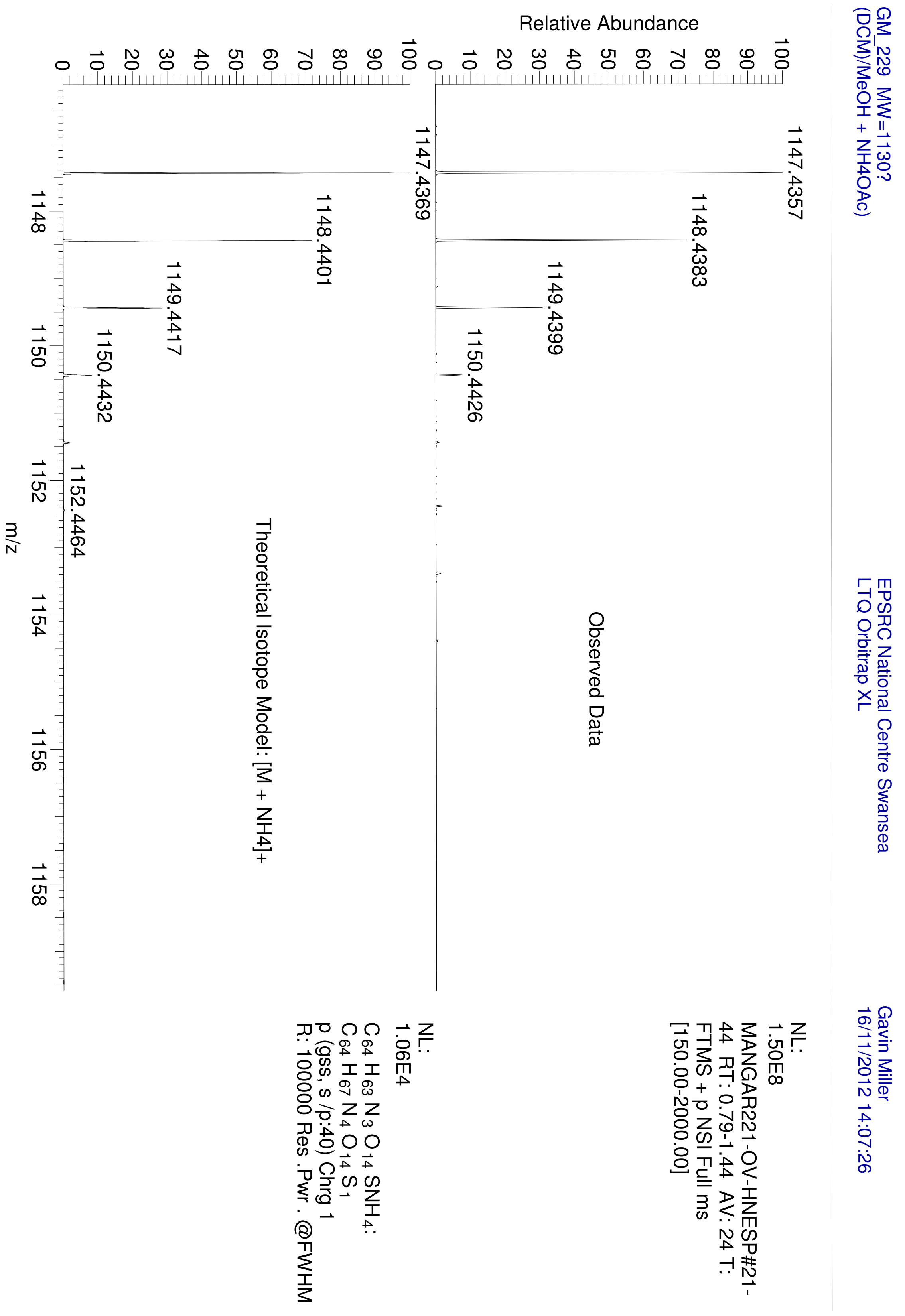

Decasaccharide 25

^1H NMR

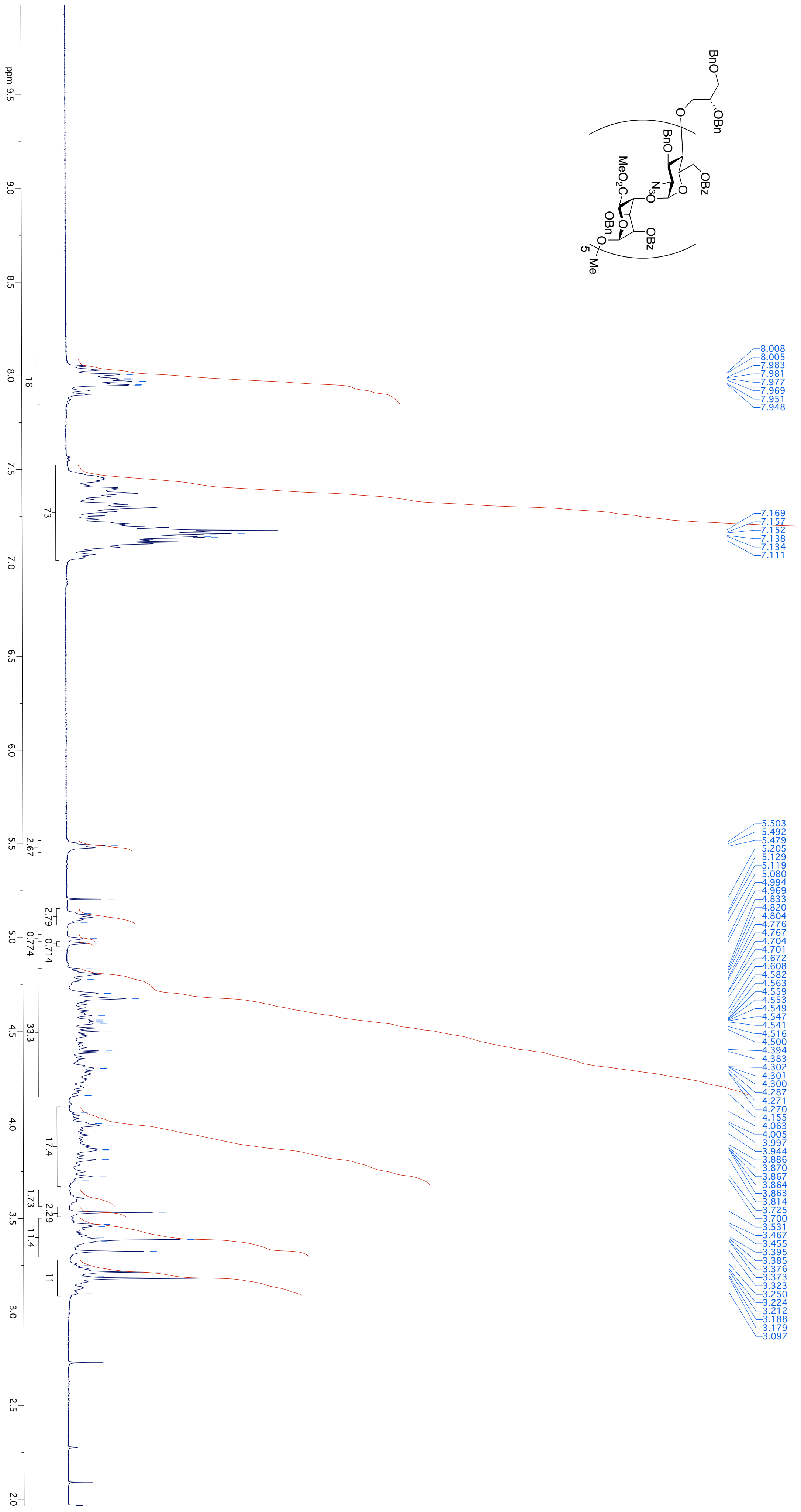

400 MHz, $CDCl_3$

Decasaccharide 25

^{13}C NMR

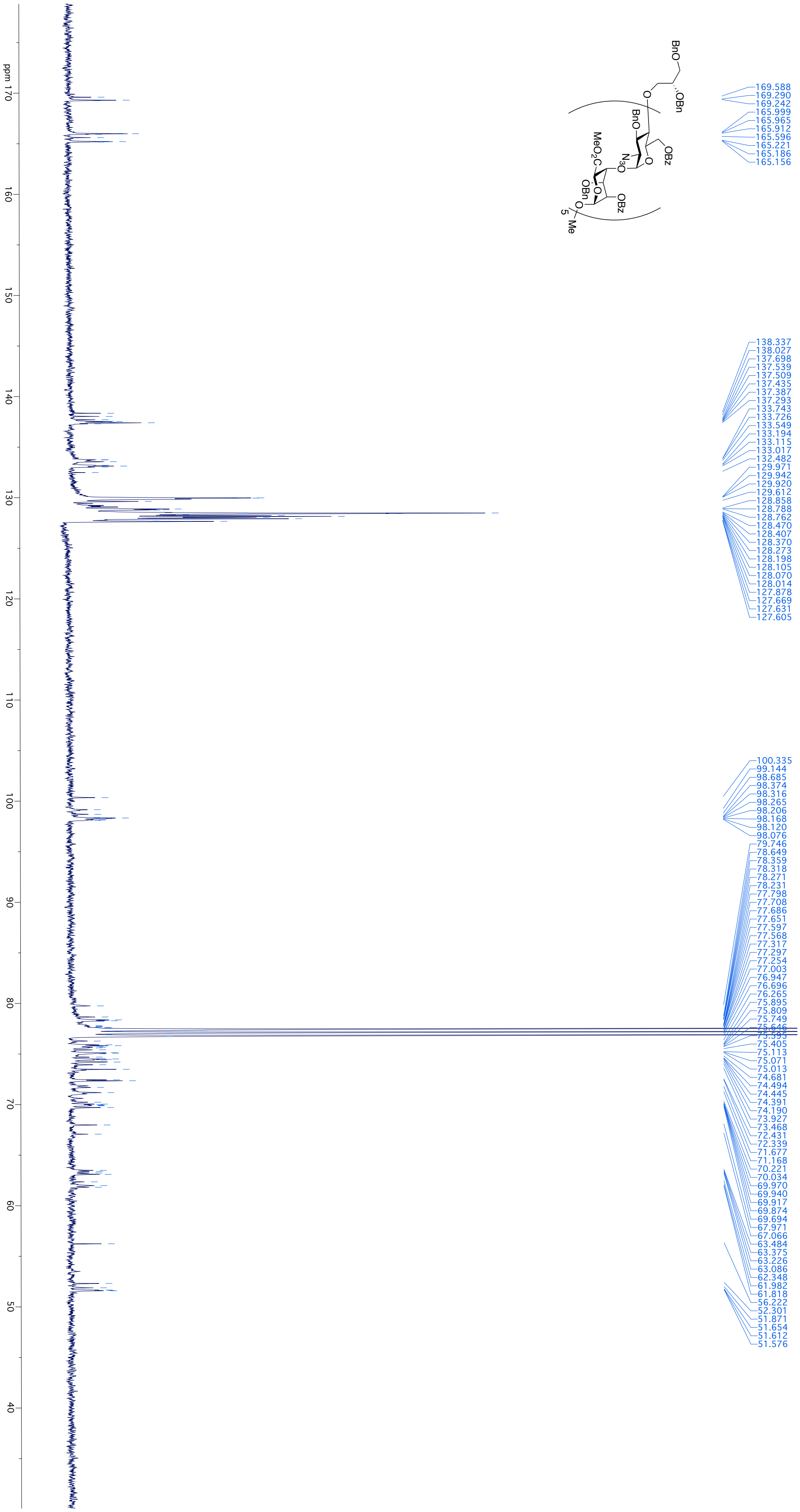

100 MHz, $CDCl_3$

Decasaccharide 25

COSY

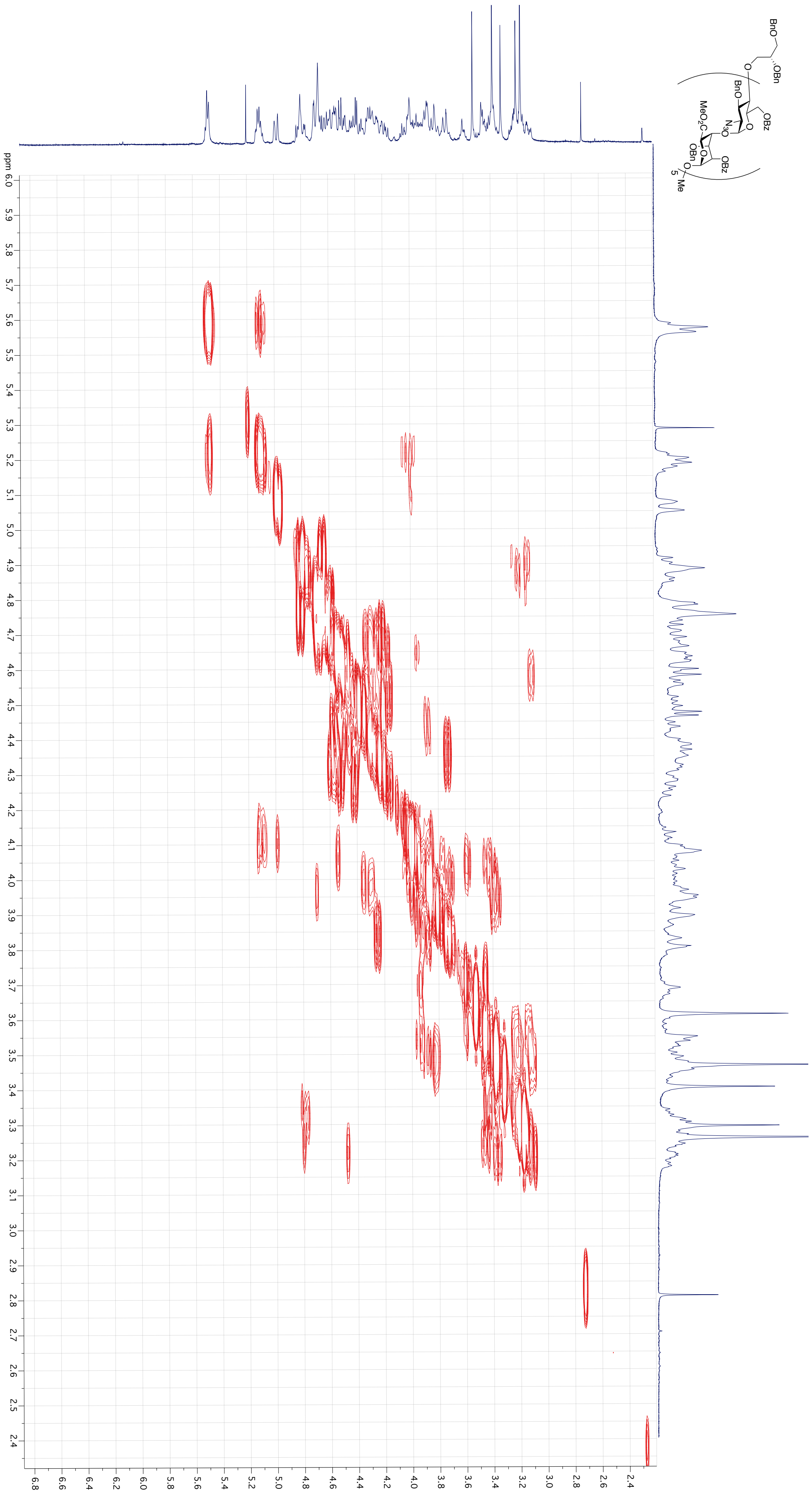

400 MHz, $CDCl_3$

Decasaccharide 25

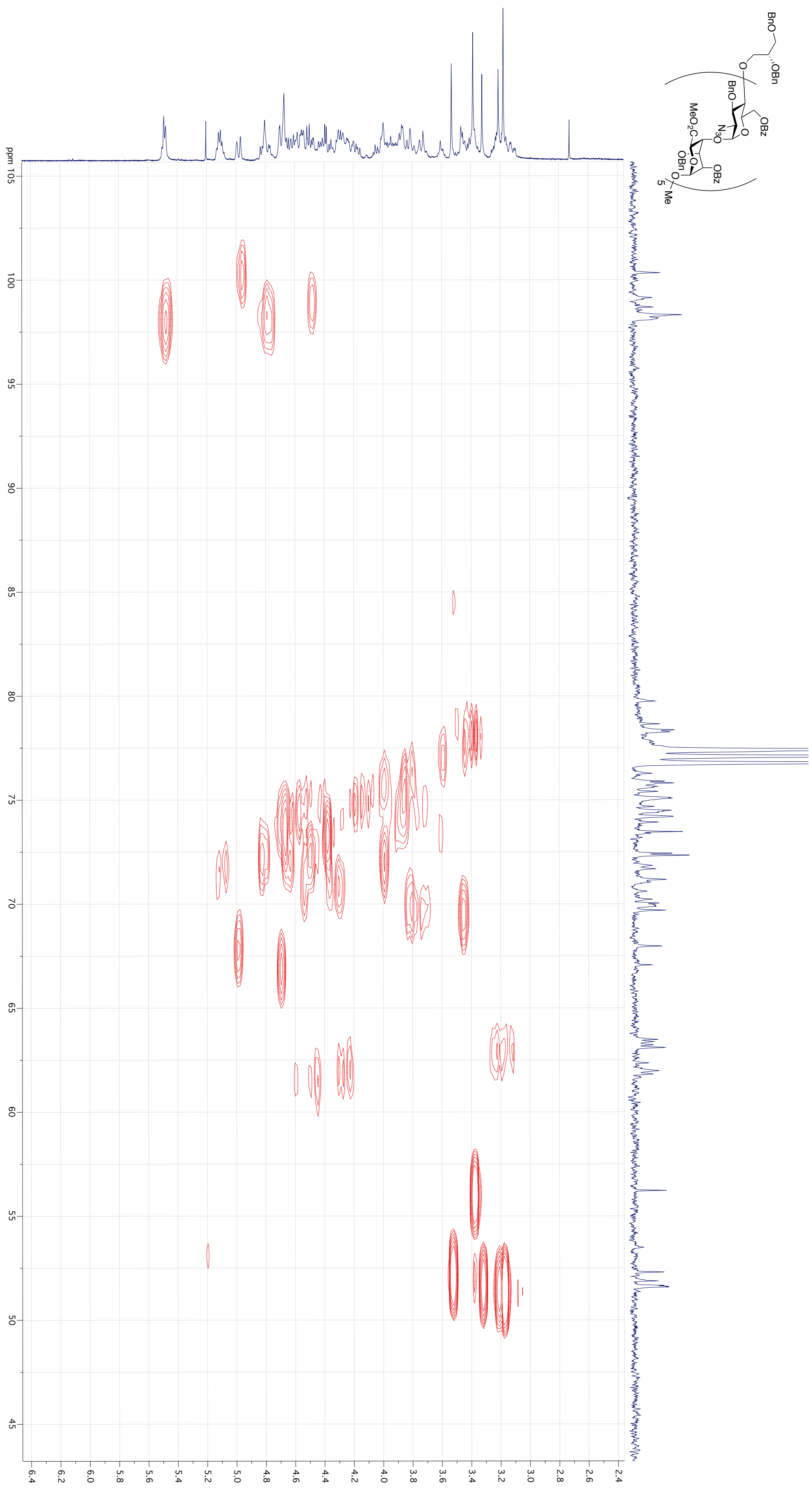

HMQC

400 MHz, $CDCl_3$

Decasaccharide 25- ESI MS

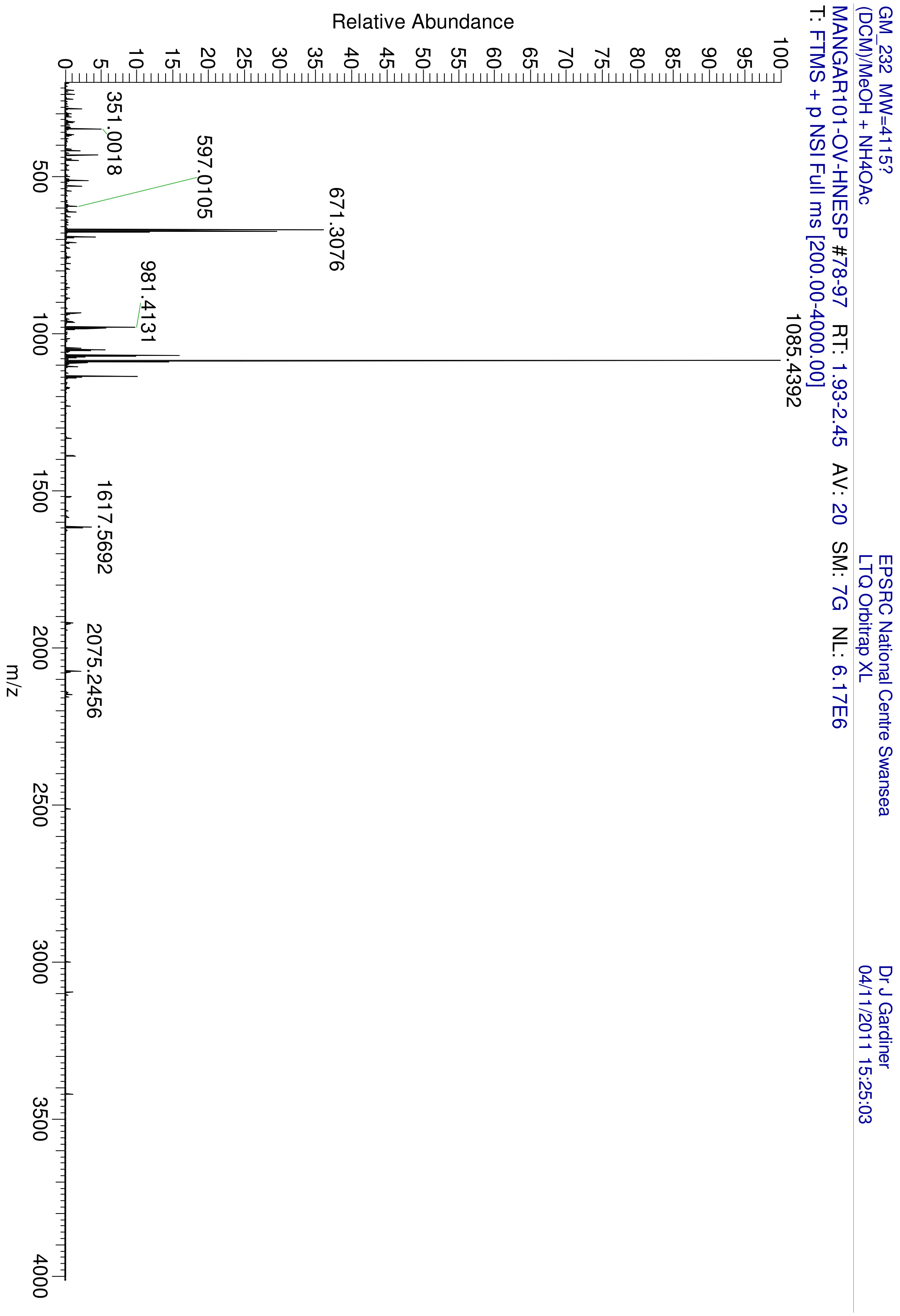

Decasaccharide 25-ESI MS Isotope Pattern

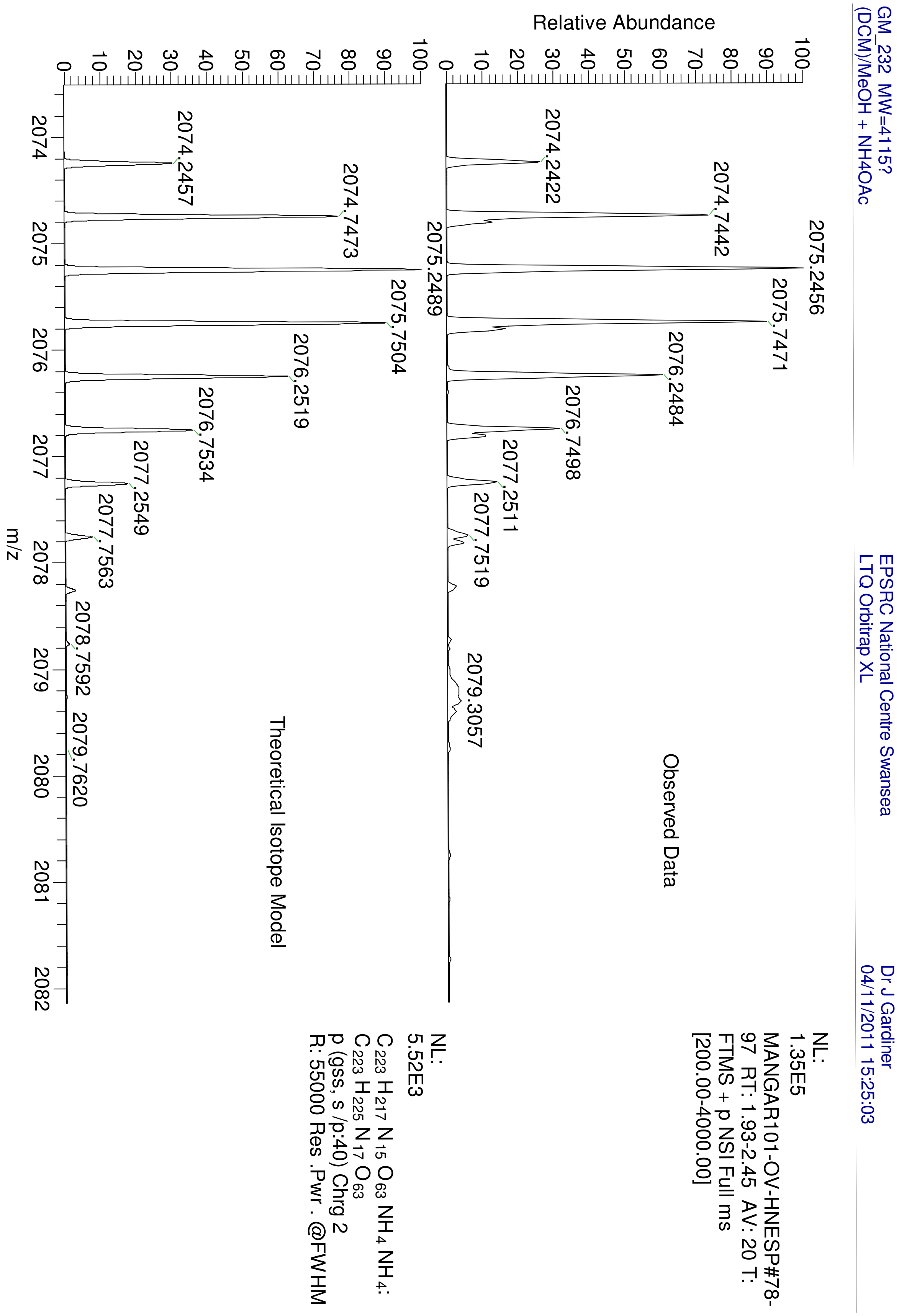

Dodecasaccharide 24

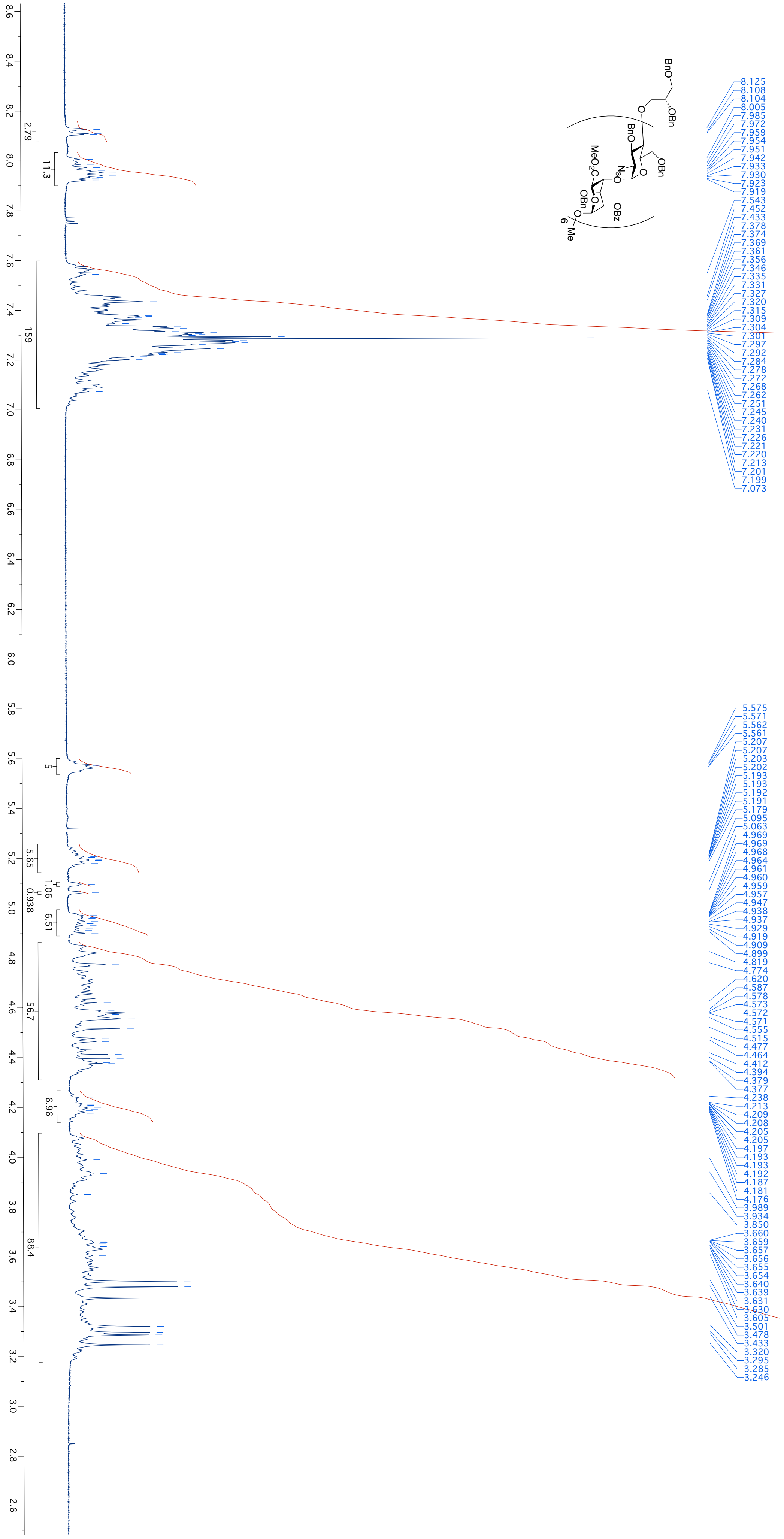

^{1}H NMR

400 MHz, $CDCl_3$

Dodecasaccharide 24

^{13}C NMR

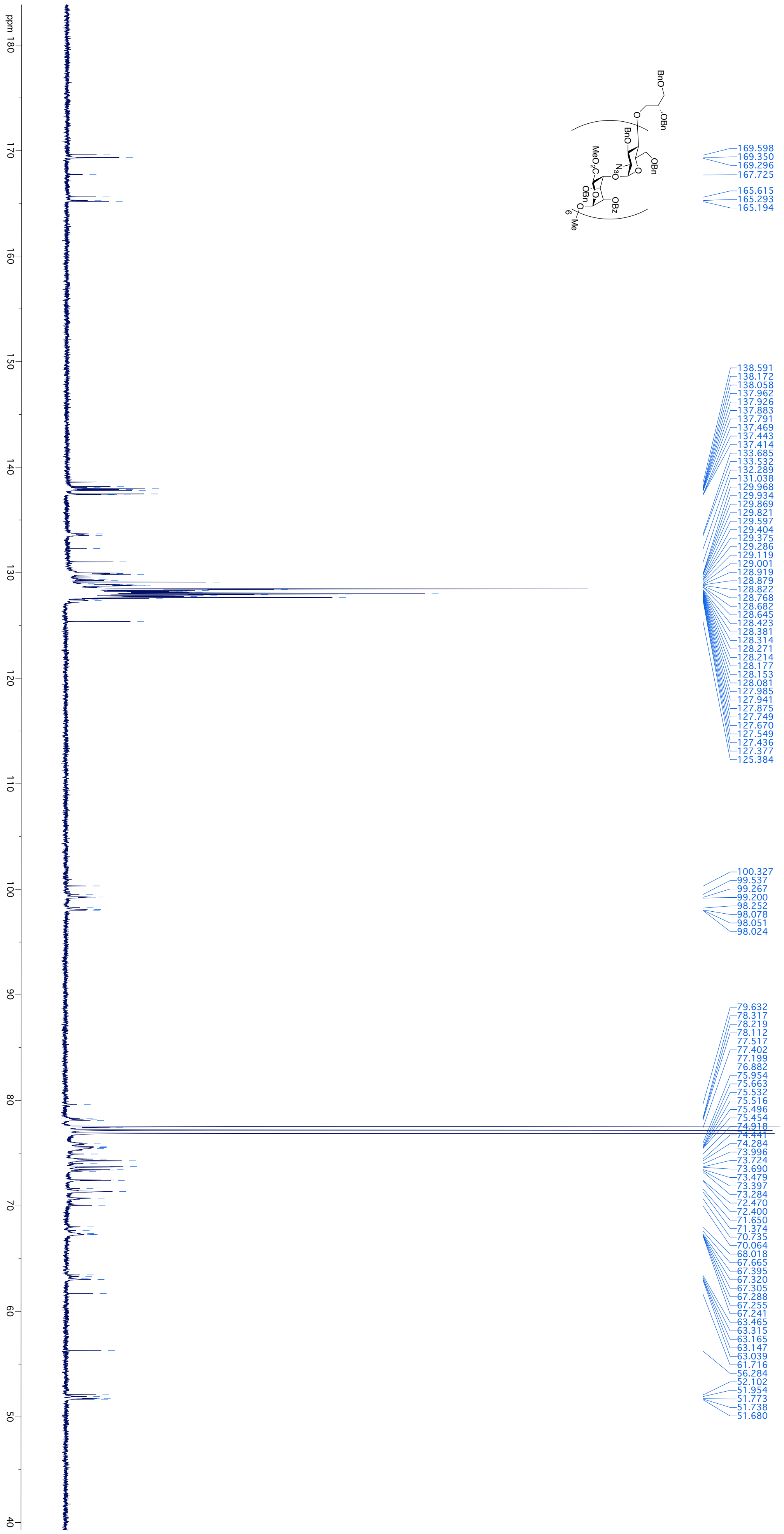

100 MHz, $CDCl_3$

Dodecasaccharide 24

COSY

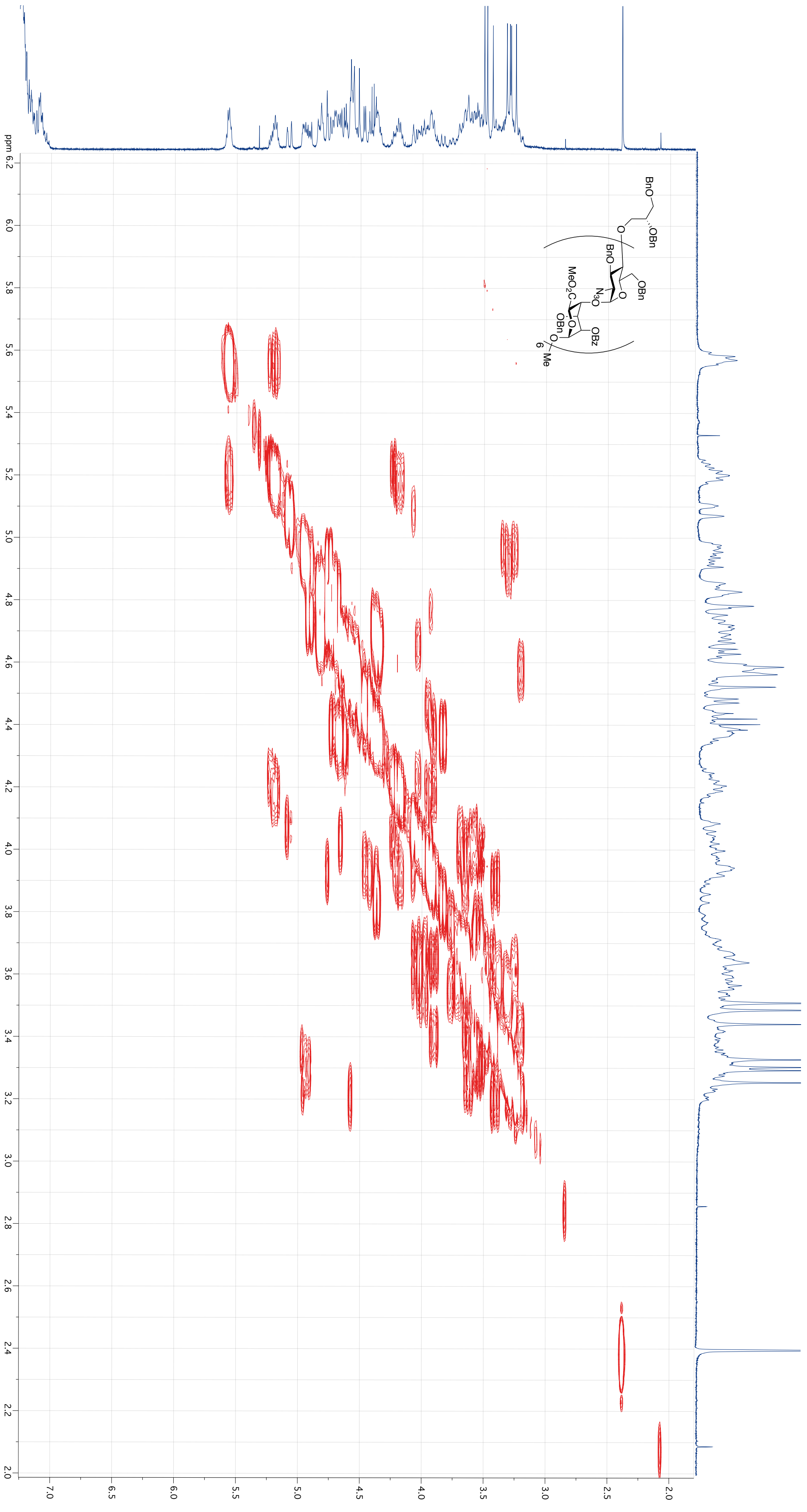

400 MHz, $CDCl_3$

Dodecasaccharide 24

HSQC

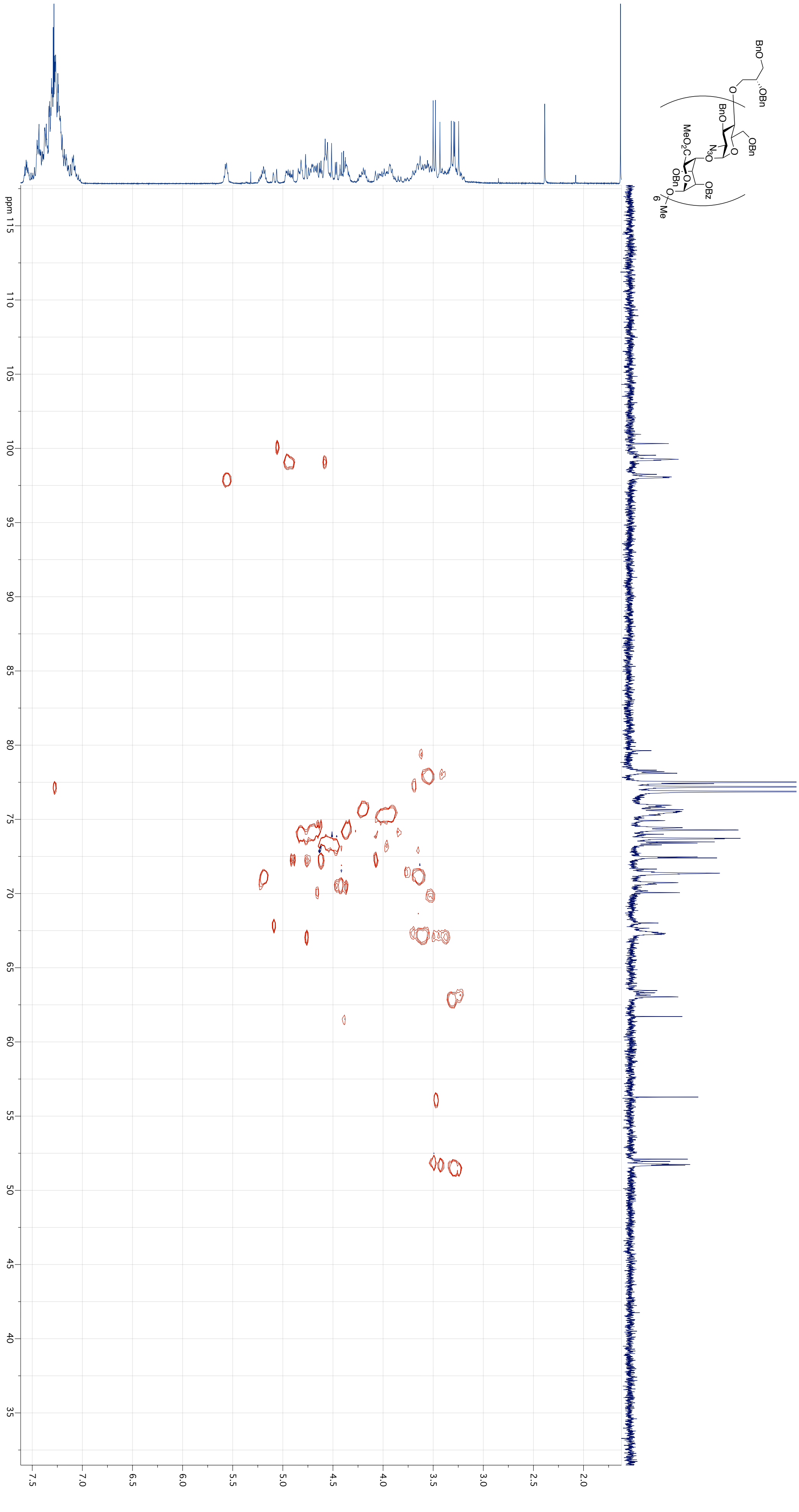

400 MHz, $CDCl_3$

Dodecasaccharide 24 - MALDI MS and isotope pattern

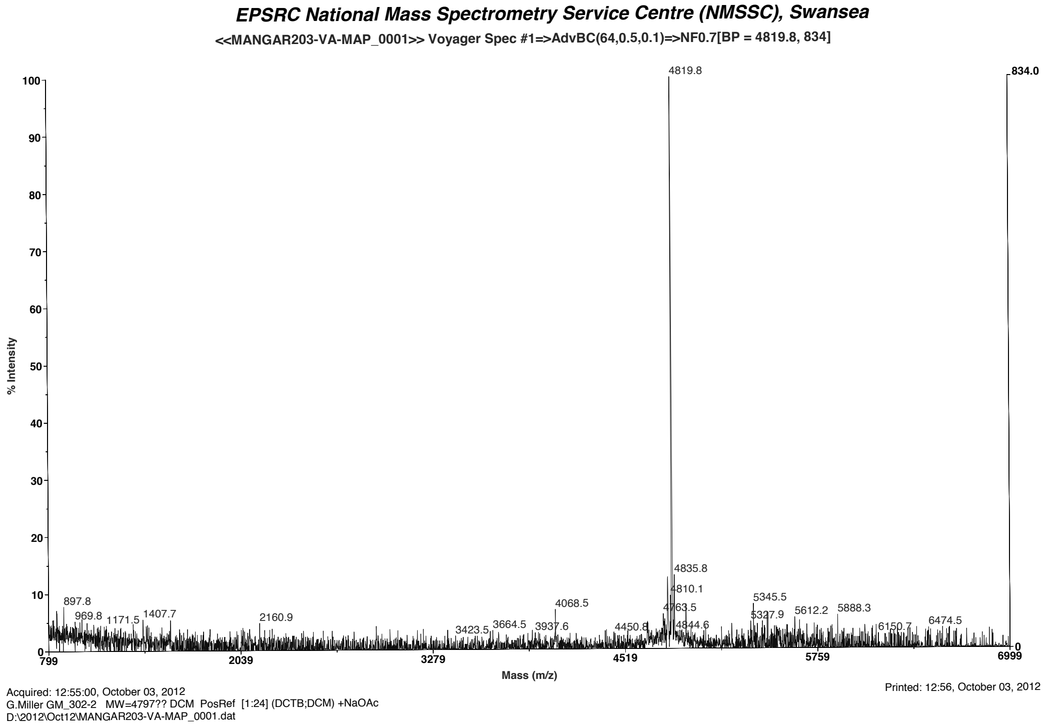

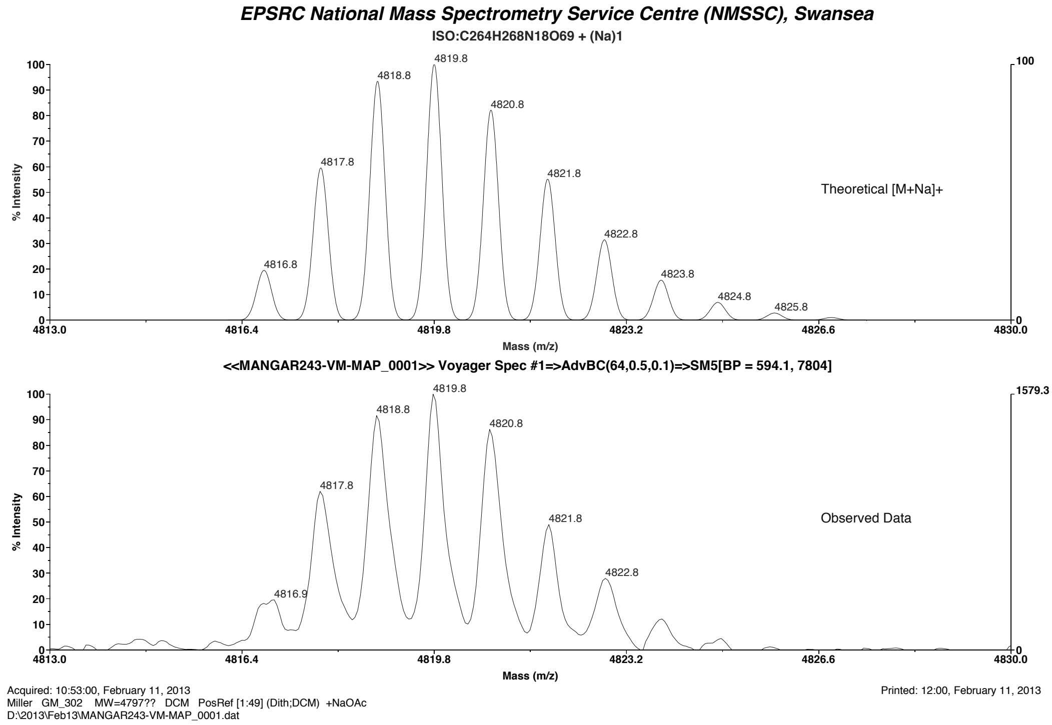

Decasaccharide Saponification

^{1}H NMR

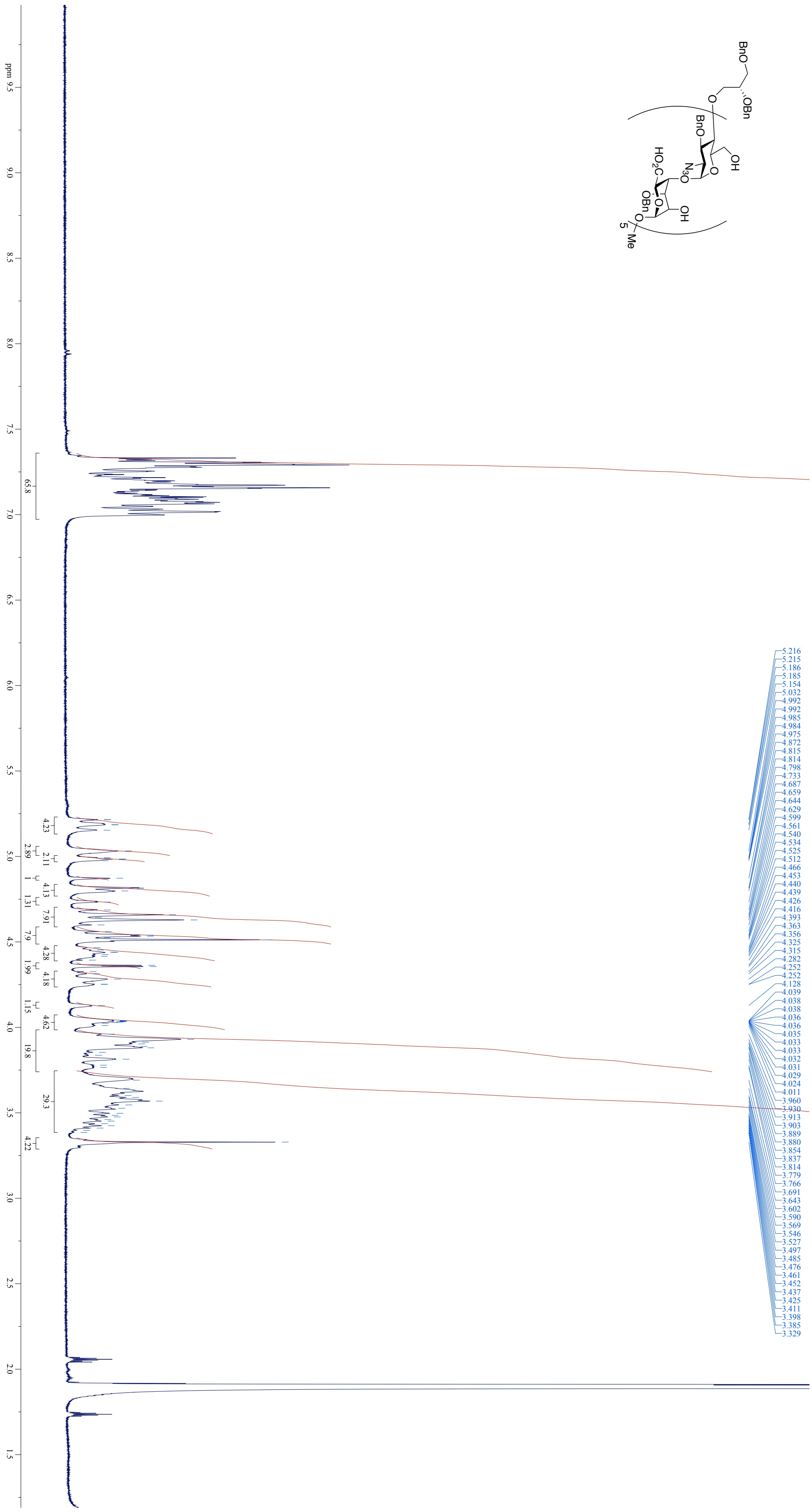

400 MHz, AcOH

Decasaccharide Saponification

COSY

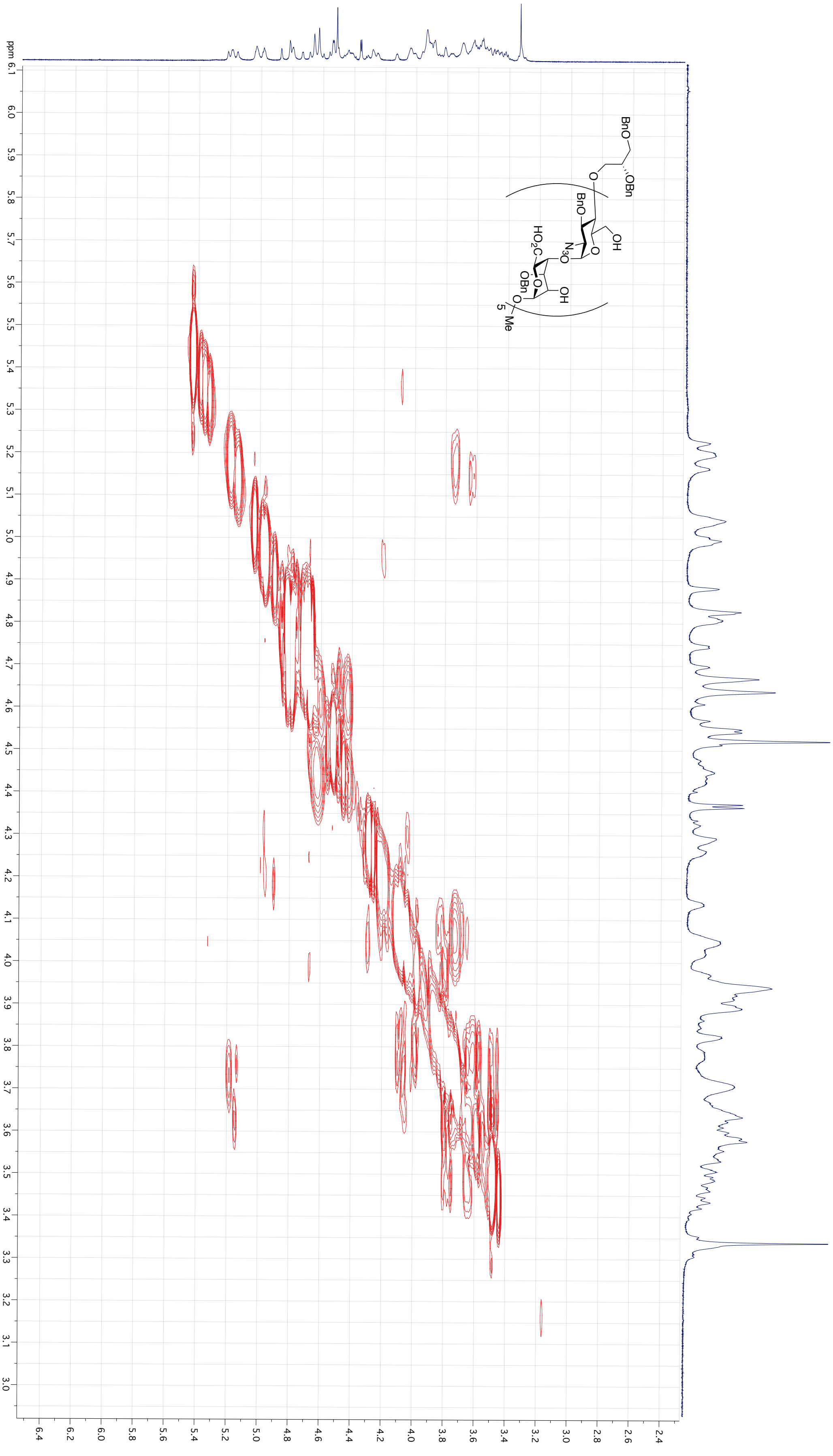

400 MHz, AcOH

Decasaccharide Saponification- ESI MS

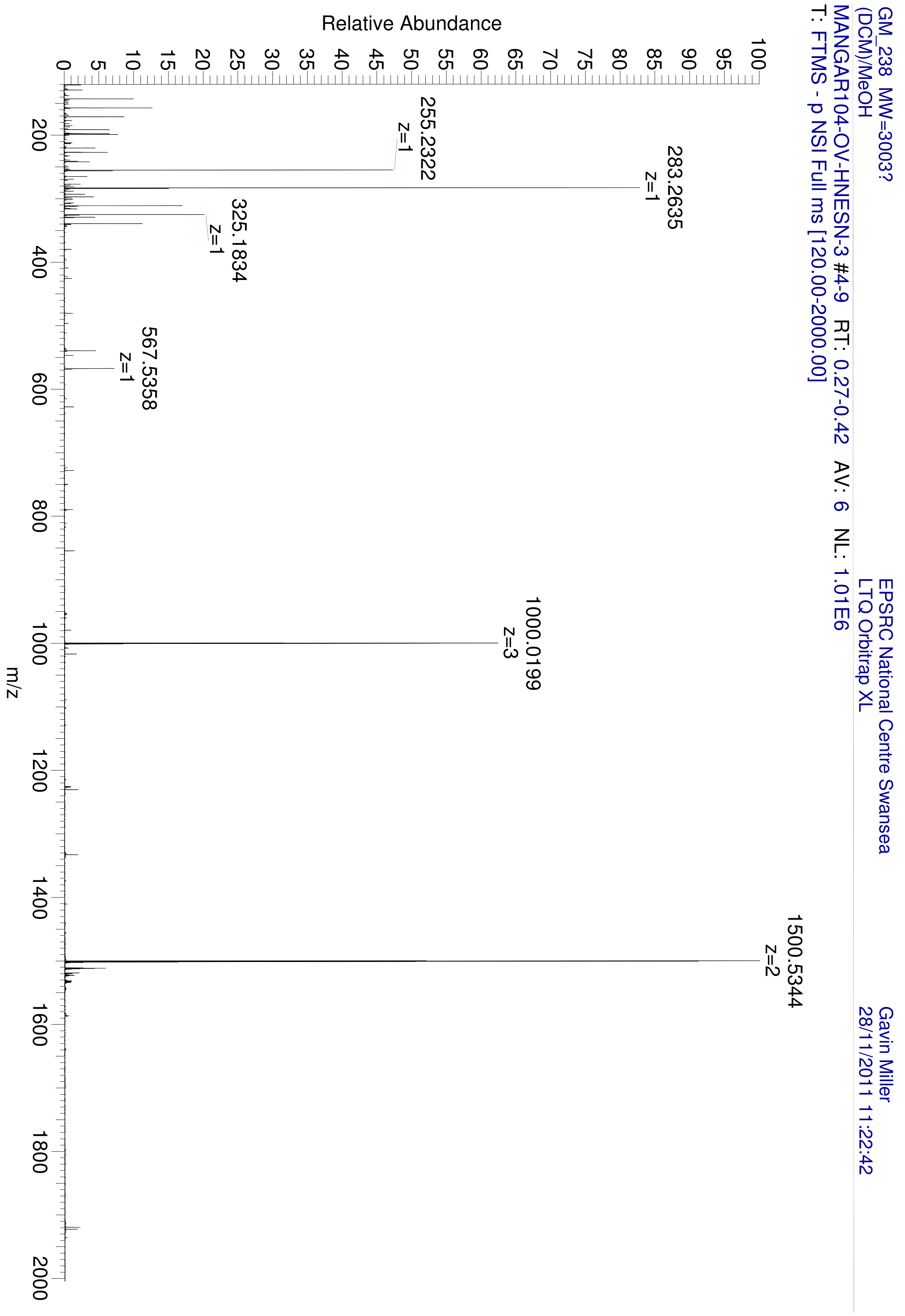

Decasaccharide Saponification-**ESI MS Isotope Pattern**

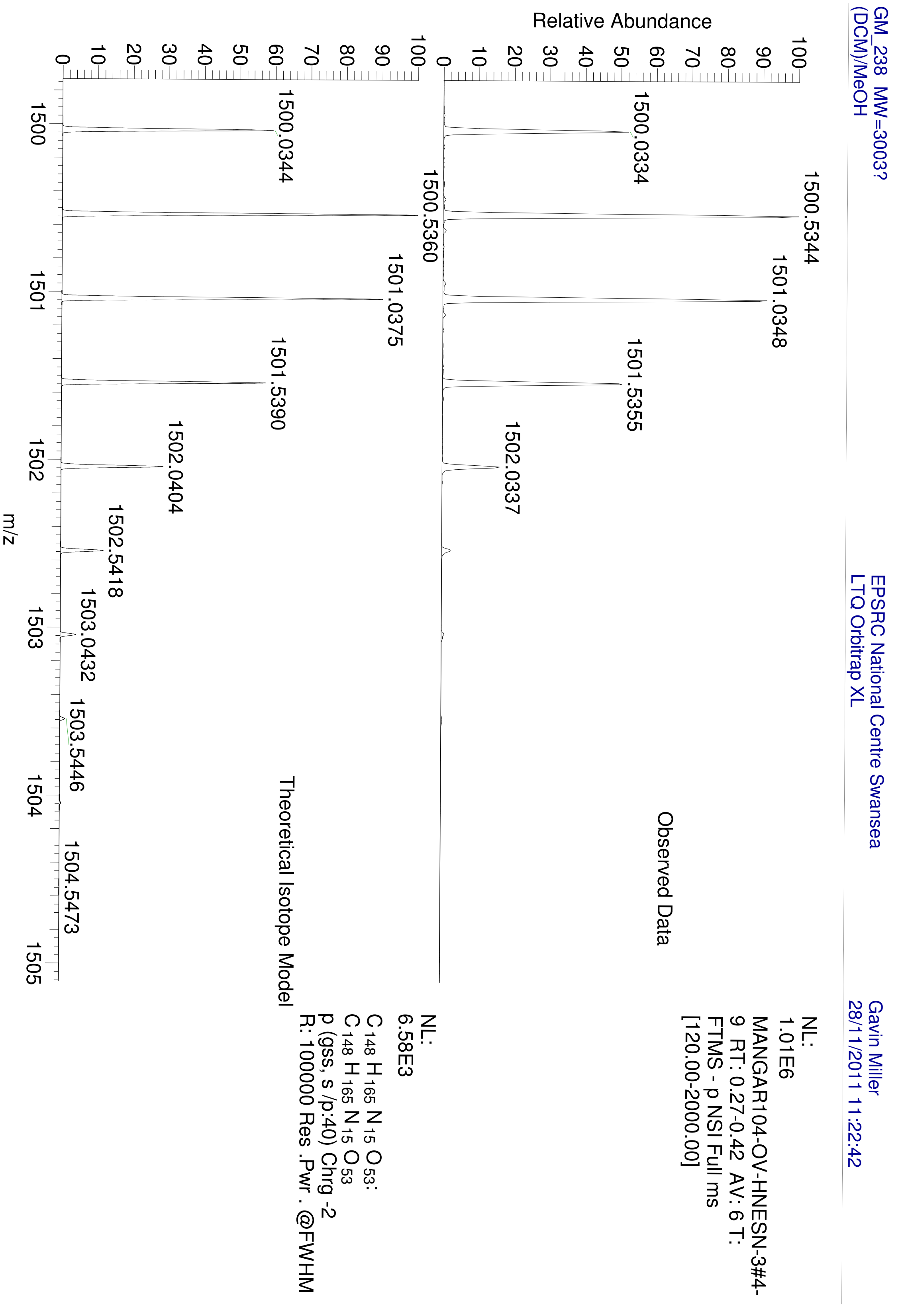

Dodecasaccharide Saponification

^{1}H NMR

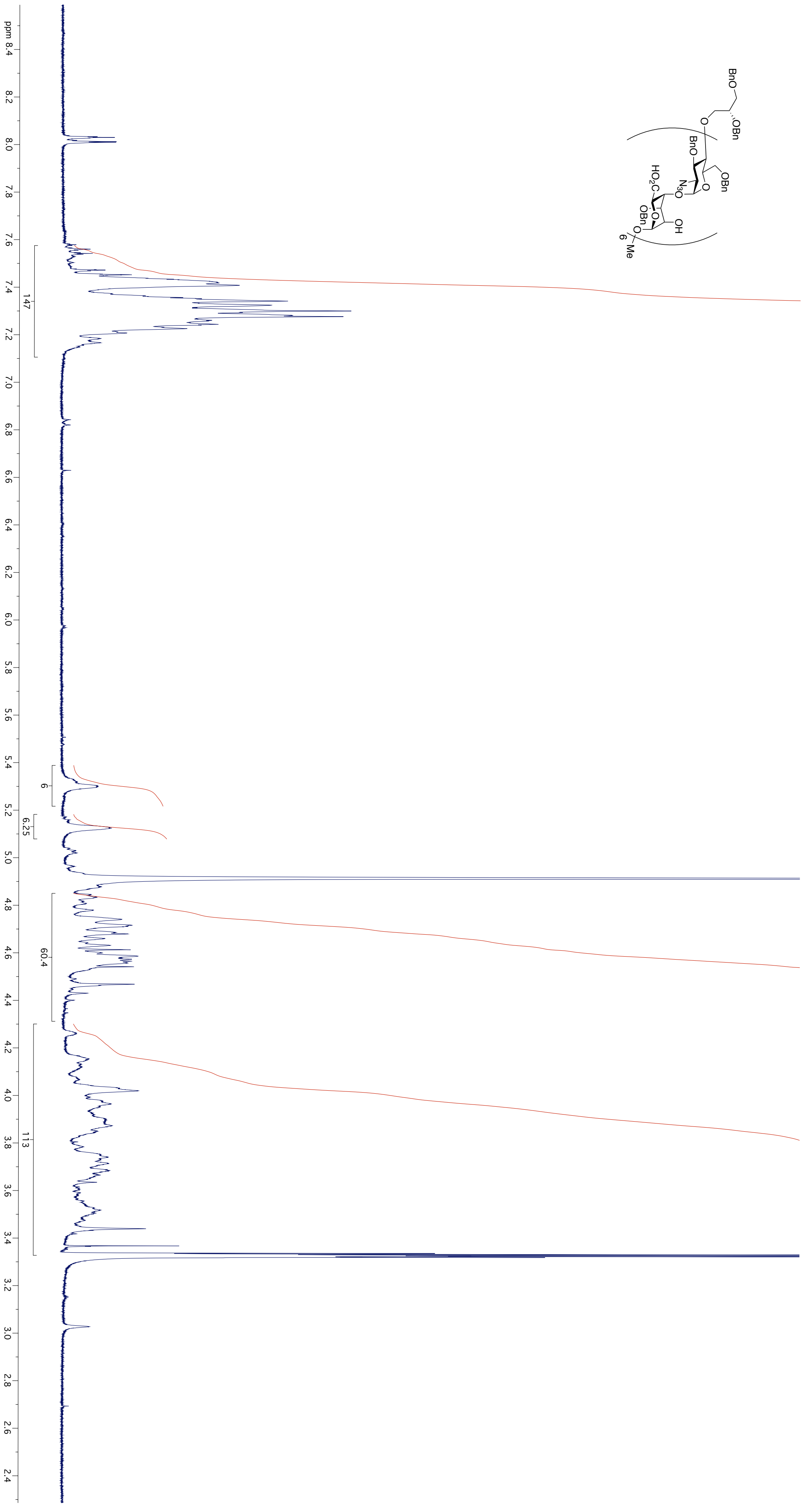

400 MHz, AcOH

Dodecasaccharide Saponification

COSY

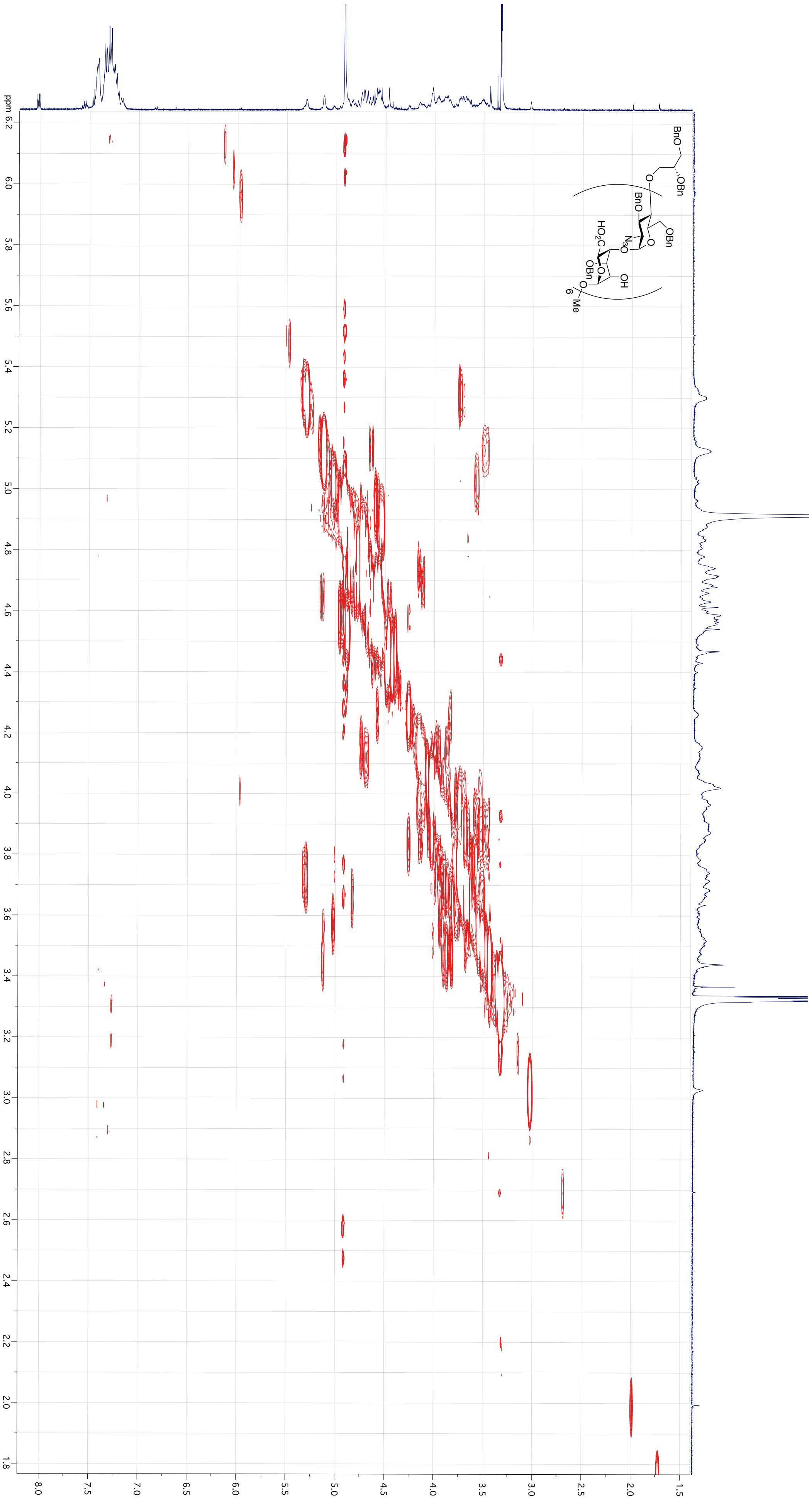

400 MHz, AcOH

Dodecasaccharide Saponification- ESI MS

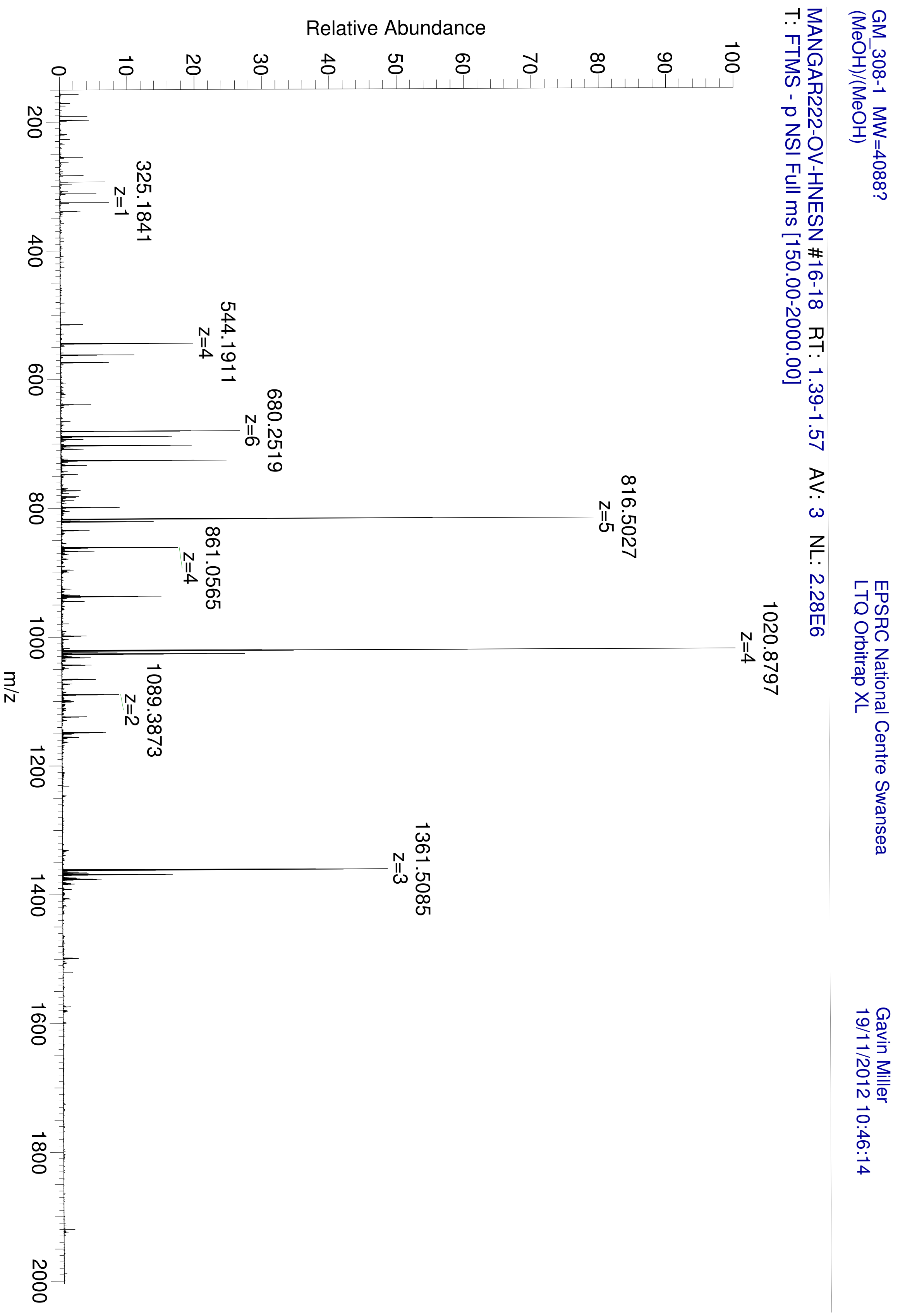

Dodecasaccharide Saponification-**ESI MS Isotope Pattern**

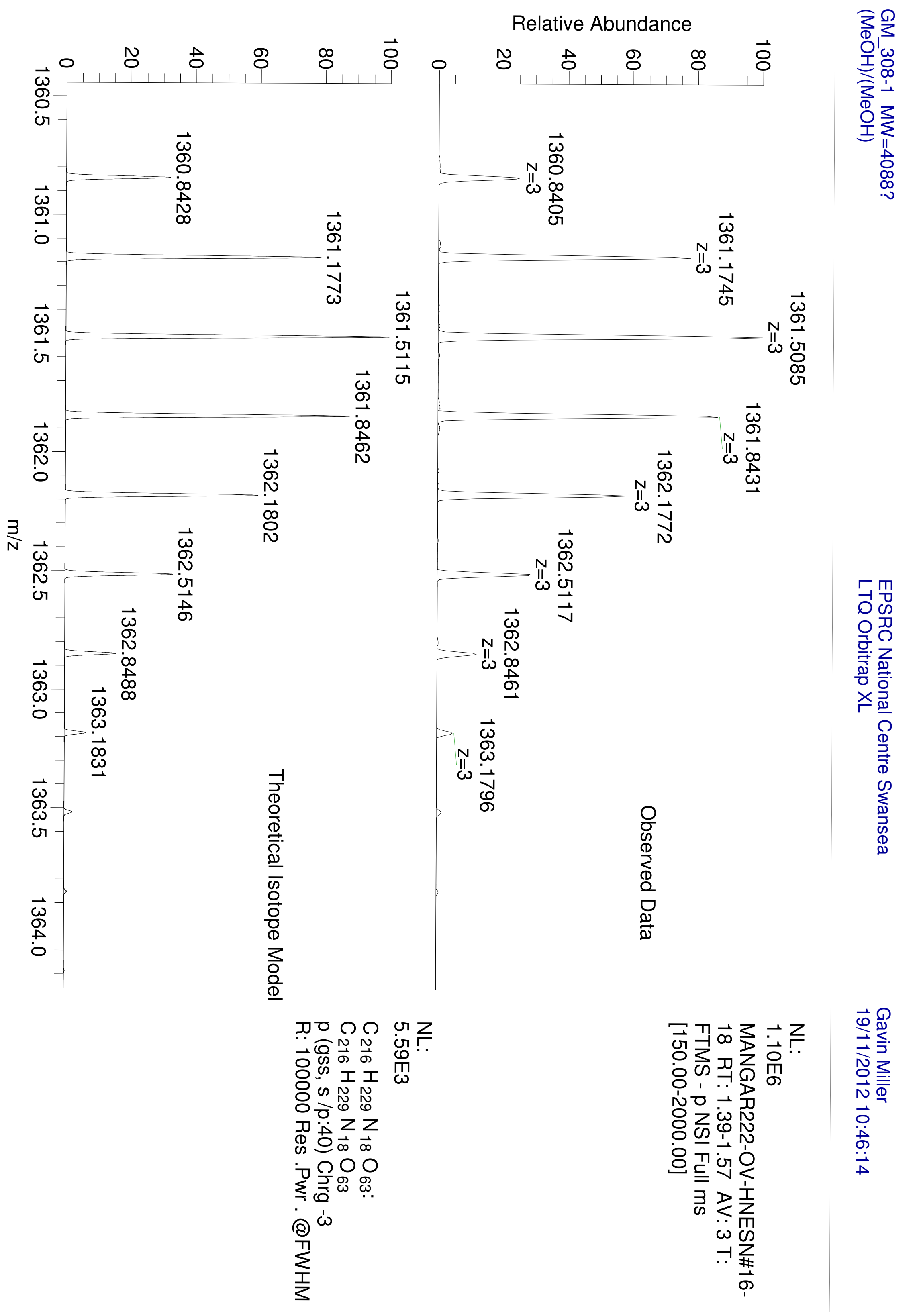

Decasaccharide hydrogenation

^{1}H NMR

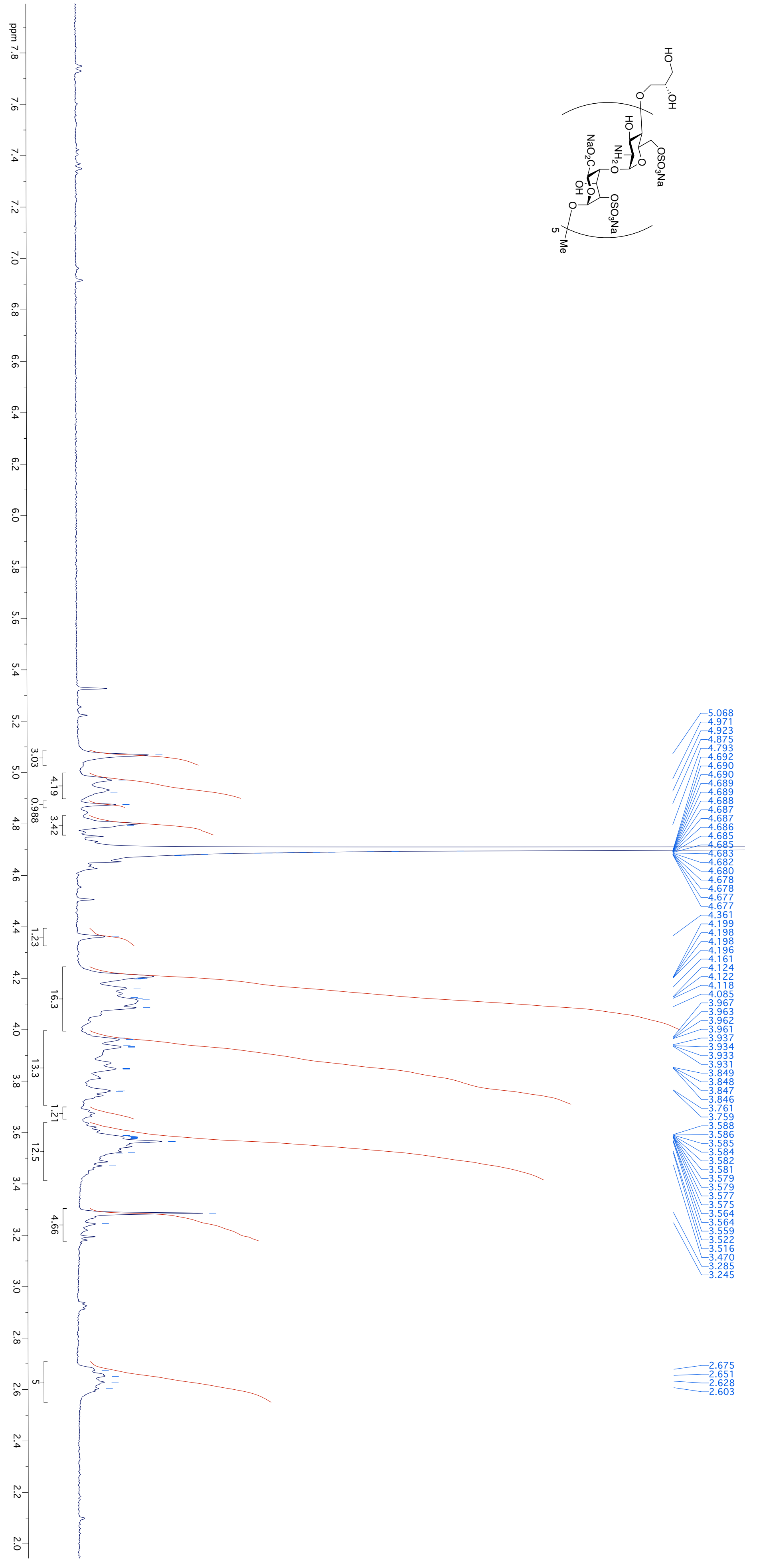

400 MHz, D_2O

Decasaccharide hydrogenation- ESI MS

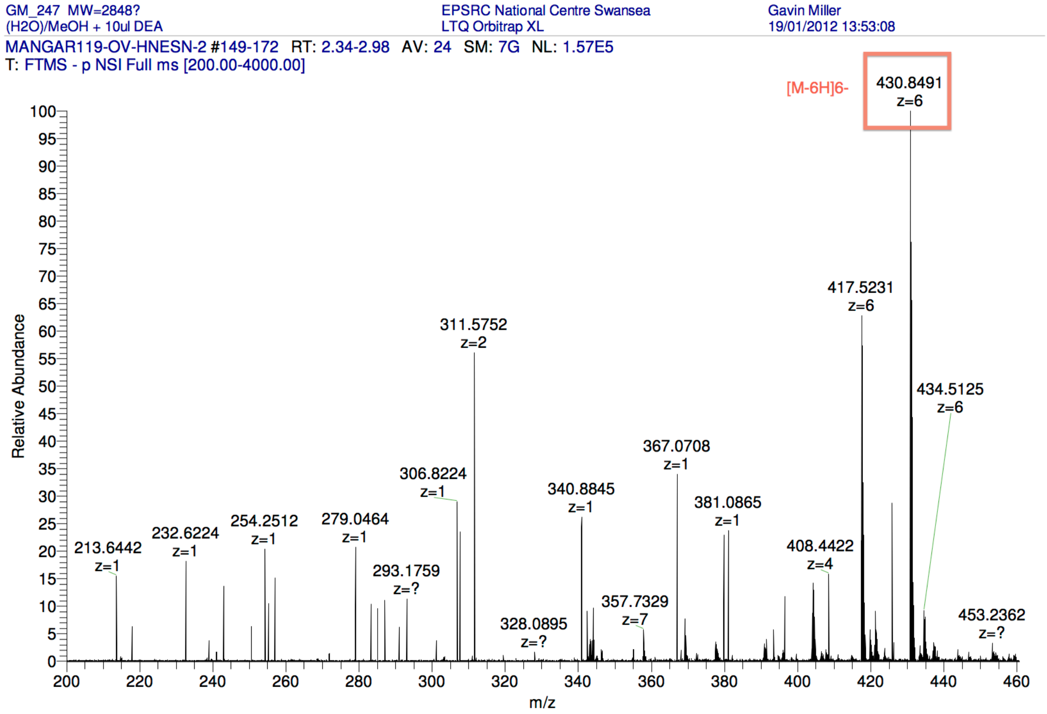

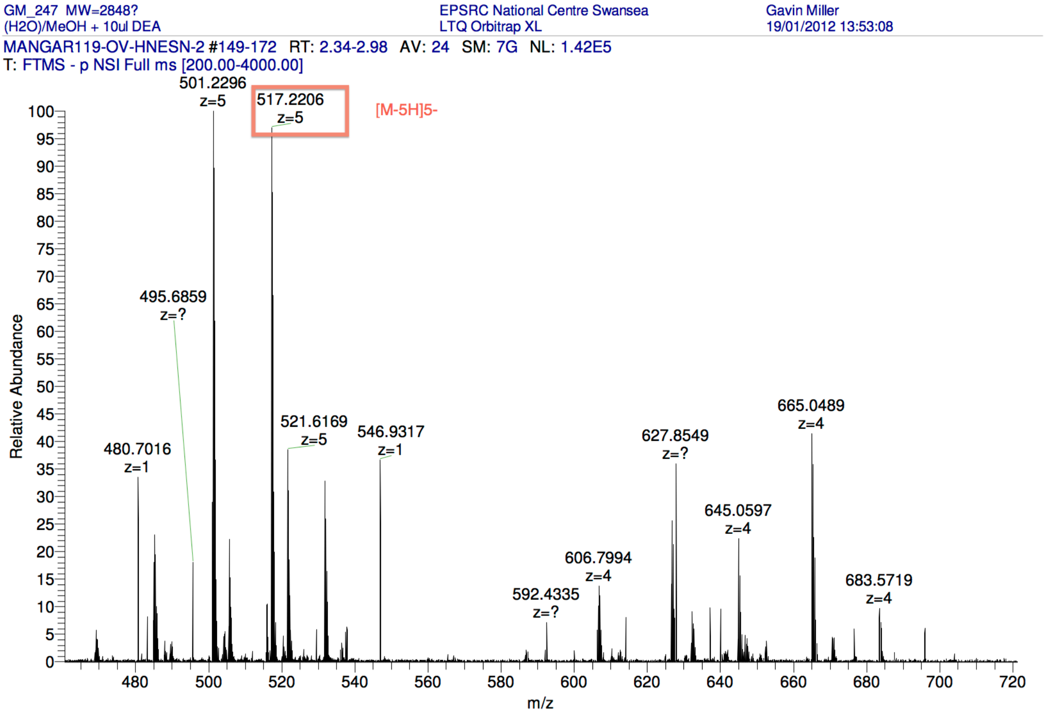

Dodecasaccharide hydrogenation

^{1}H NMR

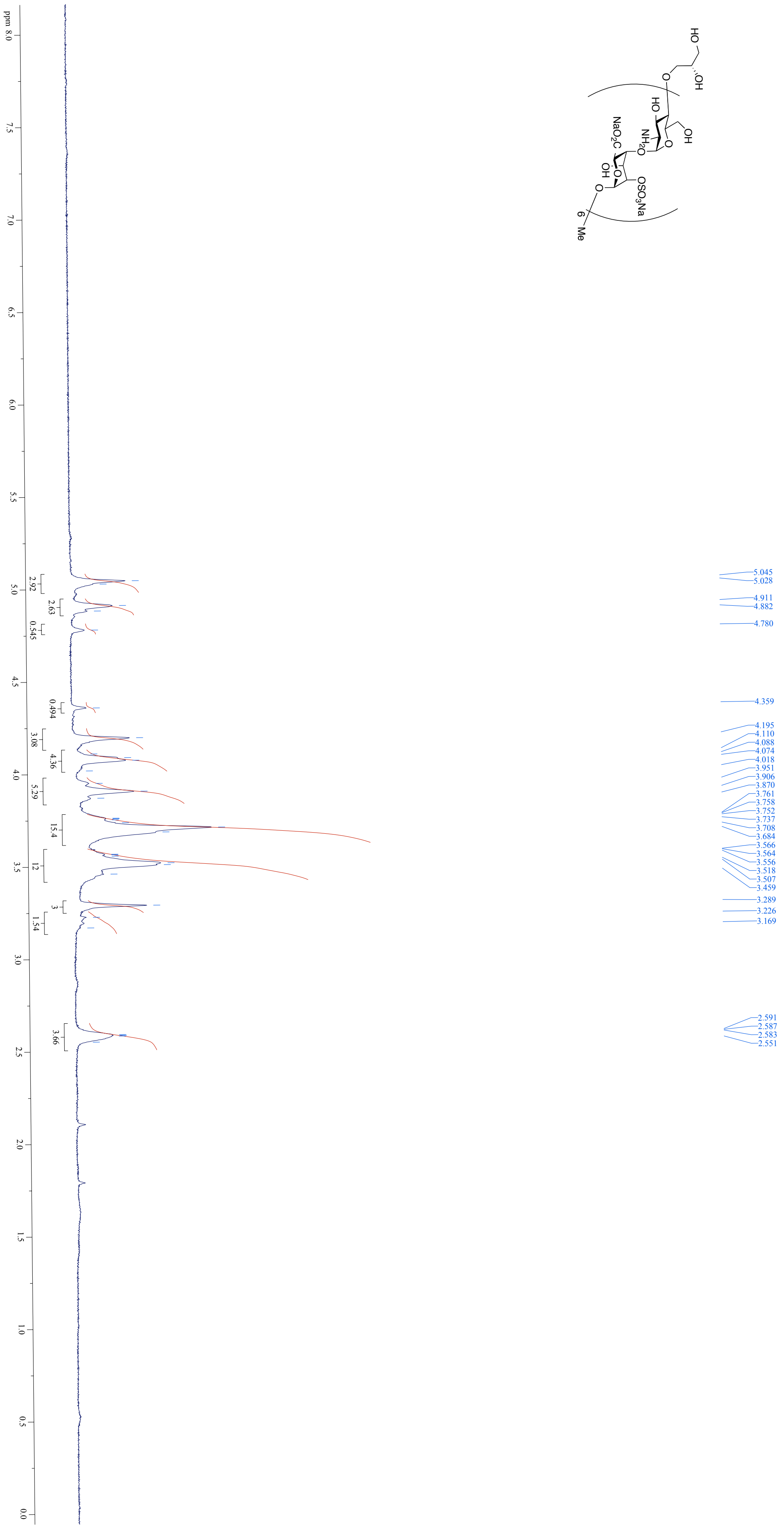

400 MHz, D_2O

Dodecasaccharide hydrogenation- ESI MS

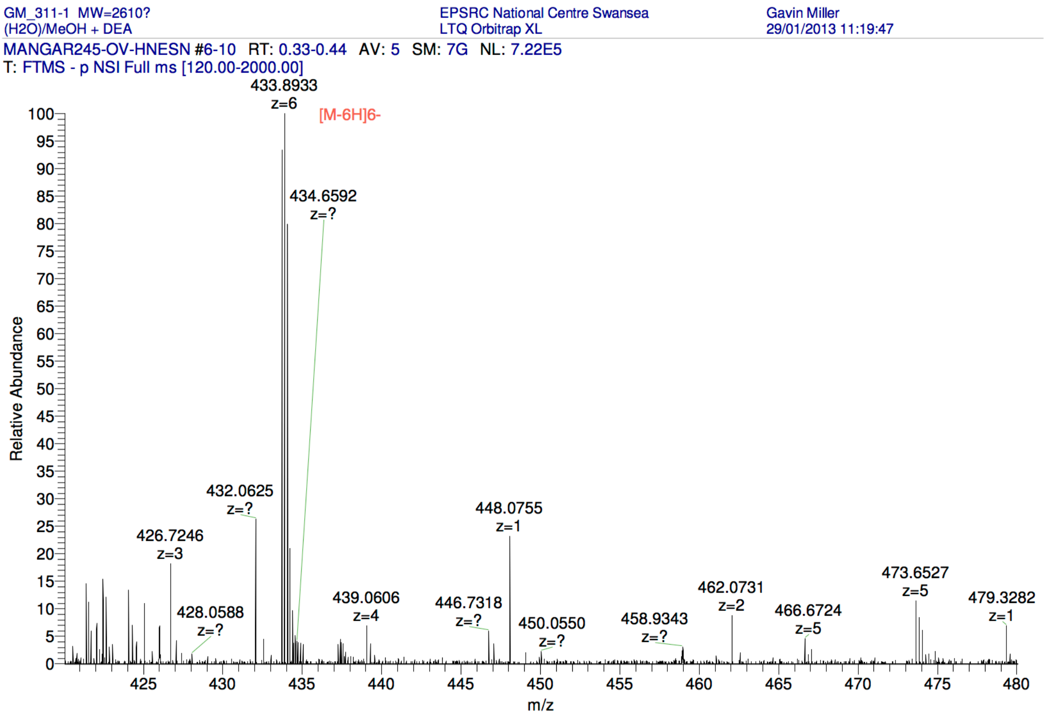

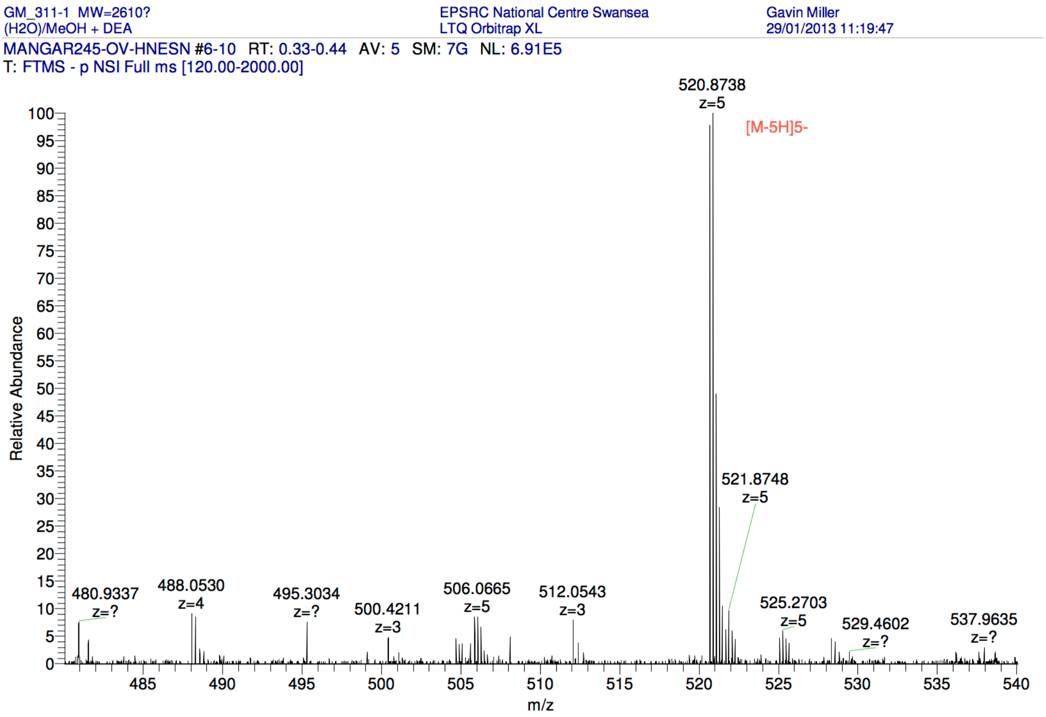

Dodecasaccharide hydrogenation- ESI MS isotope patterns

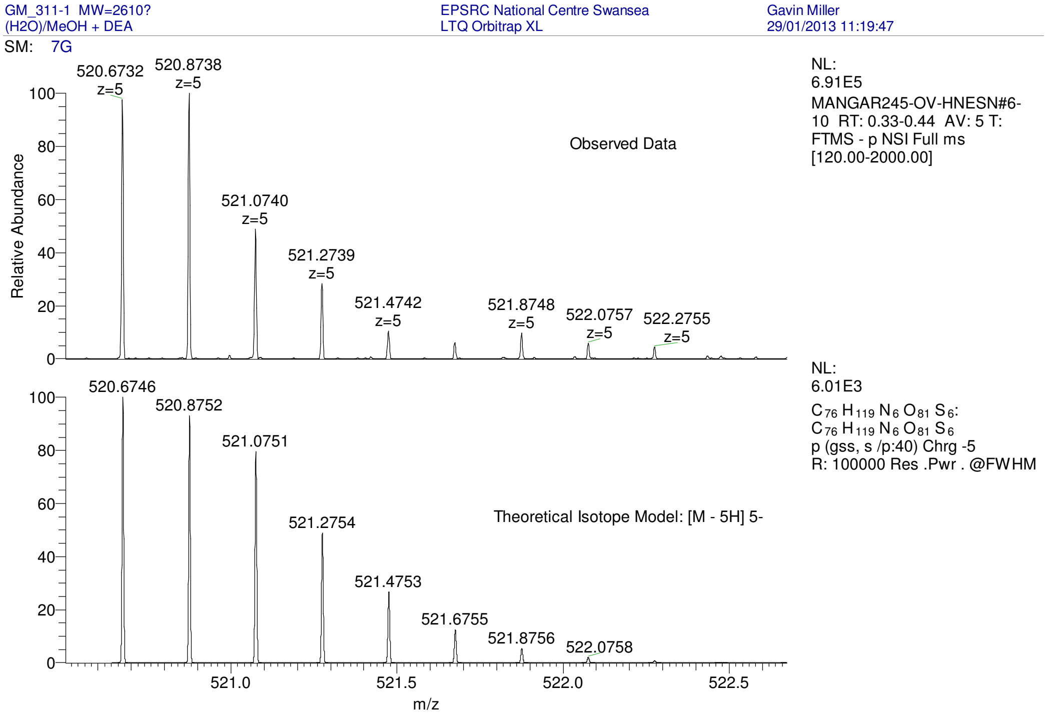

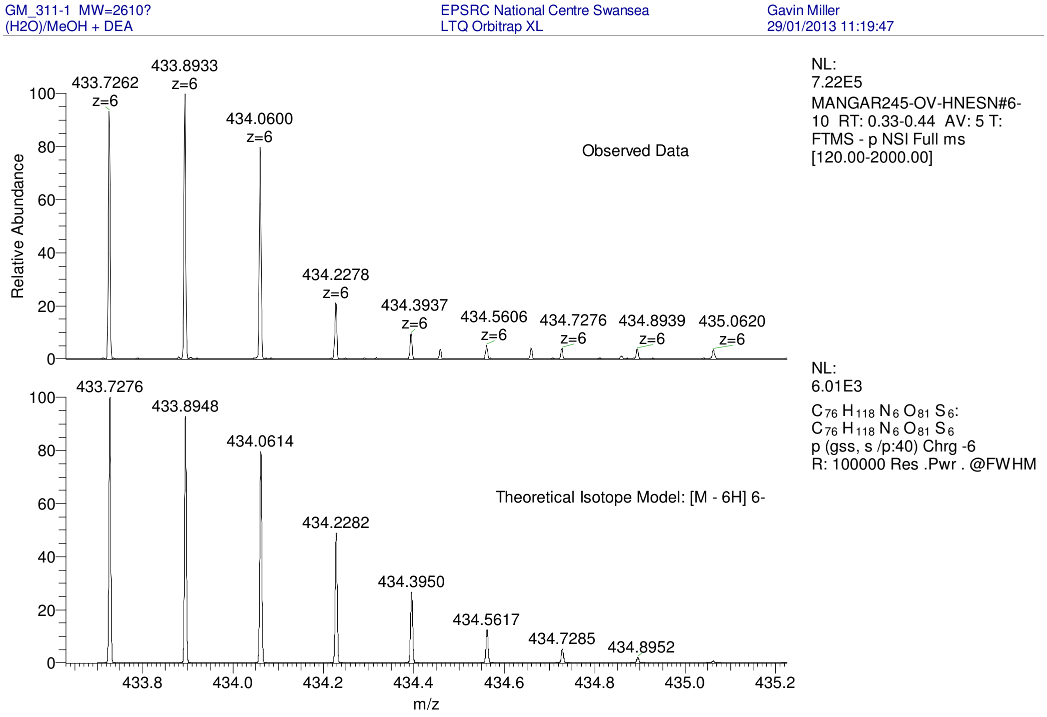

Dodecasaccharide N-sulfation

^{1}H NMR

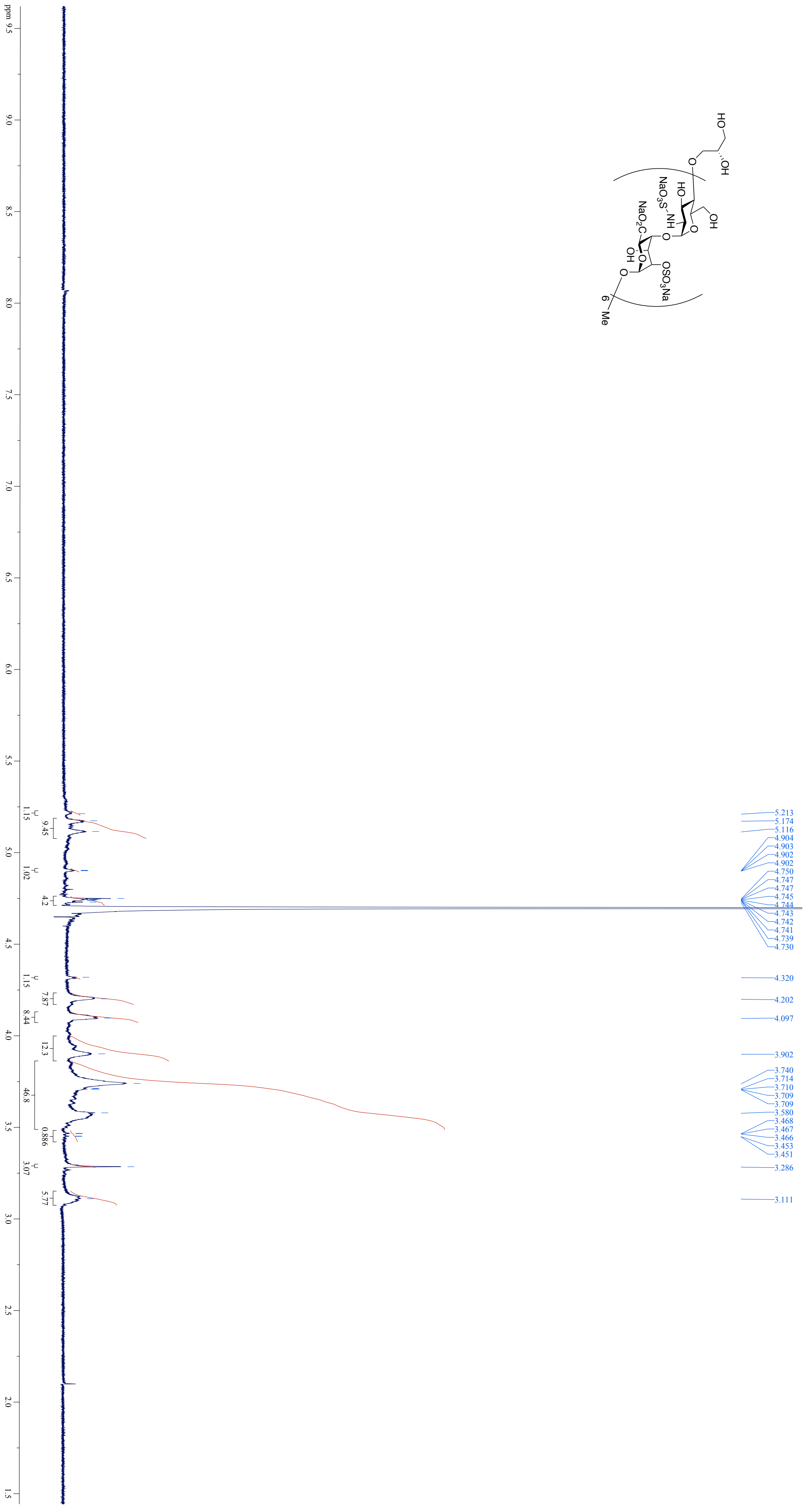

400 MHz, D_2O

Dodecasaccharide N-sulfation- ESI MS

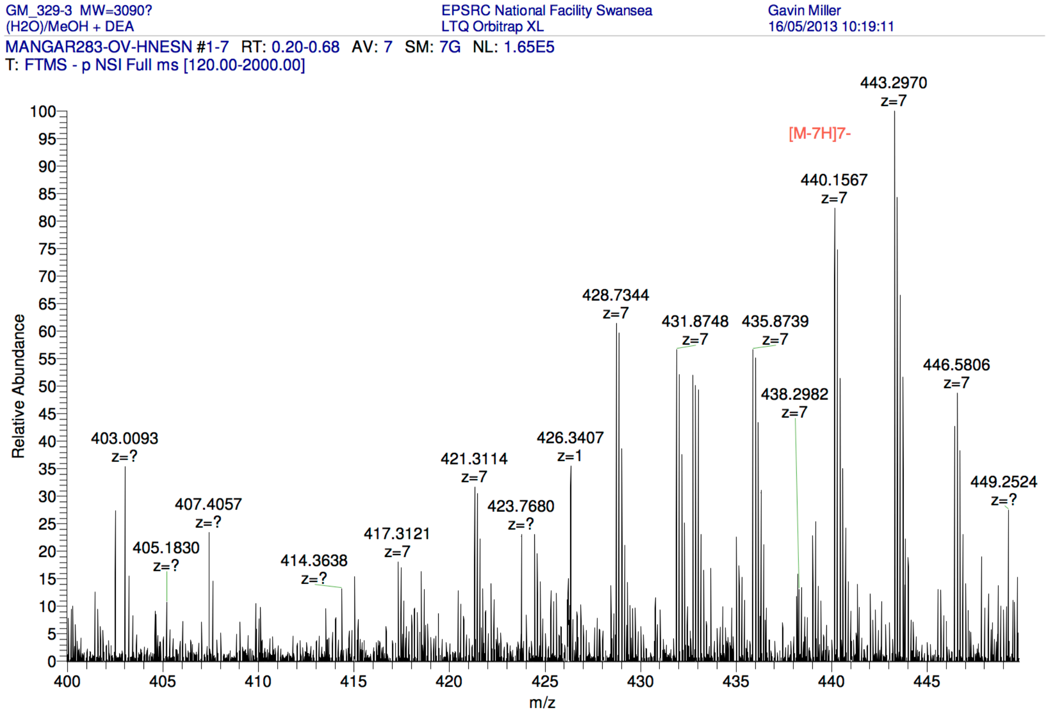

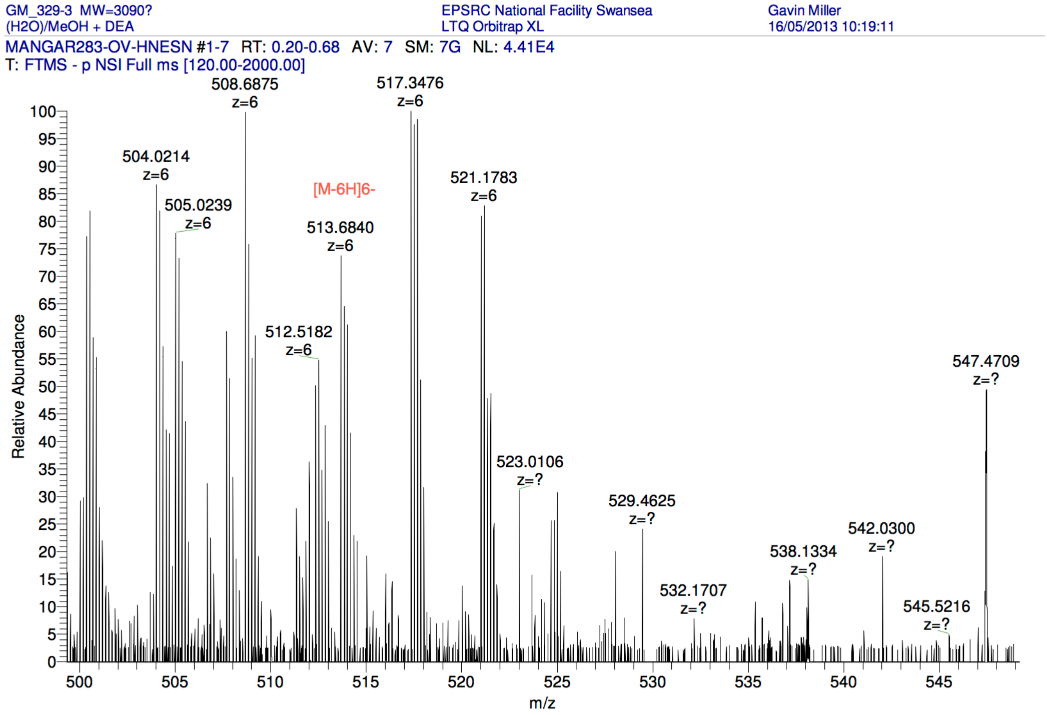

Dodecasaccharide N-sulfation- ESI MS isotope patterns

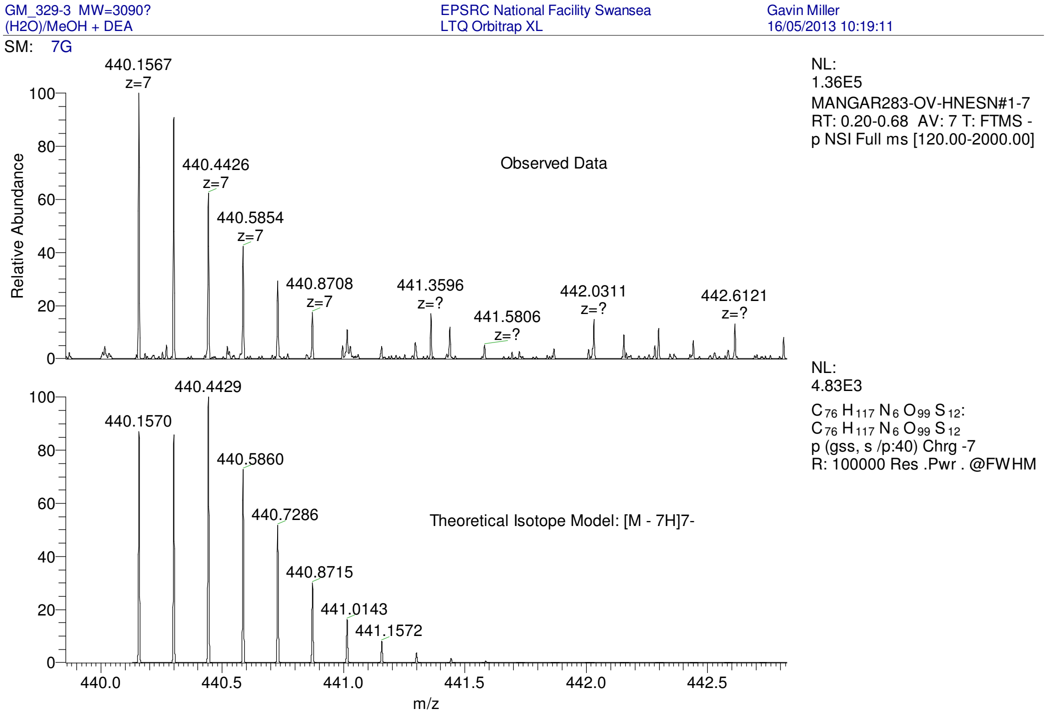

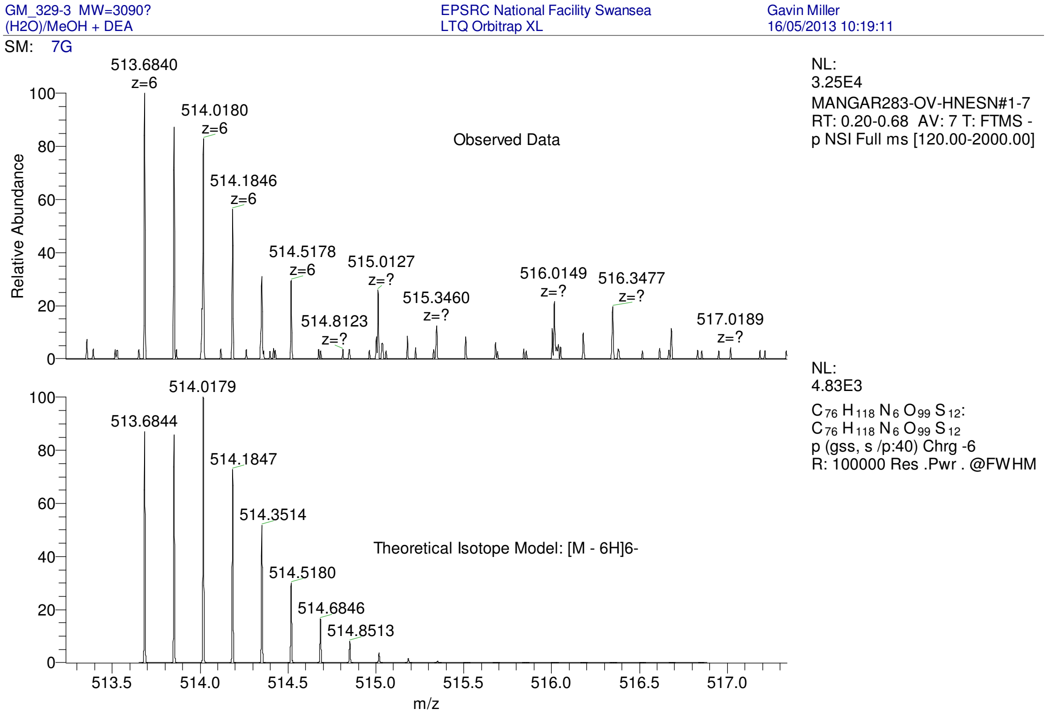

Decasaccharide periodate cleavage product 27

^{1}H NMR

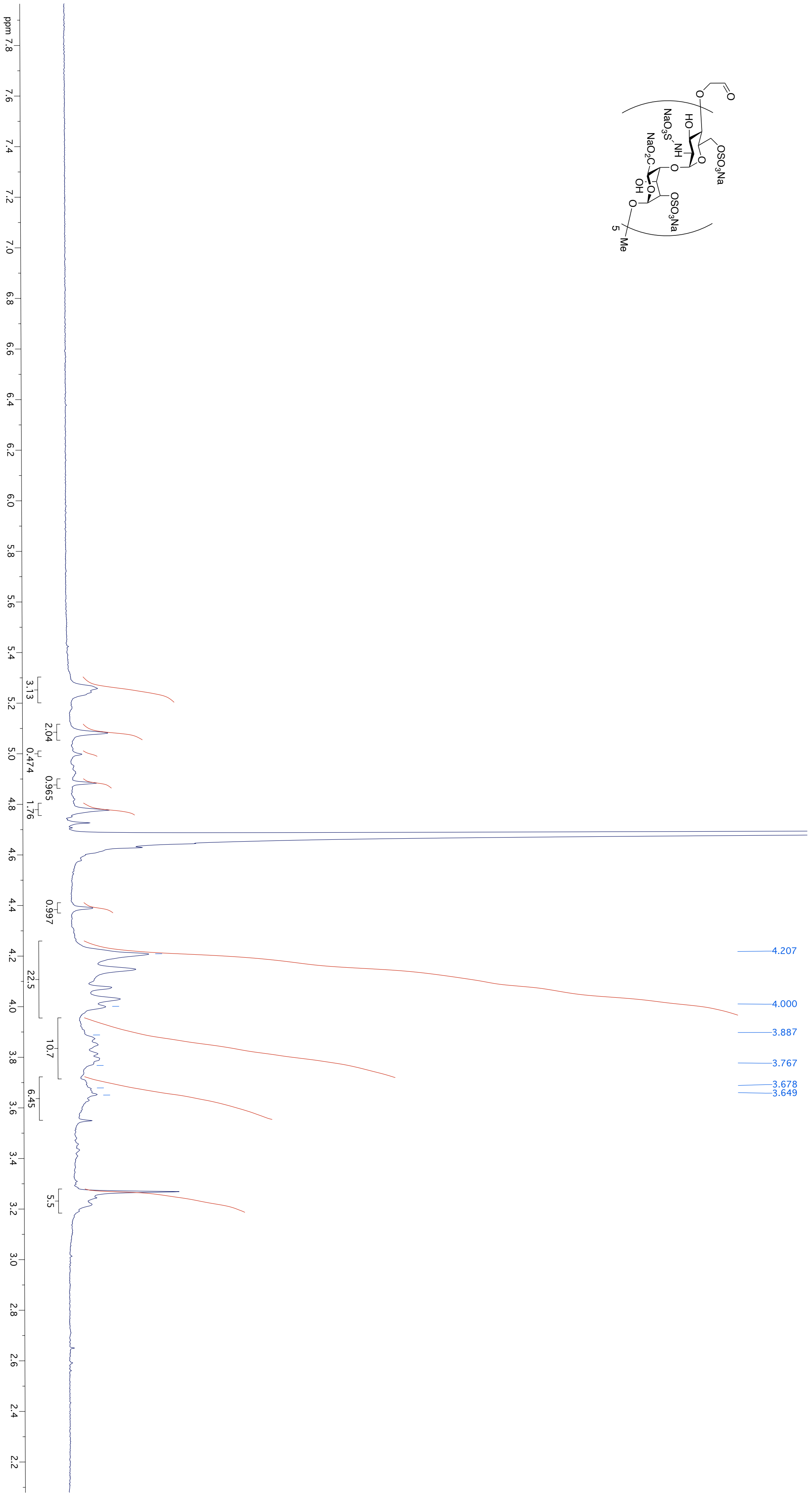

400 MHz, D_2O

Decasaccharide periodate cleavage product 27- ESI MS

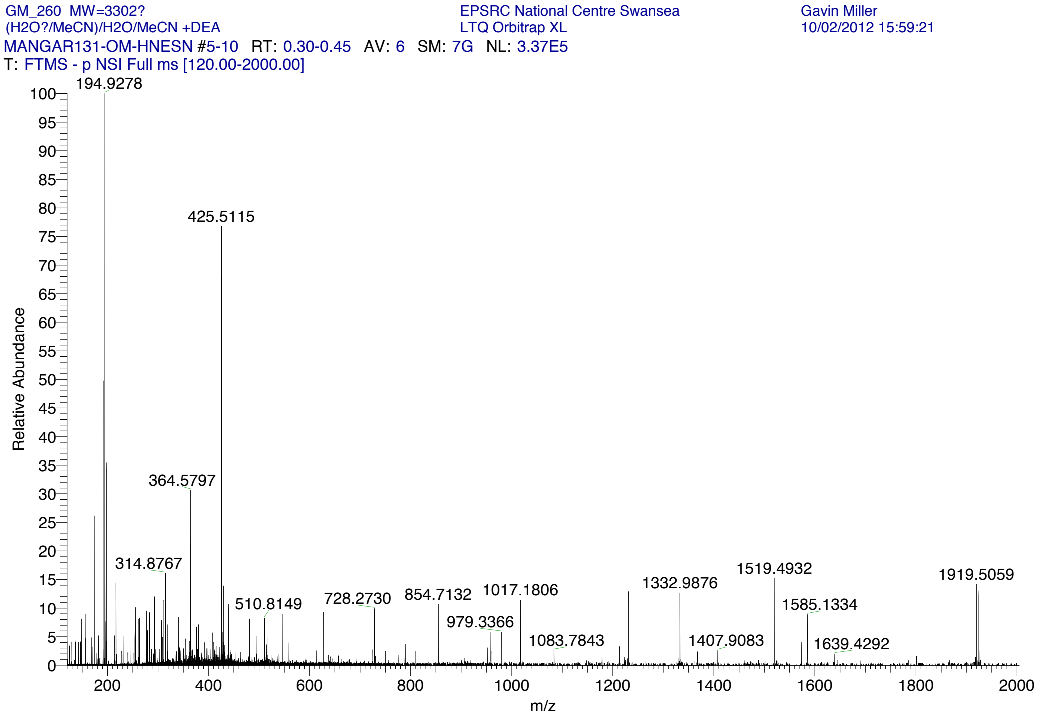

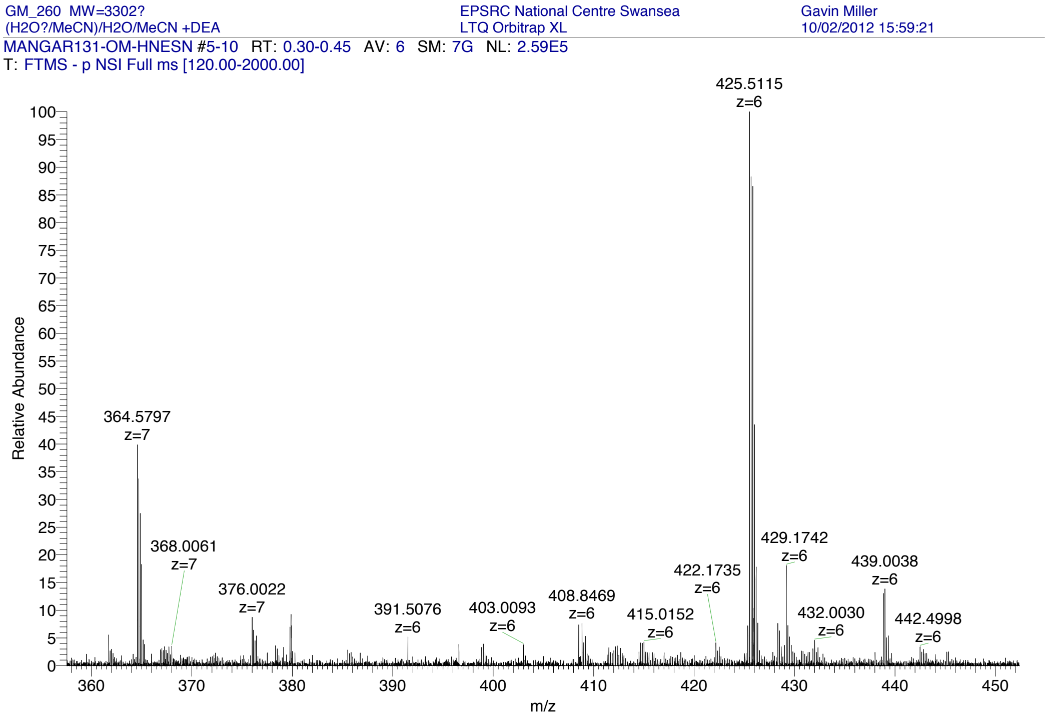

Dodecasaccharide periodate cleavage product 26

^{1}H NMR

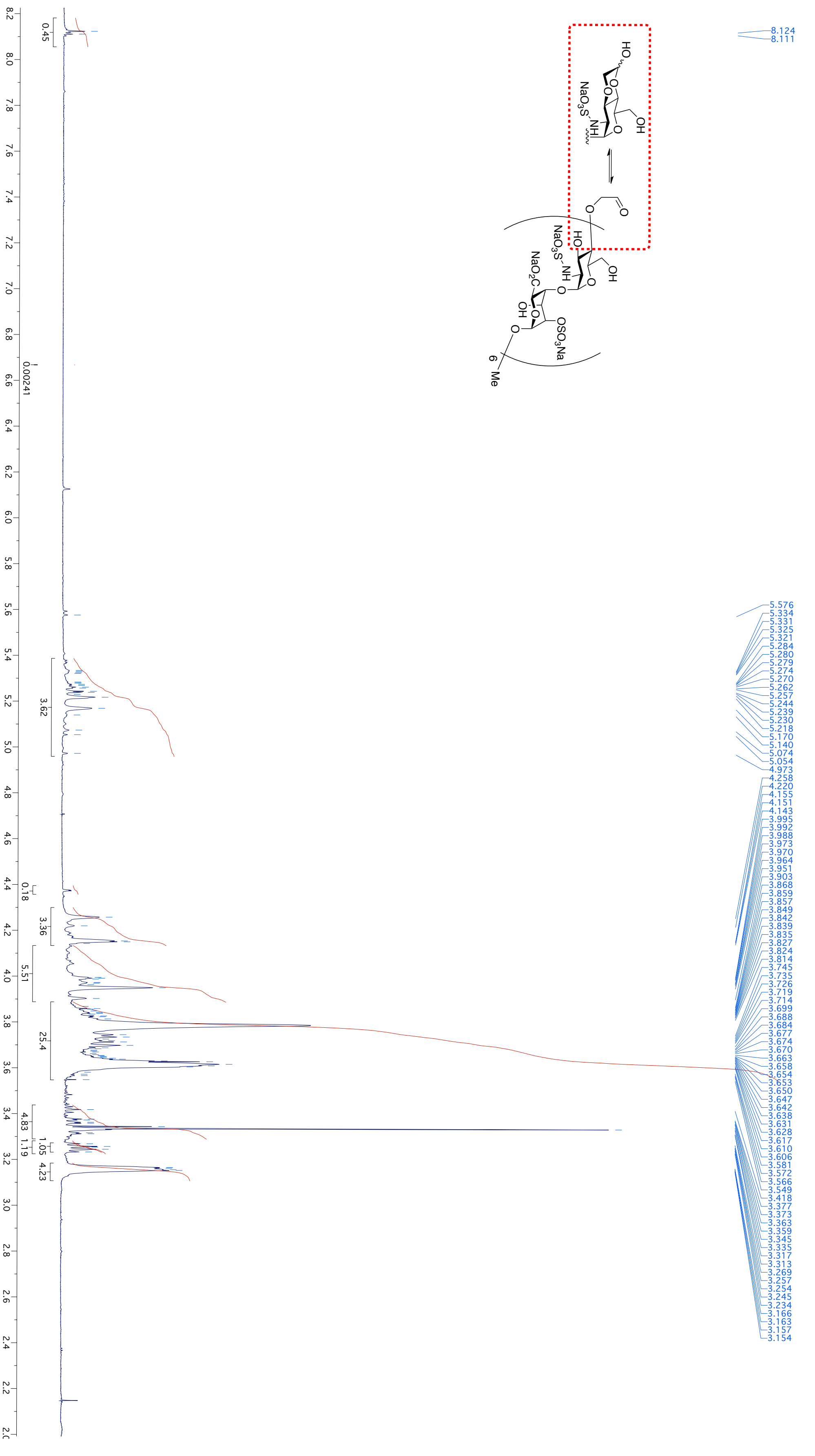

800 MHz, $CDCl_3$

Dodecasaccharide periodate cleavage product 26

COSY

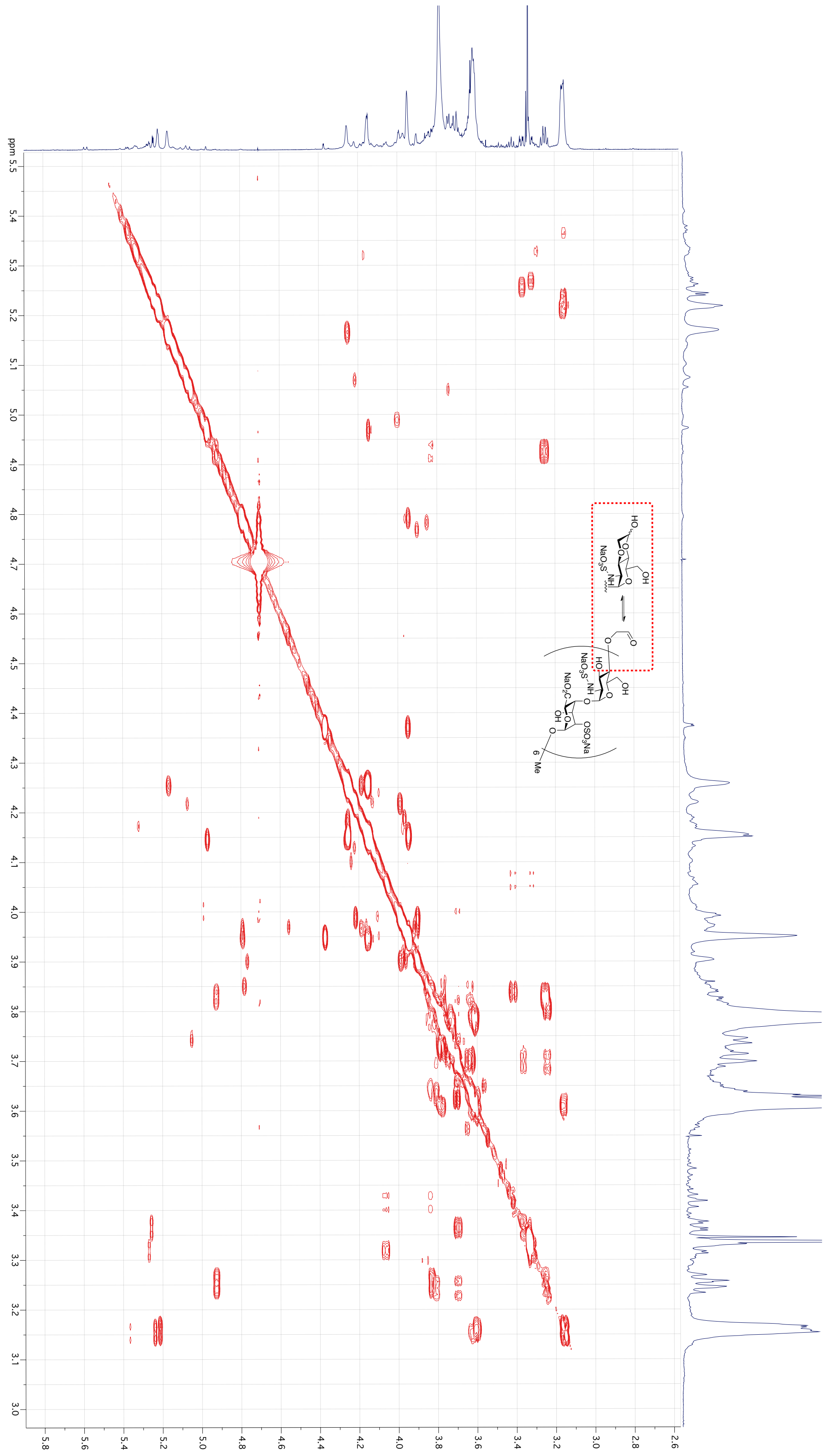

800 MHz, D_2O

Dodecasaccharide periodate cleavage product 26

HSQC

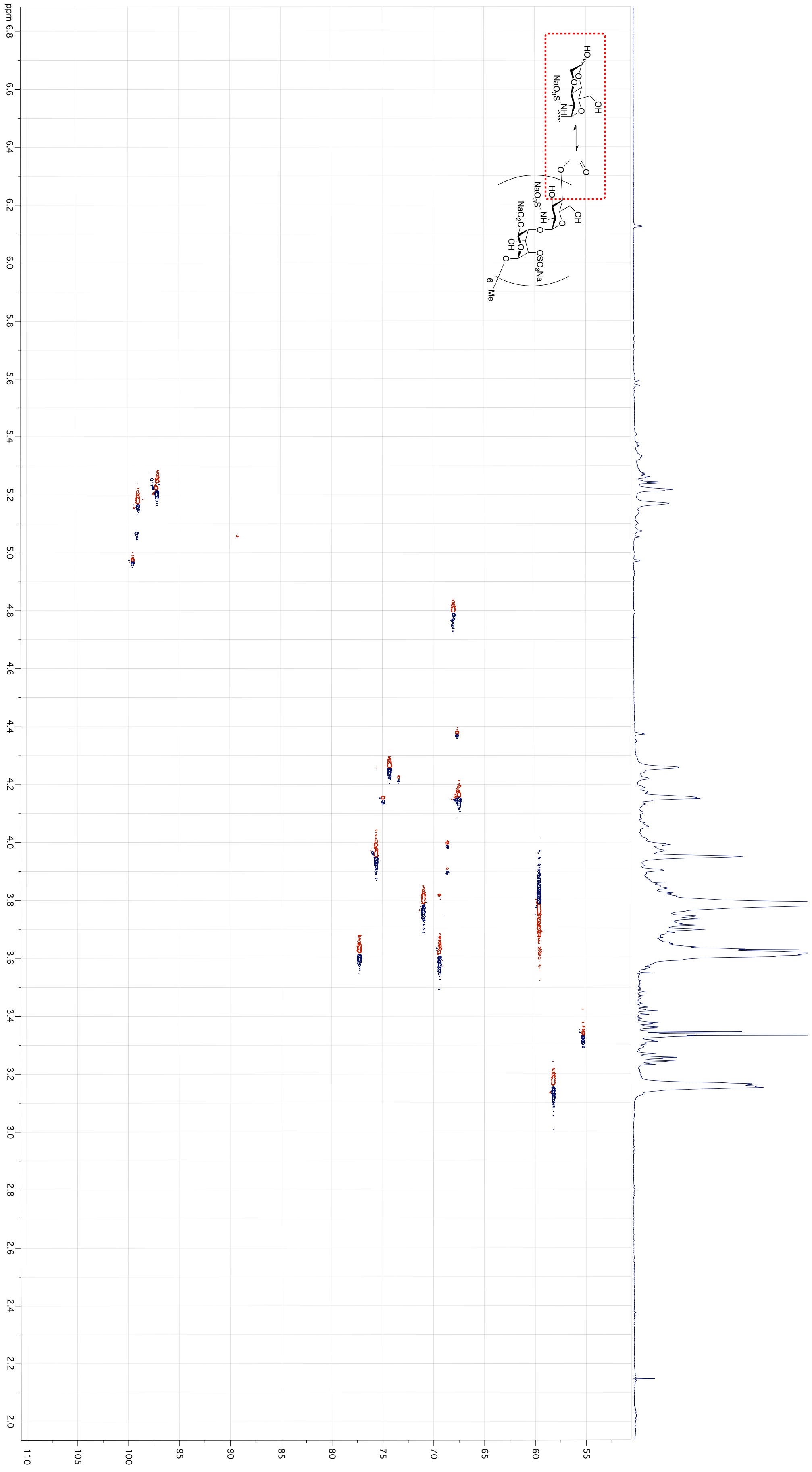

800 MHz, D_2O

Dodecasaccharide periodate cleavage product 26- ESI MS

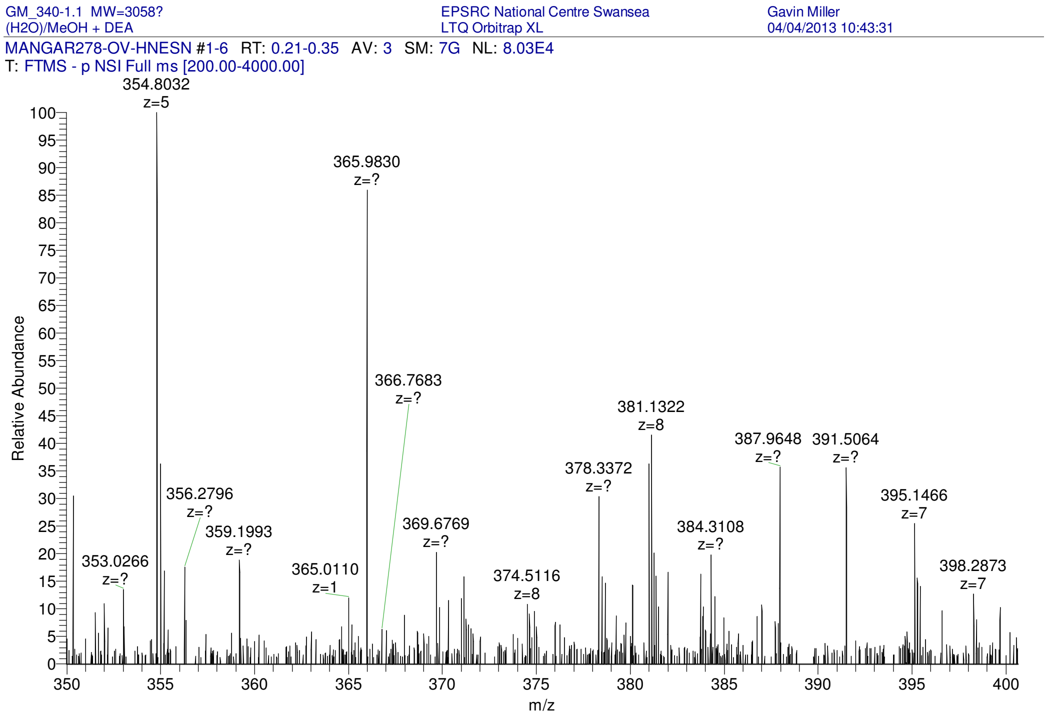

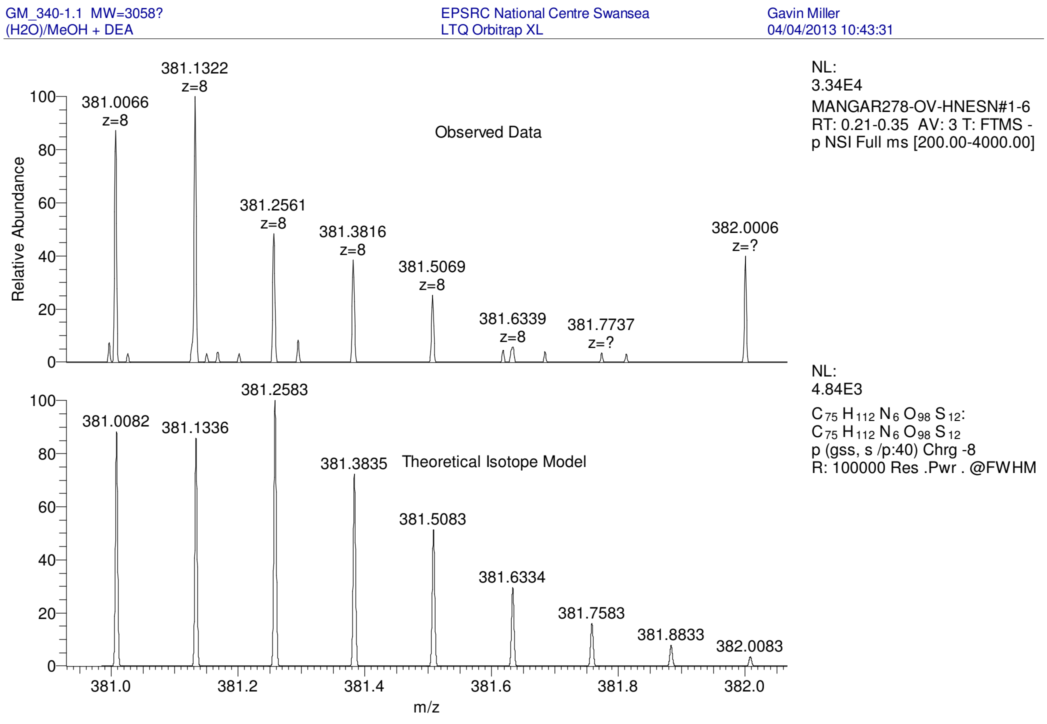

Supplement: Supplementary file 1 [file OB-013-C5OB01706H-s001.pdf]
